# Diauxic lags explain unexpected coexistence in multi‐resource environments

**DOI:** 10.15252/msb.202110630

**Published:** 2022-05-04

**Authors:** Blox Bloxham, Hyunseok Lee, Jeff Gore

**Affiliations:** ^1^ Physics of Living Systems Department of Physics Massachusetts Institute of Technology Cambridge MA USA

**Keywords:** coexistence, community assembly, diauxie, fitness tradeoffs, resource competition, Evolution & Ecology, Microbiology, Virology & Host Pathogen Interaction

## Abstract

How the coexistence of species is affected by the presence of multiple resources is a major question in microbial ecology. We experimentally demonstrate that differences in diauxic lags, which occur as species deplete their own environments and adapt their metabolisms, allow slow‐growing microbes to stably coexist with faster‐growing species in multi‐resource environments despite being excluded in single‐resource environments. In our focal example, an *Acinetobacter* species (Aci2) competitively excludes *Pseudomonas aurantiaca* (Pa) on alanine and on glutamate. However, they coexist on the combination of both resources. Experiments reveal that Aci2 grows faster but Pa has shorter diauxic lags. We establish a tradeoff between Aci2’s fast growth and Pa’s short lags as their mechanism for coexistence. We model this tradeoff to accurately predict how environmental changes affect community composition. We extend our work by surveying a large set of competitions and observe coexistence nearly four times as frequently when the slow‐grower is the fast‐switcher. Our work illustrates a simple mechanism, based entirely on supplied‐resource growth dynamics, for the emergence of multi‐resource coexistence.

## Graphical abstract

*Pseudomonas aurantiaca* unexpectedly survives in coculture in a two‐resource environment despite being excluded in the single‐resource environment. This stable coexistence between a fast‐grower and a fast‐switcher is explained by a tradeoff between growth rate and diauxic lag time.

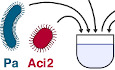

## Introduction

Explaining the rich biodiversity observed in nature and the mechanisms by which species coexist are central questions in microbial ecology (Gauss, 1934; Hardin, 1960; Hutchinson, 1961; Friedman *et al*, 2017). Microbes exist in complex environments and often have multiple resources to choose between. It is therefore important to study coexistence from the perspective of how microbes interact with their environments, including which resources they consume and how they consume them (You *et al*, 2013; Adkar *et al*, 2017; Posfai *et al*, 2017; Goyal *et al*, 2018; Wang *et al*, 2019, 2021; Fu *et al*, 2020; Dal Bello *et al*, 2021; Estrela *et al*, 2022). Previous research has suggested communities grown on a single carbon resource can be used to predict multi‐resource communities (Tilman, 1982; Enke *et al*, 2019; Fu *et al*, 2020; Pacheco *et al*, 2021). There are, however, frequent exceptions, such as a species being excluded in single‐resource communities but coexisting in multi‐resource communities (Tuncil *et al*, 2017; Fu *et al*, 2020). These exceptions suggest dynamics specific to multi‐resource environments must be considered to fully understand community assembly.

Diauxie is a commonly observed phenomenon for microbes growing in a multi‐resource environment (Monod, 1966; Harder & Dijkhuizen, 1982; Görke & Stülke, 2008; Tuncil *et al*, 2017; McGill *et al*, 2021). Diauxie was first observed in yeast in 1900 (Dienert, 1900) and then studied in greater detail in *E. coli* by Monod beginning in 1940 (Monod, 1941, 1949). When grown in a media containing two sugars, *E. coli* first displays exponential growth, then exhibits a period of little or no growth, and then resumes growing. In these cases, *E*. *coli* is consuming one sugar at a time (Monod, 1949). For example, when presented with glucose and xylose, *E. coli* consumes only glucose until glucose runs out and then switches to xylose. In between growth on glucose and growth on xylose are approximately 2 h in which *E. coli* displays no growth (Monod, 1966). This period is known as a diauxic lag and occurs when a microbe needs to reconfigure its metabolism before continuing to grow (Venturelli *et al*, 2015; Wang *et al*, 2015; Erickson *et al*, 2017). Depending on the microbe and the combination of resources diauxic lags can last anywhere from a few minutes to several hours (Monod, 1941, 1949, 1966; Loomis & Magasanik, 1967; Basan *et al*, 2020).

Diauxie is often contrasted to co‐utilization, a metabolic strategy in which a microbe consumes multiple resources at the same time. However, co‐utilizing microbes experience the same resource depletions as specializing species and also need to readjust their metabolisms (Erickson *et al*, 2017; Perrin *et al*, 2020). Lag phases associated with this readjustment occur, although they may be shorter (Wang *et al*, 2015; Chu & Barnes, 2016; Vermeersch *et al*, 2019), and produce the growth‐lag‐growth pattern characteristic of diauxie (Harder & Dijkhuizen, 1982; New *et al*, 2014; Erickson *et al*, 2017). Due to these similarities, we refer to a lag phase associated with a metabolic readjustment as a diauxic lag regardless of whether the microbe was previously co‐utilizing.

Diauxic lags can have a significant impact on interspecies competition. Two microbes often have very different lags after the same resource depletion (Friesen *et al*, 2004; Spencer *et al*, 2007; Wang *et al*, 2015). Some experiments have even shown correlations between growth rate and lag time such that slow‐growers are more likely to be fast‐switchers (Acar *et al*, 2008; New *et al*, 2014; Chu & Barnes, 2016; Basan *et al*, 2020; Balakrishnan *et al*, 2021). In co‐culture, this tradeoff can create a back‐and‐forth in which a fast‐grower outpaces a slow‐grower before a resource depletion, but the slow‐grower, by being the fast‐switcher, catches up to and even overtakes the fast‐grower after the resource depletion (Friesen *et al*, 2004; New *et al*, 2014; Wang *et al*, 2015). Alternating resource supplies can also drive species to evolve shorter diauxic lags, often producing distinct slow‐switcher and fast‐switcher phenotypes (Friesen *et al*, 2004; Spencer *et al*, 2007; Sandberg *et al*, 2017). These results suggest diauxic lags could be a common source of stable coexistence.

We provide an experimental demonstration of a tradeoff between growth and diauxic lag producing stable coexistence between two species. We investigate the case of an *Acinetobacter* species (Aci2) and *Pseudomonas aurantiaca* (Pa) growing on alanine and glutamate. Despite being the slow‐grower and being competitively excluded by Aci2 in both single‐resource environments, Pa stably coexists with Aci2 in the two‐resource environment. We discover that, while Aci2 is the fast‐grower, Pa is the fast‐switcher. Through modeling and additional experiments, we confirm the tradeoff between growth and diauxic lag as the mechanism for coexistence. We extend our work by surveying additional species and resources and see that coexistence is nearly four times as likely to occur when the slow‐grower is the fast‐switcher. Our research establishes a mechanism for the otherwise unexpected coexistence of species based entirely on growth dynamics on supplied resources and simple tradeoffs between growth and diauxic lag and highlights the importance of diauxic lags on interspecies competition.

## Results

### Pa unexpectedly coexists with Aci2 in a multi‐resource environment

**Figure 1:**
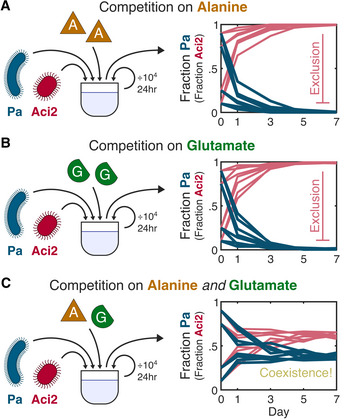
Pa stably coexists with Aci2 when co‐cultured on alanine and glutamate despite being competitively excluded when co‐cultured on either resource alone Pa (*Pseudomonas aurantiaca*) and Aci2 (*Acinetobacter* sp.) were co‐cultured with a 10^4^ dilution every 24 h and 0.1% w/v alanine supplied as the only carbon source. Two biological replicates were performed for each of four initial Pa fractions: 0.1, 0.25, 0.75, and 0.9. Population sizes were measured via colony counting after the first, third, fifth, and seventh days (Materials and Methods). With only alanine supplied, Aci2 competitively excluded Pa.An identical experiment was performed with 0.1% w/v glutamate supplied as the only carbon source. Aci2 again competitively excluded Pa.A third experiment was performed with both 0.05% w/v alanine and 0.05% w/v glutamate supplied. Pa coexisted with Aci2 and reached a population fraction between 0.34 and 0.42 independent of initial starting fraction. Two‐resource coexistence was unexpected given the single‐resource competitive exclusions.

To understand community assembly in multi‐resource environments, we cocultured an *Acinetobacter* species (Aci2) and *Pseudomonas aurantiaca* (Pa) in three environments under batch culture with daily dilution. When Aci2 and Pa were cocultured in an environment containing alanine as the only carbon source, Aci2 competitively excluded Pa (Fig 1A). When cocultured with glutamate as the only carbon source, Aci2 again competitively excluded Pa (Fig 1B). If two‐resource communities were a simple sum of single‐resource communities (Fu *et al*, 2020; Pacheco *et al*, 2021), one would expect Aci2 to competitively exclude Pa in an environment containing alanine and glutamate. Contrary to this expectation, when Aci2 and Pa were cocultured on an equal mix of alanine and glutamate, they stably coexisted (Fig 1C). Across eight replicates Pa had a population fraction between 0.34 and 0.42 that was independent of the starting fraction (Fig 1C). This was an intriguing result with the potential to illuminate mechanisms for coexistence unique to multi‐resource environments.

**Figure EV1:**
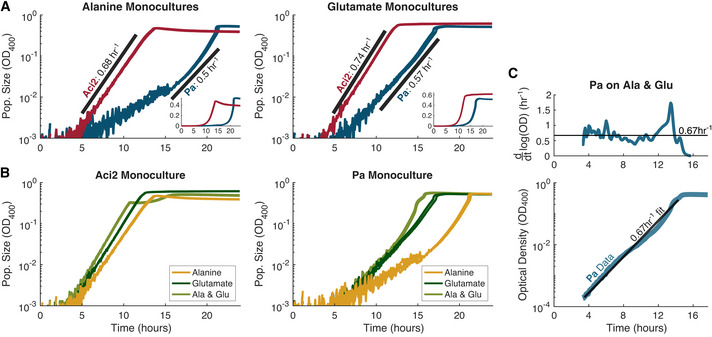
Pa is the single‐resource slow‐grower and should be considered the slow‐grower despite some fluctuations in its apparent growth rate Monoculture growth rate experiments in single resource environments for Aci2 in red and Pa in blue with growth rate fits shown in black. Insets show the same data on a linear scale. On alanine, Pa has a distinct upwards curvature to its growth curve. This could represent a density‐dependent growth rate with a peak instantaneous growth rate close to Aci2’s. Pa should nevertheless be considered the slow‐grower because for the majority of its growth it is growing much slower than Aci2 and its increase in growth rate occurs over only the last ~2 h before it saturates. The value of 0.5/h is representative across its growth.Direct comparisons of each species’ growth in each environment highlights similarity of growth rates across environments and in particular the similarity of the glutamate growth rates to the two‐resource growth rates, which is especially relevant because in competition glutamate is the resource that is most often being switched to.Pa’s two‐resource growth rate fit from Fig 2A extended across the entirety of the data. Although Pa’s measured growth rate has some small fluctuations, the growth rate fit of 0.67/h is a good overall fit. The spike in growth rate around 13 h is discussed in Appendix Fig S5, which also presents reasons why Pa’s optical density may not be a constant function of its biomass or population size and why modeling the small variations would likely be overfitting to experimental artifacts and not actual growth dynamics.

**Figure 2:**
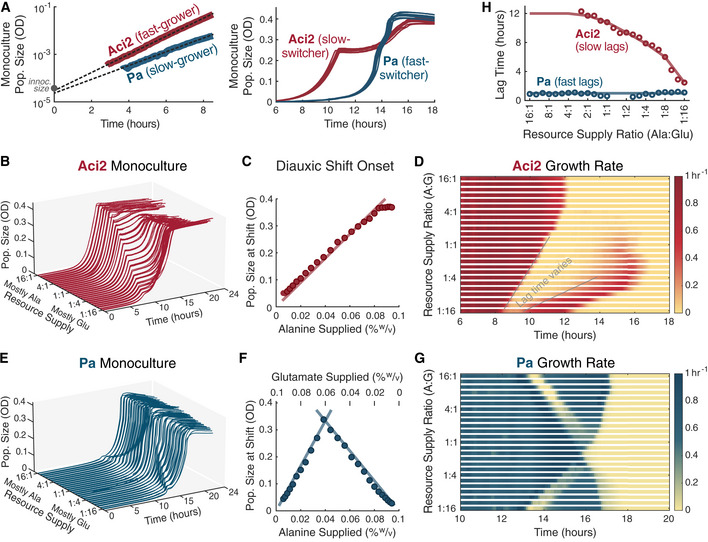
Monoculture growth dynamics reveal Aci2 is a fast‐grower but slow‐switcher whereas Pa is a slow‐grower but fast‐switcher Aci2 and Pa were both grown in monoculture in the two‐resource environment. The same data is shown in both plots. The left plot shows the average of eight technical replicates on a log scale, while the right plot shows each of the replicates on a linear scale. Overlain on the left plot are growth rate fits of *g*_Aci2_ = 0.88/h and *g*_Pa_ = 0.67/h.Aci2 was grown with alanine and glutamate supplied at 25 different ratios from 1:16 to 16:1 with the total supply kept constant at 0.1% w/v (Materials and Methods).The population size at which Aci2’s diauxic shift occurred is linearly correlated to the alanine supply, indicating that Aci2 initially consumes almost entirely alanine.Instantaneous growth rates were extracted from the Aci2 monoculture data (Materials and Methods). A variation in Aci2’s lag time is clearly visible in this representation. An alternative visualization in Appendix S6 plots growth rate on a z‐axis to provide a better visualization of the shape of the growth rate recoveries as functions of time.The same monoculture experiments were performed for Pa. The appearance of Pa having a long initial lag is primarily due to the growth that it needs to accomplish before its population size becomes significant on a linear scale as well as a small optical density artifact (Appendix Fig S5).Pa’s population size at the onset of its diauxic shift correlates to the supply concentration of whichever resource is supplied in a more limiting amount, indicating coutilization.Instantaneous growth rates extracted from the Pa monoculture data.Diauxic lag times were fit from the monoculture data (Materials and Methods) and are plotted as circles. Lag times could not be fit for conditions with too little growth on the remaining glutamate. The lag times used in the modeling are shown as lines through the data. Data Information: More detail on the Pa growth rate fit is provided in Fig EV1C. Data in A were collected at 400 nm. Data in B–G were collected at 600 nm. Example lag time fits are shown in Fig EV2A, and the full set in Appendix Figs S3 and S4. Source data are available online for this figure.

We began our investigation into the coexistence of Aci2 and Pa by ruling out one of the simplest explanations. Species generally grow faster as the number of available resources increases (Hermsen *et al*, 2015; Taheri‐Araghi *et al*, 2015). We hypothesized that Aci2 might be the fast‐grower in both single‐resource environments while Pa might be the fast‐grower in the two‐resource environment. We measured growth rates and saw that Aci2 was the fast‐grower in both single‐resource environments (Fig EV1A), which explained why Aci2 excluded Pa in those cocultures. However, we also saw that Aci2 was the fast‐grower in the two‐resource environment, with a growth rate 31 ± 2% faster than Pa’s (0.88 ± 0.01/h versus 0.67 ± 0.01/h) (Fig 2A and Appendix Fig S1). That Aci2 was the consistent fast‐grower made it even more surprising that Pa had coexisted with Aci2 in the alanine‐glutamate environment.

### Aci2 is the fast‐grower, but Pa is the fast‐switcher

The growth rate experiments did, however, contain a hint toward another explanation. Aci2 had a several‐hour diauxic lag around half‐saturation, whereas Pa had a short diauxic lag lasting no more than an hour (Fig 2A). Aci2 was the fast‐grower but slow‐switcher, whereas Pa was the slow‐grower but fast‐switcher. We hypothesized that a tradeoff between these properties could be the source of coexistence.

To test whether Pa’s short lag could explain its coexistence with Aci2, we first needed a more detailed understanding of each species. To determine which resource each species consumed first, we varied the resource supply ratio in our monoculture experiments. The population density at which Aci2 displayed its diauxic shift was proportional to the alanine supply concentration (Fig 2B and C), such that the amount Aci2 could grow before its diauxic shift directly matched the amount of alanine available, indicating that Aci2 first consumes almost entirely alanine.

In contrast to Aci2’s initial single‐resource consumption, Pa’s dynamics showed clear signs of co‐utilization. At a low supply fraction of glutamate, Pa had a diauxic lag that occurred at larger population sizes with increasing glutamate supply. But at a low alanine supply, Pa’s lag occurred at larger population sizes with increasing alanine supply (Fig 2E and F). As a supply ratio of 2:3 alanine:glutamate was approached, the population density at which Pa’s diauxic shift occurred converged toward the saturation density, such that near a 2:3 supply ratio Pa simply saturated with no diauxic shift. These observations indicated that Pa consumed alanine and glutamate in a fixed ratio of approximately 2:3 when growing in the two‐resource environment.

**Figure EV2:**
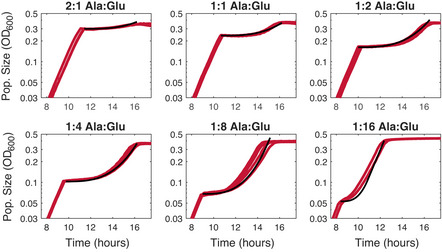
Example lag time fits The Aci2 monoculture data from Fig 2B are shown in red with the fits used to produce the lag time estimates shown in red (Materials and Methods). The remaining Aci2 fits are presented in Appendix Fig S3. The Pa fits are presented in Appendix Fig S4 with a note on interpretation of Pa’s optical density data provided in Appendix Fig S5. These fits were performed using the species’ two‐resource growth rates as their post‐recovery steady‐state growth rates to maintain consistency with modeling decisions. Fits using the species’ single‐resource growth rates are provided in Appendix Fig S7 and differ only slightly.

These monoculture experiments also yielded additional information about the diauxic lag times of each species. Notably, Aci2’s diauxic lag time varied considerably with resource supply ratio. For fitting, we assumed growth rates recovered proportional to the square of the time since the resource depletion (Materials and Methods). This recovery shape was chosen for having a better empirical fit to Aci2’s monoculture growth curves than other shapes considered (Appendix Fig S2) while maintaining a simple, single‐parameter functional form. When mostly glutamate was supplied Aci2’s lag time was the shortest (~2 h), and as the supply of alanine increased Aci2’s lag time became longer (up to ~12 h) (Figs 2D and H, and EV2). We observed no variation with initial population size but note that Aci2’s lag time could be a function of either resource supply or its population size at the onset of its diauxic shift (Appendix Fig S8; Materials and Methods). Examples of variable diauxic lag times have been studied (Solopova *et al*, 2014; Venturelli *et al*, 2015; Wang *et al*, 2015; Vermeersch *et al*, 2019). One particularly similar example concluded that larger supplies of the preferred resource and larger population sizes just before the resource depletion increased the overall rate of resource consumption and suddenness of the depletion, giving the population less time to prepare and a longer diauxic lag (Solopova *et al*, 2014). This explanation could be applicable here, but we did not investigate further, as our focus was on the ecological consequences, not the origins, of the species’ diauxic lags.

### Simple growth‐lag model reproduces monoculture dynamics

**Figure 3:**
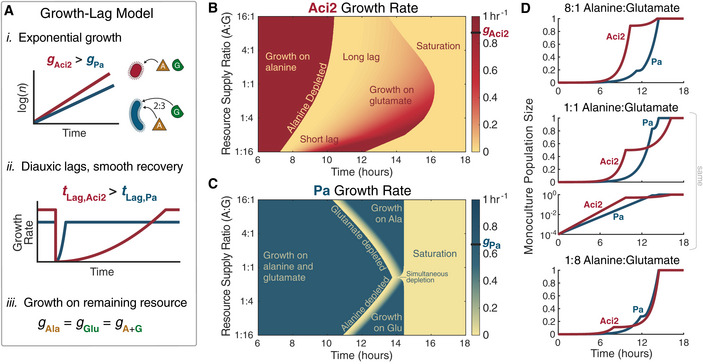
Growth‐lag model reproduces monoculture growth dynamics A simple model of growth and lag phases. (i) Species initially grow exponentially with Aci2 initially consumes only alanine and Pa consuming alanine and glutamate in a fixed 2:3 ratio. (ii) When a resource is depleted, both species experience a diauxic lag. Aci2’s lag time is long and varies with resource supply. Pa’s lag is short and constant at 1 h. Growth rates recover with a quadratic time dependence. (iii) Once recovered from its diauxic lag, each species grows on the remaining resource at its initial growth rate. (Modeling details in Materials and Methods.)Model predictions for Aci2’s monoculture growth for different resource supply ratios when started at a 10^4^ dilution from carrying capacity. Each row of pixels is the calculation for a separate condition.The equivalent predictions for Pa’s growth.Comparison of the monoculture growth calculations at 8:1, 1:1, and 1:8 resource supply ratios. The appearance of initial lag is due to the growth that needs to happen before population sizes become significant on a linear scale. Population sizes are presented in dimensionless units (Materials and Methods).

With an understanding of each species’ growth dynamics, we developed a minimal growth‐lag model in which the only tradeoff would be between growth rate and diauxic lag time. We used a single exponential growth rate for each species that applied to all environments to eliminate any tradeoff between two‐resource and single‐resource growth rates (Fig 3A). The model used the experimentally determined two‐resource growth rates: 0.88/h for Aci2 and 0.67/h for Pa (Fig 2A). Lag times were interpolated from experimentally determined values (Fig 2H), including the dependence of Aci2’s lag time on resource supply ratios, and growth rate recoveries were modeled as having the same quadratic shape as used for fitting lag times (Materials and Methods). No additional complexities were included in order to have a minimal model with which we could later test the ability of a growth‐lag tradeoff to explain coexistence and predict qualitative trends in community assembly without the need to invoke other, more complicated interactions.

The growth‐lag model reproduced the key features of Aci2’s monoculture dynamics (Fig 3B in comparison to Fig 2D). Aci2 initially consumed only alanine, so in both model and experiment, Aci2’s diauxic shift occurred later the more alanine was supplied. Saturation, meanwhile, occurred latest at roughly equal resource supply ratios. Quick saturation at low alanine supply fractions was the result of Aci2’s shorter diauxic lag. Quick saturation at high alanine supply fractions was achieved despite Aci2’s long lag because Aci2 did not need to significantly recover its growth rate to complete the small amount of remaining growth (Fig 3B and D). The reproduction of these features indicates that our simple model was accurately capturing Aci2’s growth and resource consumption behavior.

**Figure EV3:**
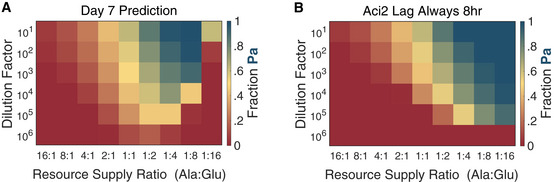
Predicted mean fraction at Day 7 and predicted steady‐state if Aci2’s lag did not vary with resource supply Predicted mean fraction Pa after seven dilution cycles of competition. Shown is the average fraction Pa from competitions started with a Pa fraction of 0.1, 0.25, 0.75, and 0.9 (same as in experiments).Predicted steady‐state fraction Pa if Aci2’s lag did not vary with resource supply but was instead constant at 8 h. The value of 8 h was chosen for being close to Aci2’s mean lag time of 9 ± 1 h and providing a close fit to the experimental results.

The growth‐lag model for Pa contained the same elements as for Aci2, but defining Pa as initially co‐utilizing alanine and glutamate and giving Pa a shorter, constant lag produced distinct dynamics. These dynamics were again a close match between model and experiment (Fig 3C in comparison to Fig 2G). The onset of Pa’s diauxic shift occurred as a result of alanine running out at small alanine supply and as a result of glutamate running out at small glutamate supply. Pa’s short and constant diauxic lag meant it saturated at the same time for most resource supply ratios. When the supply ratio matched Pa’s consumption ratio, alanine and glutamate ran out at the same time so Pa had no diauxic shift and saturated slightly earlier. These features of Pa’s growth dynamics all agreed between model and experiment. The timing of Pa’s diauxic shift was, however, approximately 2 h earlier in the modeling than in the experiments. One hour of this offset is accounted for by the small initial lag that Pa displayed in the experiments (Fig 2A). The remaining hour could be explained by a variety of hypotheticals, such as a slightly density‐dependent growth rate (Fig EV3C). Further exploration and additional modeling complexity could have produced a closer fit, but the most significant features are already captured by the presented model and any additional complexity would have diminished interpretability of later results. The growth‐lag model therefore reproduced the distinct growth and resource‐consumption dynamics of both Aci2 and Pa.

### Tradeoff between growth rate and lag time is sufficient for coexistence

**Figure 4:**
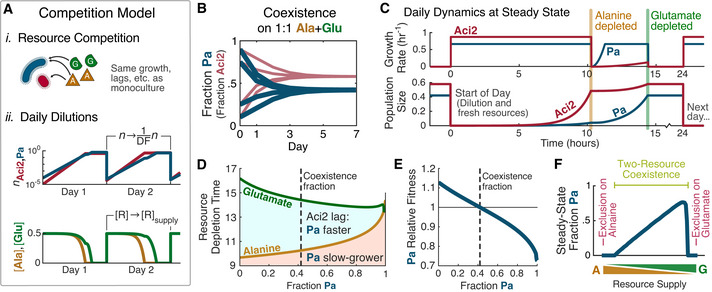
Growth‐lag model uses the tradeoff between Aci2’s fast growth and Pa’s short lag to explain two‐resource coexistence The competition model is an extension of the monoculture model. (i) Species have the same growth, lags, and resource consumption as in monocultures, but now compete for the same resource pool. (ii) After saturation, population sizes are divided by the dilution factor (10^4^ in this figure) and resource concentrations are reset to the supply concentrations. (Modeling details in Materials and Methods.)With parameters matching those used experimentally in Fig 1C, the growth‐lag model predicts coexistence of Aci2 and Pa on alanine and glutamate, as observed in competition experiments.Model prediction of growth rates and population sizes over the course of 1 day after the 1:1 alanine:glutamate co‐culture has reached steady state. Pa starts the day with a population fraction of 0.42, declines to a population fraction of 0.07 at alanine depletion, and recovers to a population fraction of 0.42 by the time glutamate is depleted.The alanine and glutamate resource depletion times vary with the (start‐of‐day) population fractions due to different resource preferences and initial growth rates.These variations modulate the relative fitness of the two strains and in doing so facilitate a negative‐frequency dependent interaction that stabilizes coexistence. Relative Fitness of Pa is calculated as its fold growth over the course of a day divided by Aci2’s fold growth (Materials and Methods), and shown as a function of its population fraction at the start of the day.The model predicts the competitive outcome will depend on the resource supply fractions with exclusion occurring as the supply approaches entirely alanine or entirely glutamate.

With a growth‐lag model developed and shown to accurately reproduce the monoculture dynamics, we were ready to test whether the tradeoff between growth rate and lag time was sufficient for the experimentally observed coexistence. In competition, the two species maintained the same resource consumption dynamics as in monoculture but now competed for the same resource pool. Each time the species depleted both resources, their populations were divided by the dilution factor and resource concentrations were returned to the supply concentrations (Fig 4A; Materials and Methods). We used this model to predict the species’ population fractions over seven dilution cycles (as was done experimentally in Fig 1C).

Our model successfully reproduced stable coexistence of Aci2 and Pa on alanine and glutamate with the same steady state being reached regardless of initial starting fractions (Fig 4B). At steady state, population dynamics were driven by diauxic resource consumption, with two distinct periods of growth causing population fractions to vary considerably over the course of a day. Aci2 initially grew faster, causing Pa’s population fraction to decrease significantly over the first ~10 h until alanine was depleted. After alanine depletion, Pa’s growth rate quickly recovered while Aci2 suffered a long lag, allowing Pa to catch back (Fig 4C). The balance of these two growth phases allowed the model to reproduce coexistence of the two species.

Coexistence was stable due to negative frequency‐dependent selection mediated by changes to the resource depletion times. If Pa’s population fraction were increased above its steady‐state (and Aci2’s correspondingly decreased), alanine would be depleted later and glutamate sooner (Fig 4D) because the population would overall be consuming glutamate faster and alanine slower. A later alanine depletion and earlier glutamate depletion would both lengthen the period of Aci2’s initial fast growth and shorten the period after alanine depletion during which Pa could catch up. An increase in Pa’s population fraction would therefore affect the resource depletion times in ways that decrease Pa’s fitness relative to Aci2 (Fig 4E). Similarly, a decrease in Pa’s population fraction would increase its relative fitness through an opposite set of effects. Thus, feedback between population fractions and resource depletion times created negative frequency‐dependent selection and stable coexistence.

### Accurate prediction of response to environmental changes validates growth‐lag model

**Figure 5:**
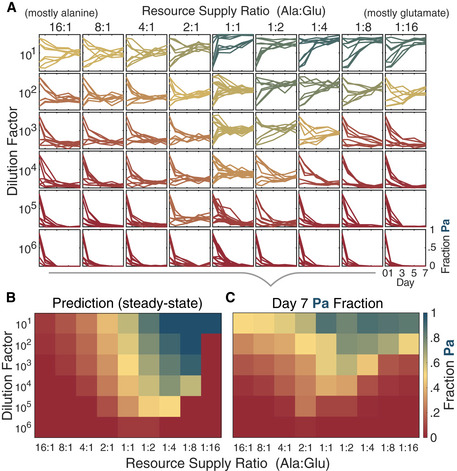
Diauxic‐lag model successfully predicts how community composition responds to changes in the dilution factor and resource supply In order to validate the modeling and test its predictive power, we repeated the competition experiments between Aci2 and Pa across a 54‐condition grid of resource supply ratios and dilution factors (Materials and Methods). Shown is the fraction Pa on Days 1, 3, 5, and 7 for eight total replicates of each condition (two biological replicates, each with four technical replicates started at different initial species fractions; Materials and Methods).We used the model to predict steady‐state population fractions for each condition.We calculated mean final population fractions from the experimental data and compared these fractions to the model predictions. Source data are available online for this figure.

Our model predicted community composition would vary considerably with changes to resource supply ratios (Fig 4F), so we decided to test whether the tradeoff between growth rate and lag time could be used to accurately predict the response to environmental changes. We chose a 54‐condition parameter grid of nine resource supply ratios and six dilution factors, calculated the model’s steady‐state predictions (Fig 5B), and performed the coculture competitions (Fig 5A and C and Appendix Fig S9).

The model and experiment agreed on several notable features (Fig 5C in comparison to Fig 5B). First, both featured a broad region of coexistence that covered more than half the conditions tested (32/54 in model and 42/54 in experiment, with 30/42 experimental observations of coexistence correctly predicted). Second, Aci2 competitively excluded Pa at large dilution factors regardless of resource supply ratio due to species spending more time in the initial growth phase, during which Aci2 had an advantage. Aci2 also excluded or nearly excluded Pa at intermediate dilution factors and large supply fractions of either alanine or glutamate. This trend centered the region of coexistence on roughly equal resource supply ratios (Fig 5B and C).

Third, the model accurately predicted that Pa would achieve its largest population fractions at low dilution factors and large glutamate supply fractions. This exclusion was the result of relatively little growth occurring before alanine ran out and more growth occurring during Aci2’s diauxic lag when Pa had a strong but temporary advantage. The model predicted four conditions at which Pa would even competitively exclude Aci2 (Fig 5B). This competitive exclusion was not observed experimentally, but there were four conditions at which Pa obtained population fractions greater than 0.83 (Fig 5C). That the slow‐grower could become the majority of the population at specific conditions was a surprising and nontrivial result of both the model and experiment.

**Figure EV4:**
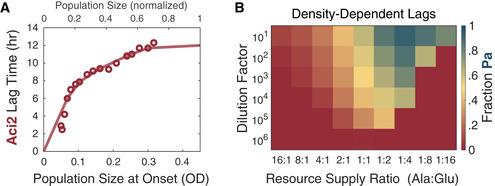
Modeling Aci2’s lags as dependent on its population density at the onset of its diauxic shift (instead of initial alanine supply) prevents Pa from excluding Aci2 Using the lag times presented in Fig 2H and the population sizes at the onset of Aci2’s diauxic shift presented in Fig 2C allows Aci2’s lag time to be alternatively expressed as a function of its population size at the onset of its diauxic shift. Circles represent lag time data and the line defines the piecewise linear function used for modeling. Whether Aci2’s lag is most accurately modeled as a function of alanine supply or of population size at the time of its diauxic shift could not be definitively determined (Appendix Fig S8).Steady‐state phase space for the Aci2 versus Pa on alanine and glutamate competition at various resource supply ratios and dilution factors when modeling Aci2’s lag as a function of its population size at the onset of the diauxic shift. The most significant change relative to the original prediction (Fig 5B) is that Pa never fully excludes Aci2. Pa cannot exclude Aci2 because as Aci2 is driven extinct its population size at its diauxic shift as well as its diauxic lag time converge to zero, eliminating the period when Pa can catch up to Aci2.

The model did not, however, capture all the experimental observations. In particular, at high alanine supply fractions and low to intermediate dilution factors, the model predicted exclusion or near‐exclusion of Pa whereas the experimental observation was coexistence. This discrepancy was also present to a lesser degree at high glutamate supply fractions and notably at low dilution factors in single‐resource competitions (Appendix Fig S9). There are several possible explanations. For example, modeling Aci2’s lag time as dependent on its population size at the time of the diauxic shift rather than the initial resource supply ratio would predict coexistence at some conditions in which exclusion is falsely predicted under the presented model (Fig EV4). Dynamics occurring during the stationary phase, a slight density‐dependence of Pa’s growth rate, and crossfeeding are all alternative possible explanations. But, without reason to pursue any explanation in particular and a possibility of a number of factors each playing a small role, we were satisfied with how many of the key features had been accurately predicted.

Overall, across all conditions tested the root‐mean‐square error between the model prediction and the experimental observation was a species fraction of 0.24 (Appendix Fig S10). This error could have been reduced by adding additional model complexities or fitting parameters to competitive outcomes unconstrained by the monoculture characterizations. For example, one complexity would be to use the species’ single‐resource growth rates for post‐shift growth instead of having a single growth rate for each species, but this addition would change the modeling prediction surprisingly little (Appendix Fig S11). The goal of our modeling was not, however, to obtain the closest possible empirical fit, but to instead show that a simple model with a minimal number of elements could predict qualitative features over a wide range of environmental conditions. Reducing the model to an even simpler form, it was observed that many of the key features were preserved even when Aci2 was assigned a constant lag time (Fig EV3B). The predictions also varied surprisingly little if Pa were modeled as a specialist instead of as a co‐utilizer (Appendix Fig S12), confirming that it was indeed the lag time difference and not the resource preference difference that yielded coexistence. We took these results as confirmation that the competitive dynamics between Aci2 and Pa are primarily driven by the tradeoff between Aci2’s fast growth and Pa’s short diauxic lags.

### Coexistence is more likely when slow‐growers are fast‐switchers

**Figure 6:**
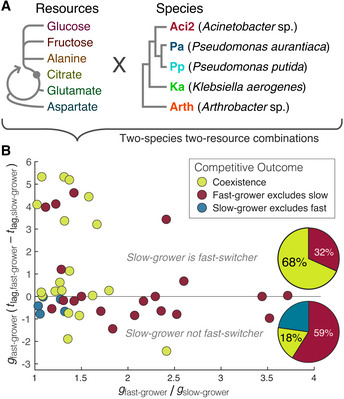
Slow‐growers are more likely to coexist with fast‐growers when the slow‐growers are fast‐switchers We selected six resources and five species for additional experiments. Resources are shown in relation to glycolysis and the citric acid cycle. Species are shown with a phylogenic tree with connections in the correct order but not to scale.Monoculture experiments were performed to determine species’ growth rates and lag times for each two‐resource environment. For each pair of species, coculture competition experiments were performed in each two‐resource environment, and qualitative competitive outcomes were determined (Materials and Methods). There were 44 competitions in which both species could be assigned growth rates and lag times (Fig 6 Source Data). Each point represents one competition. Pie charts summarize the competitive outcomes for cases in which the slow‐grower was the fast‐switcher (above the Δ*t*_*lag*_ = 0 line) and in which the slow‐grower was not the slow‐switcher (on and below Δ*t*_*lag*_ = 0). Data Information: All monoculture data with growth rate and lag time fits, all competition data, and notes on specific competitions are available in the source data. Source data are available online for this figure.

Having established the fitness tradeoff between Aci2’s fast growth and Pa’s fast diauxic shift as the mechanism by which they coexisted, we proceeded to explore whether this fitness tradeoff could be a common mechanism for coexistence between species. We added *Pseudomonas putida* (Pp), *Klebsiella aerogenes* (Ka), and an *Arthrobacter* species (Arth) to our set of species and fructose, glucose, citrate, and aspartate to our set of resources (Fig 6A). We performed coculture competitions for each species pair in each two‐resource environments (Fig 6 Source Data). We then determined initial growth rates and diauxic lag times for each species in each two‐resource environment (Fig 6 Source Data) and calculated differences in growth rates and lag times for each species pair in each two‐resource environment. We excluded some cases in which we could not fit growth rates and lag times, such as when a species did not grow on both resources. There were 22 competitions in which the slow‐grower was the fast‐switcher and 22 competitions in which it was not. When we compared these categorizations to the competitive outcomes, we saw that coexistence occurred 3.8 times more frequently when the slow‐grower had been the fast‐switcher (*P* = 0.002, Fisher’s exact test), occurring in 68 ± 8% of cases compared to just 18 ± 8% of cases (Fig 6B). These results do not prove that the tradeoff between growth rate and diauxic lag time caused coexistence in each of these cases, but they do argue that such tradeoffs may be a significant factor supporting the coexistence of species in multi‐resource environments.

Surprisingly, there were five cases in which the slow‐grower was the slow‐switcher but actually excluded the fast‐grower. In four of these cases, the excluded fast‐grower was Arth, which tended to have long initial lags and slow post‐shift growth rates. These unexpected exclusions highlight how other elements of species’ monoculture growth dynamics can also be important determinants of competitive fitness. The relative importance of initial and diauxic lags and single‐ and two‐resource growth rates on determining competitive outcomes is explored in the Appendix, but the higher‐dimensional complexity of these tradeoffs meant a thorough, integrated study needed to be left for future work.

The data also contained examples of species being the slow‐switcher in some scenarios but the fast‐switcher in others. The competitions between Pp and Arth are one set of examples. There were two environments (fructose and citrate and fructose and alanine) in which Pp was the fast‐grower but slow‐switcher and one environment (fructose and aspartate) in which Arth was the fast‐grower but slow‐switcher. In all three of these environments, the two species coexisted. There were also three environments (glucose and citrate, alanine and glutamate, and alanine and aspartate) in which Pp was both the fast‐grower and the fast‐switcher, and in all three of these environments, Pp excluded Arth (see Fig 6 Source Data for details). These examples highlight the importance of the specific environment to the growth dynamics of species and assembly of ecological communities, as well as the power of a single growth‐lag characterization to predict community assembly across a wide range of environments.

## Discussion

In this paper, we began by investigating a case of unexpected coexistence in a two‐resource environment with Pa surviving on the combination of alanine and glutamate despite being competitively excluded on either resource alone and despite being the consistent slow‐grower. We discovered that, while Pa was the slow‐grower compared to Aci2, Pa was the fast‐switcher. We developed a model based on the tradeoff between Aci2’s fast growth and Pa’s short diauxic lag and showed that this tradeoff was sufficient to explain the coexistence result. The model even predicted how the community composition would respond to environmental changes. We finished by showing that across a set of 44 two‐species two‐resource competitions, the slow‐grower being the fast‐switcher made coexistence nearly four times as likely to occur. Our results establish the power of diauxic lags to effect coexistence and suggest such a mechanism may be common.

Previous studies have identified metabolic tradeoffs whereby fast‐growers optimize their growth strategies for their current environment at the expense of being able to quickly adapt after resource depletions (New *et al*, 2014; Solopova *et al*, 2014; Chu & Barnes, 2016; Balakrishnan *et al*, 2021). When such a tradeoff exists, slow‐growers will tend to be fast‐switchers while fast‐growers will be slow‐switchers. In our focal example of Aci2 and Pa, a tradeoff between Aci2’s fast growth and Pa’s short diauxic lags produced stable coexistence. We also showed that slow‐growers are more likely to coexist with fast‐growers when the slow‐growers are fast‐switchers. We did not investigate whether such a tradeoff was indeed responsible for coexistence in each of the cases we identified. A similar tradeoff has, however, been seen between different strains of *E. coli* growing on glucose and acetate (Friesen *et al*, 2004; Spencer *et al*, 2007), establishing that Aci2 and Pa on alanine and glutamate is not the only case in which coexistence is the demonstrable result of a tradeoff between growth and lag. The combination of previous results and those presented here suggests tradeoffs between growth rate and diauxic lag time may be a common mechanism for the coexistence of species, although future work is needed to establish a mechanistic connection in more cases.

The relevancy of tradeoffs between growth rate and lag time will also depend on the extension to more natural communities and environments. We considered the case of two species growing on two resources whereas natural ecosystems contain many species and resources. With more than two resources supplied, species may undergo multiple diauxic shifts, which could help larger numbers of species coexist. But, in the many‐resource limit, a diauxic lag with every resource depletion would leave species endlessly trapped in lag phases, suggesting that this framework may lose realism in some cases. A recent theoretical study has, however, shown that even short diauxic lags can accelerate an ecosystem’s convergence to a coexistence steady‐state (Wang *et al*, 2021), consistent with our modeling results that diauxic lags make random species more likely to coexist (Appendix). Understanding when discrete growth and lag phases are an accurate picture of microbial growth will be a necessary step in applying tradeoffs between growth rate and lag time to more complex environments.

Cross‐feeding is thought to be one of the most important factors in the assembly of natural communities but is not an interaction we sought to study in this paper. There are, however, interesting connections between diauxic lags and cross‐feeding. Diauxic lags can occur when a microbe switches from a supplied resource to one of its own byproducts (Lemoigne *et al*, 1954; De Deken, 1966; Wolfe, 2005). In the case of a primary degrader eating a supplied resource and producing a byproduct and a cross‐feeder eating that byproduct, if the primary degrader consumes the byproduct after the supplied resource runs out and grows faster on both resources than the cross‐feeder on the byproduct, then in the absence of lags the primary degrader will always be growing faster and always increasing its population fraction relative to the cross‐feeder. But if the primary degrader has a diauxic lag, the cross‐feeder will have a chance to catch up and maintain its population fraction. These intersections between diauxic lags and cross‐feeding may turn out to be some of the cases in which tradeoffs between growth rates and diauxic lag times are the most ecologically relevant. But, that diauxic lags alone can produce stable coexistence shouldn’t be overlooked as our results highlight how simple models that don’t attempt to capture every detail can still explain and predict surprising results in community assembly.

In addition to diauxic lags, microbes often display initial lags, which occur when previously stationary species are presented with fresh resources. Diauxic and initial lags are similar phenomena: a microbe requiring time to reach its maximum growth rate when presented with a new environment. Diauxic lags do, however, allow for coexistence in ways that initial lags do not, primarily because diauxic lags divide the growth phase into two subphases with distinct frequency‐dependent dynamics while initial lags only affect a single growth phase. Recent theoretical work has, however, shown that tradeoffs between growth rate and initial lag can be a source of coexistence with only a single growth phase (Manhart & Shakhnovich, 2018; Manhart *et al*, 2018), but for this coexistence to be possible without parameter fine‐tuning a density‐dependent dilution factor is necessary. In the Appendix, we explore the effects of adding initial or diauxic lags in a variety of scenarios including already‐biphasic growth and conclude that diauxic lags have a greater tendency to produce coexistence than initial lags do. Regardless of their relative potency, both initial and diauxic lags are ubiquitous phenomena with considerable ecological relevancy that warrant ongoing theoretical and ecological study, ideally from a unifying approach.

Our work adds to a growing body of research exploring the cases in which the fastest‐growing species is not necessarily the strongest competitor in an ecosystem. Previous work has shown that decreasing the dilution factor or mortality rate favors slower‐growing species. This universal property was originally derived from a Lotka‐Volterra model and demonstrated experimentally (Abreu *et al*, 2019). Our focal example of Aci2 and Pa on alanine and glutamate maintained this property, and additional modeling in the Appendix explores various scenarios in which diauxic lags allow a slow‐growing species to coexist with, or even exclude, a faster‐growing competitor as well as a scenario in three species stably coexist on two resources. In spatially structured ecosystems, it is known that a slow‐grower can outcompete a fast‐grower by being the fast‐disperser and colonizing new locations before the fast‐grower can arrive (Nadell & Bassler, 2011; Yawata *et al*, 2014; Lee *et al*, 2022). The tradeoff between growth rate and dispersal is a parallel to the tradeoff between growth rate and lag time: Aci2 could be loosely thought of as outcompeting Pa on the original two‐resource island while Pa survives by dispersing to the post‐shift single‐resource island faster. Future research can continue to elucidate the various roles of diauxie and diauxic lags in supporting the coexistence of diverse communities in multi‐resource environments.

## Materials and Methods

### Species and media

Aci2 and Arth were previously isolated from soil and identified as an *Acinetobacter* species and an *Arthrobacter* species (Lax 2020). Pa, Pp, and Ka were obtained from ATCC. Pa is *Pseudomonas aurantiaca* (ATCC 33663), Pp *Pseudomonas putida* (ATCC 12633), and Ka *Klebsiella aerogenes* (ATCC 13048). Species were streaked from glycerol freezer stocks onto agar plates and single colonies were picked for each experiment. Agar plates contained 15 g/l agar, 5 g/l peptone, and 3 g/l yeast extract. This recipe was also used for the colony counting plates in competition experiments. Aci2 and Arth are available upon request.

All experiments were performed in an M9 media. The media contained final concentrations of 11.28 g/l Sigma‐Aldrich M9 Minimal Salts (3 g/l KH_2_PO_4_, 0.5 g/l NaCl, 6.78 g/l Na_2_HPO_4_, 1 g/l NH_4_Cl), 1.9 mM MgSO_4_, 0.95 mM CaCl_2_, 3.8 mg/l FeSO_4_‐7H_2_O, 3.8 mg/l MnCl_2_‐4H_2_O, 2.1 mg/l CuSO_4_‐5H_2_O, 1.2 mg/l ZnSO_4_‐7H_2_O, 240 μg/l NaMoO_4_‐2H_2_O, 240 μg/l CoCl_2_‐6H_2_O, 9.5 mg/l Na_2_EDTA, and a total of 0.1% w/v supplied carbon source. Fructose was supplied as D‐(–)‐fructose, glucose as D‐(+)‐glucose, citrate as citric acid, alanine as l‐alanine, glutamate as l‐glutamic acid monosodium salt, and aspartate as l‐aspartic acid monopotassium salt. Weight per volume calculations excluded the weight of any metal ions. The pH was not adjusted after preparing the solutions, but all competition media consistently had pH 6.9 ± 0.1 with the exception of citrate media, which had pH 6.5 ± 0.3.

### Coculture competitions

Two days before the start of the competitions experiments individual colonies were picked off agar plates and grown in monoculture for 24 h in LB media. The day before the start of the experiments, the monocultures were diluted 1:1,000 and transferred into M9 media containing equal parts alanine and glutamate when experiments related to the case of Aci2 and Pa on alanine and glutamate were being run and into M9 media containing equal parts fructose, glucose, citrate, alanine, glutamate, and aspartate when the survey of additional species and resources was being run. These monocultures were grown for an additional 24 h before being used to inoculate the experiments. To initialize the coculture experiments, monocultures were mixed at 90:10, 75:25, 25:75, and 10:90 ratios, and the resulting mixtures were diluted by factors matching the experimental daily dilution factors and used to inoculate the competition media. 75:25 and 25:75 inoculum ratios were only used for experiments involving Aci2 and Pa on alanine and/or glutamate.

Competitions were incubated at 25°C on 96‐well plates with 200 µl in each well and orbital shaking at 350 rpm. Every 24 h, the cocultures were diluted by a factor corresponding to the experimental condition. At the first, third, fifth, and seventh dilutions, the media was diluted by a factor of 10^6^ and 10uL droplets were plated onto agar plates. Between five and ten replicates of the plating were performed. This yielded 100–200 total colonies from most wells. These colonies were counted to determine species’ population fractions. All colonies were easily distinguishable (Appendix Fig S13). For the survey of additional species and resources, competitions were only run for 5 days, so no Day 7 plating was performed.

In the survey of additional species and resources, uncertainties on the fractions of competitions that produced coexistence came from a beta distribution with

$$\sigma =\sqrt{\frac{\left(N_{\mathrm{Coex}}+1\right)\left(N_{\mathrm{Excl}}+1\right)}{{\left(N_{\mathrm{Coex}}+N_{\mathrm{Excl}}+2\right)}^{2}\left(N_{\mathrm{Coex}}+N_{\mathrm{Excl}}+3\right)}}$$

while categorization into exclusion and coexistence outcomes was done using a Day 5 species fraction of 0.02 as an approximate cutoff, although whether species fractions were stable or decreasing from Day 3 to Day 5 also influenced these categorizations. Notes on all borderline categorizations are included in the Fig 6 Source Data.

### Monoculture experiments

The monoculture experiments began with the same two‐step starter‐culture procedure as in the coculture experiments. Monoculture experiments involving Aci2 and Pa on alanine and/or glutamate were inoculated with a 10^−4^ dilution. The other experiments were inoculated at 2*10^−3^ or 5*10^−4^. (These dilution factors were used instead of 10^−4^ due to the slower growth rates of some species.) Population size was measured as optical density at either 400 or 600 nm every 5 min, using a Tecan Infinite M200 Pro multiplate reader. Which wavelength was used for each experiment is provided in the corresponding figure caption or axis label. The noise level on our machine was approximately ± 0.001 OD, and all data presented in the Main Text remained within the linear range. Plates were kept at 25°C with orbital shaking in between OD measurements. For each well, the minimum OD value after median filtering with a bin width of seven measurements was used as a background value and subtracted from the measured values.

For the experiments in which the alanine:glutamate ratio was varied between 25 different ratios (Fig 2B–G), those ratios were defined by 1:2*^n^* for *n* ranging with −4 to 4 with a step size of 1/3. For the survey of additional species and resources (Fig 6), the resource supply ratios were 4:1, 1:1, and 1:4. The resource supply combinations used in the data shown in Appendix Fig S8 are defined in Appendix Fig S8A.

### Growth rate measurements

Two methods of growth rate measurement were used in this paper: one for determining steady‐state growth rates when a single value was desired (e.g., reporting values in the text and determining values to be used in modeling) and one for calculating instantaneous growth rates over time (e.g., Fig 2D and G).

To calculate steady‐state growth rate values, optical density data were collected and background‐corrected as described above. For Aci2 and Pa on alanine and/or glutamate optical density data to be used for this purpose was collected at 400 nm. We defined 4.5‐h windows when optical density was above the noise level of our machine but no diauxic shifts had begun and when growth appeared as close to linear on a log‐linear plot as available in the data. We then used a Thiel‐Sen estimator in which a slope was fit through every pair of points, and the median slope was used as the reported growth rate. The reported uncertainty is the standard deviation of the set of slopes. The Pa monoculture data contained some minor optical density artifacts (Appendix Fig S5), but the obtained rate value of 0.67/h was a good overall fit to the data (Fig EV1C).

To extract instantaneous growth rates over time, a linear least square fit was performed on population sizes from a 30‐min rolling window (7 measurements) and the slope of this fit was divided by the population size at the center of the window. When plotting, replicates were median filtered to have a single growth rate time series for each condition.

For the survey of additional species and resources, growth rates were determined by manually fitting a line through the data on a log‐linear plot. These fits are provided in the Fig 6 Source Data. In some cases, a reliable growth rate could not be obtained (usually due to there not being a sufficient period of steady‐state exponential growth to work with), and the data and corresponding competitions were excluded from the analysis.

### Lag time measurements

Lag times were fit using a growth rate recovery shape in which growth rate recovered proportional to the square of the time since the resource depletion,

$$g\left(t\right)=g_{\mathrm{SS}}{\left(\frac{t‐t_{0}}{t_{\mathrm{lag}}}\right)}^{2}\;if\;t<t_{\mathrm{lag}},\;else\;g\left(t\right)=g_{\mathrm{SS}},$$

where *g_SS_* is the steady‐state growth rate, *t*_0_ is the resource depletion time, and *t*_*lag*_ is the species lag time. This recovery shape was used because it was a single‐parameter fit that was a close empirical match to post‐shift recoveries observed in the monoculture data, particularly in comparison to other recovery shapes considered (Appendix Figs S2 and S6). In the Main Text, lag times are reported as a function of resource supply ratio. Lag times were also fit for different initial dilution factors but no variation was observed (Appendix Fig S8).

Integrating the quadratic growth rate recovery yielded a simple cubic form for log‐scaled population size as a function of time:

$$\log\;n\left(t\right)=g_{\mathrm{SS}}\frac{{\left(t‐t_{0}\right)}^{3}}{3\;t_{\mathrm{lag}}^{2}}\;\;if\;t<t_{\mathrm{lag}},\;\;else\;\log\;n\left(t\right)=g_{\mathrm{SS}}\left(t‐\frac{2}{3}t_{\mathrm{lag}}\right).$$

For fitting Aci2 and Pa lag times on alanine and glutamate, *g_SS_* was fixed as the species’ two‐resource growth rates (0.88/h and 0.67/h; Fig 2A). (Fitting lag times using the species’ single‐resource growth rates had only a minor impact on the results (Appendix Fig S7).) Because *g_SS_* was fixed, the lag time fit was a single‐parameter fit. Lag time fits were performed manually. First, the onset of the diauxic shift was defined on a log‐linear plot of optically density over time. Second, the value of *t*_*lag*_ was adjusted until the best empirical was obtained. Upper and lower bound estimates were obtained by determining the maximum and minimum values of *t*_*lag*_ that produced remotely reasonable fits. All fits, including upper and lower bound estimates, are presented in Appendix Figs S3 and S4.

Pa has small spikes in optical density at the onset of its diauxic shift and at saturation (Appendix Fig S5). These spikes were ignored when fitting the Pa lag times such that lag times correspond to the time it takes Pa to reach its steady‐state growth rate not the maximum observed growth rate as this is believed to be an artifact. We did not engage in a mechanistic study of these spikes in optical density, but *Pseudomonas* have been previously observed to rapidly increase per capita optical density under environmental changes due to morphological changes (Bernheim, 1963).

For the survey of additional species and resources, lag times were fit to data from whichever of the three resource ratios (4:1, 1:1, 1:4) produced the earliest‐occurring diauxic shift (to maximize post‐shift growth available for fitting). For these fits, *g_SS_* was not fixed and was manually adjusted in parallel to *t*_*lag*_ being adjusted. These fits are all shown in the Fig 6 Source Data. The Aci2 and Pa lag times were refit for this comparison with *g_SS_* unfixed so as to be subject to the same biases (if any) as the other species’ fits. In some cases, a reliable lag time fit could not be obtained. In these cases, the data and corresponding competitions were excluded from the analysis. Notes on specific fits are provided in the Fig 6 Source Data.

### Modeling

Modeling was done using dimensionless resource concentrations and population sizes but with an explicit time dimension (hours). Monoculture and coculture simulations followed the same set of equations. The total resource supply was set to 1 (i.e., *s*_Ala_ + *s*_Glu_ = 1 where *s*_Ala_ is the alanine supply concentration and *s*_Glu_ is the glutamate supply concentration), and all yields were implicitly set to 1.

For competition simulations, the system was initiated with the resource concentrations, *c*_Ala_(*t*) and *c*_Glu_(*t*), equal to the supply concentrations and the total population sizes normalized to the carrying capacity divided by the dilution factor DF (which range from 10 to 10^6^ in our modeling and experiments),

$$n_{Aci2}\left(0\right)+n_{\mathrm{Pa}}\left(0\right)=\frac{1}{DF‐1}$$

where $\frac{1}{DF‐1}$ appears instead of $\frac{1}{\mathrm{DF}}$ due to the carry capacity being $\frac{\mathrm{DF}}{DF‐1}$ after correcting for the day‐to‐day population carryover (Appendix).

For monoculture simulations, the system was initiated with the resource concentrations equal to the supply concentrations and *n*(0) = 10^−4^ for the species being simulated and *n*(0) = 0 for the other species.

Before a resource depletion (i.e., while *c*_*Ala*_(*t*) > 0 and *c*_*Glu*_(*t*) > 0) the system evolved according to.

$$n_{Aci2}^{\prime }\left(t\right)=\left(0.88\;h^{‐1}\right)n_{Aci2}\left(t\right)$$

$$n_{\mathrm{Pa}}^{\prime }\left(t\right)=\left(0.67\;h^{‐1}\right)n_{\mathrm{Pa}}\left(t\right)$$

$$c_{\mathrm{Ala}}^{\prime }\left(t\right)=‐n_{Aci2}^{\prime }\left(t\right)‐\frac{2}{5}n_{\mathrm{Pa}}^{\prime }\left(t\right)$$

$$c_{\mathrm{Glu}}^{\prime }\left(t\right)=‐\frac{3}{5}n_{\mathrm{Pa}}^{\prime }\left(t\right)$$

until either *c*_*Ala*_(*t*) = 0 or *c*_*Glu*_(*t*) = 0.

After the first‐resource depletion, the system evolved according to the same growth rate recovery shape as used in fitting the lag times and with yields still implicitly set to 1,

$$n_{\mu }^{\prime }\left(t\right)=g_{\mu }\left(t\right)n_{\mu }\left(t\right)\;with\;g_{\mu }\left(t\right)=g_{SS,\mu }{\left(\frac{t‐t_{0}}{t_{lag,\mu }}\right)}^{2}\;if\;t<t_{lag,\mu },\;else\;g_{\mu }\left(t\right)=g_{SS,\mu }$$

$$c_{i}^{\prime }\left(t\right)=‐n_{Aci2}^{\prime }\left(t\right)‐n_{\mathrm{Pa}}^{\prime }\left(t\right)$$

where *μ* indicates each species and *i* is the remaining resource. Post‐switch steady‐state growth rates were the same as the pre‐shift growth rates (*g*_SS,*Aci2*_ = 0.88/h and *g*_SS,*Pa*_ = 0.67/h) to avoid a tradeoff between two‐resource and single‐resource growth rates complicating the interpretation of the results and because the effect of using the measured single‐resource growth rates for post‐shift *g*_SS_ was minor (Appendix Fig S11). Pa’s lag time was 1 h in all cases. Aci2’s lag time was zero if glutamate was depleted first and function of the resource supply ratio if alanine was depleted first. This function of resource supply ratio was a linear interpolation through the following points, which were taken from the monoculture lag time fits (Fig 2H, Appendix Fig S3):

**Table:** 

Resource Supply Ratio (A:G)	1:16	1:8	1:4	1:2	1:1	2:1	4:1	8:1	16:1
Aci2 Lag Time (hours)	12.0	12.0	12.0	11.7	10.8	9.38	7.88	6.00	2.48

From monoculture experiments, it could not be determined whether Aci2’s lag time was a function of the alanine supply concentration (equivalent to being a function of resource supply ratio with the total concentration constant) or of its population size at the onset of its diauxic shift (Appendix Fig S8). Modeling predictions if Aci2’s lag time were a function of its population size were also determined and seen to be similar (Fig EV4). Other researchers have suggested variable lag times may be the result of larger populations consuming resources more quickly and having less time to prepare for the switch to the next resource (Solopova *et al*, 2014). If this is the case here, lag times would be approximate functions of the resource supply ratio because both Aci2 and Pa contribute to the coculture‐wide resource consumption rate and the total population size at alanine depletion is closely linked to the alanine supply concentration.

After the second resource depletion, the population sizes were divided by the dilution factor DF and resource concentrations were reset to the supply concentrations. No dynamics during the stationary phase (between the second resource depletion and the start of the next day) were modeled. All reported species fractions, except those explicitly labeled as being functions of time within a day, are end‐of‐day population fractions.

Simulations were performed by directly solving a sum‐of‐exponentials equation for the time at which resources would be depleted and then calculating species population sizes using that value, so no numerical integration of differential equations was required.

For the frequency‐dependent relative fitness of Pa against Aci2 on alanine and glutamate (Fig 4E), populations where initiated with $n_{\mathrm{Pa}}\left(0\right)=\frac{f_{\mathrm{Pa}}}{DF‐1}$ and $n_{Aci2}\left(0\right)=\frac{1‐f_{\mathrm{Pa}}}{DF‐1}$ and grown for one dilution cycle. The relative fitness of Pa was calculated as $\frac{n_{\mathrm{Pa}}\left(t_{\mathrm{sat}}\right)/n_{\mathrm{Pa}}\left(0\right)}{n_{Aci2}\left(t_{\mathrm{sat}}\right)/n_{Aci2}\left(0\right)}$.

## Author contributions

**Blox Bloxham:** Conceptualization; Formal analysis; Investigation; Visualization; Methodology; Writing—original draft; Writing—review & editing; BB conceptualized and designed the experiments, performed the experiments, performed mathematical modeling, analyzed the data, and wrote the manuscript. **Hyunseok Lee:** Conceptualization; Methodology; Writing—original draft; Writing—review & editing; HL conceptualized and designed the experiments, analyzed the data, and wrote the manuscript. **Jeff Gore:** Conceptualization; Supervision; Funding acquisition; Methodology; Writing—original draft; Writing—review & editing; JG acquired funding, conceptualized and designed the experiments, analyzed the data, and wrote the manuscript.

In addition to the CRediT author contributions listed above, the contributions in detail are:

BB, HL, and JG conceptualized and designed the experiments. BB performed the experiments. BB performed mathematical modeling. BB, HL, and JG analyzed the data. BB, HL, and JG wrote the manuscript. JG acquired funding.

## Disclosure and competing interests statement

The authors declare that they have no conflict of interest.

## Supporting information



Appendix

Expanded View Figures PDF

Source Data for Figure 2

Source Data for Figure 5

Source Data for Figure 6

## Data Availability

This study includes no data deposited in external repositories. All essential data are available as Source Data (see Source Data for Figs 2, 5 and 6).

## References

[msb202110630-bib-0001] Abreu CI , Friedman J , Andersen Woltz VL , Gore J (2019) Mortality causes universal changes in microbial community composition. Nat Commun 10: 1–9 31073166 10.1038/s41467-019-09925-0 PMC6509412

[msb202110630-bib-0002] Acar M , Mettetal JT , Van Oudenaarden A (2008) Stochastic switching as a survival strategy in fluctuating environments. Nat Genet 40: 471–475 18362885 10.1038/ng.110

[msb202110630-bib-0003] Adkar BV , Manhart M , Bhattacharyya S , Tian J , Musharbash M , Shakhnovich EI (2017) Optimization of lag phase shapes the evolution of a bacterial enzyme. Nat Ecol Evol 16: 1–6 10.1038/s41559-017-0149 PMC5640271 28812634

[msb202110630-bib-0004] Balakrishnan R , de Silva RT , Hwa T , Cremer J (2021) Suboptimal resource allocation in changing environments constrains response and growth in bacteria. Mol Syst Biol 17: e10597 34928547 10.15252/msb.202110597 PMC8687047

[msb202110630-bib-0005] Basan M , Honda T , Christodoulou D , Hörl M , Chang Y‐F , Leoncini E , Mukherjee A , Okano H , Taylor BR , Silverman JM *et al* (2020) A universal trade‐off between growth and lag in fluctuating environments. Nature 584: 470–474 32669712 10.1038/s41586-020-2505-4 PMC7442741

[msb202110630-bib-0006] Bernheim F (1963) Factors which affect the size of the organisms and the optical density of suspensions of *Pseudomonas aeruginosa* and *Escherichia coli* . J Gen Microbiol 30: 53–58 13971086 10.1099/00221287-30-1-53

[msb202110630-bib-0007] Chu D , Barnes DJ (2016) The lag‐phase during diauxic growth is a trade‐off between fast adaptation and high growth rate. Sci Rep 6: 1–15 27125900 10.1038/srep25191 PMC4850433

[msb202110630-bib-0008] Dal Bello M , Lee H , Goyal A , Gore J (2021) Resource–diversity relationships in bacterial communities reflect the network structure of microbial metabolism. Nat Ecol Evol 510: 1424–1434 10.1038/s41559-021-01535-8 34413507

[msb202110630-bib-0009] De Deken RH (1966) The Crabtree effect: a regulatory system in yeast. J Gen Microbiol 44: 149–156 5969497 10.1099/00221287-44-2-149

[msb202110630-bib-0010] Dienert F (1900) Sur la fermentation du galactose et sur l’accoutumance des levures à ce sucre. Ann Inst Pasteur 14: 139–189

[msb202110630-bib-0011] Enke TN , Datta MS , Schwartzman J , Cermak N , Schmitz D , Barrere J , Pascual‐García A , Cordero OX (2019) Modular assembly of polysaccharide‐degrading marine microbial communities. Curr Biol 29: 1528–1535.e6 31031118 10.1016/j.cub.2019.03.047

[msb202110630-bib-0012] Erickson DW , Schink SJ , Patsalo V , Williamson JR , Gerland U , Hwa T (2017) A global resource allocation strategy governs growth transition kinetics of *Escherichia coli* . Nature 551: 119–123 29072300 10.1038/nature24299 PMC5901684

[msb202110630-bib-0013] Estrela S , Vila JCC , Lu N , Bajić D , Rebolleda‐Gómez M , Chang C‐Y , Goldford JE , Sanchez‐Gorostiaga A , Sánchez Á (2022) Functional attractors in microbial community assembly. Cell Syst 13: 29–42.e7 34653368 10.1016/j.cels.2021.09.011 PMC8800145

[msb202110630-bib-0014] Friedman J , Higgins LM , Gore J (2017) Community structure follows simple assembly rules in microbial microcosms. Nat Ecol Evol 1: 109 28812687 10.1038/s41559-017-0109

[msb202110630-bib-0015] Friesen ML , Saxer G , Travisano M , Doebeli M (2004) Experimental evidence for sympatric ecological diversification due to frequency‐dependent competition in *Escherichia coli* . Evolution 58: 245–260 15068343

[msb202110630-bib-0016] Fu H , Uchimiya M , Gore J , Moran MA (2020) Ecological drivers of bacterial community assembly in synthetic phycospheres. Proc Natl Acad Sci USA 117: 3656–3662 32015111 10.1073/pnas.1917265117 PMC7035482

[msb202110630-bib-0017] Gauss GF (1934) The struggle for existence. New York, NY: Hafner Press

[msb202110630-bib-0018] Görke B , Stülke J (2008) Carbon catabolite repression in bacteria: many ways to make the most out of nutrients. Nat Rev Microbiol 6: 613–624 18628769 10.1038/nrmicro1932

[msb202110630-bib-0019] Goyal A , Dubinkina V , Maslov S (2018) Multiple stable states in microbial communities explained by the stable marriage problem. ISME J 12: 2823–2834 30022156 10.1038/s41396-018-0222-x PMC6246551

[msb202110630-bib-0020] Harder W , Dijkhuizen L (1982) Strategies of mixed substrate utilization in microorganisms. Philos Trans R Soc Lond B Biol Sci 297: 459–480 6180444 10.1098/rstb.1982.0055

[msb202110630-bib-0021] Hardin G (1960) The competitive exclusion principle. Science 131: 1292–1297 14399717 10.1126/science.131.3409.1292

[msb202110630-bib-0022] Hermsen R , Okano H , You C , Werner N , Hwa T (2015) A growth‐rate composition formula for the growth of *E. coli* on co‐utilized carbon substrates. Mol Syst Biol 11: 801 25862745 10.15252/msb.20145537 PMC4422558

[msb202110630-bib-0023] Hutchinson GE (1961) The paradox of the plankton. Am Nat 95: 137–145

[msb202110630-bib-0024] Lee H , Gore J , Korolev KS (2022) Slow expanders invade by forming dented fronts in microbial colonies. Proc Natl Acad Sci USA 119: e2108653119 34983839 10.1073/pnas.2108653119 PMC8740590

[msb202110630-bib-0025] Lemoigne M , Aubert J , Millet J (1954) La production d’alcool et le rendement de croissance de la levure de boulangerie cultivée en aérobiose [Ethyl alcohol production and growth of baker’s yeast cultured under aerobic conditions]. Ann Inst Pasteur 87: 427–439 13218380

[msb202110630-bib-0026] Loomis WF , Magasanik B (1967) Glucose‐lactose diauxie in *Escherichia coli* . J Bacteriol 93: 1397–1401 5340309 10.1128/jb.93.4.1397-1401.1967 PMC276614

[msb202110630-bib-0027] Manhart M , Adkar BV , Shakhnovich EI (2018) Trade‐offs between microbial growth phases lead to frequency‐dependent and non‐transitive selection. Proc R Soc B Biol Sci 285: 20172459 10.1098/rspb.2017.2459 PMC5829199 29445020

[msb202110630-bib-0028] Manhart M , Shakhnovich EI (2018) Growth tradeoffs produce complex microbial communities on a single limiting resource. Nat Commun 9: 1–9 30097583 10.1038/s41467-018-05703-6 PMC6086922

[msb202110630-bib-0029] McGill SL , Yung Y , Hunt KA , Henson MA , Hanley L , Carlson RP (2021) Pseudomonas aeruginosa reverse diauxie is a multidimensional, optimized, resource utilization strategy. Sci Rep 11: 1457 33446818 10.1038/s41598-020-80522-8 PMC7809481

[msb202110630-bib-0030] Monod J (1941) Recherches sur la croissance des cultures bactériennes. Doctoral thesis. Université de Paris, Paris

[msb202110630-bib-0031] Monod J (1949) The growth of bacterial cultures. Annu Rev Microbiol 3: 371–394

[msb202110630-bib-0032] Monod J (1966) From enzymatic adaptation to allosteric transitions. Science 154: 475–483 5331094 10.1126/science.154.3748.475

[msb202110630-bib-0033] Nadell CD , Bassler BL (2011) A fitness trade‐off between local competition and dispersal in *Vibrio cholerae* biofilms. Proc Natl Acad Sci USA 108: 14181–14185 21825170 10.1073/pnas.1111147108 PMC3161532

[msb202110630-bib-0034] New AM , Cerulus B , Govers SK , Perez‐Samper G , Zhu B , Boogmans S , Xavier JB , Verstrepen KJ (2014) Different levels of catabolite repression optimize growth in stable and variable environments. PLoS Biol 12: 1001764 10.1371/journal.pbio.1001764 PMC3891604 24453942

[msb202110630-bib-0035] Pacheco AR , Osborne ML , Segrè D (2021) Non‐additive microbial community responses to environmental complexity. Nat Commun 121: 1–11 10.1038/s41467-021-22426-3 PMC8062479 33888697

[msb202110630-bib-0036] Perrin E , Ghini V , Giovannini M , Di Patti F , Cardazzo B , Carraro L , Fagorzi C , Turano P , Fani R , Fondi M (2020) Diauxie and co‐utilization of carbon sources can coexist during bacterial growth in nutritionally complex environments. Nat Commun 11: 1–16 32561713 10.1038/s41467-020-16872-8 PMC7305145

[msb202110630-bib-0037] Posfai A , Taillefumier T , Wingreen NS (2017) Metabolic trade‐offs promote diversity in a model ecosystem. Phys Rev Lett 118: 28103 10.1103/PhysRevLett.118.028103 PMC5743855 28128613

[msb202110630-bib-0038] Sandberg TE , Lloyd CJ , Palsson BO , Feist AM (2017) Laboratory evolution to alternating substrate environments yields distinct phenotypic and genetic adaptive strategies. Appl Environ Microbiol 83: e00410‐17 28455337 10.1128/AEM.00410-17 PMC5478979

[msb202110630-bib-0039] Solopova A , Van Gestel J , Weissing FJ , Bachmann H , Teusink B , Kok J , Kuipers OP (2014) Bet‐hedging during bacterial diauxic shift. Proc Natl Acad Sci USA 111: 7427–7432 24799698 10.1073/pnas.1320063111 PMC4034238

[msb202110630-bib-0040] Spencer CC , Bertrand M , Travisano M , Doebeli M (2007) Adaptive diversification in genes that regulate resource use in *Escherichia coli* . PLoS Genet 3: e15 17238290 10.1371/journal.pgen.0030015 PMC1779306

[msb202110630-bib-0041] Taheri‐Araghi S , Bradde S , Sauls JT , Hill NS , Levin PA , Paulsson J , Vergassola M , Jun S (2015) Cell‐size control and homeostasis in bacteria. Curr Biol 25: 385–391 25544609 10.1016/j.cub.2014.12.009 PMC4323405

[msb202110630-bib-0042] Tilman D (1982) Resource competition and community structure. Princeton, NJ: Princeton University Press 7162524

[msb202110630-bib-0043] Tuncil YE , Xiao Y , Porter NT , Reuhs BL , Martens EC , Hamaker BR (2017) Reciprocal prioritization to dietary glycans by gut bacteria in a competitive environment promotes stable coexistence. MBio 8: e01068‐17 29018117 10.1128/mBio.01068-17 PMC5635687

[msb202110630-bib-0044] Venturelli OS , Zuleta I , Murray RM , El‐Samad H (2015) Population diversification in a yeast metabolic program promotes anticipation of environmental shifts. PLOS Biol 13: e1002042 25626086 10.1371/journal.pbio.1002042 PMC4307983

[msb202110630-bib-0045] Vermeersch L , Perez‐Samper G , Cerulus B , Jariani A , Gallone B , Voordeckers K , Steensels J , Verstrepen KJ (2019) On the duration of the microbial lag phase. Curr Genet 65: 721–727 30666394 10.1007/s00294-019-00938-2 PMC6510831

[msb202110630-bib-0046] Wang J , Atolia E , Hua B , Savir Y , Escalante‐Chong R , Springer M (2015) Natural variation in preparation for nutrient depletion reveals a cost‐benefit tradeoff. PLOS Biol 13: e1002041 25626068 10.1371/journal.pbio.1002041 PMC4308108

[msb202110630-bib-0047] Wang T , Goyal A , Dubinkina V , Maslov S (2019) Evidence for a multi‐level trophic organization of the human gut microbiome. PLoS Comput Biol 15: e1007524 31856158 10.1371/journal.pcbi.1007524 PMC6922320

[msb202110630-bib-0048] Wang Z , Goyal A , Dubinkina V , George AB , Wang T , Fridman Y , Maslov S (2021) Complementary resource preferences spontaneously emerge in diauxic microbial communities. Nat Commun 121: 1–12 10.1038/s41467-021-27023-y PMC8602314 34795267

[msb202110630-bib-0049] Wolfe AJ (2005) The acetate switch. Microbiol Mol Biol Rev 69: 12–50 15755952 10.1128/MMBR.69.1.12-50.2005 PMC1082793

[msb202110630-bib-0050] Yawata Y , Cordero OX , Menolascina F , Hehemann JH , Polz MF , Stocker R (2014) Competition‐dispersal tradeoff ecologically differentiates recently speciated marine bacterioplankton populations. Proc Natl Acad Sci USA 111: 5622–5627 24706766 10.1073/pnas.1318943111 PMC3992678

[msb202110630-bib-0051] You C , Okano H , Hui S , Zhang Z , Kim M , Gunderson CW , Wang YP , Lenz P , Yan D , Hwa T (2013) Coordination of bacterial proteome with metabolism by cyclic AMP signalling. Nature 500: 301–306 23925119 10.1038/nature12446 PMC4038431

