# Supplementary material for: Diauxic lags explain unexpected coexistence in multi‐resource environments
Source: Mol Syst Biol. 2022 May 4;18(5):e10630. doi: 10.15252/msb.202110630 (PMC9067609; doi:10.15252/msb.202110630)
Supplement: Supplementary file 1 — Appendix [file MSB-18-e10630-s003.pdf]

Appendix

for

Diauxic lags explain unexpected coexistence in multi-resource environments

Blox Bloxham[1], Hyunseok Lee[1], Jeff Gore[1]
[1]Physics of Living Systems, Department of Physics, Massachusetts Institute of Technology, Cambridge MA, USA

Contents

1. Appendix Figures S1 – S13 **3**
Appendix Figure S1. Two-resource growth rates do not vary with resource supply ratio. 3
Appendix Figure S2. A quadratic growth rate recovery shape allows for more confident lag time fits than a linear or a sharp recovery. 4
Appendix Figure S3. All Aci2 lag time fits. 5
Appendix Figure S4. All Pa lag time fits. 6
Appendix Figure S5. Small spikes in Pa's optical density at the onset of its diauxic shift and at saturation appear to be artifacts and can be easily accounted for in the data analysis. . . . 7
Appendix Figure S6. Detail on Aci2's post-shift growth rates. 8
Appendix Figure S7. Lag times fit using single-resource growth rates for post-shift steady-state growth rates are similar to the lag times fit using the two-resource growth rates. 9
Appendix Figure S8. Aci2's lag times do not vary with initial dilution or time spent growing pre-shift, but may be a function of the alanine supply or of Aci2's population size at the onset of its diauxic shift. 10
Appendix Figure S9. Aci2 vs Pa on alanine and/or glutamate competition data including single-resource conditions. 11
Appendix Figure S10. Quantification of Aci2 and Pa on alanine and glutamate model prediction vs experimentally observed outcome. 12
Appendix Figure S11. Removing the assumption of equal pre- and post-shift growth rates changes modeling predictions only slightly. 13
Appendix Figure S12. Predicted competitive outcomes vary relatively little with Pa's resource preference. 13
Appendix Figure S13. Images of the colonies formed by each of the five species. 14

2. Modeling Comparison of Initial and Diauxic Lags **15**
2.1 Daily dilution competitions 15
2.2 Exponential growth with diauxic lags 17
In the simplest diauxie model coexistence occurs in an unbounded region of large growth rate and lag time differences. 17

When species have opposite preferences, coexistence occurs in an unbounded region of large lag times but does not require any difference in lag times. 20
2.3 Exponential growth with initial lags 21
With all yields equal, initial lags do not allow for coexistence. 21
Initial-lag coexistence requires disequal yields and dilutions to a total population size and occurs only in a narrow parameter region. 21
2.4 Three species on two resources with either initial or diauxic lags 24
Up to three species can coexist on two resources with diauxic lags and dilutions to a total population size. 24
It is plausible that three species could coexist on two resources with initial lags, dilutions to a total population size, and separate growth rates for each resource. 26
2.5 Likelihood of random species coexisting with either initial or diauxic lags 27
With separate growth rates for each resource and constant-factor dilutions, at most two species can survive with diauxic lags, initial lags, or no lags, but the frequency of coexistence may still vary. 27
Invasibility can be used as an approximation of whether species coexist. 28
With random species from reasonable distributions, coexistence is more likely if species have diauxic lags than if they have initial. 30
Initial lags benefit coexistence at small to intermediate lag time values, while diauxic lags benefit coexistence at large lag time values. 31
2.6 Summary: Diauxic lags support coexistence to a greater extent than initial lags 32

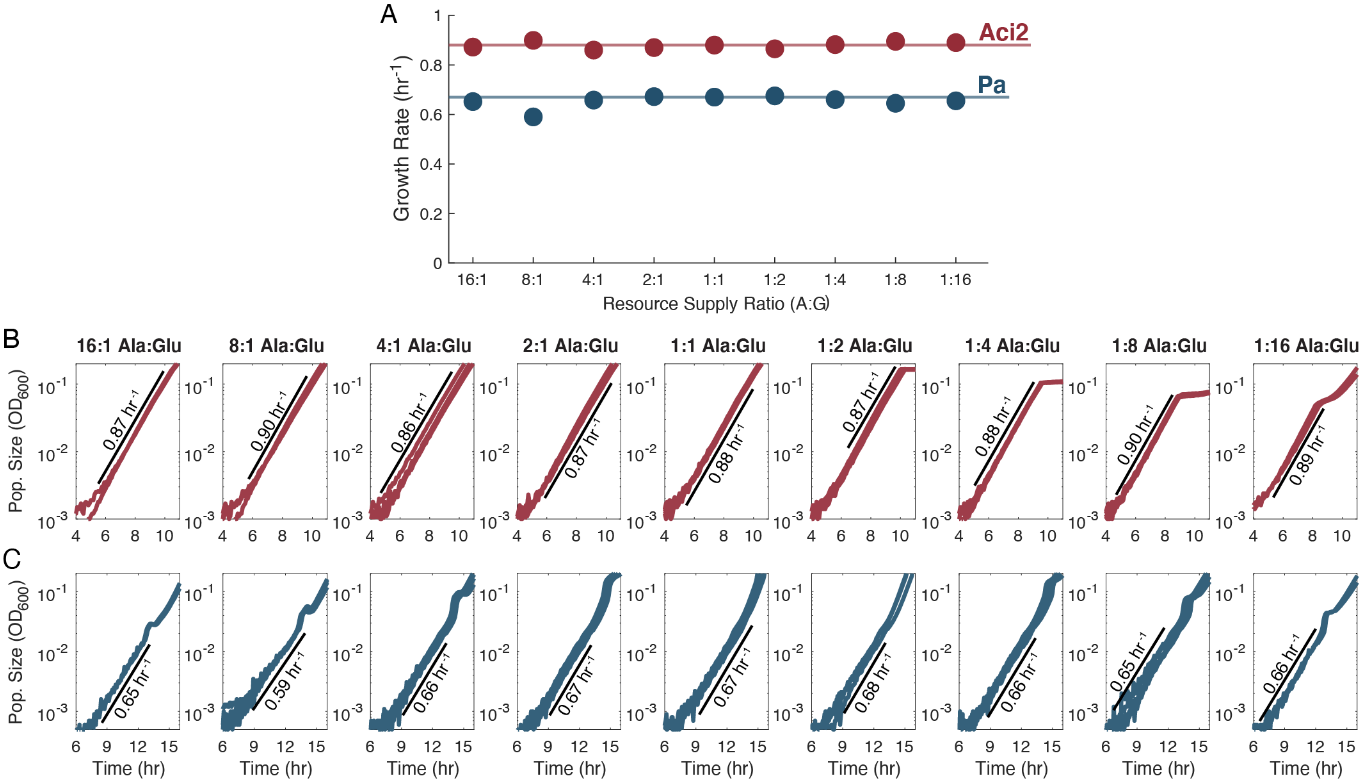

Appendix Figure S1. Two-resource growth rates do not vary with resource supply ratio. (A) Growth rates for Aci2 and Pa at nine different ratios of alanine and glutamate (total supply constant at 0.1%w/v). Aci2's growth rate fluctuates by ± 0.01 hr^{-1} and Pa's by ± 0.03 hr^{-1} (standard deviations of the nine growth rate fits), compared to uncertainties of approximately ± 0.01 hr^{-1} on each individual fit. **(B)** The Aci2 optical density data from Fig 2B in red with the Aci2 growth rates from **A** in black. **(C)** The Pa optical density data from Fig 2E in blue with the Pa growth rates from **A** in black.

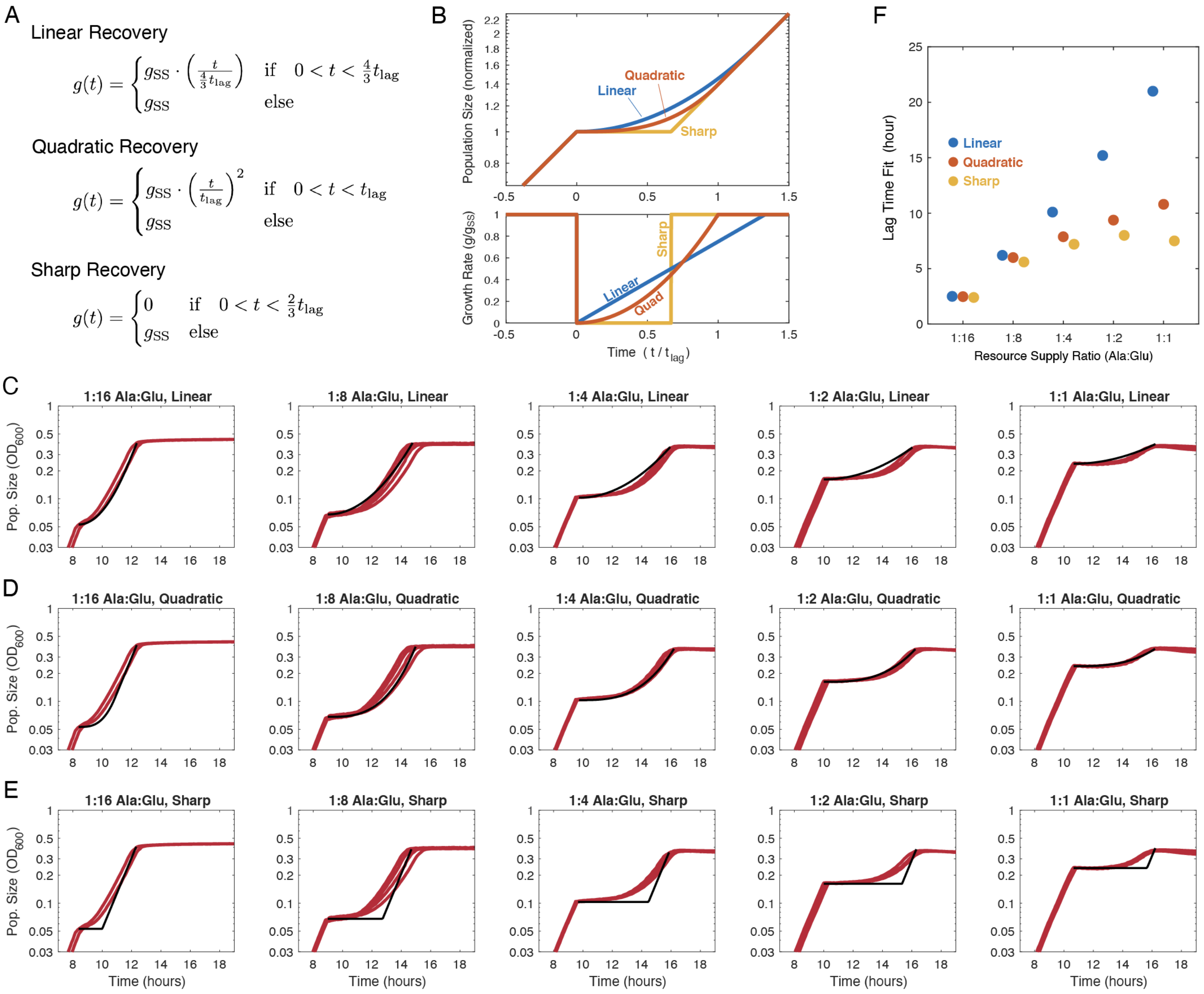

Appendix Figure S2. A quadratic growth rate recovery shape allows for more confident lag time fits than a linear or a sharp recovery. (A) The equations governing three simple empirical models for growth rate (and, after integration, population size) over time after a diauxic shift that starts at time $t = 0$. The steady-state growth rate is g_{SS} and the lag time is t_{lag}. The prefactors in front of t_{lag} in the linear and sharp recovery shapes are included to have the population sizes from each shape converge at time $t \gg t_{\text{lag}}$. **(B)** The growth rates (bottom) and population sizes (top) derived from those shapes. The top panel illustrates how the quadratic growth rate shape can be thought of as in between the linear and sharp recovery shapes. **(C – D)** Lag time fits using the linear, quadratic, and sharp growth rate recovery shapes. In all fits $g_{SS} = 0.88\text{hr}^{-1}$, so the only free parameter is t_{lag} (fit independently for each recovery shape). The Aci2 optical density data from Fig 2B and EV2A is in the red and the best fit is in black. The quadratic fits in **D** are the same as Fig EV2A and reproduced here to facilitate comparison. **(C)** The linear recovery shape allows for close, confident fits at large glutamate supply fractions (two leftmost panels), but at more equal supply ratios (three center through rightmost panels) it is unclear whether the most appropriate fit would be a long lag time fit that predicts population sizes close to the experimental data before saturation is reached or a shorter lag time fit that would eventually converge

towards an extrapolation of the experimental data if saturation were not reached. **(E)** Similarly, the sharp recovery shape can be fit asymptotically at large glutamate supply fractions but becomes more uncertain at more equal supply ratios. **(F)** Comparing the lag times fit using each recovery shape reinforces these considerations. Using the linear recovery shape yields excessively long lag times, while the sharp recovery shape yields lag time values similar to the quadratic model but that actually decrease at the 1:1 Ala:Glu condition (relative to the 1:2 condition) due to the biases of using that recovery shape.

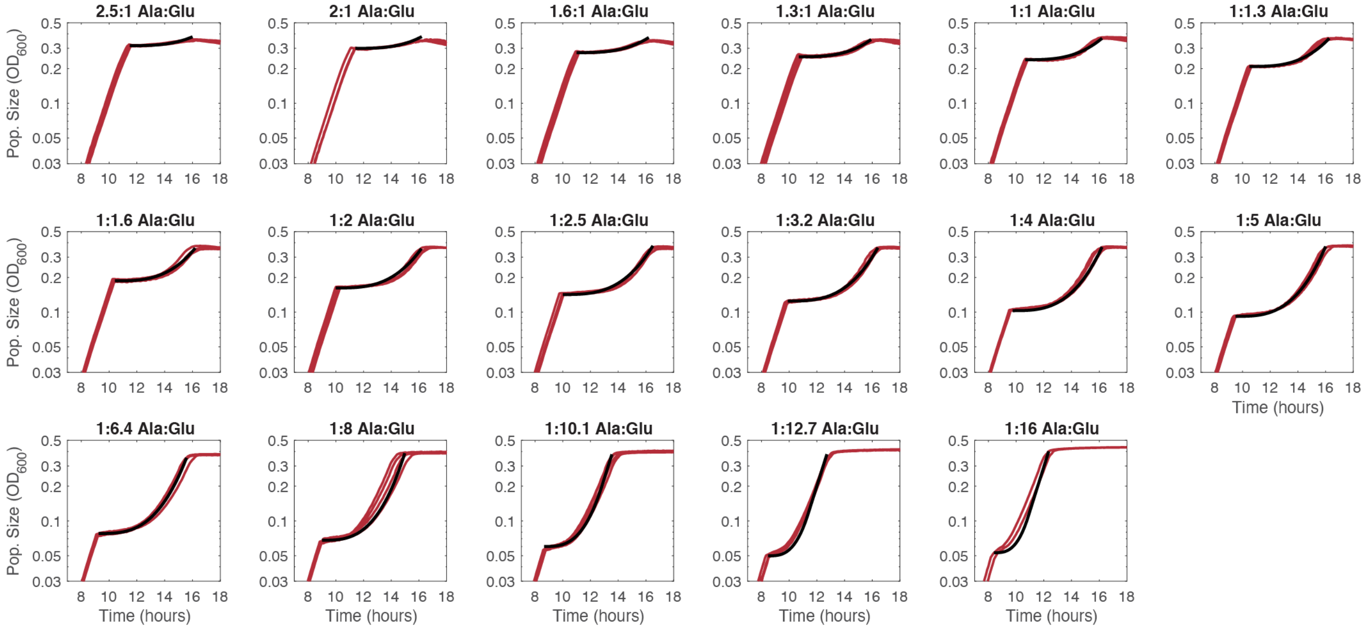

Appendix Figure S3. All Aci2 lag time fits. In all plots, the same data as in Fig 2B is shown in red and the best-fit lag recoveries (from which the reported lag times in Fig 2H are extracted) are shown in black.

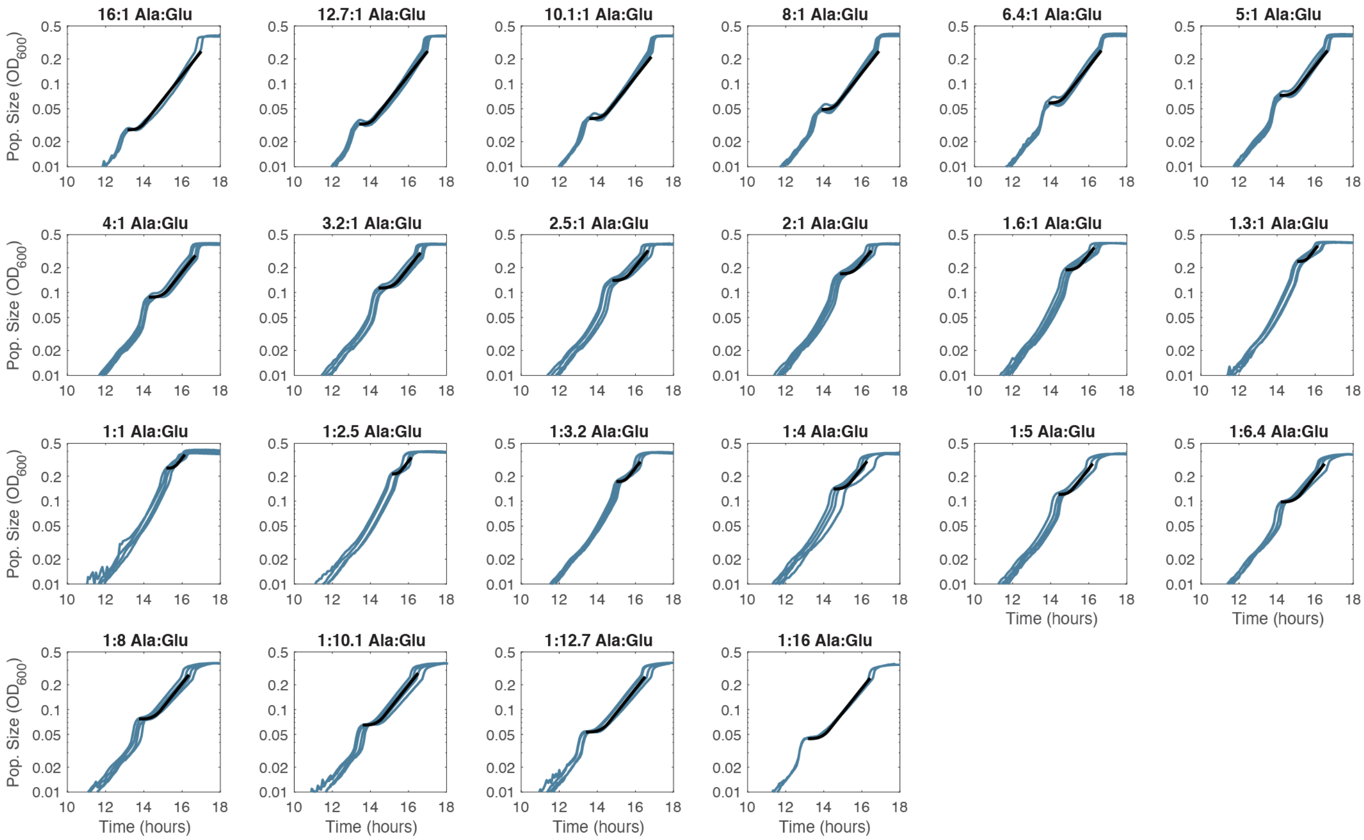

Appendix Figure S4. All Pa lag time fits. In all plots, the same data as in Fig 2E is shown in blue and the best-fit lag recoveries (from which the reported lag times are extracted) are shown in black. Sudden spikes in optical density when Pa undergoes a diauxic shift or saturates are visible in nearly all these plots. Interpretation and handling of these artifacts is detailed in Appendix Fig S5.

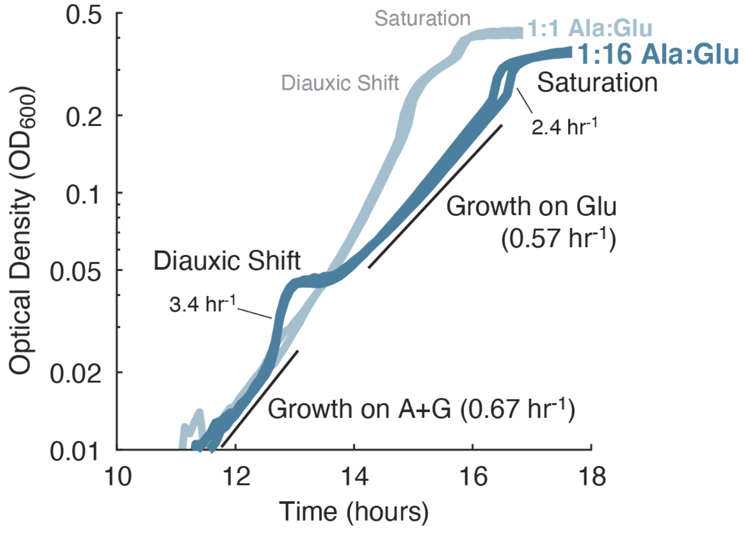

Appendix Figure S5. Small spikes in Pa's optical density at the onset of its diauxic shift and at saturation appear to be artifacts and can be easily accounted for in the data analysis. Shown is an annotated growth curve for Pa growing in a 1:16 Ala:Glu environment (dark) with a growth curve for Pa growing in a 1:1 environment shown for comparison (light and behind). Periods of steady-state growth can be seen first in the two-resource environment and later on the remaining glutamate. Pa's steady-state growth rate on the remaining glutamate matches the value of 0.57hr^{-1} from single-resource experiments (Fig EV1A). In addition to periods of steady growth, sudden spikes in optical density are also clearly visible. If these spikes corresponded to ordinary growth (i.e. increase in biomass), Pa's growth rate would reach as high as 3.4hr^{-1} at the time of its diauxic shift. This is implausible, so these spikes must be changes in per capita or per biomass optical density. We did not study these spikes in optical density any further, but sudden increases of similar magnitude to the optical density of Pseudomonas have been previously observed and linked to changes in cell morphology resulting from environmental perturbations.[1] For fitting Pa's lag times, the time for Pa to reach its steady-state growth rate (and not the time until the spike in optical density at saturation) is what's relevant (Materials and Methods). The greatest uncertainty in the Pa lag time fits comes from determining at what time and optical density to define the onset of Pa's diauxic shift. In the reported fits (Appendix Fig S4) we have defined the onset such that we obtain lag time values in the middle of the possible range. Other choices could have affected Pa's lag times by up to ±20 minutes, but Pa's lag times would still be very short compared to Aci2's and reasonably approximated by a constant value of 1 hour.

[1] Bernheim "Factors which affect the size and optical density of *Pseudomonas aeruginosa*" *J Gen Microbiol.* 1964.

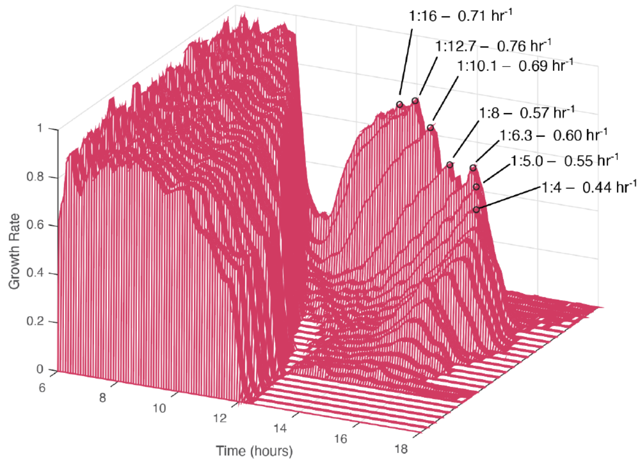

Appendix Figure S6. Detail on Aci2's post-shift growth rates. The same instantaneous growth rates as in Fig 2D are plotted in an alternative manner to show additional detail. Labels show the maximum instantaneous growth rates for each condition.

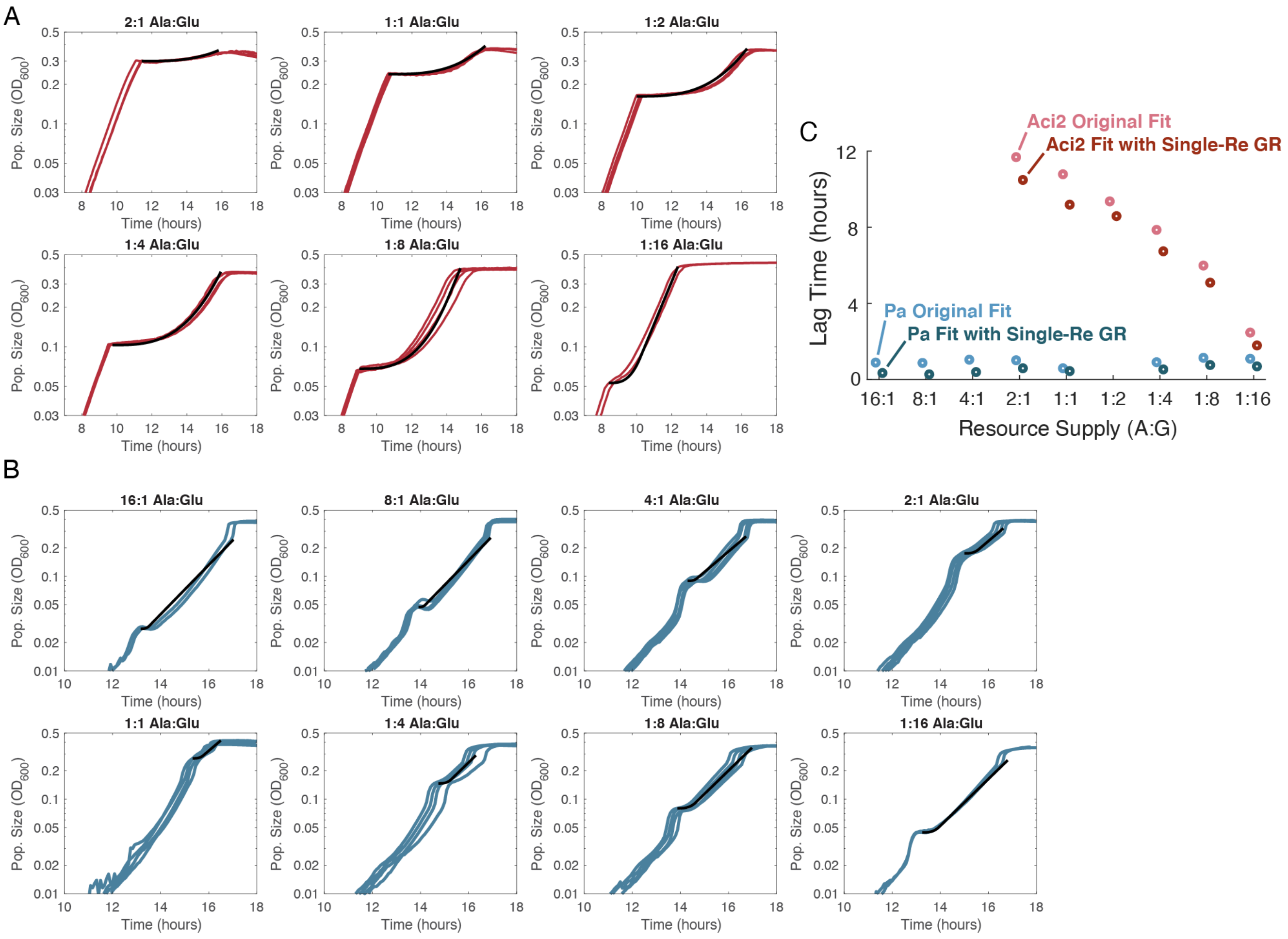

Appendix Figure S7. Lag times fit using single-resource growth rates for post-shift steady-state growth rates are similar to the lag times fit using the two-resource growth rates. The lag times used in all Main Text modeling were fit using g_{SS} (i.e. $g(t \gg t_{\text{lag}})$) values equal to the species' two-resource growth rates. This decision was made to maintain consistency with the modeling having only a single growth rate for each species. The measured single-resource growth rates for each species were, however, 15-25% slower than the two-resource growth rates (Fig EV1A). **(A–B)** Lag times were refit using the species' single-resource growth rates for their post-shift g_{SS}. The same Aci2 and Pa optical density data as in Fig 2B (and Appendix Fig 3 and 4) are shown in red and blue respectively, the updated best-fit lag recoveries (from which the lag times are extracted) are shown in black. **(C)** Summary of Aci2 (red) and Pa (blue) diauxic lag times as fit and modeled with in the Main Text narrative (light) and as refit in A and B using the species' single-resource growth rates (dark). Aci2's lag times are on average only 15% ±7% shorter. Pa's lag times are on average 54% ±16% shorter, but, because Pa's lag times are already short, this is only 28 ±4 minutes shorter. Across the five conditions with lag times for both Aci2 and Pa, the difference in lag times (i.e. $t_{\text{lag,Aci2}} - t_{\text{lag,Pa}}$) decreases by just 13% ±2%. Results from modeling with these updated lag times are provided in Appendix Fig S11. (Means and standard errors reported.)

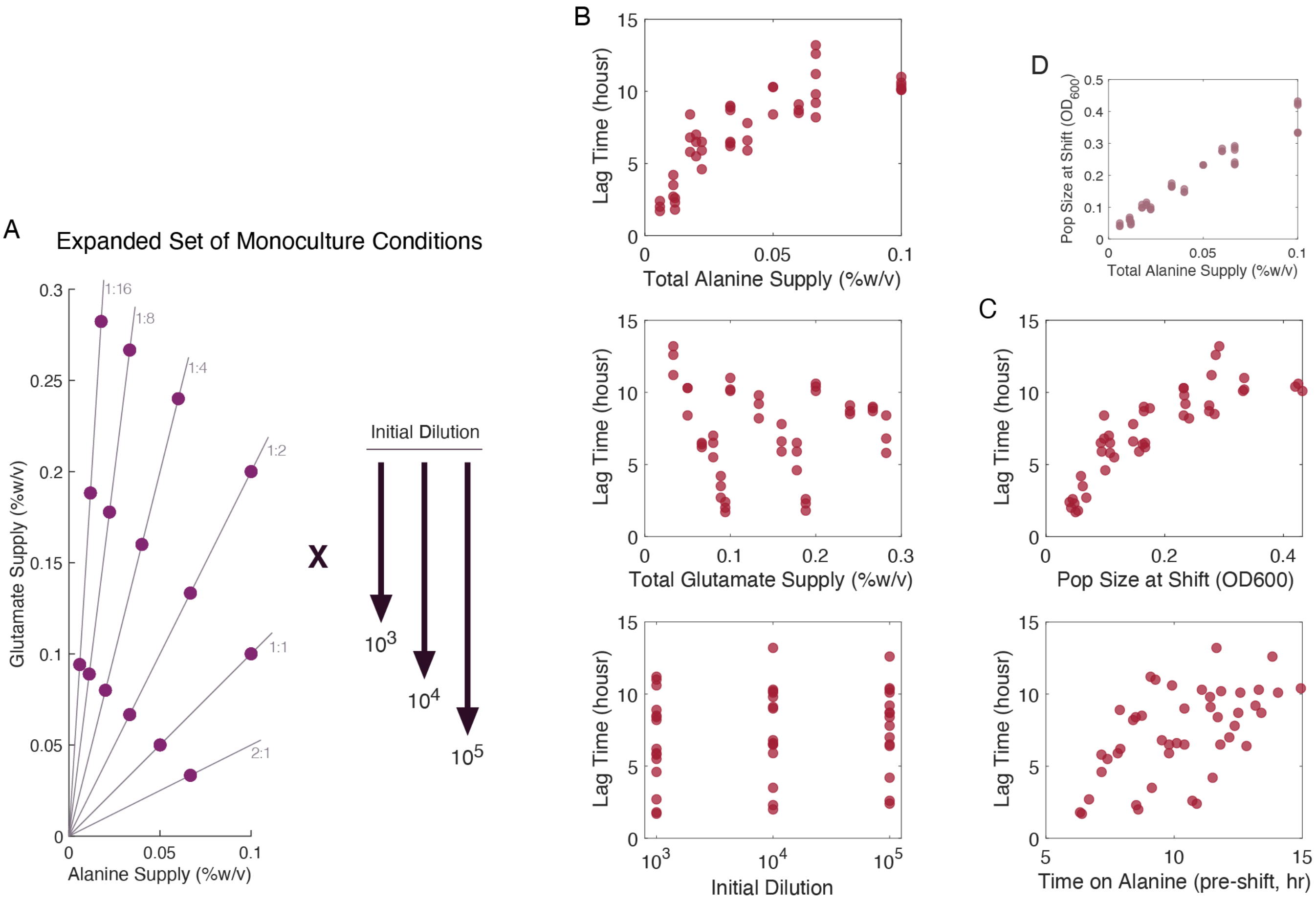

Appendix Figure S8. Aci2's lag times do not vary with initial dilution or time spent growing pre-shift, but may be a function of the alanine supply or of Aci2's population size at the onset of its diauxic shift. **(A)** To further investigate with which parameters Aci2's lag time truly varies we repeated the monoculture lag time measurements (Fig 2B-C) at an expanded set of conditions, defined by resource supply ratios of 1:16 through 2:1 Ala:Glu at 1x, 2x, and 3x the total resource concentrations used in the Main Text and initial dilutions of 10^3, 10^4, and 10^5 from an overnight starter culture. The plot indicates the alanine and glutamate supply combinations used. All 15 supplies were tested at each of the three dilutions. **(B)** Extracted lag times vs each of the three directly controlled parameters. Lag time appears to correlate well with alanine supply and notably has no variation with initial dilution. That lag time does not vary with initial dilution justifies modeling Aci2's lag time as a function of resource supply but not dilution factor. **(C)** Extracted lag times vs time and population size at the onset of the diauxic shift. Aci2's lag time correlates well with its population size at the onset of its diauxic shift but not with time spent growing on alanine before its diauxic shift. **(D)** Aci2's lag time correlates equally well to the alanine supply and to its population size at the onset of the diauxic shift because in monoculture the alanine supply determines Aci2's population size at the onset of its diauxic shift (at which point Aci2shas consumed all the alanine and nothing else). This correlation meant we could not rule out that Aci2's lag time might truly be a function of its population size at the onset of its diauxic shift. For simplicity the Main Text modeling assumes Aci2's lag time varies with the experimentally controlled parameter. Fig EV4 shows the results of instead modeling Aci2's lag time as varying with its population size at the onset of its diauxic shift.

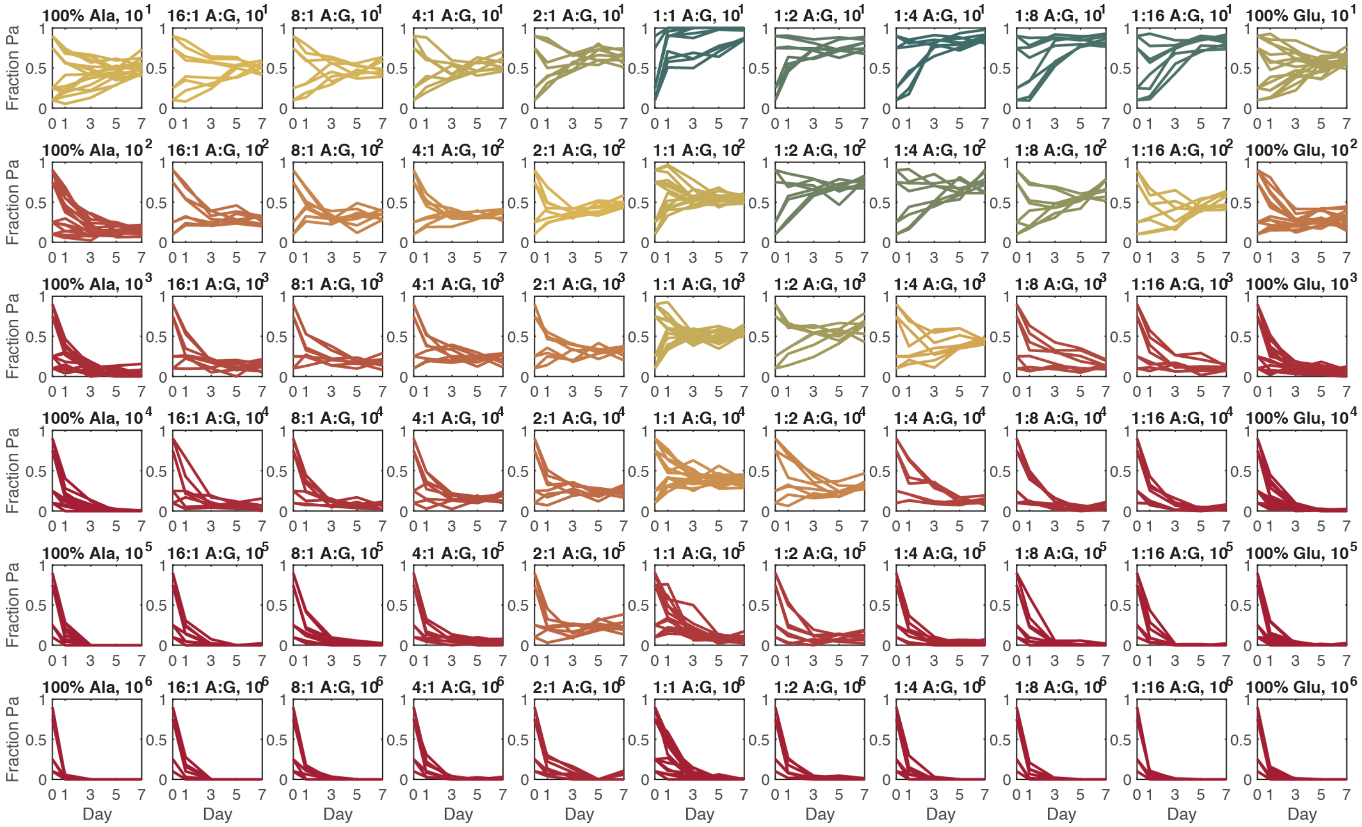

Appendix Figure S9. Aci2 vs Pa on alanine and/or glutamate competition data including single-resource conditions. Same as Fig 5A but including the single-resource competitions in the left-most and right-most columns. 146,566 colonies were counted to produce precise species fractions.

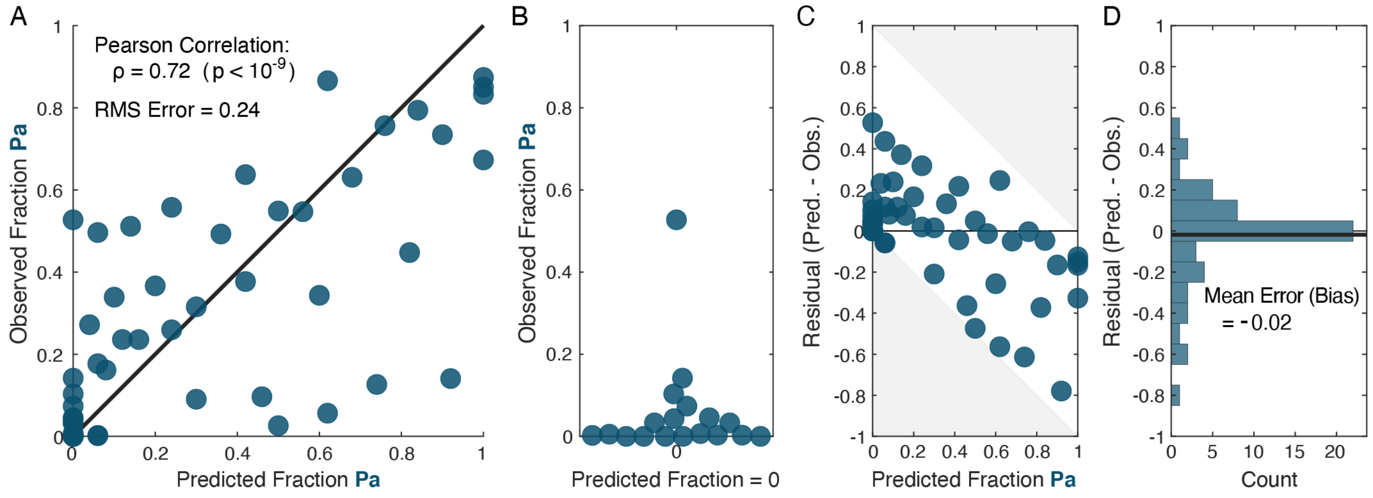

Appendix Figure S10. Quantification of Aci2 and Pa on alanine and glutamate model prediction vs experimentally observed outcome. **(A)** Steady-state predicted fraction Pa vs experimentally observed fraction Pa for all resource supply ratios and dilution factors tested. **(B)** Observed fraction Pa for all cases in which Pa is predicted to go extinct. Horizontal spacing is added to separate the data points that are otherwise stacked (e.g. in the bottom left corner of **A**). **(C)** Residual plot of the same data. Shaded gray regions are the disallowed regions that would require an observed fraction less than zero or greater than one. **(D)** Histogram of the residuals. Dark horizontal line near center indicates the mean residual, which is a species fraction of -0.02.

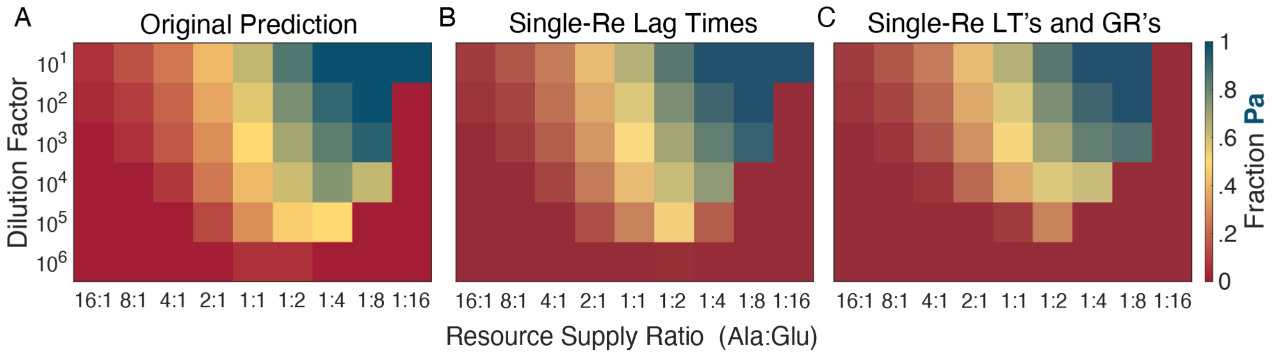

Appendix Figure S11. Removing the assumption of equal pre- and post-shift growth rates changes modeling predictions only slightly. (A) Same modeling prediction as the Main Text (i.e. same as Fig 5B) reproduced here to facilitate comparison. This modeling used the species' two-resource growth rates for both pre- and post-shift growth to have a single growth rate for each species and used lag times fit with post-shift g_{SS} fixed as the species' two-resource growth rates for consistency. **(B)** Same modeling repeated, still using two-resource growth rates pre- and post-shift, but now using the lag time values fit using single-resource growth rates for post-shift g_{SS} (Appendix Fig S7). **(C)** Modeling repeated again using single-resource growth rates post-shift (such that each species now has three total growth rate) as well as lag times fit using single-resource growth rates.

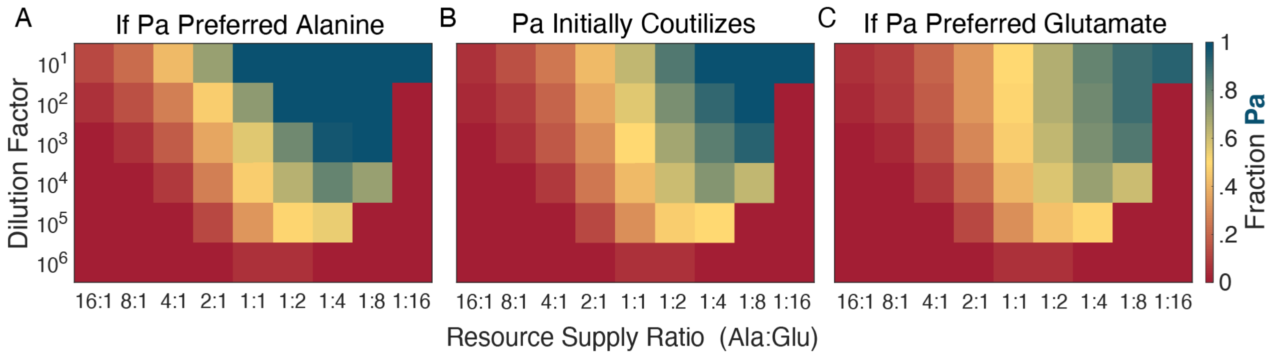

Appendix Figure S12. Predicted competitive outcomes vary relatively little with Pa's resource preference. (A) Pa modeled as initally consuming only alanine (i.e. having the same resource preference as Aci2). **(B)** Pa modeled as initially coutilizing with a 2:3 Ala:Glu uptake ratio. This is the same prediction presented in Fig 5B and is reproduced here to facilitate comparison. **(C)** Pa modeled as initially consuming only glutamate (i.e. having the opposite preference from Aci2). Across **A**−**C**, whether Pa survives never depends on its resource preference because as Pa is driven extinct it has increasingly little impact on the population's overall resource consumption and therefore on the resource depletion times and each species' per-cycle growth. This conservation of Pa's extinction or survival illustrates that the slow-grower Pa's ability to survive in coculture with the fast-grower Aci2 is not dependent on any complimentarity of resource preferences (e.g. on Aci2 initially consuming only alanine while Pa initially coutilizes with a larger uptake fraction of glutamate).

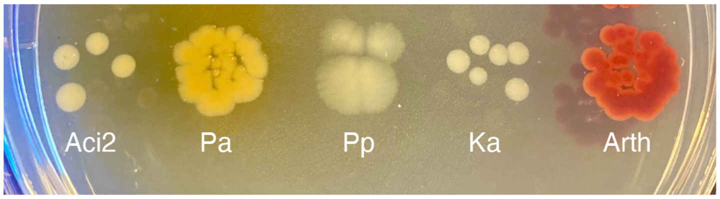

Appendix Figure S13. Images of the colonies formed by each of the five species. The color difference between Aci2 and Ka is becomes more apparent under a transmitted light microscope with Aci2 being considerably more opaque than Ka.

2. Modeling Comparison of Initial and Diauxic Lags

Blox Bloxham, Hyunseok Lee, Jeff Gore

In the Main Text we state that diauxic lags have a greater ability to support coexistence than do initial lags because diauxic lags divide the growth phase into two subphases with their own distinct dynamics whereas initial lags merely affect the dynamics of a single growth phase. This modeling comparison is presented to support those claims. We begin by looking at the simplest models of initial and diauxic lags under otherwise exponential growth and find that diauxic lags support coexistence within simpler models than those necessary for initial lags to produce coexistence. We specifically show that diauxic lags can support coexistence as the result of a growth rate − lag time tradeoff whereas initial lags require a more complex growth rate − lag time − yield tradeoff to produce coexistence. We also show that diauxic lags produce coexistence in larger parameter regions, which implies greater stability against perturbations. Finally, we sample random species in a scenario in which both initial and diauxic lags can support the coexistence of two species and see coexistence occur more frequently when lags are diauxic. These results lead us to conclude that from a variety of perspectives diauxic lags do indeed support coexistence to a greater extent than initial lags.

2.1 Daily dilution competitions

All modeling presented in the Main Text and Appendix considers the case of two or three species competing for one or two supplied resources under a daily dilution scheme. Our central question is determining which species survive and at what (end-of-day) population sizes after a large number of dilution cycles (also referred to as 'days'). We begin by defining the basics of the daily dilution:

Resources are supplied at constant quantities $\{s_i\}$ at the start of each day. Concentrations are $\{c_i(t)\}$.

Species population sizes are $\{n_\mu(t)\}$, occasionally labeled as $\{n_\mu^{(\text{day } i)}(t)\}$ when that clarity is needed. (The time t is time-within-a-day.) Species begin each day at some population sizes $\{n_\mu^{(\text{day } i)}(0)\}$ and grow until all resources have been depleted (at t_{sat}), at which point the coculture is saturated and population sizes remain constant for the rest of the day.[1]

At the start of the next day, population sizes are diluted. There are two forms of dilution we will consider: constant-factor dilutions and dilutions to a specific total population size.

[1]For clarity, throughout the Appendix, species will be α, β, and γ and indexed over with μ and ν, while resources will be R_1 and R_2 and indexed with i and j.

A **constant-factor dilution** obeys a simple form,

$$n_\mu^{(\text{day } i)}(0) = \frac{1}{\text{DF}} n_\mu^{(\text{day } i-1)}(t_\text{sat}) \tag{1}$$

where DF is the dilution factor and t_sat is the time at which the population saturates (meaning $\{n_\mu(t_\text{sat})\}$ are the end-of-day population sizes). Under constant-factor dilution, the dilution of each species μ is independent of the population sizes of any other species. This form of dilution captures the common experimental daily dilution scheme in which a well-mixed coculture is diluted once per day by transferring a small volume into fresh media.

A useful thing to know about a constant-factor dilution is the steady-state population sizes if total growth over the course of a day is known. Derived using ys as the total growth or the course of a day (which will often be something like yield times resource supply):

$$\sum_\mu n_\mu(t_\text{sat}) = \sum_\mu n_\mu(0) + ys \qquad \& \qquad \sum_\mu n_\mu(0) = \frac{1}{\text{DF}} \sum_\mu n_\mu(t_\text{sat})$$

$$\sum_\mu n_\mu(0) = \frac{ys}{\text{DF}-1} \qquad \& \qquad \sum_\mu n_\mu(t_\text{sat}) = \frac{\text{DF}\ ys}{\text{DF}-1} \tag{2}$$

Equation (2) will later be referred to the correction for the day-to-day carryover.

An alternative modeling of a daily dilution is a **dilution to a specific population size** n_0 while maintaining constant species population fractions:

$$\frac{n_\mu^{(\text{day } i)}(0)}{\sum_\nu n_\nu^{(\text{day } i)}(0)} = \frac{n_\mu^{(\text{day } i-1)}(t_\text{sat})}{\sum_\nu n_\nu^{(\text{day } i-1)}(t_\text{sat})} \qquad \& \qquad \sum_\mu n_\mu^{(\text{day } i)}(0) = n_0 \tag{3}$$

Equation (3) is satisfied by

$$n_\mu^{(\text{day } i)}(0) = \frac{n_0}{\sum_\nu n_\nu^{(\text{day } i-1)}(t_\text{sat})} n_\mu^{(\text{day } i-1)}(t_\text{sat}) \ . \tag{4}$$

So the effective dilution factor at the start of each day is

$$\text{DF}_\text{eff} = \frac{\sum_\mu n_\mu(t_\text{sat})}{n_0} \ . \tag{5}$$

If the total end-of-day population size $\sum_\mu n_\mu(t_\text{sat})$ is known to be the same each day regardless of the specific species fractions (e.g. if all yields are constant, as will be discussed later), then the two implementations of dilution are equivalent. But, when DF_eff varies with the population composition, the behavior of the two implementations becomes much different, with a reset to a specific total population size acting like another frequency-dependent selection phase that can allow for additional coexistence.

It certain contexts, the dilution to a specific population size can be thought of as a frequency-dependent dilution. For example, at fixed point two species (α and β) with yields y_α and y_β growing on a resource supplied in quantity s on each day obey

$$n_\alpha(0) = \frac{n_\alpha(t_\text{sat})}{\text{DF}_\text{eff}} \quad \& \quad n_\beta(0) = \frac{n_\beta(t_\text{sat})}{\text{DF}_\text{eff}} \quad \& \quad \frac{n_\alpha(t_\text{sat}) - n_\alpha(0)}{y_\alpha} + \frac{n_\beta(t_\text{sat}) - n_\beta(0)}{y_\beta} = s_1 \ . \tag{6}$$

Equation (6) along with $n_\alpha(0) + n_\beta(0) = n_0$ can be solved for DF_eff to get

$$\text{DF}_\text{eff} = 1 + \frac{y_\alpha y_\beta (s/n_0)}{y_\beta + (y_\alpha - y_\beta) f_\beta} \ , \tag{7}$$

where $f_\beta \equiv n_\beta(0)/(n_\alpha(0) + n_\beta(0))$ is the frequency or population fraction of species β. As DF_eff is now written as a function of f_β, the dilution to a specific population size can (with certain other assumptions) be thought of as having a frequency-dependent dilution factor.

The ability of the population composition to modulate DF_eff is important because, if some species do better in competition at small DF_eff and others at large DF_eff, then DF_eff can be the environmental parameter that mediates a negative frequency-dependent selection and stabilizes coexistence. This occurs in the growth rate – initial lag – yield tradeoff in section 2.3.

2.2 Exponential growth with diauxic lags

In the simplest diauxie model coexistence occurs in an unbounded region of large growth rate and lag time differences. We begin by studying the simplest case of diauxic lags and show that coexistence is readily possible in even the simplest model. We define this simplest case as two species with the same resource preference and the same growth rate for each resource. Specifically:

- Two species, α and β, compete for two resources, R_1 and R_2, supplied at concentrations $s_1 + s_2 = 1$.
- Each species has the same growth rate on each resource ($g_{\alpha 1} = g_{\alpha 2}$ and $g_{\beta 1} = g_{\beta 2}$).
- Each species consumes entirely R_1 then R_2.
- All yields are equal ($y_{\mu i} = 1$).
- Diauxic lags are sharp (defined below).
- Dilutions are constant-factor dilutions by DF.

The two species grow at rates g_α and g_β, consuming one dimensionless unit of R_1 per dimensionless unit of biomass gained until R_1 has been depleted ($\Delta n_\alpha + \Delta n_\beta = s_1$). At this point the species experience diauxic lags with no growth for $t_{\text{lag}\alpha}$ and $t_{\text{lag}\beta}$ and then grow until R_2 has also been depleted. At this point populations are divided by DF, resource concentrations are reset to s_1 and s_2, and the cycle repeats. This model is essentially the Main Text model except species are purely diauxic (no coutilization) and lags are 'sharp' (no growth for $t_{\text{lag}\mu}$ instead of a quadratic growth rate recovery).

The growth dynamics for species μ growing on R_i then R_j with a sharp diauxic lag of length $t_{\text{lag}\mu}$ (and no initial lag) are defined by

$$\frac{dn_\mu}{dt} = \tilde{g}_\mu(t) n_\mu(t) \quad \text{with} \quad \tilde{g}_\mu(t) = \begin{cases} g_{\mu i} & \text{if } 0 < t < t_{\text{dep}i} \\ 0 & \text{if } t_{\text{dep}i} < t < (t_{\text{dep}i} + t_{\text{lag}\mu}) \\ g_{\mu j} & \text{if } (t_{\text{dep}i} + t_{\text{lag}\mu}) < t < t_{\text{dep}j} \end{cases} \ , \tag{8}$$

where $\tilde{g}_\mu(t)$ has been introduced as the instantaneous growth rate of species μ and $t_{\text{dep}i}$ is the time at which resource R_i runs out (i.e. $c_i(t) = 0$ based on equation (10) defined below). This definition means

that species grow at a constant rate all the way until resource depletion, have no growth for the duration of their lag, then resume growing at their new maximum growth rate.

In the simple case being considered for now, the instantaneous growth rate can be simplified to

$$\tilde{g}_\mu(t) = \begin{cases} g_\mu & \text{if } 0 < t < t_{\text{dep1}} \\ 0 & \text{if } t_{\text{dep1}} < t < (t_{\text{dep1}} + t_{\text{lag}\mu}) \\ g_\mu & \text{if } (t_{\text{dep1}} + t_{\text{lag}\mu}) < t < t_{\text{dep2}} \end{cases} , \tag{9}$$

because species have only a single growth rate g_μ and will always be switching from R_1 to R_2.

Meanwhile, the dynamic equations for the resource concentrations are

$$\frac{dc_1}{dt} = \begin{cases} -\sum_\mu \frac{dn_\mu}{dt} & \text{if } 0 < t < t_{\text{dep1}} \\ 0 & \text{else} \end{cases} \quad \text{and} \quad \frac{dc_2}{dt} = \begin{cases} -\sum_\mu \frac{dn_\mu}{dt} & \text{if } t_{\text{dep1}} < t < t_{\text{dep2}} \\ 0 & \text{else} \end{cases} , \tag{10}$$

because both species are consuming R_1 then R_2 with all yields equal to one.

We can **determine whether species coexist** using fixed point analysis. On each cycle species grow by

$$\Delta \log n_\mu \equiv \log\left(\frac{n_\mu(t_{\text{sat}})}{n_\mu(0)}\right) = \begin{cases} g_\mu(t_{\text{dep2}} - t_{\text{lag}\mu}) & \text{if } t_{\text{lag}\mu} < (t_{\text{dep2}} - t_{\text{dep1}}) \\ g_\mu(t_{\text{dep1}}) & \text{else} \end{cases} . \tag{11}$$

With a constant factor dilution, a fixed point is defined by

$$\Delta \log n_\alpha = \Delta \log n_\beta = \log \text{DF} , \tag{12}$$

which is a simple statement that species' growth during each day is by exactly the same factor as they are then diluted by between days.

Equations (11) and (12) combine with an important consequence: No fixed point exists in which both species finish their diauxic lags. If $t_{\text{lag}\alpha} < (t_{\text{dep2}} - t_{\text{dep1}})$ and $t_{\text{lag}\beta} < (t_{\text{dep2}} - t_{\text{dep1}})$ then solving equation (12) requires,

$$g_\alpha(t_{\text{dep2}} - t_{\text{lag}\alpha}) = \log \text{DF} \quad \& \quad g_\beta(t_{\text{dep2}} - t_{\text{lag}\beta}) = \log \text{DF} ,$$

which cannot be satisfied by any value of t_{dep2}.

The case in which α does not finish its lag but β does can be solved:

$$g_\alpha(t_{\text{dep1}}) = \log \text{DF} \quad \& \quad g_\beta(t_{\text{dep2}} - t_{\text{lag}\beta}) = \log \text{DF}$$

$$t_{\text{dep1}} = \frac{\log \text{DF}}{g_\alpha} \quad \& \quad t_{\text{dep2}} = \frac{\log \text{DF}}{g_\beta} + t_{\text{lag}\beta} \tag{13}$$

For this solution to exist the following must be satisfied:

$$t_{\text{lag}\beta} < (t_{\text{dep2}} - t_{\text{dep1}}) < t_{\text{lag}\alpha}$$

$$t_{\text{lag}\beta} < \left(\frac{\log \text{DF}}{g_\beta} + t_{\text{lag}\beta} - \frac{\log \text{DF}}{g_\alpha}\right) < t_{\text{lag}\alpha}$$

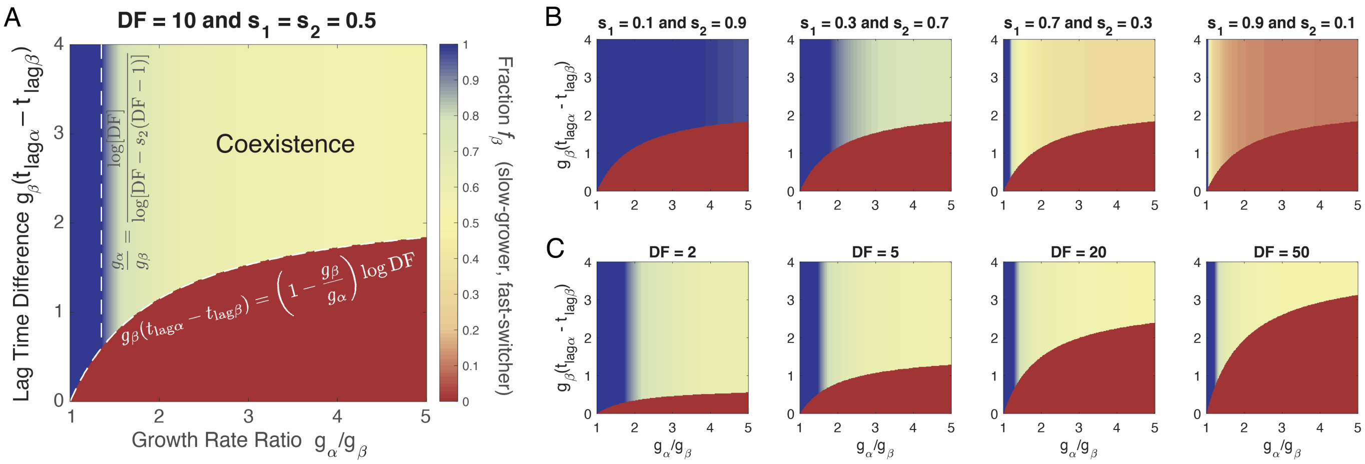

Appendix Figure S14: Simplest diauxie model supports two-species coexistence over a broad parameter range. (A) Species fractions plotted as a function of growth rate ratio and lag time difference for dilution factor DF = 10 and a balanced resource supply $s_1 = s_2$. The fast-grower α has an increasing growth advantage to the right while the fast-switcher β has an increasing lag time advantage upwards. The region of the fast-grower α excluding the slow-grower β is shown in red, and the region of the fast-switcher β excluding the slow-switcher α is shown in blue. The boundaries between coexistence and exclusion correspond to equations (14) and (16). (B) Varying the relative supplies of R_1 and R_2. (C) Varying the dilution factor DF. Same colormap applies to all subplots.

$$\frac{t_{\text{lag}\alpha} - t_{\text{lag}\beta}}{g_\alpha - g_\beta} > \frac{\log \text{DF}}{g_\alpha g_\beta} \tag{14}$$

Equation (14) is a statement that in order for the species to coexist, the difference in lag times relative to the difference in growth rates must be sufficiently large (or else both species would finish their lags at the fixed point). This is a necessary but not sufficient condition; the rest is below.

Equation (13) defines the unique pair of resource depletion times that can correspond to a fixed point. It can be further shown that only a single population composition can produce these resource depletion times by looking at the condition that the species with the shorter lag time (assumed to be β) exactly finishes the supply of the second resource with the necessary timings:

$$n_\beta(t_{\text{dep2}}) = n_\beta(t_{\text{dep1}} + t_{\text{lag}\beta}) + s_2$$

$$n_\beta(t_{\text{sat}}) = n_\beta(t_{\text{sat}}) \exp\left[-g_\beta \left(t_{\text{dep2}} - t_{\text{dep1}} - t_{\text{lag}\beta}\right)\right] + s_2$$

$$n_\beta(t_{\text{sat}}) = \frac{s_2}{1 - \text{DF}^{(g_\beta/g_\alpha - 1)}} \tag{15}$$

We can check equation (15) specifies a realizable population size (i.e. $0 < n_\beta(t_{\text{sat}}) < \frac{\text{DF}}{\text{DF}-1}$ as specified by equation (2)). First, $0 < n_\beta(t_{\text{sat}})$ is given by $g_\alpha > g_\beta$, which is already necessary to satisfy equation (14) if $t_{\text{lag}\alpha} > t_{\text{lag}\beta}$ (i.e. the slow-grower needs to the be fast-switcher in order to survive). And, second, $n_\beta(t_{\text{sat}}) < \frac{\text{DF}}{\text{DF}-1}$ can be manipulated into a requirement on the ratio between the two growth rates, g_α/g_β:

$$\frac{s_2}{1 - \text{DF}^{(g_\beta/g_\alpha - 1)}} < \frac{\text{DF}}{\text{DF} - 1}$$

$$\frac{g_\alpha}{g_\beta} > \frac{\log\left[\text{DF}\right]}{\log\left[\text{DF} - s_2\left(\text{DF} - 1\right)\right]} \tag{16}$$

If equations (14) and (16) are satisfied (enforcing that the difference in lag times and the difference in growth rates are both sufficiently large), a fixed point exists. Invasibility requirements (whether α grows

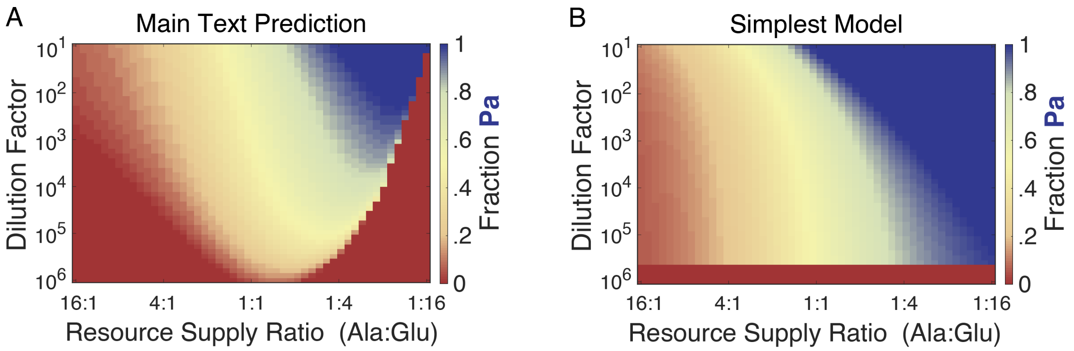

Appendix Figure S15: Many features of the Main Text model prediction are already present in the simplest diauxie model. (A) Main Text model prediction for fraction Pa as a function of dilution factor and resource supply ratio (e.g. Fig 5B) calculated at a denser array of points. (B) Predicted species fractions under the simplest diauxie model using $g_{\mathrm{Aci2}} = 0.88\mathrm{hr}^{-1}$, $g_{\mathrm{Pa}} = 0.67\mathrm{hr}^{-1}$, $t_{\mathrm{lag,Aci2}} = 5.33\mathrm{hr}^{-1}$, $t_{\mathrm{lag,Pa}} = 0.67\mathrm{hr}^{-1}$, and Pa having the same alanine preference as Aci2. There is a factor of $\frac{2}{3}$ difference between how lag times are defined in the Main Text and this section of the Appendix (by comparison of equation (8) to formulae given in the Materials and Methods and Appendix Fig S2), so this lag time values are comparable to $t_{\mathrm{lag,Aci2}} = 8\mathrm{hr}^{-1}$ and $t_{\mathrm{lag,Pa}} = 1\mathrm{hr}^{-1}$ using the Main Text quadratic growth rate recovery shape (Fig EV3B).

by at least DF when invading β's steady state, and vice versa) can be used as a coherence check and to demonstrate that this fixed point is stable.[2]

In Appendix Fig S14A, population size is plotted as a function of the growth rate ratio g_α/g_β and the normalized difference in lag times $g_\beta(t_{\mathrm{lag}\alpha} - t_{\mathrm{lag}\beta})$ in the quadrant in which α is the fast grower while β is the fast-switcher. One striking feature of this plot is the sharp boundary between a region of roughly 50:50 coexistence and a region of α excluding β. This boundary occurs at $g_\beta(t_{\mathrm{lag}\alpha} - t_{\mathrm{lag}\beta}) = \left(1 - \frac{g_\beta}{g_\alpha}\right) \log \mathrm{DF}$, which is a rearrangement of equation (14), the condition for whether or not β can finish R_2 before α finishes its lag. If α cannot finish its lag then β consumes all of s_2 and must have a population fraction of at least $s_2/(s_1 + s_2)$. If α does finish its lag then it excludes β. This creates a sharp boundary.

The most relevant feature of the Appendix Fig S14A is, however, the broad and unbounded region of coexistence. If the growth rate ratio and difference in lag times are large enough then the species coexist, with the fast-grower initially dominating before t_{dep1} and the fast-switcher dominating after. The breadth of this region also implies significant stability of the coexistence state against perturbations to species' growth rates and lag times. Appendix Fig S14B-C explore to the sensitivity to changes to the environmental parameters. If the resource supply consists mostly of the second resource then the fast-switcher β gains a significant advantage because t_{dep1} occurs earlier and the period of time when it dominates lengthens. Conversely, if the dilution factor is increased then the fast-grower α gains an advantage because t_{dep1} occurs later and the period of time when it dominates lengthens. Nevertheless, the region of coexistence remains large under moderate environmental perturbations.

When species have opposite preferences, coexistence occurs in an unbounded region of large lag times but does not require any difference in lag times. If species have opposite preferences, only one species (whichever finishes its preferred resource first) experiences its lag. If this species finishes its lag then a fixed point is not possible for the same reason a fixed point was not possible with both species finishing their diauxic lags in the previous model. Specifically, assuming α prefers R_1 and β prefers R_2 and

[2] Not provided here for the sake of brevity, so left as an exercise to the reader or available in the Supplementary Material for the 2021 bioRxiv version of this paper (Bloxham, Lee, Gore "Diauxic Lags Explain Coexistence" BioRxiv 2021.).

that α is the species that experienced and finished its lag, a fixed point requires

$$g_\alpha \left(t_{\mathrm{dep2}} - t_{\mathrm{lag}\alpha}\right) = g_\beta t_{\mathrm{dep2}} = \log \mathrm{DF} \ , \tag{17}$$

which cannot be solved for any value of t_{dep2}.

Working with the knowledge that the first species to finish its resource cannot finish its diauxic lag at any fixed point and following analysis similar to the case of both species having the same preference, it can be shown that coexistence occurs iff

$$t_{\mathrm{lag}\alpha} > \frac{g_\alpha - g_\beta}{g_\alpha g_\beta} \log \mathrm{DF} \qquad \& \qquad t_{\mathrm{lag}\beta} > \frac{g_\beta - g_\alpha}{g_\alpha g_\beta} \log \mathrm{DF} \ . \tag{18}$$

Notably, equation (18) depends only on the magnitudes of the lags and not on the difference between them and is satisfied in the large lag limit. So, if species have opposite resource preferences and diauxic lags are assumed to be large, coexistence is expected. It is even possible for a slow-grower that is also the slow-switcher to survive if both species have sufficiently long lag times.

2.3 Exponential growth with initial lags

With all yields equal, initial lags do not allow for coexistence. As previously noted, with all yields equal the two forms of daily dilution are equivalent. Therefore, at a fixed point equation (12) still holds and two species can only coexist if

$$g_\alpha(t_{\mathrm{sat}} - t_{\mathrm{lag}\alpha}) = \log \mathrm{DF} \qquad \& \qquad g_\beta(t_{\mathrm{sat}} - t_{\mathrm{lag}\beta}) = \log \mathrm{DF} \ , \tag{19}$$

which, with a fixed value of DF, cannot be solved for any value of t_{sat}.

This is an important point because it is a direct comparison between initial and diauxic lags that concludes that diauxic lags have a robust ability to produce coexistence in the simplest case while initial cannot produce coexistence. This is illustrated in Appendix Fig S16.

Initial-lag coexistence requires disequal yields and dilutions to a total population size and occurs only in a narrow parameter region. Initial lags have been shown to be a source of stable coexistence[3,4], which is in seeming contradiction to our previous conclusion. The difference between these analyses is the form of daily dilution that is used. With a dilution by a constant factor, initial lags and exponential growth on a single resource cannot produce stable coexistence. But, with a dilution to a constant population size and the slow-grower/fast-switcher having a higher yield than the fast-grower/slow-switcher, initial lags can lead to coexistence.

The following repeats analysis in Manhart 2018a,b but with a choice of variables that matches the previous diauxic lag calculations.

[3]Manhart, Adkar, and Shakhnovich "Trade-offs between microbial growth phases" *Proc Royal Soc B.* 2018.
[4]Manhart and Shakhnovich "Growth tradeoffs produce complex microbial communities" *Nat Commun.* 2018.

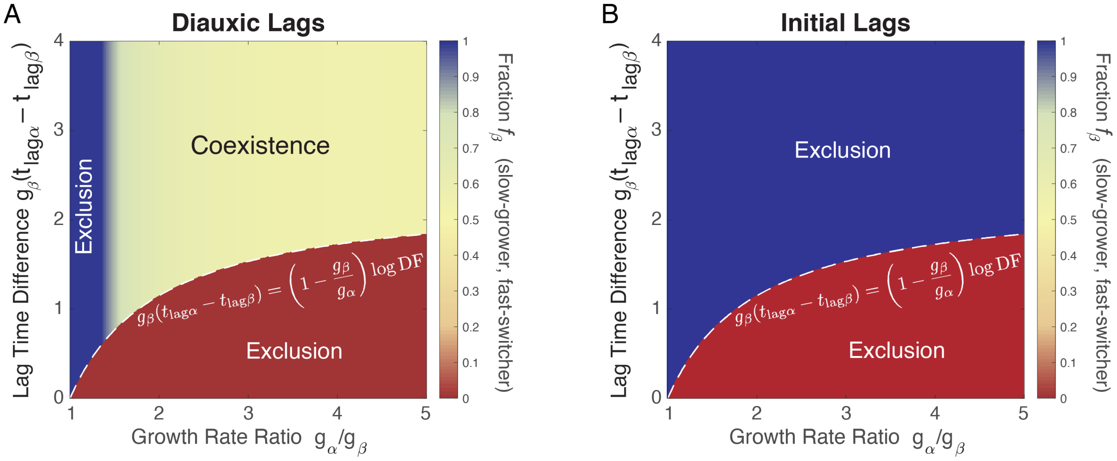

Appendix Figure S16: In simplest model, diauxic lags allow for coexistence whereas initial lags do not. (A) Same phase space as in Appendix Fig S14A. (B) Same phase space when species have initial lags and dilutions are still constant-factor dilutions by DF = 10 (same as A). The broad region of coexistence has been replaced by a region of the fast-switcher excluding the slow-switcher.

With DF replaced by $\mathrm{DF}_{\mathrm{eff}}$, where $\mathrm{DF}_{\mathrm{eff}}$ is now an environmental variable that can be affected by the population composition, equation (19) can now be solved by

$$t_{\mathrm{sat}} = \frac{g_\alpha t_{\mathrm{lag}\alpha} - g_\beta t_{\mathrm{lag}\beta}}{g_\alpha - g_\beta} \qquad \& \qquad \log \mathrm{DF}_{\mathrm{eff}} = \frac{g_\alpha g_\beta (t_{\mathrm{lag}\alpha} - t_{\mathrm{lag}\beta})}{g_\alpha - g_\beta} \ , \tag{20}$$

which by linearity of equation (19) is the unique solution.

Population sizes can be calculated from knowing the total population at the start of the start of the day and that the resource supply is totally consumed after both species have grown by a factor of $\mathrm{DF}_{\mathrm{eff}}$:

$$n_\alpha(0) + n_\beta(0) = n_0 \qquad \& \qquad \frac{n_\alpha(0)(\mathrm{DF}_{\mathrm{eff}} - 1)}{y_\alpha} + \frac{n_\alpha(0)(\mathrm{DF}_{\mathrm{eff}} - 1)}{y_\alpha} = s$$

$$n_\alpha(0) = \frac{y_\alpha n_0}{y_\alpha - y_\beta} - \frac{y_\alpha y_\beta s}{(\mathrm{DF}_{\mathrm{eff}} - 1)(y_\alpha - y_\beta)} \qquad \& \qquad n_\beta(0) = \frac{y_\beta n_0}{y_\beta - y_\alpha} + \frac{y_\alpha y_\beta s}{(\mathrm{DF}_{\mathrm{eff}} - 1)(y_\alpha - y_\beta)} \ , \tag{21}$$

which is again a unique solution by linearity.

To find constraints on when this fixed point can exist, we solve for the condition $n_\mu(0) > 0$:

$$\frac{y_\alpha n_0}{y_\alpha - y_\beta} - \frac{y_\alpha y_\beta s}{(\mathrm{DF}_{\mathrm{eff}} - 1)(y_\alpha - y_\beta)} > 0 \qquad \& \qquad \frac{y_\beta n_0}{y_\beta - y_\alpha} + \frac{y_\alpha y_\beta s}{(\mathrm{DF}_{\mathrm{eff}} - 1)(y_\alpha - y_\beta)} > 0$$

After some simplification and handling of which yield is larger, we find that in order for the fixed point to exist, it must be the case that

$$\frac{y_{\mathrm{min}} s}{n_0} + 1 < \log \mathrm{DF}_{\mathrm{eff}} < \frac{y_{\mathrm{max}} s}{n_0} + 1 \ , \tag{22}$$

where y_{min} is the smaller of the two yields (i.e. $\min(y_\alpha, y_\beta)$) and y_{max} the larger.

Equation (23) is also equivalent to

$$\mathrm{DF}_{\mathrm{eff,min}} < \mathrm{DF}_{\mathrm{eff}} < \mathrm{DF}_{\mathrm{eff,max}}, \tag{23}$$

where $\mathrm{DF}_{\mathrm{eff,min}}$ is the effective dilution factor for a monoculture of the species with the lowest yield and $\mathrm{DF}_{\mathrm{eff,max}}$ is the effective dilution factor for a monoculture of the species with the largest yield.

Plugging equation (20) into equation (23) yields

$$\log\left(\frac{y_{\min}}{n_0/s}+1\right) < \frac{t_{\mathrm{lag}\alpha}-t_{\mathrm{lag}\beta}}{(g_\alpha-g_\beta)/g_\alpha g_\beta} < \log\left(\frac{y_{\max}}{n_0/s}+1\right). \tag{24}$$

If equation (24) is satisfied a fixed point will exist. Manhart 2018a,b previously showed that the fixed point will be stable and the species will coexist if and only if the species with the higher yield is the slow-grower/fast-switcher. (Otherwise the fixed point will be the separatix of a bistability.)

Important to note in equation (24) is that while a sufficiently large difference in lag times $|t_{\mathrm{lag}\alpha}-t_{\mathrm{lag}\beta}|$ is necessary for coexistence, if the difference becomes too large the species will no longer coexist. This is in contrast to the previously explored case of diauxic lags, in which a larger difference in lag times can only ever benefit coexistence. In this way, initial lags as a source of coexistence, while producing genuinely stable (non-neutral) coexistence, only do so in a narrow parameter region and the resulting state will likely be more fragile against environmental perturbations, compared diauxic lags as a source of coexistence occurring in a larger, more robust parameter region. The width of the region of coexistence is also directly limited by the ratio between the yields $y_{\max}/y_{\min}$ because

$$\log\left(\frac{y_{\max}}{n_0/s}+1\right)-\log\left(\frac{y_{\min}}{n_0/s}+1\right) < \log\left(\frac{y_{\max}}{y_{\min}}\right) . \tag{25}$$

Yields are an essential part of the tradeoff that stabilizes coexistence in this model. That one species is the fast-grower and the fast-starter means the curves $\Delta\log n_\mu = g_\mu(t_{\mathrm{sat}}-t_{\mathrm{lag}\mu})$ cross at some point $(t^*_{\mathrm{sat}}, \mathrm{DF}^*_{\mathrm{eff}})$. But for this point to correspond to stable coexistence, there needs to be a feedback mechanism between the population sizes and the environmental parameters t_{sat} and $\mathrm{DF}_{\mathrm{eff}}$.

Assuming species α has the faster growth rate, longer lag time, and lower yield compared to β (i.e. $g_\alpha > g_\beta$, $t_{\mathrm{lag}\alpha} > t_{\mathrm{lag}\beta}$, and $y_\alpha < y_\beta$): If α's population size is above its equilibrium value, then because α has the smaller yield the overall population size must be smaller. A smaller total population size means the effective dilution $\mathrm{DF}_{\mathrm{eff}}$ is smaller than at equilibrium. A smaller $\mathrm{DF}_{\mathrm{eff}}$ means the population needs to grow for less time to return to saturation (i.e. t_{sat} occurs earlier than at equilibrium). If $t_{\mathrm{sat}} < t^*_{\mathrm{sat}}$ then $\Delta\log n_\alpha$ and $\Delta\log n_\beta$ never cross. Because α is the slow-starter and fast-grower, α's population size declines relative to β if $t_{\mathrm{sat}} < t^*_{\mathrm{sat}}$. This completes a negative feedback loop that stabilizes the coexistence state. The relative growth rates, lag times, and yields and the species' ability to affect both t_{sat} and $\mathrm{DF}_{\mathrm{eff}}$ are all essential parts of the negative frequency-dependent interaction between α and β and the fixed point's stability.

Equations (20) and (21) can be combined to calculate species fractions

$$f_\beta = \frac{y_\beta}{y_\alpha - y_\beta}\left(\frac{y_\alpha(s/n_0)}{\exp\left[\frac{g_\alpha g_\beta(t_{\mathrm{lag}\alpha}-t_{\mathrm{lag}\beta})}{g_\alpha-g_\beta}\right]-1}-1\right) , \tag{26}$$

which are plotted as a function of the various parameters in Appendix Fig S17.

For further discussion of coexistence within this initial lag model see Manhart 2018a,b.

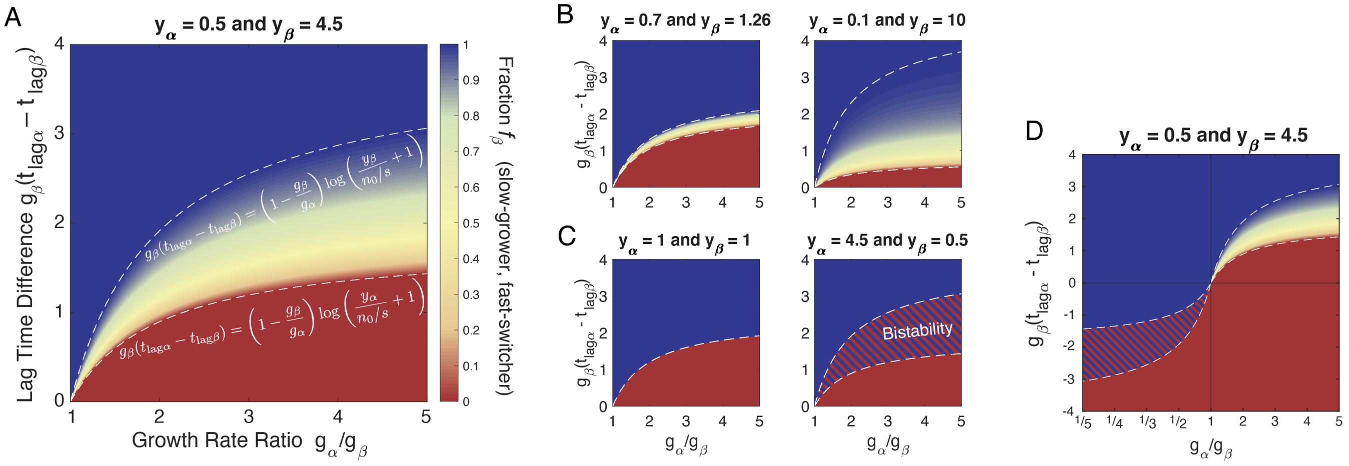

Appendix Figure S17: Initial lags produce coexistence but only in a much narrower parameter region. (A) Species fractions plotted as a function of growth rate ratio and lag time difference for $n_0 = 0.1$ and $s = 1$. The values $y_\alpha = 0.5$ and $y_\beta = 4.5$ are chosen such that if $f_\beta = 0.5$ then $\mathrm{DF}_{\mathrm{eff}} = 10$ (for comparability to Appendix Fig S14A,B and S16). It is noted that in contrast to coexistence produced by diauxic lags, there now exists an upper bound (in terms of $g_\beta(t_{\mathrm{lag}\alpha} - t_{\mathrm{lag}\beta})$) on the region in which coexistence can be achieved. (B) As yields are made more similar the region of coexistence shrinks. Both panels continue to use $n_0 = 0.1$ and the values $y_\alpha = 0.7$ and $y_\beta = 4.5$ are chosen to again have $\mathrm{DF}_{\mathrm{eff}} = 10$ when $f_\beta = 0.5$. The lower bound varies with $\frac{y_\alpha}{n_0/s}$ and the upper bound varies with $\frac{y_\beta}{n_0/s}$, so if n_0 or s were changed both bounds would move up to down and any coexistence point would quickly end up in one of the exclusion regions. (C) If the yields are equal there is no region of coexistence, and if the yields are flipped the region of coexistence becomes a region of bistability in which one species will exclude the other depending on initial fractions. (D) Across all four quadrants (i.e. allowing lag and growth rate ordering to switch), only a small region of one quadrant allows for coexistence. This highlights how the necessary tradeoff must involve growth rates, lag times, *and* yields in order for coexistence to be realized. This is in contrast to diauxic lags as a source of coexistence, with which yields are not a necessary part of the tradeoff and both the top right and the lower left quadrants would include large regions of coexistence.

2.4 Three species on two resources with either initial or diauxic lags

Up to three species can coexist on two resources with diauxic lags and dilutions to a total population size. Because the dilution implementation made a difference as to whether one or two species could survive with initial lags, it is reasonable to ask whether it will make the difference between two or three species surviving with diauxic lags. The answer turns out to be yes: three species can survive on two resources with diauxic lags (and only a single growth rate for each species) if dilutions are implemented as being to a constant population size.

The first question to answer is how many species finish their diauxic lag at a three-species fixed point. At least one must because otherwise the second resource wouldn't get eaten. If only one finishes, we end up with an equation such as $g_\alpha t_{\mathrm{dep1}} = g_\beta t_{\mathrm{dep1}}$, which can't be solved. And if all species finish their diauxic lags, we end up with the equation $g_\alpha(t_{\mathrm{dep2}} - t_{\mathrm{lag}\alpha}) = g_\beta(t_{\mathrm{dep2}} - t_{\mathrm{lag}\beta}) = g_\gamma(t_{\mathrm{dep2}} - t_{\mathrm{lag}\gamma})$, which also can't be solved. Therefore, we know that if there is a fixed point with three species surviving, exactly two of those species (which we'll assume are β and γ) must finish their diauxic lags, while the third (α) does not.

For simplicity, we will assume that each species has only a single yield (i.e. $y_{\mu 1} = y_{\mu 2}$).

Proceeding with fixed point analysis, equation (12) requires

$$\Delta \log n_\alpha = \Delta \log n_\beta = \Delta \log n_\gamma = \log \mathrm{DF}_{\mathrm{eff}} \ .$$

Beginning with $\Delta \log n_\beta = \Delta \log n_\gamma$ and then moving towards $\log \mathrm{DF}_{\mathrm{eff}}$ and $\Delta \log n_\alpha$:

$$g_\beta(t_{\mathrm{dep2}} - t_{\mathrm{lag}\beta}) = g_\gamma(t_{\mathrm{dep2}} - t_{\mathrm{lag}\gamma})$$

$$t_{\mathrm{dep2}} = \frac{g_\beta t_{\mathrm{lag}\beta} - g_\gamma t_{\mathrm{lag}\gamma}}{g_\beta - g_\gamma} \tag{27}$$

$$\log \mathrm{DF}_{\mathrm{eff}} = \frac{g_\beta g_\gamma (t_{\mathrm{lag}\beta} - t_{\mathrm{lag}\gamma})}{g_\beta - g_\gamma} \tag{28}$$

$$t_{\mathrm{dep1}} = \frac{g_\beta g_\gamma (t_{\mathrm{lag}\beta} - t_{\mathrm{lag}\gamma})}{g_\alpha (g_\beta - g_\gamma)} \tag{29}$$

A quick check can show that $0 < t_{\mathrm{dep1}} < t_{\mathrm{dep2}}$ is true if $g_\alpha > g_\beta$ and $g_\alpha > g_\gamma$ and either $g_\beta > g_\gamma$ and $t_{\mathrm{lag}\beta} > t_{\mathrm{lag}\gamma}$ or $g_\gamma > g_\beta$ and $t_{\mathrm{lag}\gamma} > t_{\mathrm{lag}\beta}$, which were all already logistically necessary conditions. Important to note is that these are the unique $\{t_{\mathrm{dep1}}, t_{\mathrm{dep2}}, \mathrm{DF}_{\mathrm{eff}}\}$ that allow all three species to grow by the same factor as each other and on each day.

We can now set up a system of three linear equations for $\{n_\alpha(0), n_\beta(0), n_\gamma(0)\}$: one from initial population sizes summing to n_0, one from exact consumption of R_1 at t_{dep1}, and one from exact consumption of R_2 at t_{dep2}.

$$\begin{pmatrix} 1 & 1 & 1 \\ \frac{e^{g_\alpha t_{\mathrm{dep1}}}-1}{y_\alpha} & \frac{e^{g_\beta t_{\mathrm{dep1}}}-1}{y_\beta} & \frac{e^{g_\gamma t_{\mathrm{dep1}}}-1}{y_\gamma} \\ 0 & \frac{e^{g_\beta (t_{\mathrm{dep2}} - t_{\mathrm{lag}\beta})} - e^{g_\beta t_{\mathrm{dep1}}}}{y_\beta} & \frac{e^{g_\gamma (t_{\mathrm{dep2}} - t_{\mathrm{lag}\gamma})} - e^{g_\gamma t_{\mathrm{dep1}}}}{y_\gamma} \end{pmatrix} \cdot \begin{pmatrix} n_\alpha(0) \\ n_\beta(0) \\ n_\gamma(0) \end{pmatrix} = \begin{pmatrix} n_0 \\ s_1 \\ s_2 \end{pmatrix} \tag{30}$$

The analytic solution to equation (30) is too messy to be printed here, but the linear nature of the equation does mean any solution for $\{n_\mu(0)\}$ will be unique. Furthermore, there are parameter regions in which solutions with $n_\mu(0) > 0$ exist. For example, when the parameters

$$\begin{array}{llll} g_\alpha = 0.3\ \mathrm{hr}^{-1} & y_\alpha = 0.15 & t_{\mathrm{lag}\alpha} = 13\ \mathrm{hr} & n_0 = 0.075 \\ g_\beta = 0.14\ \mathrm{hr}^{-1} & y_\beta = 0.2 & t_{\mathrm{lag}\beta} = 5\ \mathrm{hr} & s_1 = 0.5 \\ g_\gamma = 0.1\ \mathrm{hr}^{-1} & y_\gamma = 0.7 & t_{\mathrm{lag}\gamma} = 0.5\ \mathrm{hr} & s_2 = 0.5 \end{array} \tag{31}$$

are used, equation (30) is solved by

$$\begin{aligned} n_\alpha(0) &= 0.013 \\ n_\beta(0) &= 0.024 \\ n_\gamma(0) &= 0.038 \quad . \end{aligned}$$

Analytically determining the stability of this fixed point would be cumbersome, so we instead simulate the community and plot the results. The parameters presented as equation (31) produce a stable fixed point that is a universal attractor. This demonstrates that three species can stably coexist on two resources with diauxic lags and dilutions to a specific population size.

This is a rich situation because there is simultaneously a growth rate − lag time tradeoff and a growth rate − yield tradeoff. A dedicated study of this model may be warranted by future research. The basic interactions are:

- Species β and γ both finish their lags, so they are unaffected by changes to t_{dep1}, each growing by $\Delta \log n_\mu = g_\mu(t_{\mathrm{dep2}} - t_{\mathrm{lag}\mu})$. Of the two, β is the fast-grower so it benefits from a later t_{dep2}.

- Species β has a smaller yield than γ, so increasing β's population fraction relative to γ will result in smaller end-of-day populations and smaller effective dilution factors $\mathrm{DF}_{\mathrm{eff}} = (n_\alpha + n_\beta + n_\gamma)/n_0$.
- A smaller effective dilution will result in earlier resource depletion times, which increase γ's growth relative to β. This establishes a negative frequency-dependent selection between β and γ.
- Meanwhile, α is the fast-grower and, near the fixed point, does not finish its lag, so it's growth is entirely determined by t_{dep1}. Increasing α's population fraction will tend to move t_{dep1} earlier because it's the species that consumes R_1 the fastest. An earlier t_{dep1} decreases α's growth, so a negative frequency-dependent selection between α and the other two species is established.
- Thus, β and γ's negative frequency-dependent interaction holds t_{dep2} and $\mathrm{DF}_{\mathrm{eff}}$ at the fixed point values, while α holds t_{dep1} at its fixed point value.

These are complicated dynamics, but the key feature is that

$$\Delta \log n_\alpha(t_{\mathrm{dep1}}, t_{\mathrm{dep2}}) = \Delta \log n_\beta(t_{\mathrm{dep1}}, t_{\mathrm{dep2}}) = \Delta \log n_\gamma(t_{\mathrm{dep1}}, t_{\mathrm{dep2}}) \tag{32}$$

can indeed by solved for t_{dep1} and t_{dep2} because there is no fixed value of $\log \mathrm{DF}$ such that we must have $\Delta \log n = \mathrm{DF}$ at any fixed point. Instead, there is some $\Delta \log n$ value that all species can obtain at the same time with the right values t_{dep1} and t_{dep2} (i.e. $\Delta \log n_\mu(t_{\mathrm{dep1}}, t_{\mathrm{dep2}})$ all intersect somewhere) and the population sizes then adjust (through a dilution factor-mediated negative frequency-dependent interaction) until $\mathrm{DF}_{\mathrm{eff}} = \Delta \log n$ (which can be obtained due to species all having different yields).

It is plausible that three species could coexist on two resources with initial lags, dilutions to a total population size, and separate growth rates for each resource. The previous results leads us to expect that three species should also be able to coexist with initial lags if t_{dep1} and t_{dep2} can both be made relevant. Without diauxic lags, we need to introduce separate second-resource growth rates for each species, such that species are now characterized by four parameters (compared to the previous three): a lag time, a yield, and two growth rates.

Analysis mirrors the previous calculations and is provided here in brief.

To find a fixed point, choose parameters, solve:

$$\begin{pmatrix} g_{\alpha 1} - g_{\alpha 2} & g_{\alpha 2} & -1 \\ g_{\beta 1} - g_{\beta 2} & g_{\beta 2} & -1 \\ g_{\gamma 1} - g_{\gamma 2} & g_{\gamma 2} & -1 \end{pmatrix} \cdot \begin{pmatrix} t_{\mathrm{dep1}} \\ t_{\mathrm{dep2}} \\ \log \mathrm{DF}_{\mathrm{eff}} \end{pmatrix} = \begin{pmatrix} g_{\alpha 1} t_{\mathrm{lag}\alpha} \\ g_{\beta 1} t_{\mathrm{lag}\beta} \\ g_{\gamma 1} t_{\mathrm{lag}\gamma} \end{pmatrix} \tag{33}$$

then solve:

$$\begin{pmatrix} 1 & 1 & 1 \\ \frac{e^{g_{\alpha 1}(t_{\mathrm{dep1}} - t_{\mathrm{lag}\alpha})} - 1}{y_\alpha} & \frac{e^{g_{\beta 1}(t_{\mathrm{dep1}} - t_{\mathrm{lag}\beta})} - 1}{y_\beta} & \frac{e^{g_{\gamma 1}(t_{\mathrm{dep1}} - t_{\mathrm{lag}\gamma})} - 1}{y_\gamma} \\ \frac{\mathrm{DF}_{\mathrm{eff}}\left(1 - e^{-g_{\alpha 2}(t_{\mathrm{dep2}} - t_{\mathrm{dep1}})}\right)}{y_\alpha} & \frac{\mathrm{DF}_{\mathrm{eff}}\left(1 - e^{-g_{\beta 2}(t_{\mathrm{dep2}} - t_{\mathrm{dep1}})}\right)}{y_\beta} & \frac{\mathrm{DF}_{\mathrm{eff}}\left(1 - e^{-g_{\gamma 2}(t_{\mathrm{dep2}} - t_{\mathrm{dep1}})}\right)}{y_\gamma} \end{pmatrix} \cdot \begin{pmatrix} n_\alpha(0) \\ n_\beta(0) \\ n_\gamma(0) \end{pmatrix} = \begin{pmatrix} n_0 \\ s_1 \\ s_2 \end{pmatrix} \tag{34}$$

and then confirm $t_{\mathrm{lag}\mu} < t_{\mathrm{dep1}} < t_{\mathrm{dep2}}$ and $\forall_\mu\ n_\mu(0) > 0$.

While we were able to identify fixed points, none of them were stable. It is possible that a stable fixed point would require one or two of the species to not finish its initial lag until after t_{dep1} or that we simply failed to identify one during our limited search. We suspect stable fixed points should be possible, but this is left for future work to establish.

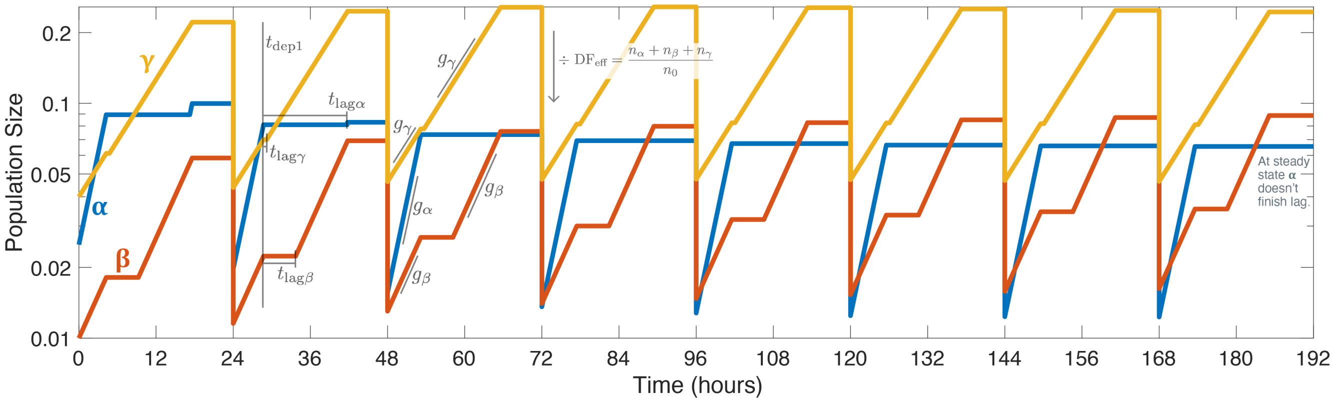

Appendix Figure S18: Three species survive on two resources with diauxic lags and a dilution to a specific population size. Parameters are those labeled as equation (31). The system is initiated at $\{n_\alpha, n_\beta, n_\gamma\} = \{0.025, 0.01, 0.04\}$ and run for 1000 days (of which the first eight are shown) to confirm stability of the fixed point. Species begin each day growing exponentially until the first resource is depleted (when $\frac{\Delta n_\alpha}{y_\alpha} + \frac{\Delta n_\beta}{y_\beta} + \frac{\Delta n_\gamma}{y_\gamma} = s_1$). Species then experience diauxic lags before resuming growth at their original growth rates. After saturation, population sizes are divided by a population-size dependent dilution factor $\mathrm{DF_{eff}}$ to reset to $n_\alpha + n_\beta + n_\gamma = n_0$.

Assuming three species can coexist in this scenario, it is worth noting that this requires each species to be characterized by four parameters (instead of the three parameters per species for the case of three species surviving as the result of diauxic lags) and for a first-resource vs second-resource growth rate tradeoff to become involved. This continues the pattern of diauxic lags consistently being slightly ahead of initial lags in terms of ability to support coexistence. The strength of diauxic lags in supporting coexistence comes from their division of the growth phase into two separate subphases with their own distinct dynamics without the need for multiple growth rates and the associated increase in tradeoff complexity.

As a final note, some form of lag is necessary to have three species surviving on two resources even when they have separate R_1 and R_2 growth rates. With no lags we would have:

$$\forall \mu \quad g_{\mu 1} t_{\mathrm{dep1}} + g_{\mu 2}(t_{\mathrm{dep2}} - t_{\mathrm{dep1}}) = \log \mathrm{DF_{eff}}$$

or

$$\begin{pmatrix} g_{\alpha 1} - g_{\alpha 2} & g_{\alpha 2} & -1 \\ g_{\beta 1} - g_{\beta 2} & g_{\beta 2} & -1 \\ g_{\gamma 1} - g_{\gamma 2} & g_{\gamma 2} & -1 \end{pmatrix} \cdot \begin{pmatrix} t_{\mathrm{dep1}} \\ t_{\mathrm{dep2}} \\ \log \mathrm{DF_{eff}} \end{pmatrix} = \begin{pmatrix} 0 \\ 0 \\ 0 \end{pmatrix} \tag{35}$$

Because the matrix will have a nonzero determinant without fine tuning the growth rates, equation (35) is only solved by $t_{\mathrm{dep1}} = t_{\mathrm{dep2}} = \log \mathrm{DF_{eff}} = 0$, which is a nonphysical solution. More intuitively, the fixed point is impossible because $\Delta \log n_\mu = (g_{\mu 1} = g_{\mu 2}) t_{\mathrm{dep1}} + (g_{\mu 2}) t_{\mathrm{dep2}}$ are three planes that intersect at the origin and therefore nowhere else (whereas with lags the planes are offset from the origin and intersect somewhere else).

2.5 Likelihood of random species coexisting with either initial or diauxic lags

With separate growth rates for each resource and constant-factor dilutions, at most two species can survive with diauxic lags, initial lags, or no lags, but the frequency of coexistence may still vary. Having taken an analytic approach to asking whether coexistence is possible under various

scenarios with initial or diauxic lags, we now approach our central question from a different angle and ask how frequently coexistence is expected to be observed. To set up a fair comparison we want a case in which both initial and diauxic lags support the same maximum number of surviving species.

We choose the case of two species growing on two resources with separate growth rates for each resource and a constant factor dilution (with all yields set to 1). There are two environmental parameters that feedback and affect the species: t_{dep1} and t_{dep2}. In the absence of lags, it is well-established that two species can survive on two resources if one is the R_1 fast-grower and the other is the R_2 slow-grower (with appropriate bounds on the necessary values of $g_{\mu i}$ relative to the dilution factor and resource supply). Adding in lags does not, however, increase the maximum number of surviving species because we still need to solve

$$\Delta \log n_\mu(t_{\text{dep1}}, t_{\text{dep2}}) = \log \text{DF} \tag{36}$$

for each species. With only two free variables we can solve equation (36) for at most two species.

For our comparison of initial and diauxic lags, we can sample species from some reasonable distributions and ask whether they coexist (i) with initial lags, (ii) with diauxic lags, and (iii) without any lags. So, although we know coexistence is possible, we are now asking how frequently it occurs.

Invasibility can be used as an approximation of whether species coexist. Fixed point analysis has been working great so far but would now become more tedious and computationally slower. So we instead turn to invasibility criteria. If species α and species β can each invade each other's steady-state then we conclude they coexist. We have a continuous mapping of population fractions on one day to population fractions on the next, so if there is a single fixed point then invasibility criteria are sufficient to determine the qualitative competitive outcome. If each species can invade the other, neither can be driven extinct, and both must survive.[5]

We begin with **diauxic lags** and consider the invasibility of slow-switcher α by fast-switcher β:

The environmental parameters for slow-switcher α's steady-state can be calculated as the time it would take α to finish R_1 then R_2 on its own (remembering equation (2) for the day-to-day carryover correciton):

$$n_\alpha(t_{\text{dep1}|\alpha}) - n_\alpha(0) = s_1 \qquad \& \qquad n_\alpha(t_{\text{dep2}|\alpha}) - n_\alpha(t_{\text{dep1}|\alpha}) = 1 - s_1$$

$$\frac{1}{\text{DF}-1}\exp\left(g_{\alpha 1} t_{\text{dep1}|\alpha}\right) - \frac{1}{\text{DF}-1} = s_1 \qquad \& \qquad \frac{\text{DF}}{\text{DF}-1} - \frac{\text{DF}}{\text{DF}-1}\exp\left(-g_{\alpha 2} t_{\text{dep2}|\alpha}\right) = 1 - s_1$$

$$t_{\text{dep1}|\alpha} = \frac{1}{g_{\alpha 1}}\log\left(s_1(\text{DF}-1)+1\right) \qquad \& \qquad t_{\text{dep2}|\alpha} - t_{\text{dep1}|\alpha} = t_{\text{lag}\alpha} + \frac{1}{g_{\alpha 2}}\log\left(\frac{\text{DF}}{s_1(\text{DF}-1)+1}\right) \tag{37}$$

Knowing α's steady-state, we can determine if β can invade by asking whether β grows by at least a factor of DF on each cycle – i.e. if $g_{\beta 1} t_{\text{dep1}|\alpha} + g_{\beta 1}(t_{\text{dep2}|\alpha} - t_{\text{dep1}|\alpha}) > \log \text{DF}$.

[5] We cannot, however, rule out the possibility of two fixed points. Assuming α is the slow-switcher: With initial lags there may be both a fixed point in which α finishes its lag in time to eat some of R_1 and a separate fixed point in which α does not finish its lag in time and only eats R_2. Likewise, with diauxic lags there may be both a fixed point in which α finishes its lag in time to eat some of R_2 and a separate fixed point in which α does not finish its lag in time and only eats R_1. In both cases, each fixed point would correspond to different values of t_{dep1} and t_{dep2} and, in turn, different population sizes. If two fixed points do exist, the qualitative competitive outcome will be a bistability between a coexistence state and a competitive exclusion. Ideally we would detect these cases and use the width of the basins of attractions in calculating the likelihood of coexistence. But, in the interest of simplicity, we will assume the presence of both fixed points is rare (later backed up by a demonstration that an unstable fixed point is already rare) and use invasibility as an analytically simple and computationally efficient approximation for whether the species coexist.

So, with diauxic lags, fast-switcher β can invade slow-switcher α iff

$$\frac{g_{\beta 1}}{g_{\alpha 1}} \log\left(s_1(\mathrm{DF}-1)+1\right) + \frac{g_{\beta 2}}{g_{\alpha 2}} \log\left(\frac{\mathrm{DF}}{s_1(\mathrm{DF}-1)+1}\right) + g_{\beta 2} t_{\mathrm{lag}\alpha} > \log \mathrm{DF} \ . \tag{38}$$

Similarly, fast-switcher β's steady-state is

$$t_{\mathrm{dep1}|\beta} = \frac{1}{g_{\beta 1}} \log\left(s_1(\mathrm{DF}-1)+1\right) \qquad \& \qquad t_{\mathrm{dep2}|\beta} - t_{\mathrm{dep1}|\beta} = \frac{1}{g_{\beta 2}} \log\left(\frac{\mathrm{DF}}{s_1(\mathrm{DF}-1)+1}\right), \tag{39}$$

and so, with diauxic lags, α can invade β iff

$$\frac{g_{\alpha 1}}{g_{\beta 1}} \log\left(s_1(\mathrm{DF}-1)+1\right) + \max\left[0,\ \frac{g_{\alpha 2}}{g_{\beta 2}} \log\left(\frac{\mathrm{DF}}{s_1(\mathrm{DF}-1)+1}\right) - g_{\alpha 2} t_{\mathrm{lag}\alpha}\right] > \log \mathrm{DF} \ , \tag{40}$$

where the $\max[0,\ \ldots]$ is necessary to account for the possibility that $t_{\mathrm{lag}\alpha} > t_{\mathrm{dep2}|\beta} - t_{\mathrm{dep1}|\beta}$ and prevent the second term from becoming negative.

With **initial lags** instead of diauxic, all that changes about α's steady state is that $t_{\mathrm{dep1}|\alpha}$ is now lengthened by $t_{\mathrm{lag}\alpha}$ instead of $t_{\mathrm{dep2}|\alpha} - t_{\mathrm{dep1}|\alpha}$ being lengthen:

$$t_{\mathrm{dep1}|\alpha} = t_{\mathrm{lag}\alpha} + \frac{1}{g_{\alpha 1}} \log\left(s_1(\mathrm{DF}-1)+1\right) \qquad \& \qquad t_{\mathrm{dep2}|\alpha} - t_{\mathrm{dep1}|\alpha} = \frac{1}{g_{\alpha 2}} \log\left(\frac{\mathrm{DF}}{s_1(\mathrm{DF}-1)+1}\right) \tag{41}$$

So, with initial lags, fast-switcher β can invade slow-switcher α iff

$$\frac{g_{\beta 1}}{g_{\alpha 1}} \log\left(s_1(\mathrm{DF}-1)+1\right) + g_{\beta 1} t_{\mathrm{lag}\alpha} + \frac{g_{\beta 2}}{g_{\alpha 2}} \log\left(\frac{\mathrm{DF}}{s_1(\mathrm{DF}-1)+1}\right) > \log \mathrm{DF} \ . \tag{42}$$

Because β is assumed to have no lag, its steady state is still

$$t_{\mathrm{dep1}|\beta} = \frac{1}{g_{\beta 1}} \log\left(s_1(\mathrm{DF}-1)+1\right) \qquad \& \qquad t_{\mathrm{dep2}|\beta} - t_{\mathrm{dep1}|\beta} = \frac{1}{g_{\beta 2}} \log\left(\frac{\mathrm{DF}}{s_1(\mathrm{DF}-1)+1}\right). \tag{43}$$

Determining whether α can invade β is, however, a slightly more complicated expression than before. This is because α may or may not finish its lag before R_1 is depleted. Specifically:

With initial lags slow-switcher α can invade fast-switcher β iff

$$\begin{aligned} &\max\left[0,\ \frac{g_{\alpha 1}}{g_{\beta 1}} \log\left(s_1(\mathrm{DF}-1)+1\right) - g_{\alpha 1} t_{\mathrm{lag}\alpha}\right] + \ldots \\ g_{\alpha 2}&\left(\frac{1}{g_{\beta 2}} \log\left(\frac{\mathrm{DF}}{s_1(\mathrm{DF}-1)+1}\right) - \max\left[0,\ t_{\mathrm{lag}\alpha} - \frac{\log\left(s_1(\mathrm{DF}-1)+1\right)}{g_{\beta 1}}\right]\right) > \log \mathrm{DF} \ . \end{aligned} \tag{44}$$

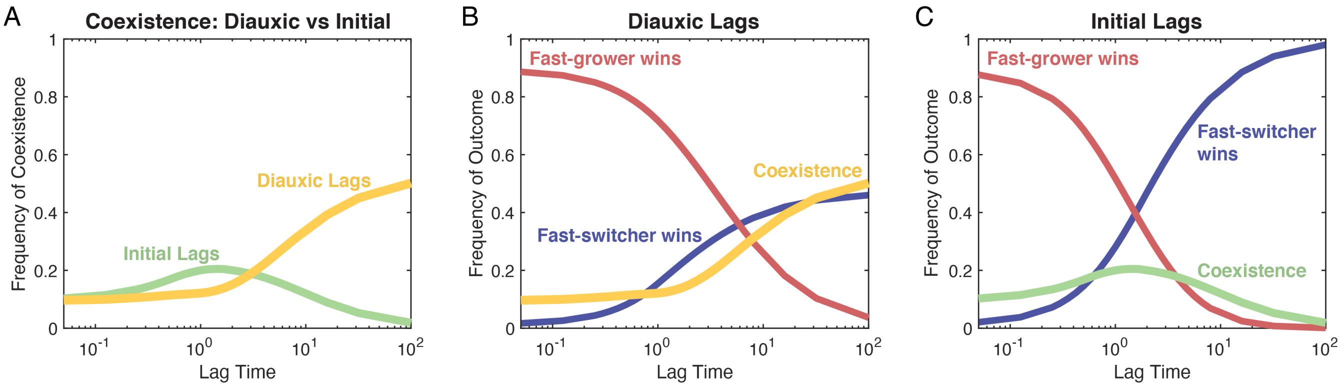

Appendix Figure S19: Initial lags benefit coexistence at small-to-intermediate lag time values, while diauxic lags benefit coexistence at large lag time values. (A) Species' growth rates were uniform-randomly sampled from the volume in which α is the overall fast-grower ($g_{\alpha 1}+g_{\alpha 2} > g_{\beta 1}+g_{\beta 2}$), both species grow fastest on R_1 ($g_{\mu 1} > g_{\mu 2}$), and growth rates ranged from 0 to 1 ($0 < g_{\mu i} < 1$). The qualitative competitive outcomes were determined for the case of each species eating R_1 then R_2 and α having either an initial or a diauxic lag. The frequency of coexistence is plotted as a function of α's lag time. Around $t_{\text{lag}\alpha} \approx 1$, initial lags roughly double the frequency of coexistence compared to the case of no lags. At larger $t_{\text{lag}\alpha}$, diauxic lags increasingly benefit coexistence, roughly quintupling the frequency of coexistence by $t_{\text{lag}\alpha} \approx 100$. The two curves cross at $t_{\text{lag}\alpha} \approx 3$, which is roughly the typical doubling time. (B) In the case of diauxic lags, increasing the fast-grower α's lag time benefits the fast-switcher but never by so much that the fast-switcher excluding the fast-grower becomes the overwhelmingly most likely outcome. (C) By contrast, in the case of initial lags, increasing the lag time eventually leads to the fast-switcher excluding the slow-switcher the nearly universal outcome and driving the likelihood of coexistence towards zero. (For brevity, 'wins' is used in place of 'excludes the other species' in the figure labels.)

With random species from reasonable distributions, coexistence is more likely if species have diauxic lags than if they have initial. With the invasibility criteria worked out, we now choose distributions to sample species from. We assume growth rates vary from 0 to 1 (with an implicit unit of hr^{-1} in mind) and lag times vary from 0 to 12 (again with a unit of hours in mind and considering the lag times seen in this paper's experimental sections as representative). We assume that species grow fastest on their preferred resource (i.e. $g_{\mu 1} > g_{\mu 2}$) and assume the fast-grower is the slow-switcher. With two growth rates there is some flexibility in defining which species is the fast-grower, so we enforce that $g_{\alpha 1} + g_{\alpha 2} > g_{\beta 1} + g_{\beta 2}$ (with α being the fast-grower/slow-switcher) to consider both growth rates.[6] We use a dilution factor of 10 and a balanced resource supply $s_1 = s_2 = 1/2$.

Stated more mathematically, we uniform-randomly sampled the volume defined by

$$\begin{cases} 0 < g_{\alpha 2} < g_{\alpha 1} < 1 \\ 0 < g_{\beta 2} < g_{\beta 1} < 1 \\ g_{\beta 1} + g_{\beta 2} < g_{\alpha 1} + g_{\alpha 2} \\ 0 < t_{\text{lag}\alpha} < 12 \end{cases} \tag{45}$$

and used $\text{DF} = 10$ and $s_1 = s_2 = 1/2$. We generated 10 million random species pairs from that volume and determined invasibility if α's lag was diauxic, if α's lag was initial, and if α had no lag. We assigned

[6] Instead using $g_{\alpha 1} > g_{\beta 1}$ would further benefit the ability of diauxic lags to support coexistence while harming the ability of initial lags to support coexistence.

qualitative competitive outcomes based on invasibility.[7] The frequencies of coexistence are:

Lag Type	Frequency of Coexistence
Diauxic	25.4%
Initial	15.3%
None	9.5%

In our results, the introduction of diauxic lags makes coexistence 2.67x more likely compared to the no-lags case, while the introduction of initial lags makes coexistence only 1.61x more likely. This is yet another example of diauxic lags benefiting coexistence to a greater extend than initial lags do.

Initial lags benefit coexistence at small to intermediate lag time values, while diauxic lags benefit coexistence at large lag time values. To develop a little more intuition, we used the same randomly sampled growth rates and varied $t_{\mathrm{lag}\alpha}$. We then looked at how often coexistence was observed at different values of $t_{\mathrm{lag}\alpha}$ (Appendix Fig S19A). Initial lags increased the frequency of coexistence from 9.5% to as high as $\sim 20\%$ at small to intermediate lag times ($0.5 \lesssim t_{\mathrm{lag}\alpha} \lesssim 5$) but actually decreased the frequency of coexistence at large lag times ($t_{\mathrm{lag}\alpha} \gtrsim 10$). By contrast, with diauxic lags, the frequency of coexistence only ever increased with increasing lag times, reaching 20% at $t_{\mathrm{lag}\alpha} \approx 4$ and 30% at $t_{\mathrm{lag}\alpha} \approx 7$.

From this information, whether initial or diauxic lags have a greater tendency to produce coexistence depends on what range of lag time values are expected. In our calculations, diauxic lags became more likely to produce coexistence than initial lags at $t_{\mathrm{lag}\alpha} \approx 3$. The mean growth rate from the sampling was $\langle g_{\mu i} \rangle = 0.5$. Putting this together we conclude that:

If the typical difference in lag times is small compared to the inverse of the typical growth rate (i.e. if $\langle \Delta t_{\mathrm{lag}} \rangle < 1.5 \langle g_{\mu i} \rangle^{-1}$) then the lags being initial will be more likely to produce coexistence than if the lag were diauxic. Conversely, if the typical difference in lag times is large (i.e. if $\langle \Delta t_{\mathrm{lag}} \rangle > 1.5 \langle g_{\mu i} \rangle^{-1}$) then the lags being diauxic will be more likely to produce coexistence. For reference, $1.5 \langle g_{\mu i} \rangle^{-1}$ is approximately the doubling time. So (with the specific distribution we have chosen) if lags are expected to last for longer than it would take the cells to divide once then giving the fast-grower a diauxic lag is more likely to produce coexistence than giving it an initial lag.

To further understand why initial and diauxic lags increase the frequency of coexistence in different ranges of $t_{\mathrm{lag}\alpha}$, we looked at which exclusion outcome occur in cases that were not coexistence ((Appendix Fig S19B-C). Under both forms of lag, the frequency of the fast-grower α excluding the slow-grower decreased with increasing $t_{\mathrm{lag}\alpha}$ while the frequency of the fast-switcher β excluding α increased. However, with diauxic lags the fast-switcher excluding the slow-switcher never exceeded 50% likelihood whereas with initial lags the fast-switcher excluding the slow-switcher eventually became the almost-always observed outcome. Increasingly large diauxic lags don't lead to the fast-switcher always excluding the slow-switcher because the slow-switcher always has a period of growth before the diauxic lags begin and can grow enough during this period to survive. (Whereas increasingly large initial lags mean the slow-switcher has less and less, and eventually no, chance to grow.) This is perhaps the main distinction between initial and diauxic

[7] Bistabilities between each species excluding the other occurred in 1.0% of cases when lags were diauxic and 0.2% of cases when lags were initial (and in no cases without lags as bistabilities were impossible). Given the low frequency of this form of bistability, we strongly suspect bistability between an exclusion and a coexistence state would be even less likely (although confirming this remains left for a more dedicated study to determine). We therefore conclude that invasibility was indeed a good proxy for coexistence. When a bistability between coexistence and exclusion states does occur, a case that was misclassified as pure exclusion actually contributes to the frequency of coexistence, so the true rates of coexistence under initial and diauxic lags can only be larger than the reported values. Thus, the presented numbers for the frequencies of coexistence under diauxic and initial lags are lower bounds suspected to be close to the true values.

lags: Diauxic lags affect only part of the growth cycle, so when the slow-switcher is the fast-grower each species has a period of distinct advantage and robust stable coexistence can readily occur. Initial lags by contrast initially delay one species but do not create two distinct periods of growth to quite the same extent and are therefore an important tradeoff but a less robust source of stable coexistence.

2.6 Summary: Diauxic lags support coexistence to a greater extent than initial lags

In this comparison of initial and diauxic lags we have shown:

- Diauxic lags allow for coexistence when all yields are constant and species have a single growth rate for both resources, but initial lags do not allow for coexistence under the same assumptions.
- For initial lags to support the coexistence of two species, the species must have different yields and dilutions must be to a specific population size (rather than by a constant factor).
- Under the equivalent scenario (different yields for each species, dilutions to a specific population size, and species still having only one growth rate) diauxic lags support three-species coexistence.
- Diauxic lags as a source of coexistence generally requires a sufficiently large difference in lag times but can occur with arbitrarily large differences in lag time, whereas initial lags generally require lag times to be within a specific range (with both upper and lower bounds).
- When sampling random species, if the difference in lag times is assumed to be more than the typical doubling time, diauxic lags are more likely to produce coexistence than initial lags.

These individual conclusions lead us to conclude that tradeoffs between growth rate and diauxic lag time are more likely to produce coexistence than tradeoffs between growth rate and initial lag time. We also conclude the coexistence states resulting from diauxic lags appear to be more robust against perturbations and require less complex tradeoffs involving fewer species parameters. This exploration is, however, limited, and considerable room is left for a dedicated study to address these questions in depth.
