# Supplementary material for: Diauxic lags explain unexpected coexistence in multi‐resource environments
Source: Mol Syst Biol. 2022 May 4;18(5):e10630. doi: 10.15252/msb.202110630 (PMC9067609; doi:10.15252/msb.202110630)
Supplement: Supplementary file 2 — Expanded View Figures PDF [file MSB-18-e10630-s004.pdf]

Expanded View Figures

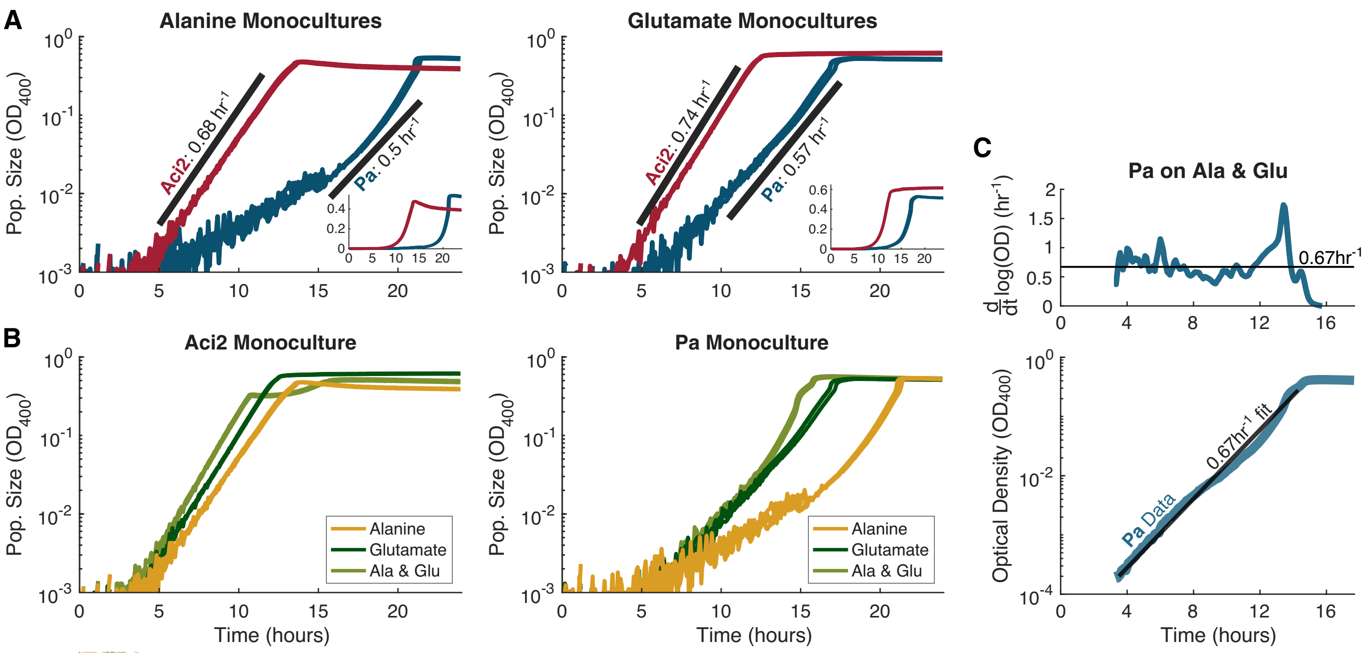

Figure EV1. Pa is the single-resource slow-grower and should be considered the slow-grower despite some fluctuations in its apparent growth rate.

A Monoculture growth rate experiments in single resource environments for Aci2 in red and Pa in blue with growth rate fits shown in black. Insets show the same data on a linear scale. On alanine, Pa has a distinct upwards curvature to its growth curve. This could represent a density-dependent growth rate with a peak instantaneous growth rate close to Aci2's. Pa should nevertheless be considered the slow-grower because for the majority of its growth it is growing much slower than Aci2 and its increase in growth rate occurs over only the last ~2 h before it saturates. The value of 0.5/h is representative across its growth.

B Direct comparisons of each species' growth in each environment highlights similarity of growth rates across environments and in particular the similarity of the glutamate growth rates to the two-resource growth rates, which is especially relevant because in competition glutamate is the resource that is most often being switched to.

C Pa's two-resource growth rate fit from Fig 2A extended across the entirety of the data. Although Pa's measured growth rate has some small fluctuations, the growth rate fit of 0.67/h is a good overall fit. The spike in growth rate around 13 h is discussed in Appendix Fig S5, which also presents reasons why Pa's optical density may not be a constant function of its biomass or population size and why modeling the small variations would likely be overfitting to experimental artifacts and not actual growth dynamics.

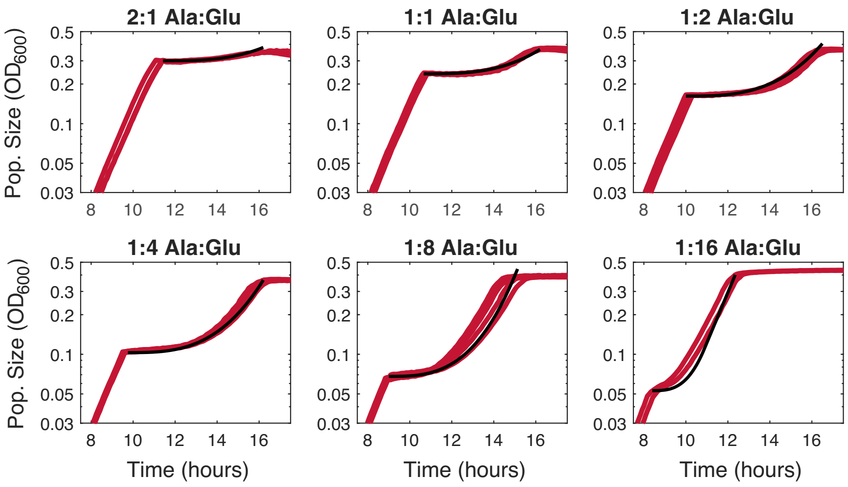

Figure EV2. Example lag time fits.

The Aci2 monoculture data from Fig 2B are shown in red with the fits used to produce the lag time estimates shown in red (Materials and Methods). The remaining Aci2 fits are presented in Appendix Fig S3. The Pa fits are presented in Appendix Fig S4 with a note on interpretation of Pa's optical density data provided in Appendix Fig S5. These fits were performed using the species' two-resource growth rates as their post-recovery steady-state growth rates to maintain consistency with modeling decisions. Fits using the species' single-resource growth rates are provided in Appendix Fig S7 and differ only slightly.

 © 2022 The Authors

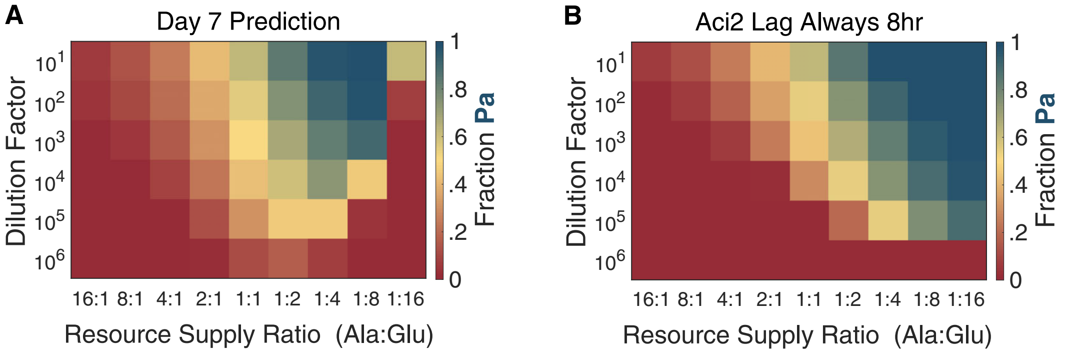

Figure EV3. Predicted mean fraction at Day 7 and predicted steady-state if Aci2's lag did not vary with resource supply.

A Predicted mean fraction Pa after seven dilution cycles of competition. Shown is the average fraction Pa from competitions started with a Pa fraction of 0.1, 0.25, 0.75, and 0.9 (same as in experiments).

B Predicted steady-state fraction Pa if Aci2's lag did not vary with resource supply but was instead constant at 8 h. The value of 8 h was chosen for being close to Aci2's mean lag time of 9 ± 1 h and providing a close fit to the experimental results.

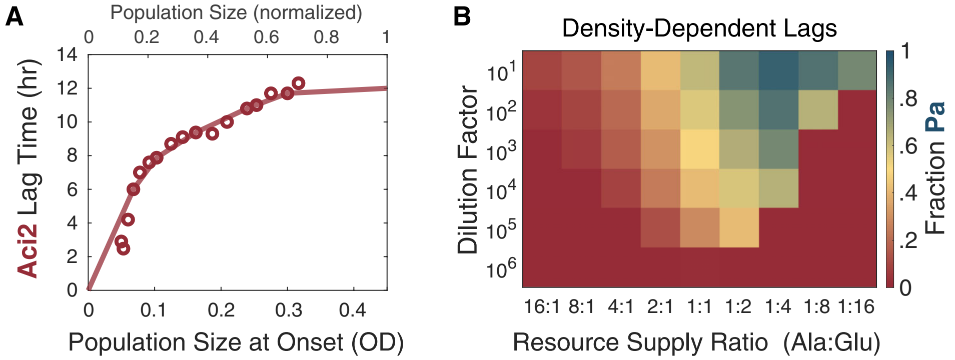

Figure EV4. Modeling Aci2's lags as dependent on its population density at the onset of its diauxic shift (instead of initial alanine supply) prevents Pa from excluding Aci2.

A Using the lag times presented in Fig 2H and the population sizes at the onset of Aci2's diauxic shift presented in Fig 2C allows Aci2's lag time to be alternatively expressed as a function of its population size at the onset of its diauxic shift. Circles represent lag time data and the line defines the piecewise linear function used for modeling. Whether Aci2's lag is most accurately modeled as a function of alanine supply or of population size at the time of its diauxic shift could not be definitively determined (Appendix Fig S8).

B Steady-state phase space for the Aci2 versus Pa on alanine and glutamate competition at various resource supply ratios and dilution factors when modeling Aci2's lag as a function of its population size at the onset of the diauxic shift. The most significant change relative to the original prediction (Fig 5B) is that Pa never fully excludes Aci2. Pa cannot exclude Aci2 because as Aci2 is driven extinct its population size at its diauxic shift as well as its diauxic lag time converge to zero, eliminating the period when Pa can catch up to Aci2.

© 2022 The Authors
