# Supplementary material for: Diauxic lags explain unexpected coexistence in multi‐resource environments
Source: Mol Syst Biol. 2022 May 4;18(5):e10630. doi: 10.15252/msb.202110630 (PMC9067609; doi:10.15252/msb.202110630)
Supplement: Supplementary file 5 — Source Data for Figure 6 [file MSB-18-e10630-s005.pdf]

Figure 6 Source Data

for

Diauxic lags explain unexpected coexistence
in multi-resource environments

Bloxham, Lee, Gore

This document contains the growth-lag characterizations and coculture competitive outcomes behind the summary statistics provided in Fig 6. Each page contains the data for a different pair of resources.

On the top half of each page, monoculture optical density is shown for 1-2 replicates of three different resource supply ratios (4:1, 1:1, 1:4) with one panel for each species. For cases in which both a growth rate and a diauxic lag time could be fit, the growth-lag fit is shown in black, and the growth rate and lag time values are given in the panel title. All units are hr^{-1} for growth rates and hours for lag times. The sixth panel shows the growth rates and lag times extracted from those fits.

Across the middle of each page, coculture species fractions over the course of five days are shown. A circle on the right edge of a panel indicates a competition in which coexistence was determined to be the competitive outcome. The location of this circle is the mean species fraction on Day 5.

At the bottom of each page, comments on specific growth-lag characterization are provided. These comments include reasons why growth rates and diauxic lag times could not fit in some cases, justifications for why specific parts of the OD curves were used for fitting growth rates and lag times, and additional detail on day-by-day species fraction when competitions were determined to be competitive exclusion and when borderline cases were determined to be coexistence.

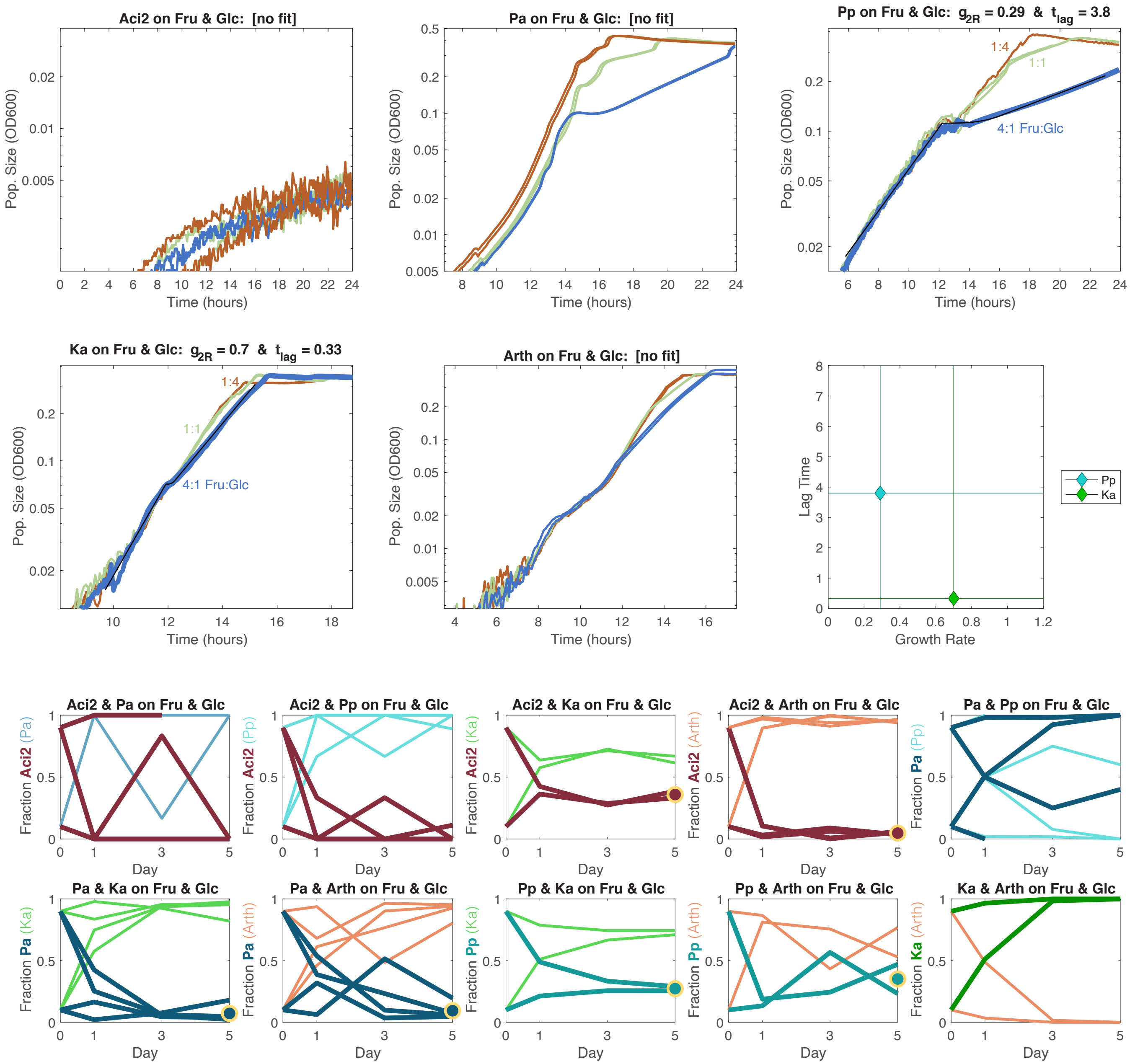

Growth-lag characterizations and competitive outcomes on fructose and glucose.

Comments on monoculture growth-lag characterizations (top half):

- Pa: Growth-lag could not be fit due to multiple extra diauxic shifts (three total observed).
- Pp: Lag time is slightly uncertain due to noise, but not by enough to change relative ordering with Ka.
- Arth: Growth rate could not be fit due lack of a sufficient period of exponential growth before the diauxic shift. Using the period from approximately 9 hours to 11 hours would lead to a negative lag time.

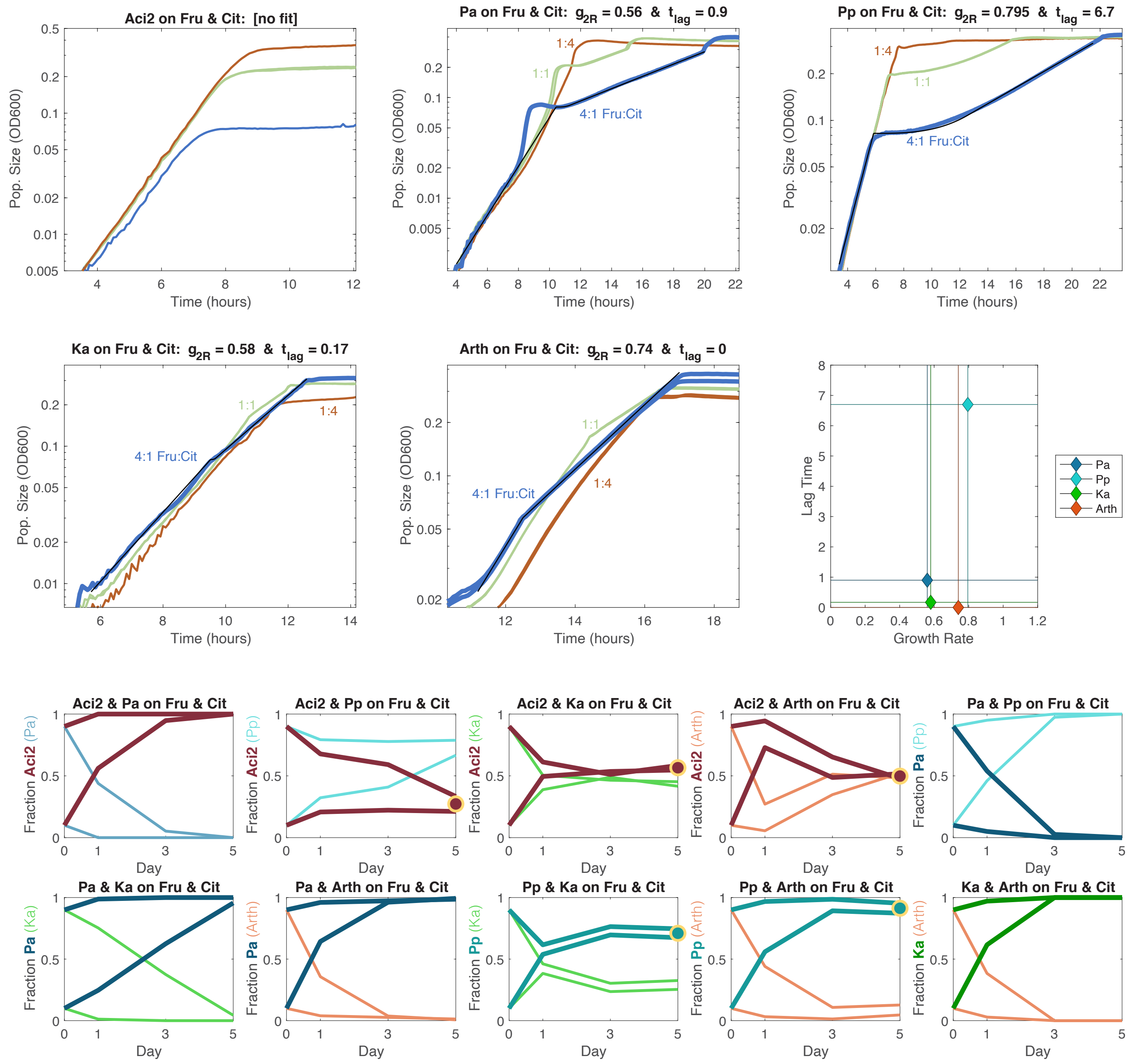

Growth-lag characterizations and competitive outcomes on fructose and citrate.

Comments on monoculture growth-lag characterizations (top half):

- Aci2: Growth-lag could not be fit due to no growth on fructose. Comment applies to all Aci2 experiments involving fructose or glucose.
- Pa: Lag time was fit using a “corner-cutting” assumption. The increase in optical density at or just before saturation is assumed to be an artifact, so the line used to fit the growth rate is extended, and the lag time is calculated starting from when the OD curve reintersects this line.

Comments on coculture competitions (bottom half):

- Pa & Pp: Neither replicate had any Pa colonies on Day 5.
- Pa & Ka: One replicate still had 4% Ka on Day but appeared to be converging steadily towards extinction, and other replicate had no Ka colonies on Day 3 or 5.
- Pa & Arth: Replicates had 1.3% and 0.7% Arth on Day 5.
- Ka & Arth: Neither replicate had any Arth colonies on Day 5.

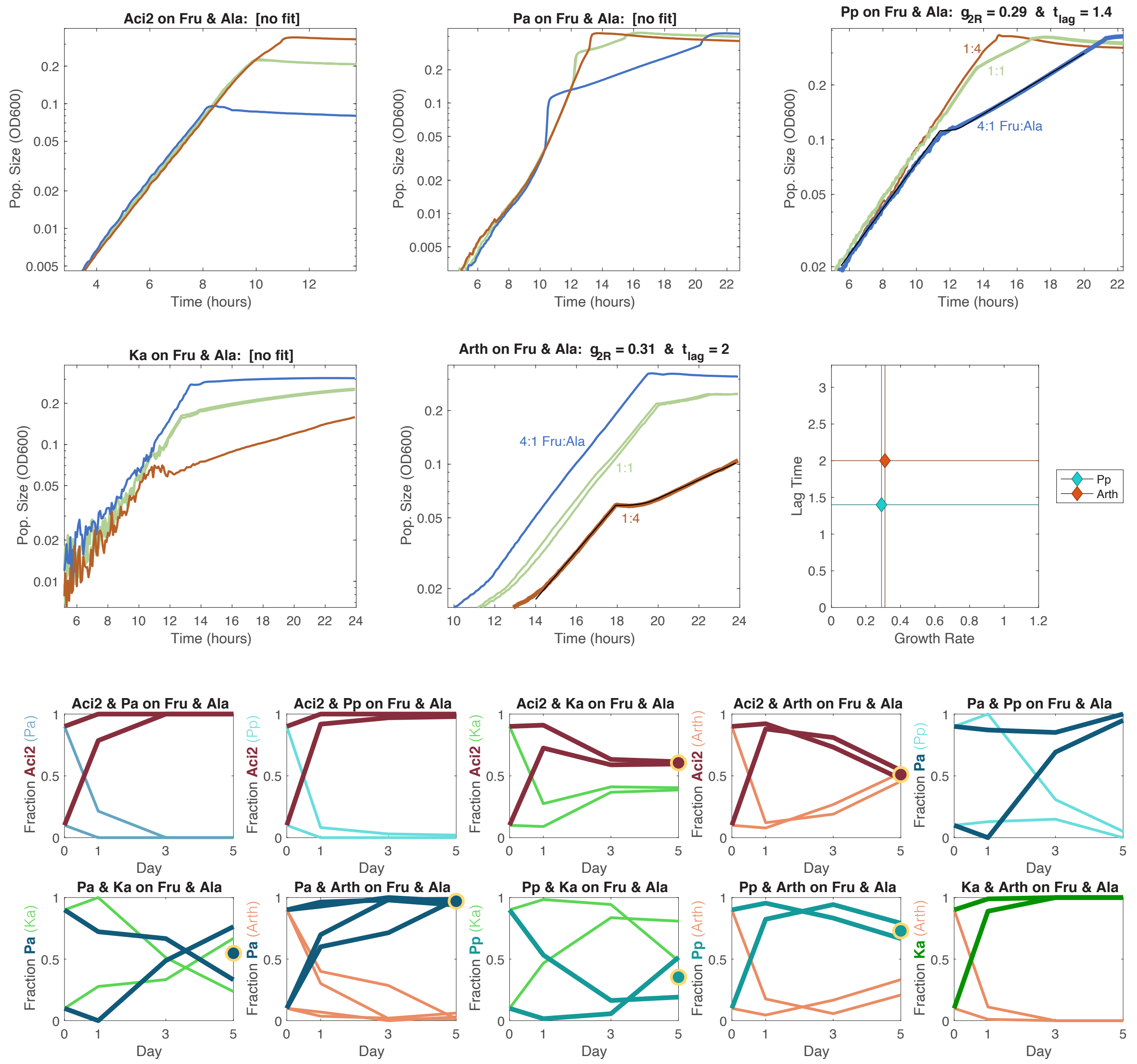

Growth-lag characterizations and competitive outcomes on fructose and alanine.

Comments on monoculture growth-lag characterizations (top half):
- Pa: Slight upwards curvature of the pre-shift growth curve and large OD spike at onset of diauxic shift led to negative lag time fits.
- Ka: Noise and gradually slowing growth rate during post-shift growth made any fit uncertain. Ka does not grow in alanine single-resource environment, and no growth-lag fits were achieved when one resource was alanine.

Comments on coculture competitions (bottom half):
- Pa & Pp: One replicate had no Pp colonies on Day 5. In the other replicate, Pp declined from 31% to 5% from Day 3 to Day 5, which was taken as evidence of convergence towards extinction.
- Pa & Arth: In the four replicates, from Day 3 to Day 5, Arth's population fraction increased from 0% (+/-3%) to 3%; decreased from 29% to 2%; increased from 2% to 6%; and decreased from 2% to 1%. Because Arth maintained a small fraction on Day 5 (rather than going extinct) in cases when it was near extinction on Day 3, this competitive outcome was classified as low-level coexistence rather than competitive exclusion.

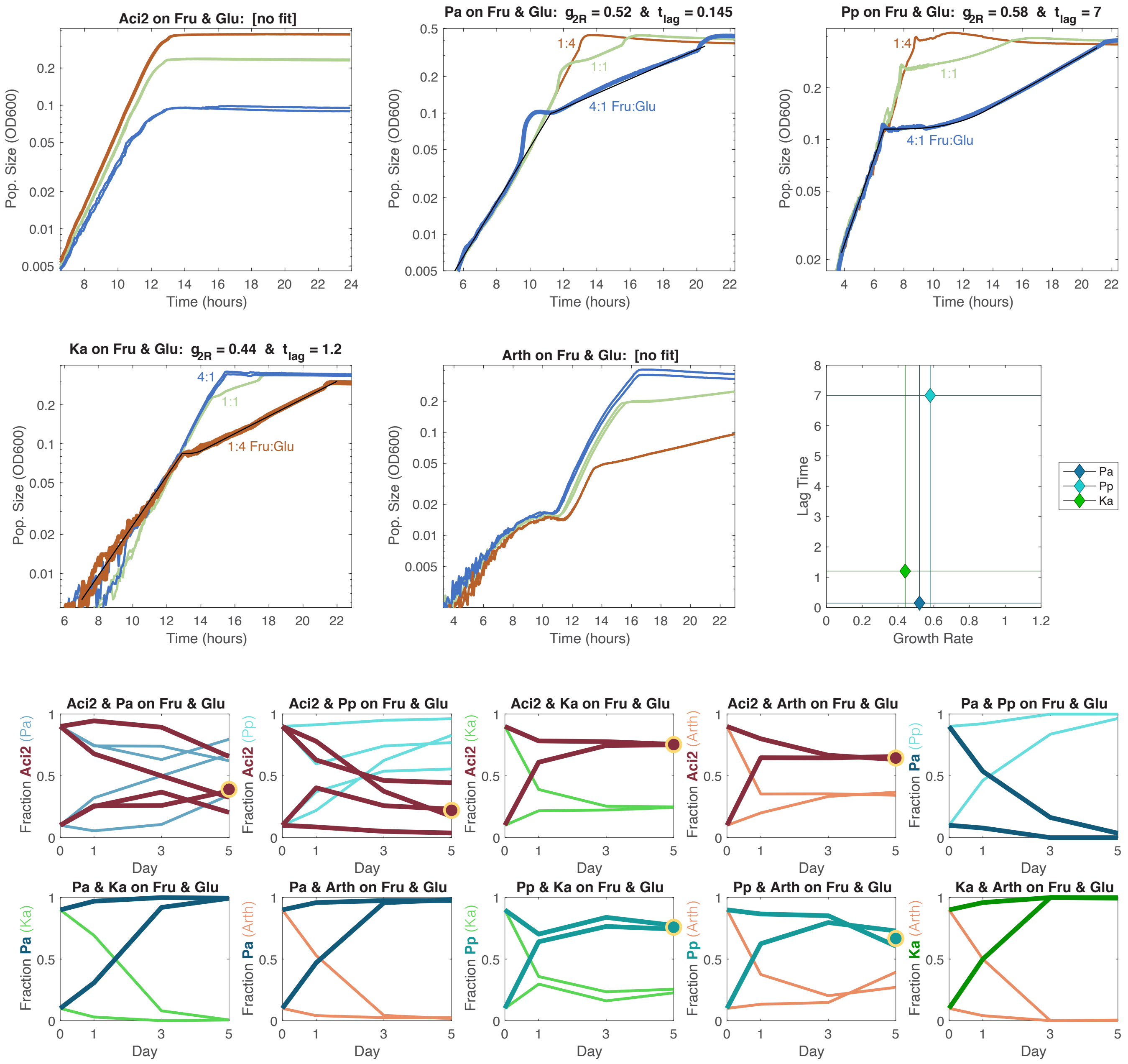

Growth-lag characterizations and competitive outcomes on fructose and glutamate.

Comments on monoculture growth-lag characterizations (top half):

- Pa: Same "corner-cutting" assumption was made as for fructose and citrate fit.
- Arth: Fit not attempted due to strange shape of growth curve.

Comments on coculture competitions (bottom half):

- Pa & Pp: One replicate had no Pp colonies on Day 5. The other replicate had 4% Pp on Day 5, having decreased from 16% on Day 3.
- Pa & Ka: Both replicates had <1% Ka on Day 5.

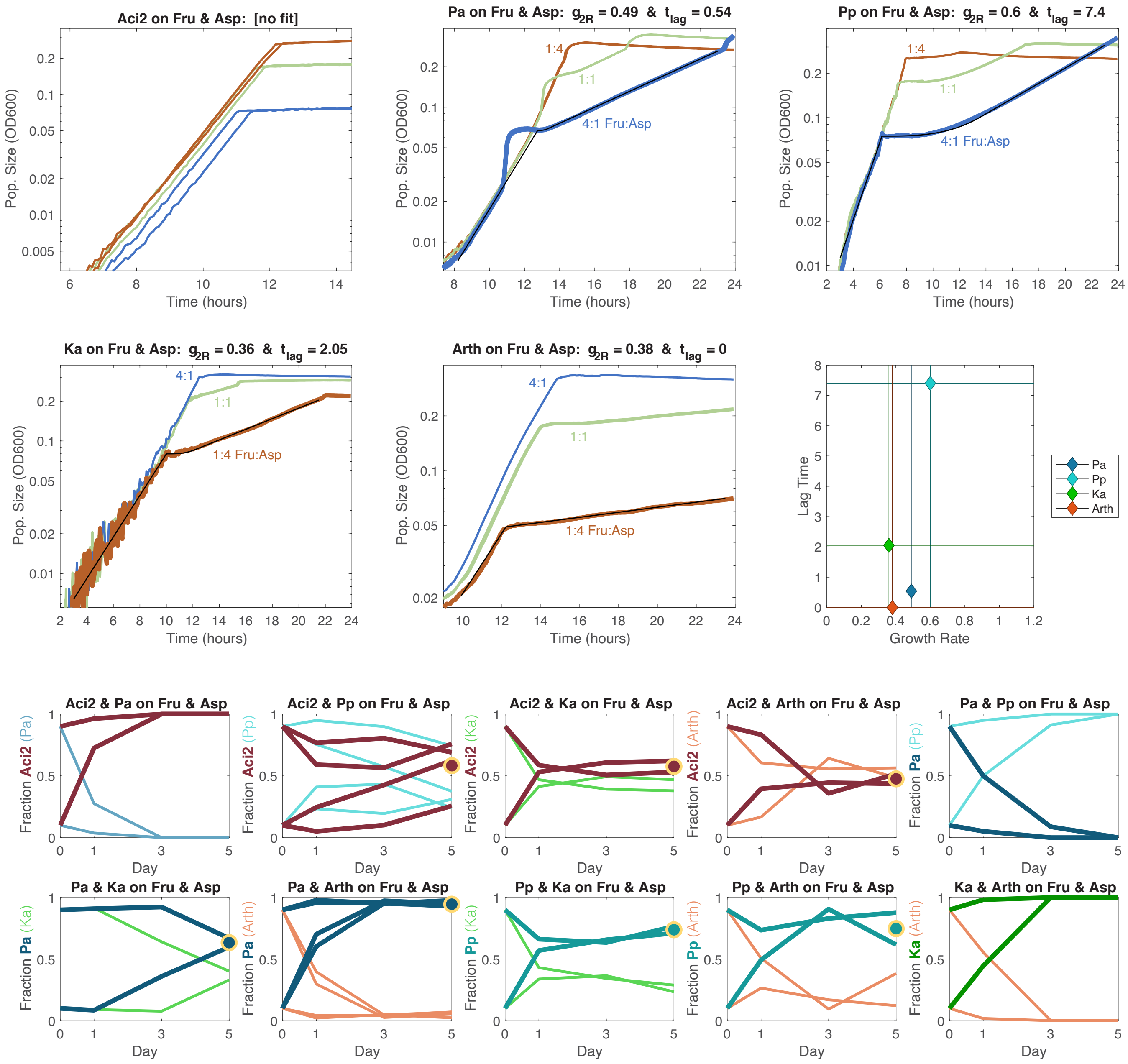

Growth-lag characterizations and competitive outcomes on fructose and aspartate.

Comments on monoculture growth-lag characterizations (top half):

- Pa: Same "corner-cutting" assumption as previous (e.g. fructose and citrate).

Comments on coculture competitions (bottom half):

- Pa & Pp: Neither replicate had any Pp colonies on Day 5.
- Pa & Arth: In the four replicates, from Day 3 to Day 5, Arth's population fraction decreased from 5% to 2%; increased from 5% to 7%; increased from 4% to 7%; and increased from 3% to 5%. (Similar to case of Pa and Arth on fructose and alanine) because Arth maintained a small fraction on Day 5 (rather than going extinct) in cases when it was near extinction on Day 3, this competitive outcome was classified as low-level coexistence rather than competitive exclusion.
- Ka & Arth: Neither replicate had any Arth colonies on Day 5.

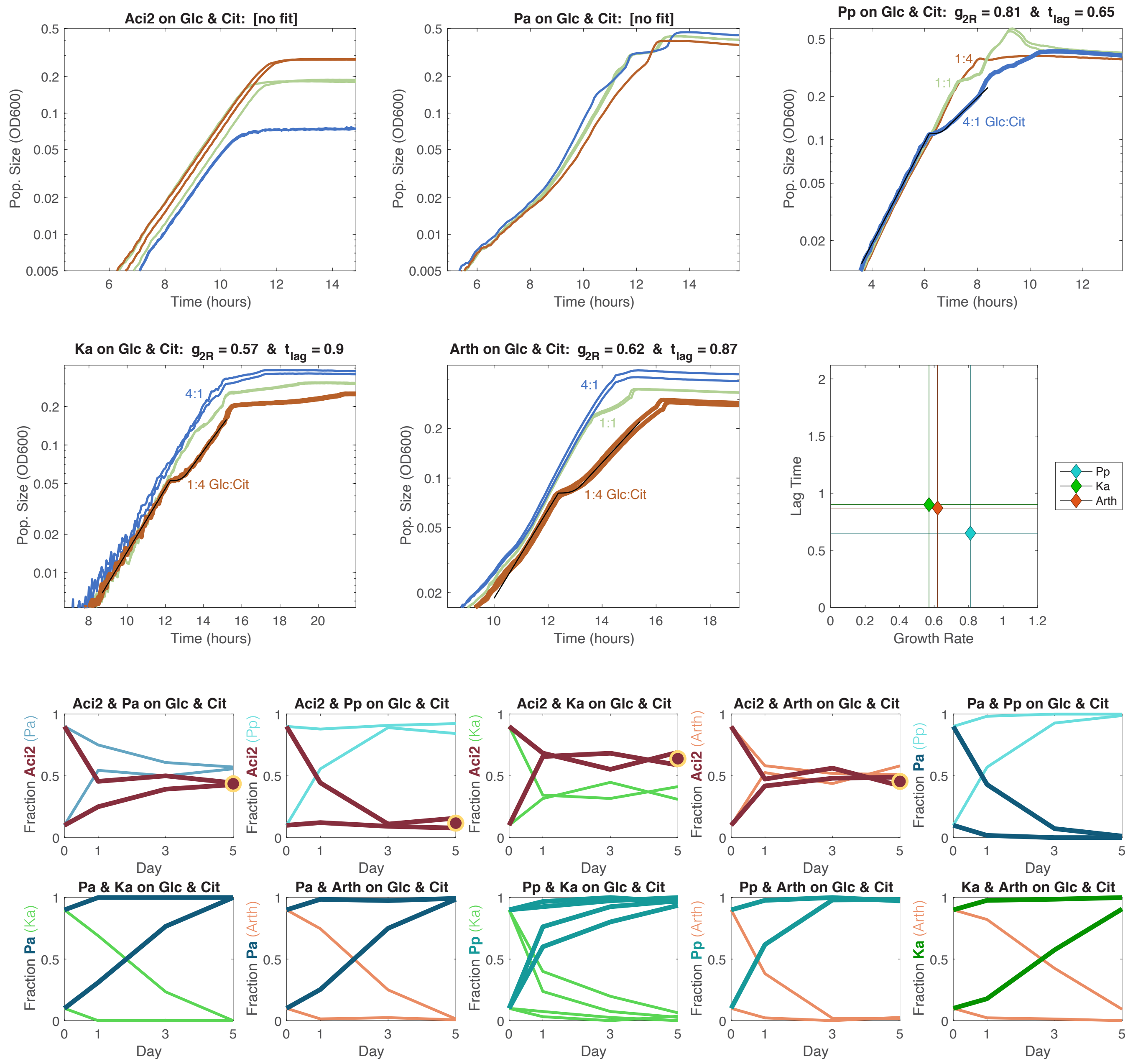

Growth-lag characterizations and competitive outcomes on glucose and citrate.

Comments on monoculture growth-lag characterizations (top half):

- Pa: Growth-lag was not fit due to extra diauxic shifts. Comment applies to all Pa experiments involving glucose.

Comments on coculture competitions (bottom half):

- Pp & Ka: In the four replicates, from Day 3 to Day 5, Ka's population fraction increased from 0% (+/-1%) to 4%; decreased from 20% to 6%; decreased from 3% to 0%; and decreased from 7% to 3%. The one replicate in which Ka's population fraction increased from 0% (+/-1%) to 4% is the only example of Ka's population fraction increasing over a sampling interval, so there was not sufficient evidence to conclude coexistence.
- Pp & Arth: The two replicates had 2% and 3% Arth on Day 5.
- Ka & Arth: One replicate had no Arth colonies on Day 5. The other replicate had 9% Arth on Day 5, having decreased from 43% on Day 3.

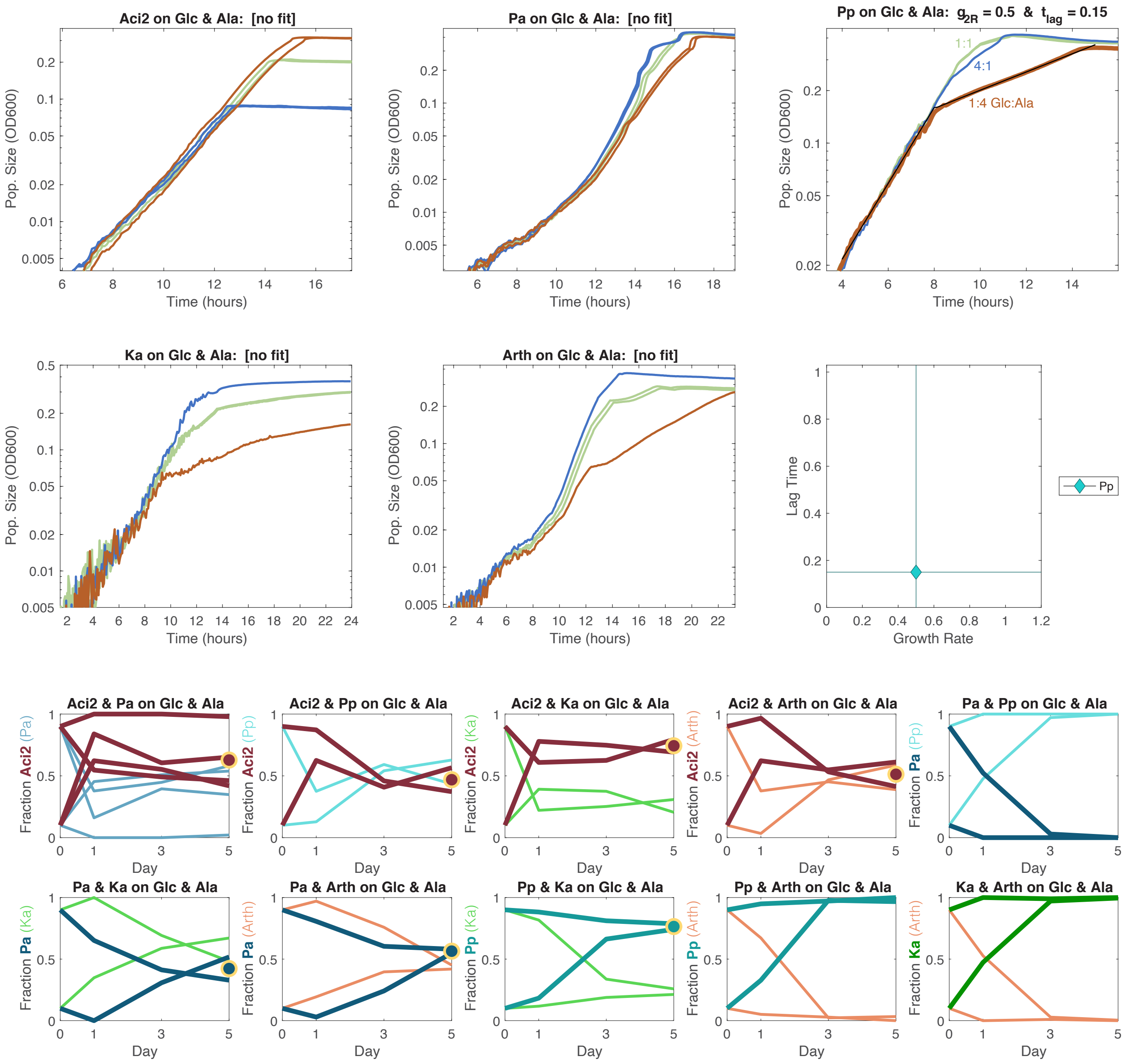

Growth-lag characterizations and competitive outcomes on glucose and alanine.

Comments on monoculture growth-lag characterizations (top half):

- Aci2: Growth-lag could not be fit due to no growth on fructose.
- Pa: Growth-lag was not fit due to extra diauxic shifts (same as all case involving glucose).
- Ka: Noise and gradually slowing growth rate during post-shift growth made any fit uncertain.
- Arth: Growth-lag was not fit due to extra diauxic shifts (most prominent in the 1:1 pale green curve) and also due to an uncertainty about which part of the curve should be used to fit the pre-shift growth rate.

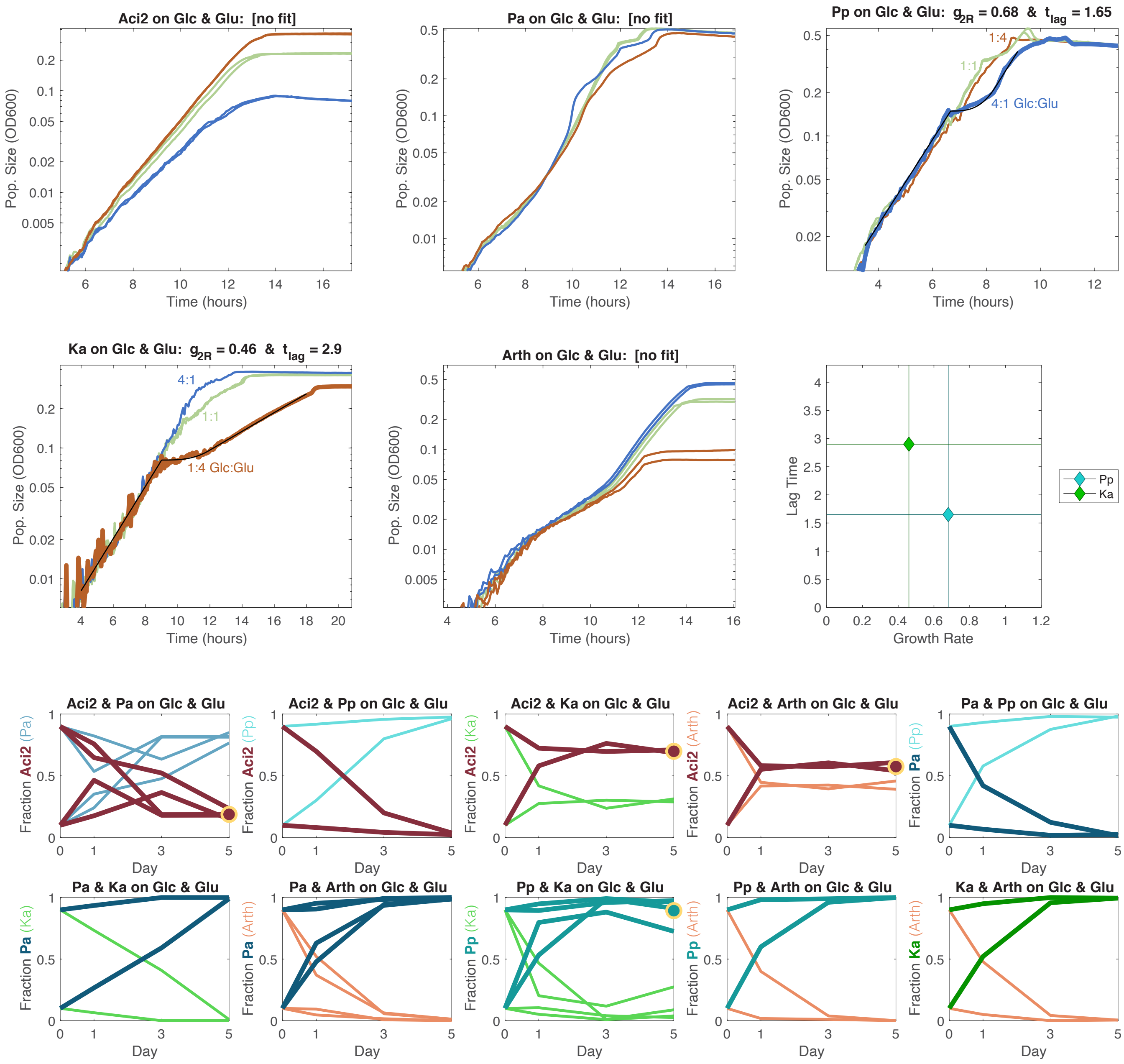

Growth-lag characterizations and competitive outcomes on glucose and glutamate.

Comments on monoculture growth-lag characterizations (top half):

- Pp: Uncertainty in the best post-shift steady-state growth rate creates some uncertainty in the lag time, but not enough to change the fast-slow ordering of Pp and Ka.
- Arth: Growth-lag could not be fit due to no growth on glutamate.

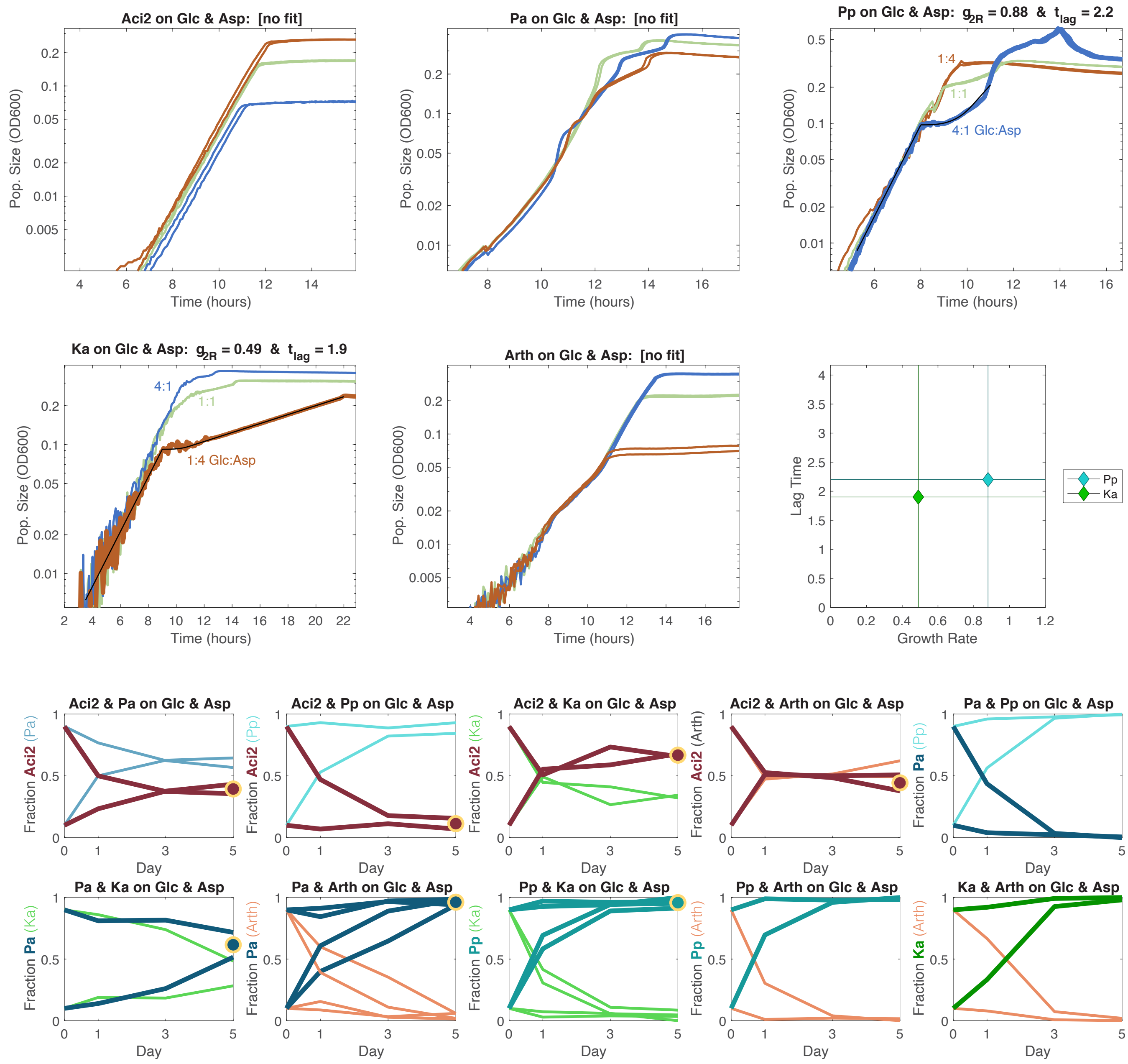

Growth-lag characterizations and competitive outcomes on glucose and aspartate.

No comments necessary; considered self-explanatory or having same explanations as previously repeated.

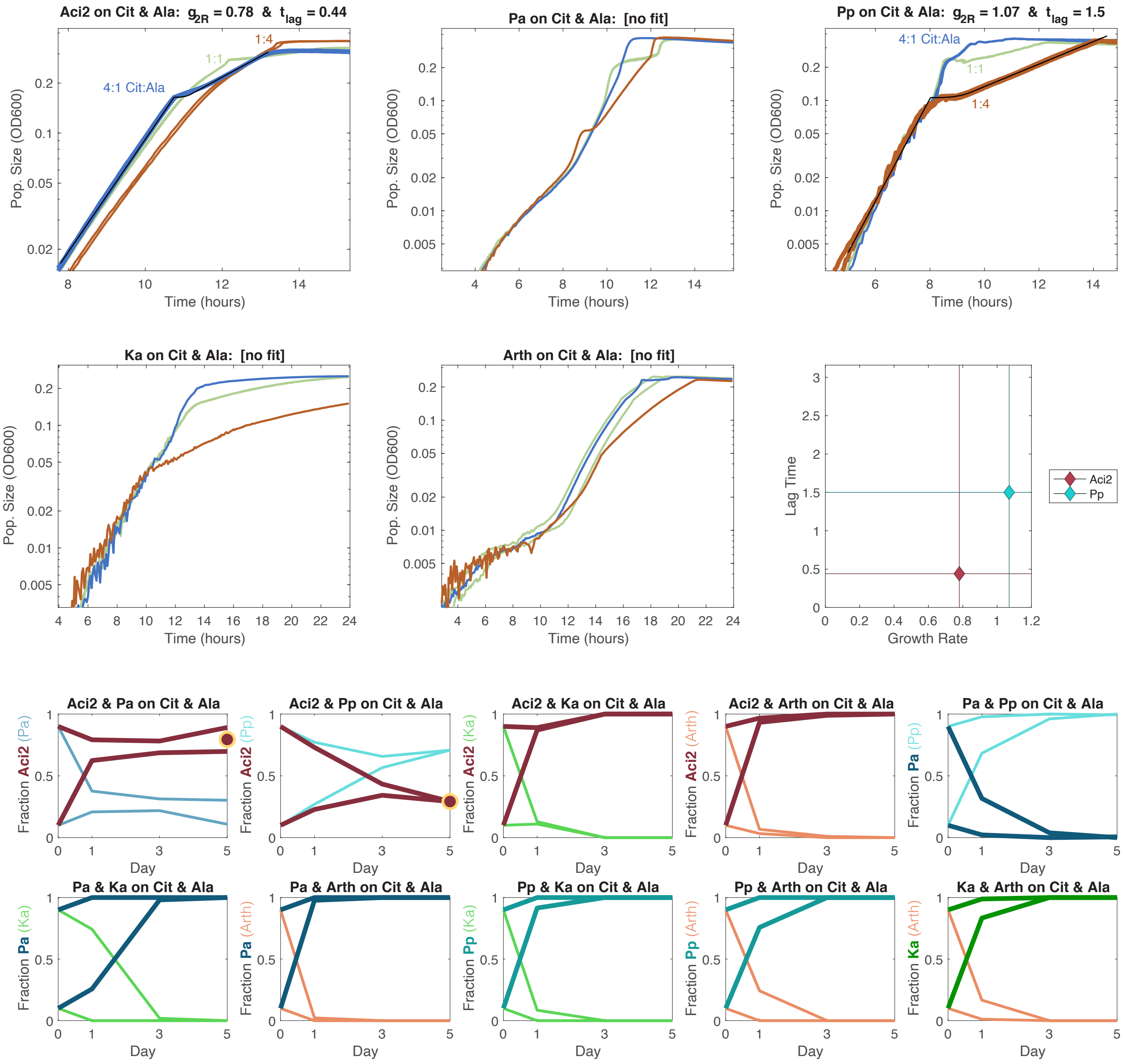

Growth-lag characterizations and competitive outcomes on citrate and aspartate.

Comments on monoculture growth-lag characterizations (top half):

- Pa: Shape of optical density curve led to negative lag-time fits. Any fit would involve too much guesswork.
- Ka: Could not be fit due to same reasons as previous cases with Ka and one resource being alanine.
- Arth: Could not be fit due to no clear choice for where to fit the pre-shift growth rate.

v

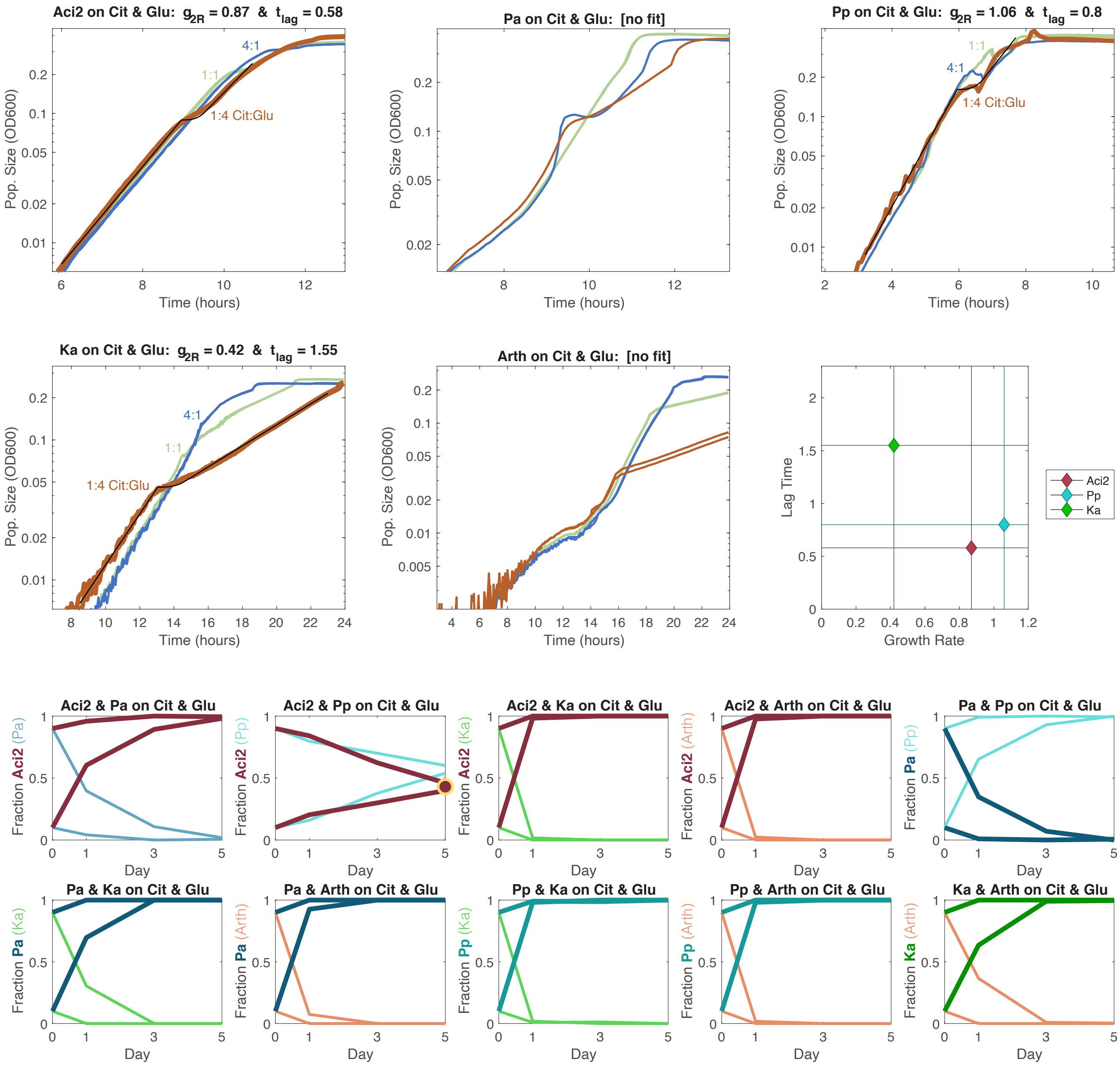

Growth-lag characterizations and competitive outcomes on citrate and glutamate.

Comments on monoculture growth-lag characterizations (top half):

- Pa: Shape of optical density curve led to negative lag-time fits. Any fit would involve too much guesswork.
- Arth: Could not be fit due to no clear choice for where to fit the pre-shift growth rate.

Comments on coculture competitions (bottom half):

- Aci2 & Ka: Neither replicate had any Ka colonies on Day 5.
- Pp & Ka: Neither replicate had any Ka colonies on Day 5.

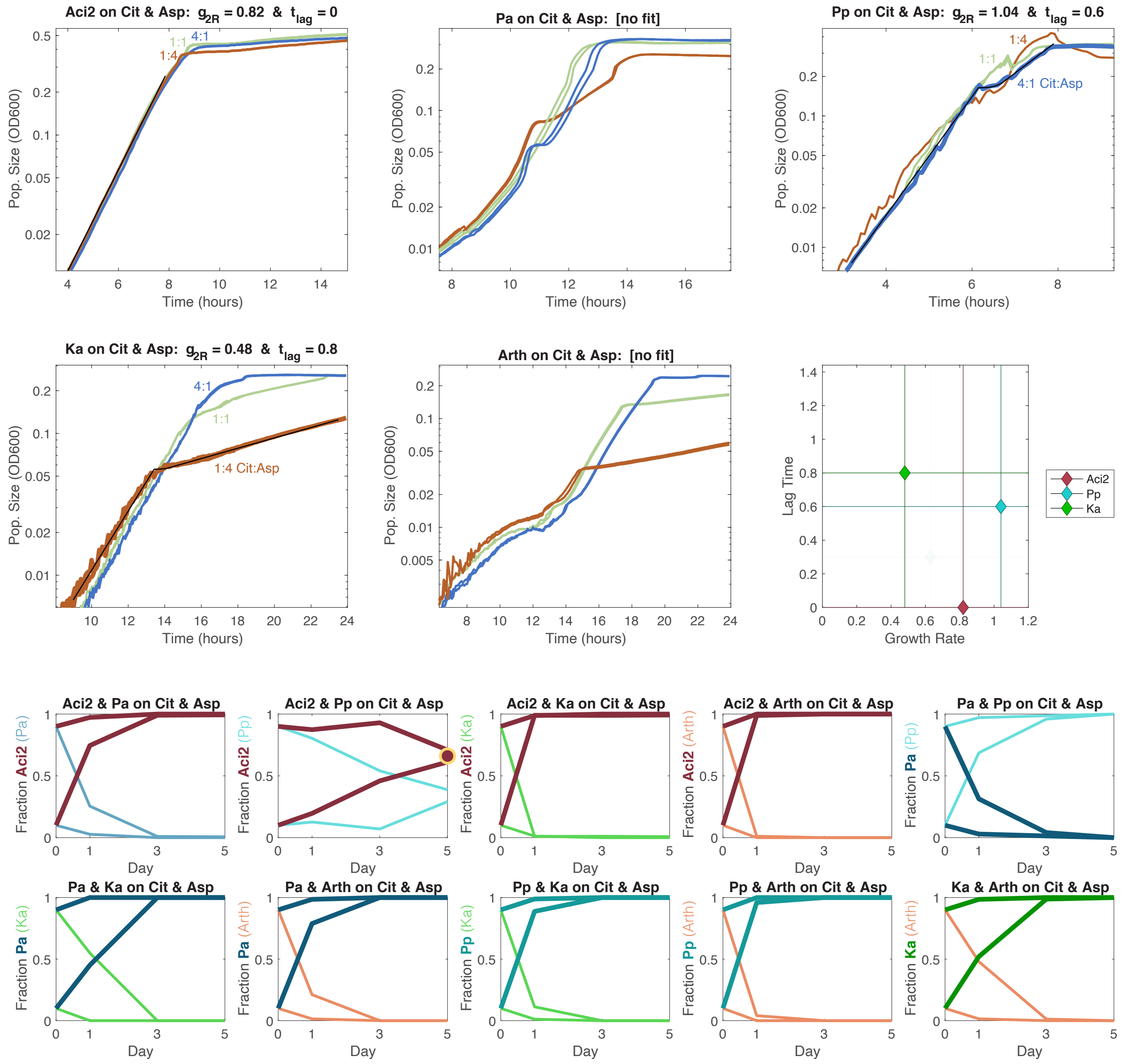

Growth-lag characterizations and competitive outcomes on citrate and aspartate.

Comments on monoculture growth-lag characterizations (top half):

- Aci2: Growth appears to be steady exponential until saturation, but saturation density varies by too little for Aci2 to only be eating one of the resources, so characterized as having no lag time.
- Pa: Shape of optical density curve led to negative lag-time fits. Any fit would involve too much guesswork.
- Arth: Fit not attempted due to strange shape of growth curve.

Comments on coculture competitions (bottom half):

- Aci2 and Ka: One replicate had no Ka colonies on Day 5. The other replicate had 0.8% Ka.
- Pp & Ka: Neither replicate had any Ka colonies on Day 5.

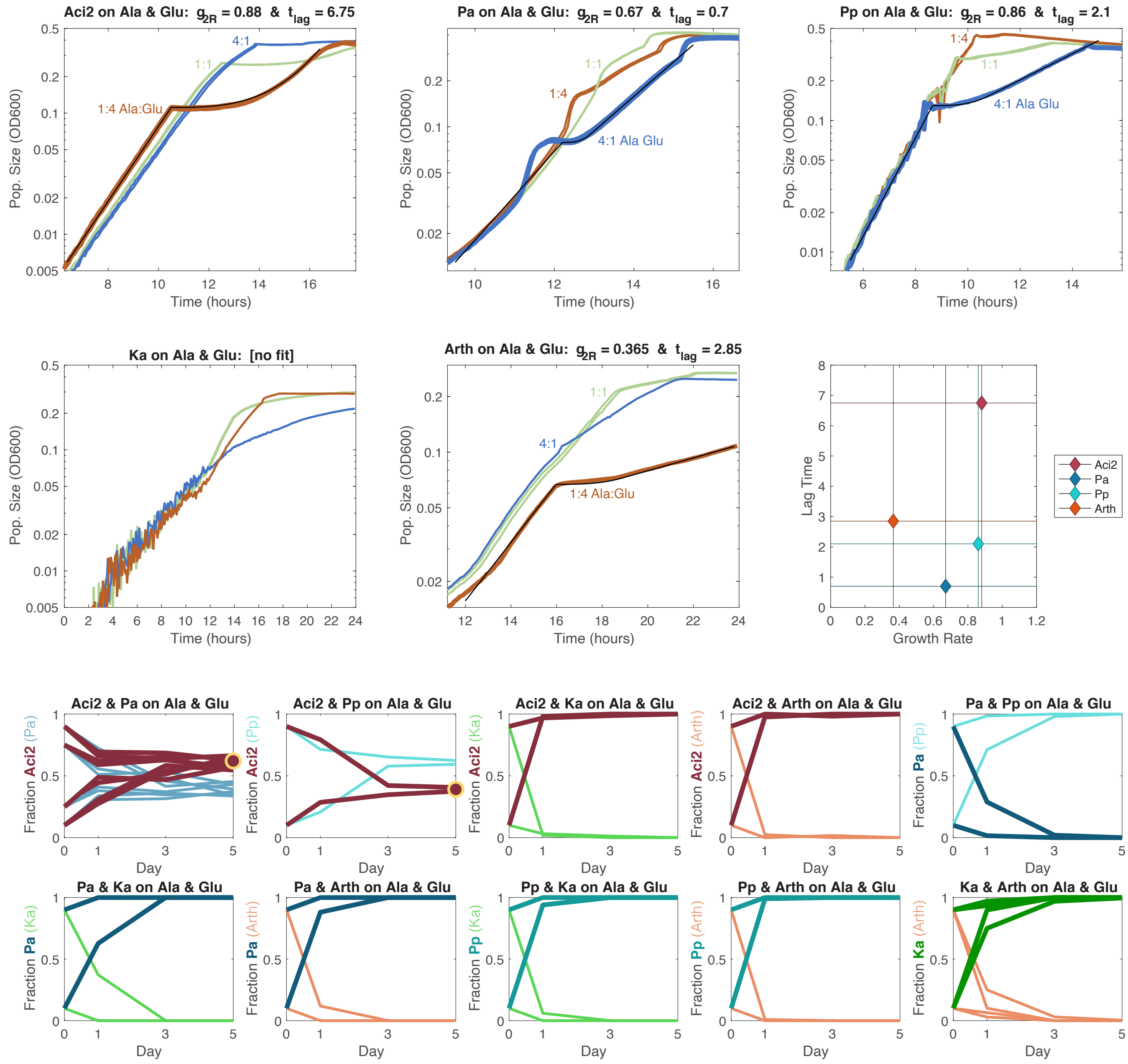

Growth-lag characterizations and competitive outcomes on alanine and glutamate.

Comments on monoculture growth-lag characterizations (top half):

- Aci2: Lag time value differs slightly from the value used elsewhere due to using Aci2's glutamate growth rate rather than its two-resource growth rate for its post-shift stead-state growth rate (Appendix Fig S7). In the modeling of Aci2 and Pa on alanine and glutamate, the two-resource growth rate was used during the lag time fits to maintain consistency with the modeling. But, for comparison to the other species, the post-shift steady-state growth rate was unconstrained so that any fitting biases would apply equally to all species.
- Pa: Same "corner-cutting" assumption as previous (e.g. fructose and citrate) and same note as for Aci2 about having a slightly different value than used elsewhere.
- Ka: Could not be fit due to same reasons as previous cases with Ka and one resource being alanine.

Comments on coculture competitions (bottom half):

- Aci2 & Arth, Pa & Pp, Pa & Arth, and Pp & Arth: Neither replicate had any colonies of the excluded species on Day 5 in each of these competitions.

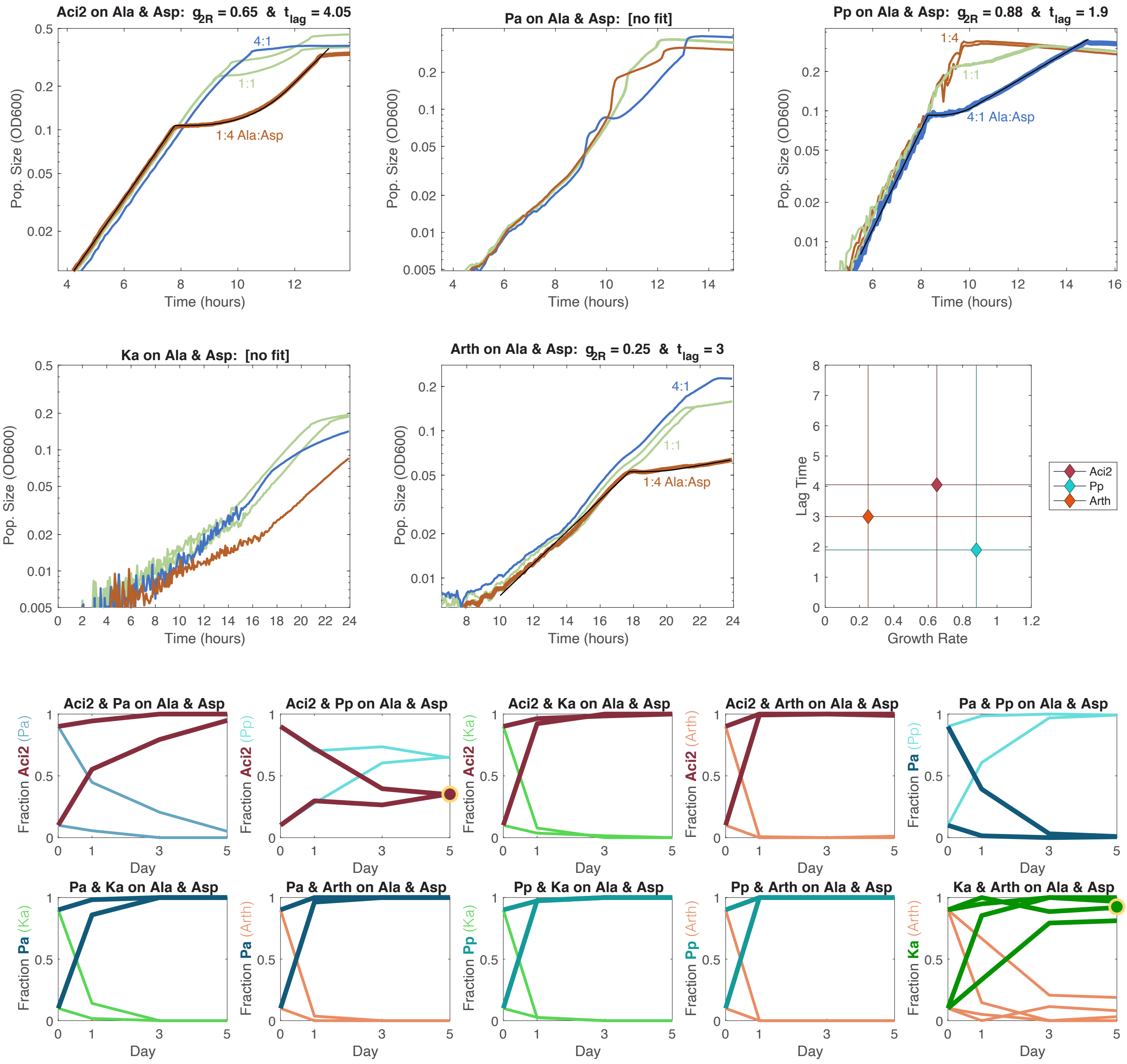

Growth-lag characterizations and competitive outcomes on alanine and aspartate.

Comments on monoculture growth-lag characterizations (top half):

- Pa: Growth-lag was not fit due to strange double-bump shape at time of shift. Additionally, shape of optical density curve led to negative lag-time fits. For either reason, any fit would involve too much guesswork.

Comments on coculture competitions (bottom half):

- Aci2 & Arth: One replicate had no Arth colonies on Day 5. The other replicate had 1% Arth.
- Pp & Arth: Neither replicate had any Arth colonies on Day 5.

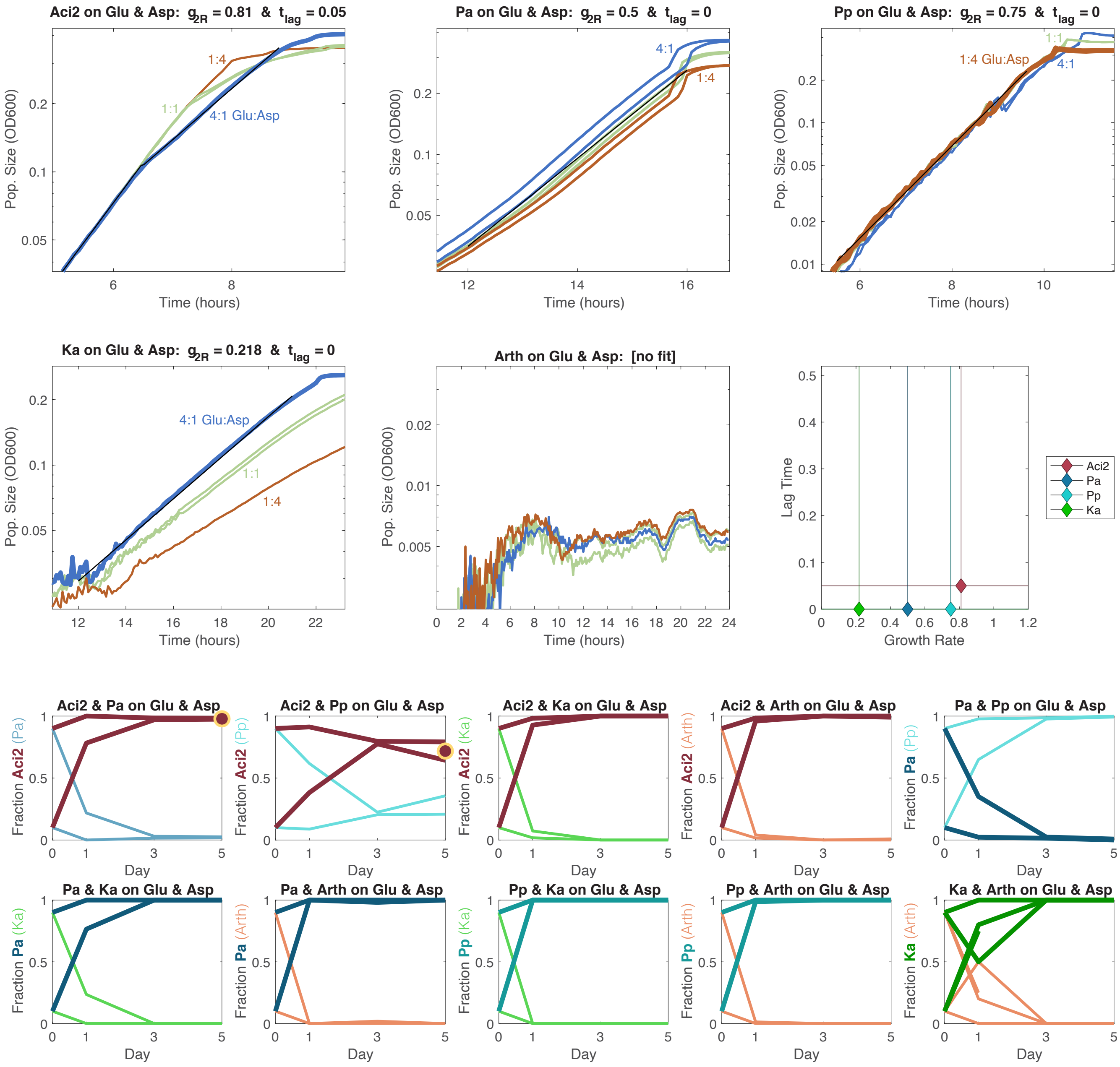

Growth-lag characterizations and competitive outcomes on glutamate and aspartate.

Comments on monoculture growth-lag characterizations (top half):

- Pa, Pp, and Ka: Growth appears to be steady exponential until saturation, but saturation densities vary by too little for these species to only be eating one of the resources, so the species were assumed to be consuming both resources with no lag time after the first depletion. (Doing this for multiple species led to three cases of species having the same lag time; these were characterize as the slow-grower not being the fast-switcher in the statistics.)

Comments on coculture competitions (bottom half):

- Aci2 & Pa: From Day 3 to Day 5, Pa's population fraction increased from 1.5% to 1.7% in one replicate and decreased from 3.0% to 2.5% in the other replicate. As both replicates held essentially steady species fractions over two days, this competitive outcome was classified as low-level coexistence.
- Aci2 & Ka: Neither replicate had any Ka colonies on Day 5.
- Pa & Pp: One replicate had no Pa colonies on Day 5. The other replicate had 0.8% Pa.
- Pa & Ka: Neither replicate had any Ka colonies on Day 5.
- Pp & Ka: Neither replicate had any Ka colonies on Day 5.
