# Supplementary material for: Diauxic lags explain unexpected coexistence in multi‐resource environments
Source: Mol Syst Biol. 2022 May 4;18(5):e10630. doi: 10.15252/msb.202110630 (PMC9067609; doi:10.15252/msb.202110630)
Supplement: Supplementary file 6 [file MSB-18-e10630-s006.pdf]

molecular systems biology

Diauxic lags explain unexpected coexistence in multi-resource environments

Blox Bloxham, Hyunseok Lee, and Jeff Gore
DOI: 10.15252/msb.202110630

Corresponding author: Jeff Gore (gore@mit.edu)

Review Timeline:

Submission Date:	6th Aug 21
Editorial Decision:	13th Sep 21
Revision Received:	21st Jan 22
Editorial Decision:	25th Mar 22
Revision Received:	31st Mar 22
Accepted:	1st Apr 22

Editor: Maria Polychronidou

Transaction Report:

(Note: With the exception of the correction of typographical or spelling errors that could be a source of ambiguity, letters and reports are not edited. Depending on transfer agreements, referee reports obtained elsewhere may or may not be included in this compilation. Referee reports are anonymous unless the Referee chooses to sign their reports.)

13th Sep 2021

RE: MSB-2021-10630, Diauxic lags explain unexpected coexistence in multi-resource environments

Thank you again for submitting your work to Molecular Systems Biology. We have now heard back from the three referees who agreed to evaluate your study. Overall, the reviewers acknowledge that the study is a relevant contribution to the field. They raise however a series of concerns, which we would ask you to address in a major revision.

I think that the reviewers' recommendations are clear and I therefore see no need to repeat the points listed below. All issues raised by the reviewers need to be satisfactorily addressed. Please contact me in case you would like to discuss in further detail any of the issues raised.

On a more editorial level, we would ask you to address the following points:

- Please include 5 keywords.

- Please include an "Author Contributions" and a "Conflict of Interest" statement in the main text.

- Please provide a .doc file for the manuscript text (including legends for the main figures) and individual production-quality files for the main figures (one file per figure).

- We have replaced Supplementary Information by the Expanded View (EV format). In this case, all additional figures can be included in a PDF called Appendix. Appendix figures should be labeled and called out as: "Appendix Figure S1, Appendix Figure S2..." etc. Each legend should be below the corresponding Figure in the Appendix. Please include a Table of Contents in the beginning of the Appendix. For detailed instructions regarding expanded view please refer to our Author Guidelines: .

- Description of Results in the Appendix should be avoided as much as possible.

- Please provide a "standfirst text" summarizing the study in one or two sentences (approximately 250 characters), three to four "bullet points" highlighting the main findings and a "synopsis image" (550px width and max 400px height, jpeg format) to highlight the paper on our homepage.

- Given the quantitative nature of the study we would encourage you to provide the Source Data for the Figure panels showing essential quantitative information. Source Data for main figures should be provided in .zip Folders labeled "Source data for Figure X". Please provide one .zip folder for each of the main figures. Source Data for Appendix Figures should all be provided in one single .zip folder labeled "Source Data for Appendix". Further information regarding Source Data can be found here: .

- Please include a Data availability section describing how the data and code have been made available. This section needs to be formatted according to the example below:
The datasets and computer code produced in this study are available in the following databases:
- Chip-Seq data: Gene Expression Omnibus GSE46748 (https://www.ncbi.nlm.nih.gov/geo/query/acc.cgi?acc=GSE46748)
- Modeling computer scripts: GitHub (https://github.com/SysBioChalmers/GECKO/releases/tag/v1.0)
- [data type]: [full name of the resource] [accession number/identifier] ([doi or URL or identifiers.org/DATABASE:ACCESSION])

- All Materials and Methods need to be described in the main text. We would encourage you to use 'Structured Methods', our new Materials and Methods format. According to this format, the Materials and Methods section should include a Reagents and Tools Table (listing key reagents, experimental models, software and relevant equipment and including their sources and relevant identifiers) followed by a Methods and Protocols section in which we encourage the authors to describe their methods using a step-by-step protocol format with bullet points, to facilitate the adoption of the methodologies across labs. More information on how to adhere to this format as well as downloadable templates (.doc or .xls) for the Reagents and Tools Table can be found in our author guidelines:
. An example of a Method paper with Structured Methods can be found here:

- For data quantification: please specify the name of the statistical test used to generate error bars and P values, the number (n) of independent experiments (specify technical or biological replicates) underlying each data point and the test used to calculate p-values in each figure legend. The figure legends should contain a basic description of n, P and the test applied. Graphs must include a description of the bars and the error bars (s.d., s.e.m.).

- The References need to be formatted according to the Molecular Systems Biology reference style.

- When you resubmit your manuscript, please download our CHECKLIST (https://bit.ly/EMBOPressAuthorChecklist) and include the completed form in your submission.
Please note that the Author Checklist will be published alongside the paper as part of the transparent process (https://www.embopress.org/page/journal/17444292/authorguide#transparentprocess).

If you feel you can satisfactorily deal with these points and those listed by the referees, you may wish to submit a revised version of your manuscript. Please attach a covering letter giving details of the way in which you have handled each of the points raised by the referees. A revised manuscript will be once again subject to review and you probably understand that we can give you no guarantee at this stage that the eventual outcome will be favorable.

Maria Polychronidou, PhD
Senior Editor
Molecular Systems Biology

If you do choose to resubmit, please click on the link below to submit the revision online *within 90 days*.

Link Not Available

IMPORTANT: When you send your revision, we will require the following items:
1. the manuscript text in LaTeX, RTF or MS Word format
2. a letter with a detailed description of the changes made in response to the referees. Please specify clearly the exact places in the text (pages and paragraphs) where each change has been made in response to each specific comment given
3. three to four 'bullet points' highlighting the main findings of your study
4. a short 'blurb' text summarizing in two sentences the study (max. 250 characters)
5. a 'thumbnail image' (550px width and max 400px height, Illustrator, PowerPoint or jpeg format), which can be used as 'visual title' for the synopsis section of your paper.
6. Please include an author contributions statement after the Acknowledgements section (see https://www.embopress.org/page/journal/17444292/authorguide)
7. Please complete the CHECKLIST available at (https://bit.ly/EMBOPressAuthorChecklist).
Please note that the Author Checklist will be published alongside the paper as part of the transparent process (https://www.embopress.org/page/journal/17444292/authorguide#transparentprocess).
8. Please note that corresponding authors are required to supply an ORCID ID for their name upon submission of a revised manuscript (EMBO Press signed a joint statement to encourage ORCID adoption).
(https://www.embopress.org/page/journal/17444292/authorguide#editorialprocess)

Currently, our records indicate that there is no ORCID associated with your account.

Please click the link below to provide an ORCID:
Link Not Available

The system will prompt you to fill in your funding and payment information. This will allow Wiley to send you a quote for the article processing charge (APC) in case of acceptance. This quote takes into account any reduction or fee waivers that you may be eligible for. Authors do not need to pay any fees before their manuscript is accepted and transferred to the publisher.

As a matter of course, please make sure that you have correctly followed the instructions for authors as given on the submission website.

*** PLEASE NOTE *** As part of the EMBO Press transparent editorial process initiative (see our Editorial at https://dx.doi.org/10.1038/msb.2010.72), Molecular Systems Biology publishes online a Review Process File with each accepted manuscripts. This file will be published in conjunction with your paper and will include the anonymous referee reports, your point-by-point response and all pertinent correspondence relating to the manuscript. If you do NOT want this File to be published, please inform the editorial office at msb@embo.org within 14 days upon receipt of the present letter.

Reviewer #1:

The authors show how tradeoffs between maximum growth rate and diauxic lag time in environments with two carbon sources

can lead to stable coexistence between pairs of strains, even when one strain has faster maximum growth rate on each carbon source separately. They first demonstrate this result in detail for a pair of soil bacteria on alanine and glutamate. Using detailed growth measurements and mathematical modeling, the authors show that their proposed mechanism is consistent with the observed coexistence between the strains. They then perform a broader survey of additional strains and carbon sources to show that tradeoffs in growth and lag occur are not only common, but also are frequently associated with stable coexistence.

The paper does not really present any molecular or systems biology-type details, and it might be nice if they added some more mechanistic details where possible (see below). But as a microbial ecology and evolution paper, I found it to be excellent: the conclusions are important for many scientists in this field, the work is technically well-executed, and the paper is well-written. Below I discuss a few major scientific points on which the paper could be improved, along with various minor issues.

Major points

1. I think the major area where the paper can improve is a discussion of how exactly coexistence works here, which will be important to theoretical ecologists who have thought deeply about this problem. Specifically, stable coexistence requires negative frequency-dependent selection between the strains. What is it about a growth rate-diauxic lag tradeoff that allows each strain to invade at low frequency? In particular, why is it not just neutral coexistence, as one would naively expect for a tradeoff between two alternating environments?

The authors discuss many more details of the model --- some along these lines --- in the supplement, but this material is poorly integrated in the main text, where there are almost no references to it. I doubt many readers (especially non-theorists) will go through it otherwise. I urge the authors to refer to and summarize the salient points of the supplement throughout the main text where relevant. This is also true for the Materials and Methods section, which is similarly not cited in the main text.

2. My second main question is why the diauxic lag time of Aci2 varies so much with the Ala:Glu supply ratio. I would've expected it not to vary at all, if the lag time is just due to the time it takes cells to shut down one set of metabolic pathways and turn on another (assuming the cells were in steady-state under the first phase). Is it a density-dependent effect? For example, what if the authors vary the initial OD of the population as it switches into glutamate, regardless of how much alanine it grew on before --- is that density sufficient to determine the lag time? Do the authors have any other proposed explanations for this? Have similar phenomena been observed elsewhere?

Furthermore, is this dependence important to the stable coexistence of the strains, or is it an independent issue here? I would guess it is not important to the coexistence, since the coexistence occurs under each Ala:Glu supply ratio separately, but I did not find any discussion of this.

3. Finally, I think the authors could make a greater case for the novelty of their result as a mechanism for coexistence. Much emphasis in microbial ecology is currently on interactions like cross-feeding or antagonism, and I think it is important to emphasize that the growth dynamics themselves, even in the absence of interactions besides resource competition, can lead to rich ecological dynamics.

Minor points

1. Lines 104, 109, 117, 120: These refer to Supp. Fig. 3, but should it be Supp. Fig. 2?

2. Lines 122-123: "That Pa would still briefly stop growing could be due to not expressing enzymes to convert between glutamate and alpha-ketoglutarate prior to resource depletion." The wording here is confusing, perhaps because of the double negative; can the authors express this more clearly?

3. Fig. 2: I would like to see plots (perhaps supplementary) that show more detail on the growth rates during the secondary growth phase of monocultures under Ala+Glu. The authors assume the secondary growth rates equal the initial ones, and Fig. 2C,E makes this sound plausible, but the dynamic range of the color bar is too large to really tell.

4. Fig. 2 caption: "Lag times could not be fit in the with too little growth" --- some words appear to be missing here.

5. Fig. 3 caption: "See Supp. Fig. 4 for comparison." Should this be Supp. Fig. 3?

6. Fig. 3 caption and Supp. Fig. 4: Why did the authors decide on a quadratic time-dependence of growth rates after the switch? Do they have any proposed mechanism for this?

7. Figs. 3 and 4: I found all the figures to be rather busy, but these two in particular had a lot of tiny details that may be hard for some readers to glean, especially Figs. 3A an 4A. Can the authors simplify these?

8. Lines 221-222: The authors say cross-feeding is a likely explanation for the unexpected coexistence at extreme Ala:Glu ratios. But what about other possibilities? Maybe there are tradeoffs in other growth traits (e.g., death or survival during stationary

phase), or history-dependent effects (e.g., growth traits change during subsequent cycles of growth, which cannot be observed in single monoculture growth curves).

9. Lines 329-341: I don't entirely agree with the authors' characterization of initial vs. diauxic lags. From the perspective of microbial physiology, I would argue they are categorically the same: both represent transitions between growth states (the former just being a transition from zero growth to non-zero growth). However, if they lead to qualitatively different consequences for ecology, then that is the important point to make here. But if that is the case, what is the fundamental difference based on the growth dynamics?

10. Suppl. Fig. 1: Can the authors please also add a legend to identify the mapping between species and colors?

11. Suppl. Fig. 3: This figure also needs a legend to distinguish data vs. fits.

12. Suppl. Fig. 4: It is hard to identify small differences in growth rates from the growth curves themselves. Why not plot inferred growth rate vs. Ala:Glu?

13. Suppl. Fig. 6: In the rightmost panel, why is Pa fraction not just zero, since it has slower growth rate in all single-resource conditions? Is it because they are measuring its frequency after finite time (7 days)?

14. Suppl. Fig. 7: What is the correlation coefficient? It might be helpful to also plot this on a log-log plot (in addition to the existing linear-linear plot) to resolve the low-frequency variation.

15. Suppl. Fig. 9 caption: "with one generalized cross-feeding product excreted from each species." Is this missing the word "each"?

16. Suppl. Fig. 9 caption: Here the authors describe a model for cross-feeding during stationary phase, but the details seems strange to me. How did they choose these details?

Reviewer #2:

In this report, Bloxham et al address the question of coexistence of species in the presence of two resources. In a specific example where Pseudomonas aurantiaca (Pa) loses in head-on competition against Acinetobacter sp (Aci2) on both alanine and glutamate independently, authors found that both species coexist when both the resources were added together. By analyzing the monoculture growth patterns in two-resource condition, they figured that Pa's shorter diauxic lag might be responsible for the coexistence with Aci2. They built a simple model based on growth rate and diauxic lag to successfully explain the phenomenon, made predictions for few more head-on competitions involving a bunch of more bacterial species and different carbon sources, and verified them experimentally. It is a useful work that sheds light on an important aspect of microbial ecology.
The importance of lag time in dictating fitness effects and relative populations of binary competitors in a single resource environment have been previously demonstrated in both experiments (Adkar, et al, Nat Ecol Evol, 2017) and theory (Manhart et al, RSB, 2018, Nat comm, 2018). It was also explicitly shown that fitness benefits of lag times are best realized under conditions of nutrient limitations. It is not unrealistic to expect that diauxic lags would be an important factor in determining fitness effects (coexistence) in two resource conditions. This idea is nicely demonstrated in this article.
I have a several major methodological concerns and are listed below, not in any order of priority:
1. It is not clear how the diauxic lag times are estimated from experimental data. It is not clearly defined anywhere in the text. Supplementary Fig 43 shows fits for the exponential phases and lag times, but it was not clear how the fits for lag times were done. Was the equation defined in 'Modeling' section used to derive the lag time here? This is a crucial information, as most of paper relies on this.
2. The lag time fits in Supplementary Fig 3 are confusing and seems that the lag times extend until saturation. In that case, lag times for Pa extend beyond 1 hr. I am sure I am not reading it right, but more explanation will help here.
3. It is not clear how you get 12h diauxic lag time for 2:1 A:G for Aci2 as shown in Supplementary Fig 3A. One way I can think of is to fix g_mu,0, and extrapolate time based on the quadratic growth recovery equation as defined in the 'Modeling' section to achieve that growth rate. However, it is not clear if that how it is done. Moreover, it is unclear what is g_mu,0. Is it the growth rate for the second phase?
4. If the lag times are derived based on the assumption of the model, i.e., the pre- and post-depletion growth rates are equal, then it may artificially overestimate diauxic lags. I am not suggesting that the diauxic lags may not vary, but in this case, it is important to derive lag times for the second transition without any constraints to make sure the variation is not an artifact. Moreover, I did not find any explanation in the manuscript justifying the assumption regarding growth rates. In fact, from most of the experimental data, it is clear that both phases do not have equal growth rates and the second growth rate is lower than the first one. This assumption of equal rates would introduce quite a bit of error in estimation of proportion of the species. However, it is surprising that despite this, the model predictions match the experimental outcomes in many cases. It will be useful if authors comment on this. I think a model which explicitly makes use of both experimental values of growth rates and lag times would be more appropriate here.
5. Two different methods of growth rate estimations are described on lines 391-393 and 421-425. It is not clear which one is

used? The later one, as described, may likely yield growth rates lower than maximum growth rates.
6. The fits in Supplementary Fig 43 don't always look reliable. It will be good to have some kind of goodness of fit estimates.
7. The slow growers that are slow-switchers should ideally always lose in competition. It is surprising that in Fig 6, there are few such strains that either coexist or even win the competition. Some insights into this will be helpful.
8. Authors should comment on if acidic carbon sources change the pH of growth media (M9), and also if it affects bacterial growth profiles.
9. Are the colonies for all different bacteria morphologically different to be distinguishable in a competition experiment? If yes, it will be good to mention this, and if possible, a photograph of plate with different bacterial colonies may be helpful.

Reviewer #3:

Microbes often co-exist as complex mixed populations. However, the establishment and maintenance of such co-existence is not always trivial, and many of the underlying mechanisms and evolutionary forces remain elusive. This study examines how coexistence is established in multi-resource environments. A clever set of experiments reveal that overall fitness of individual species in a mixture is influenced by both the growth rate in stable conditions as well as the lag times during environmental shifts. A mathematical model further formalizes this effect and shows that the tradeoffs can help support coexistence. A more complex model that also accounts for metabolic strategies further generalizes the findings.

As such, this study furthers our general understanding of microbial ecology by offering a mathematical framework to model, understand and predict the development and evolution of complex microbial communities, with a specific emphasis on the effects of fitness in stable environments vs the speed at which cells adapt to environmental changes (specialist vs generalist startegies).

While I generally like study very much, I do have a few questions and recommendations.

Minor comments:

1. In the methods section it is not specified with which machine the OD is measured and what the detection level or the linear range is of the machine. How was the raw data is processed, for example for the OD measurements, was there a correction for the background?
2. Figure 2, for A, C & E the ranges of the x-axis are not consistent. For figure 2E and figure3C there x-axis range differs which is not addressed in the text. It might be important to highlight that when the diauxic lag occurs for Pa is different for the model compared to the experimental data.
3. Top figure of 2A only the first 8 hours are shown. Is there a specific reason why the growth rates are calculated before 8 hours and an OD below 0.1? is this OD above the detection level of the machine or above the background level? Because if you look at the bottom figure of 2A for the full growth curves (here the x-axis goes from 6 to 18 hours) it seems that the maximal growth rate reached is higher for Pa then for Aci2.
4. Figure 2E and 2F, the diauxic lag time decreases the closer you get to the resource ratio of 2:3 alanine:glutamate. It could be interesting to model this decrease in lag rather than taking a constant lag duration. If you would do that I think figure 3C might resemble 2E even better since the saturation will be reached sooner the closer the ratio is to 2:3. It also seems from figure 2E the closer the resource ratio becomes to 2:3 the less the actual growth rate decreases.
5. Figure 5B & C, would it be useful to also assess the quality of the model by comparing the prediction of the composition after 7 days to the experimental data instead of using the stable state prediction? Still show the stable state prediction since that on itself is interesting.
6. For supplementary figure 7, where you show the 'observed vs predicted' is the predicted again the stable state prediction? It is also not clear how many points are around the origin. It might be good to show a residual plot, this will show for each combination the error of the prediction separately.
7. From supplementary figure 7 it also seems that the model is good at predicting exclusions but performs less well at predicting the coexistences. It might also be interesting to give the the actual correlation value in addition to the p-value.
8. Line 203: it is stated that you predict 32 coexistence out of 54, but they do not always agree with the experimental data. This merits more discussion I think.
9. Line 335: I think it could be useful to incorporate the initial lag, since Pa really seems to be growing a lot slower in the beginning, and eventually catches up. Also, in figure 2B and 2D you could see a difference in their initial lag phase +- 4hours. But I think this depends on how you define the lag phase. I would be a bit clearer on how you define the lag phases .
10. Is it necessary to square the (t-tdep)/tlag,µ since how I understand it this part is only activated when the resources are depleted and tdep will always be smaller or equal to t. So you would never have the risk of having a negative value. Or is there another reason to square it, eg a better fit?

Typo's etc

1. line 23 missing 'which' before 'must' the which refers to the dynamics

2. line 411 What does DF mean? I Found it o, the supplementary that it is the dilution factor it might be good to quickly state the abbreviation in the main text.
3. Figure 2B and D and related supplements could use some adaptation to better get the main message across and omit clutter... What I think the main takeaway is from that figure, is that the OD at which the diauxic lag occurs. It might be good to include or switch it with the supplementary figure 2. The supplementary figure is a very nice figure to show the trends at which OD's the diauxic lag occurs for the different ratios.
4. Not all the colors of the curves in supplementary figure 4 match with the legend colors.
5. Figure 3A ii. Would it be a good idea to keep the inequality signs in the same direction? This is a bit easier for the reader to see immediately that the exponential growth for Aci2 is larger but that it also means that the diauxic lag for the Aci2 is longer.
6. Figure 3D is missing '(OD) or log(OD)' also in the simulations the populations grow to much higher densities than in the experiments which have an OD of around 0.4. Would it be nice to overlay the simulations with experimental data?
7. Line 217: At lower dilution fraction does the total population grow to higher OD's or do they stay longer at the carrying capacity? And how does this effect the composition/behavior?
8. Line 270: In the example with Aci2 becoming the slow grower and fast switcher, is this caused by the other species presence or the other available resources?
9. Line 313: I like the idea of the crossfeeding, but why is it not highlighted in the results of the main text as a possible mechanisms to predict the coexistence at low dilutions?
10. Line 390: Measuring OD at 400nm or 600nm but it is not always stated when one is used over the other? Also, this is not indicated on the axis of the figures.

Reviewer #1:

The authors show how tradeoffs between maximum growth rate and diauxic lag time in environments with two carbon sources can lead to stable coexistence between pairs of strains, even when one strain has faster maximum growth rate on each carbon source separately. They first demonstrate this result in detail for a pair of soil bacteria on alanine and glutamate. Using detailed growth measurements and mathematical modeling, the authors show that their proposed mechanism is consistent with the observed coexistence between the strains. They then perform a broader survey of additional strains and carbon sources to show that tradeoffs in growth and lag occur are not only common, but also are frequently associated with stable coexistence.

The paper does not really present any molecular or systems biology-type details, and it might be nice if they added some more mechanistic details where possible (see below). But as a microbial ecology and evolution paper, I found it to be excellent: the conclusions are important for many scientists in this field, the work is technically well-executed, and the paper is well-written. Below I discuss a few major scientific points on which the paper could be improved, along with various minor issues.

We thank the reviewer for the positive summary of our work and especially for their high opinion of its novelty. We found the reviewer's feedback to be consistently insightful and constructive, and we thank them for that input. In particular, we believe the added discussion of why coexistence is stable, the deeper exploration of the variations in Aci2's lag time, clarification of why we used a quadratic growth rate recovery shape, and the restructuring of the Appendix into a comparison of initial and diauxic lags have each greatly strengthened our paper, and we are very glad to have been asked to make these changes and additions.

Major points

1. I think the major area where the paper can improve is a discussion of how exactly coexistence works here, which will be important to theoretical ecologists who have thought deeply about this problem. Specifically, stable coexistence requires negative frequency-dependent selection between the strains. What is it about a growth rate-diauxic lag tradeoff that allows each strain to invade at low frequency? In particular, why is it not just neutral coexistence, as one would naively expect for a tradeoff between two alternating environments?

We thank the reviewer for encouraging us to be more explicit regarding how diauxie leads to negative frequency dependence. Coexistence is stable due to feedback between the species' population fractions and the resource depletion times. Pa benefits from an early alanine depletion (so Aci2's lag starts sooner) and a later glutamate depletion (so it has longer to grow between the start of Aci2's lag and saturation). But, because Pa grows slower than Aci2, increasing Pa's fraction results in a later alanine depletion and an earlier glutamate depletion. This feedback creates the negative frequency-dependent selection. To include this discussion in the paper, we have added two additional subplots to Figure 4 and additional text in the results section (both provided below).

The corresponding portion of the Results (line #203 in subsection "Tradeoff between growth rate and lag time is sufficient for coexistence") now reads as:

> " Our model successfully reproduced stable coexistence of Aci2 and Pa on alanine and glutamate with the same steady state being reached regardless of initial starting fractions (Fig 4B). At steady state, population dynamics were driven by diauxic resource consumption, with two distinct periods of growth causing population fractions to vary considerably over the course of a day. Aci2 initially grew faster, causing Pa's population fraction to decrease significantly over the first ~10 hours until alanine was depleted. After alanine depletion, Pa's growth rate quickly recovered while Aci2 suffered a long lag, allowing Pa to catch back (Fig 4C). The balance of these two growth phases allowed the model to reproduce coexistence of the two species. "

> " Coexistence was stable due to negative frequency-dependent selection mediated by changes to the resource depletion times. If Pa's population fraction were increased above its steady-state (and Aci2's correspondingly decreased), alanine would be depleted later and glutamate sooner (Fig 4D) because the population would overall be consuming glutamate faster and alanine slower. A later alanine depletion and earlier glutamate depletion would both lengthen the period of Aci2's initial fast growth and shorten the period after alanine depletion during which Pa could catch up. An increase in Pa's population fraction would therefore affect the resource depletion times in ways that decrease Pa's fitness relative to Aci2 (Fig 4E). Similarly, a decrease in Pa's population fraction would increase its relative fitness through an opposite set of effects. Thus, feedback between population fractions and resource depletion times created negative frequency-dependent selection and stable coexistence. "

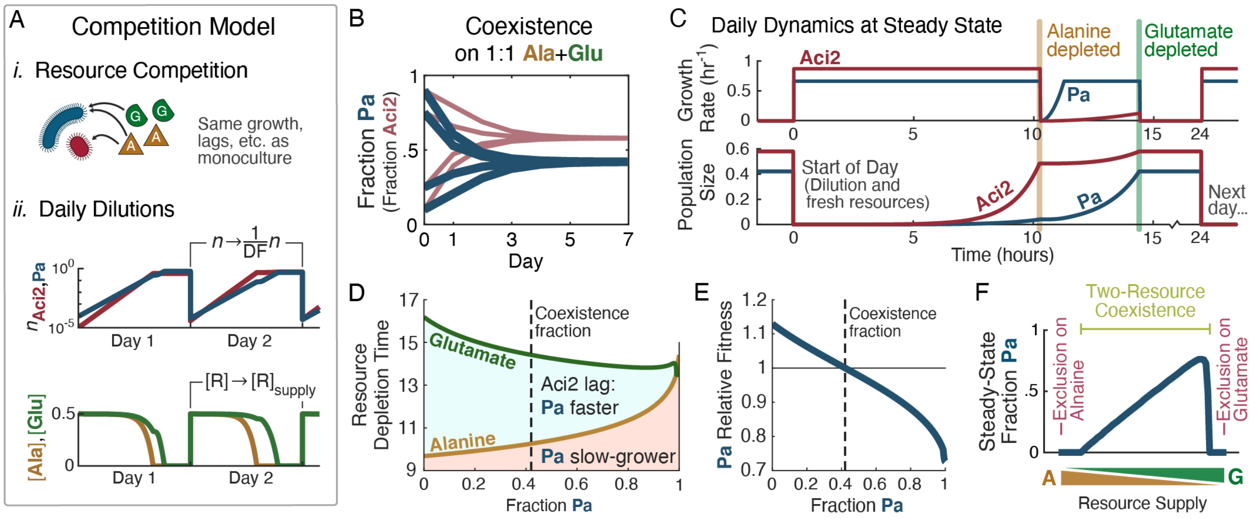

Figure 4. Growth-lag model uses the tradeoff between Aci2's fast growth and Pa's short lag to explain two-resource coexistence.

A. The competition model is an extension of the monoculture model. (*i.*) Species have the same growth, lags, and resource consumption as in monocultures, but now compete for the same resource pool. (*ii.*)

After saturation, population sizes are divided by the dilution factor (10^4 in this figure) and resource concentrations are reset to the supply concentrations. (Modeling details in Materials and Methods.)

B. With parameters matching those used experimentally in Fig 1C, the growth-lag model predicts coexistence of Aci2 and Pa on alanine and glutamate, as observed in competition experiments.

C. Model prediction of growth rates and population sizes over the course of one day after the 1:1 alanine:glutamate co-culture has reached steady state. Pa starts the day with a population fraction of 0.42, declines to a population fraction of 0.07 at alanine depletion, and recovers to a population fraction of 0.42 by the time glutamate is depleted.

D. The alanine and glutamate resource depletion times vary with the (start-of-day) population fractions due to different resource preferences and initial growth rates.

E. These variations modulate the relative fitness of the two strains and in doing so facilitate a negative-frequency dependent interaction that stabilizes coexistence. Relative Fitness of Pa is calculated as its fold growth over the course of a day divided by Aci2's fold growth (Materials and Methods), and shown as a function of its population fraction at the start of the day.

F. The model predicts the competitive outcome will depend on the resource supply fractions with exclusion occurring as the supply approaches entirely alanine or entirely glutamate.

- The authors discuss many more details of the model --- some along these lines --- in the supplement, but this material is poorly integrated in the main text, where there are almost no references to it. I doubt many readers (especially non-theorists) will go through it otherwise. I urge the authors to refer to and summarize the salient points of the supplement throughout the main text where relevant. This is also true for the Materials and Methods section, which is similarly not cited in the main text.

  We recognize that the Appendix (previously Supplement) contains significant extensions beyond the core conclusions discussed in the main text, and we are glad that the reviewer feels that these results are sufficiently interesting to warrant more highlighting in the main text. We have made significant organizational changes to the Appendix and better referenced it throughout the Main Text. With the reviewer's Minor Point #9 in mind, the detailed modeling section has now been refocused as a comparison of the ability of initial and diauxic lags to produce stable coexistence between species. Main points from this comparison include (i) with all yields equal diauxic lags allow two species to coexist but initial lags do not, (ii) with variable yields and a density-dependent dilution factor initial lags allow two species to coexist while diauxic allow three species to coexist, and (iii) with randomly sampled species coexistence is more likely when lags are diauxic rather than initial. We thank the reviewer for encouraging this reorganization and believe many more interested readers will benefit from the material as a result.

  The Materials and Methods section has been rewritten to reflect various points of feedback from all three reviewers, reorganized with better section titles to make information easier to find, and cited throughout the Main Text.

2. My second main question is why the diauxic lag time of Aci2 varies so much with the Ala:Glu supply ratio. I would've expected it not to vary at all, if the lag time is just due to the time it takes cells to shut down one set of metabolic pathways and turn on another (assuming the cells were in steady-state under the first phase). Is it a density-dependent effect? For example, what if the authors vary the initial OD of the population as it switches into glutamate, regardless of how much alanine it grew on before --- is that density sufficient to determine the lag time? Do the authors

have any other proposed explanations for this? Have similar phenomena been observed elsewhere?

We thank the reviewer for raising this question as we greatly enjoyed the exploration it prompted. The biochemical origins of lag times and their variations are not the central foci of this paper, so we have refrained from dedicating too much space to exploring these questions. Understanding why Aci2's lag time would vary so significantly is, however, an interesting question on its own, and ensuring that we modeled those variations in an appropriate manner was an important check that we are glad to have performed.

In summary:

i. We performed a large set of additional monoculture lag time experiments in which we varied multiple environmental parameters. We concluded that Aci2's lag does not vary with the dilution factor nor directly with the time it spends growing before alanine depletion, but we could not determine whether Aci2's lag time is best captured as varying with the alanine supply concentration or with its population size at the onset of its diauxic shift.
ii. The original modeling was consistent with Aci2's lag being a function of the alanine supply concentration and is still the modeling presented in the Main Text. Alternative modeling in which Aci2's lag time is a function of its population size at the onset of its diauxic shift is now presented in Extended View Figure EV4, with the results being very similar.
iii. Solopova 2014 ("Bet-hedging during bacterial diauxic shift") saw similar variations in diauxic lag times and concluded the variations were likely due to larger population sizes having faster resource consumption rates and depleting resources more suddenly with less time for cells to begin expressing their second-resource metabolism before the shift. We don't have direct evidence that this mechanism is applicable here, but it fits our observations and is easily plausible. If this is the relevant mechanism, the Main Text modeling of lag time being a function of alanine supply is a close approximation.

The new monoculture lag time experiments involved varying total resource supply as well as the resource supply ratio so that alanine supply concentration and glutamate supply concentration could be decoupled. Appendix Figure S8A (below) shows the alanine and glutamate supply combinations that were tested. We also used three initial dilution factors from the overnight starter culture: 10^3, 10^4, and 10^5. Each supply concentration combination was tested for each initial dilution factor. Lag times were then extracted in the same manner as in the original monoculture lag time experiments. When plotting Aci2's diauxic lag time against each of the experimentally controlled parameters (alanine supply, glutamate supply, and initial dilution), we saw Aci2's lag time correlated strongly with the alanine supply but not with the glutamate supply nor with the initial dilution (Appendix Figure S8B). We additionally compared Aci2's lag time to the time and population size at the onset of its diauxic shift (Appendix Figure S8C). We saw a strong correlation between Aci2's lag time and its population size at the onset of its diauxic shift but not between lag time and the time spent growing on alanine before the diauxic shift. We thus concluded that Aci2's lag time did not vary with the dilution factor, but whether Aci2's lag time was really varying with the alanine supply or with Aci2's population size at the onset of its diauxic shift remained unclear.

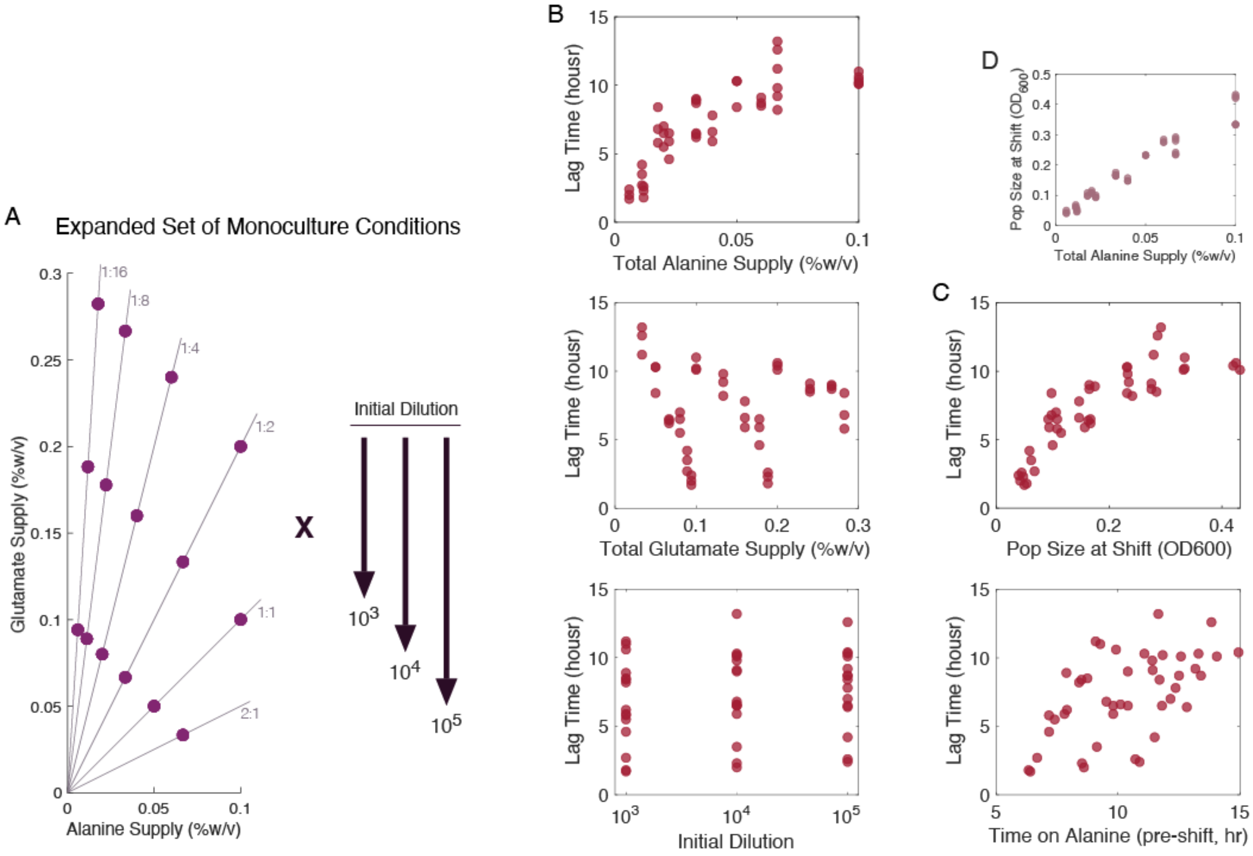

Appendix Figure S8. Aci2's lag times do not vary with initial dilution or time spent growing pre-shift, but may be a function of the alanine supply or of Aci2's population size at the onset of its diauxic shift. **(A)** To further investigate with which parameters Aci2's lag time truly varies we repeated the monoculture lag time measurements (Fig 2B-C) at an expanded set of conditions, defined by resource supply ratios of 1:16 through 2:1 Ala:Glu at 1x, 2x, and 3x the total resource concentrations used in the Main Text and initial dilutions of 10^3, 10^4, and 10^5 from an overnight starter culture. The plot indicates the alanine and glutamate supply combinations used. All 15 supplies were tested at each of the three dilutions. **(B)** Extracted lag times vs each of the three directly controlled parameters. Lag time appears to correlate well with alanine supply and notably has no variation with initial dilution. That lag time does not vary with initial dilution justifies modeling Aci2's lag time as a function of resource supply but not dilution factor. **(C)** Extracted lag times vs time and population size at the onset of the diauxic shift. Aci2's lag time correlates well with its population size at the onset of its diauxic shift but not with time spent growing on alanine before its diauxic shift. **(D)** Aci2's lag time correlates equally well to the alanine supply and to its population size at the onset of the diauxic shift because in monoculture the alanine supply determines Aci2's population size at the onset of its diauxic shift (at which point Aci2 has consumed all the alanine and nothing else). This correlation meant we could not rule out that Aci2's lag time might truly be a function of its population size at the onset of its diauxic shift. For simplicity the Main Text modeling assumes Aci2's lag time varies with the experimentally controlled parameter. Fig EV4 shows the results of instead modeling Aci2's lag time as varying with its population size at the onset of its diauxic shift.

To disentangle the alanine supply and Aci2's population size at the onset of the diauxic shift, we attempted a set of experiments in which we diluted the monoculture into a glutamate-only media either just before or just after Aci2's diauxic shift. We struggled, however, to obtain interpretable results from these experiments, in part due to condensation artifacts that arose from moving plates in and out of our OD reader during the period of interest. As this exploration was interesting but

ultimately tangential to the paper, we decided to not invest our time in an iterative debugging of the dilute-during-shift experiments and to instead present modeling corresponding to each of the two possibilities.

Modeling Aci2's lag time as a function of alanine supply is consistent with the Main Text modeling (resource supply ratio and alanine supply concentration are directly related because the total resource supply was held constant). Modeling Aci2's lag time as dependent on its population density at the onset of its diauxic shift is briefly referenced as an option in the Main Text with results presented in the Extended View:

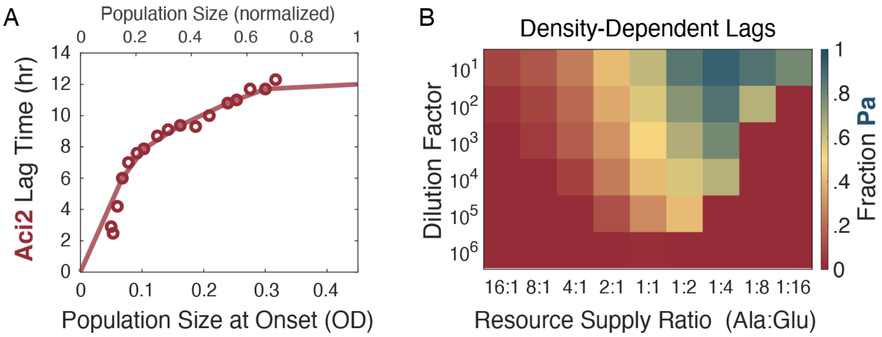

Figure EV4. Modeling Aci2's lags as dependent on its population density at the onset of its diauxic shift (instead of initial alanine supply) prevents Pa from excluding Aci2.

A. Using the lag times presented in Fig 2H and the population sizes at the onset of Aci2's diauxic shift presented in Fig 2C allows Aci2's lag time to be alternatively expressed as a function of its population size at the onset of its diauxic shift. Circles represent lag time data and the line defines the piecewise linear function used for modeling. Whether Aci2's lag is most accurately modeled as a function of alanine supply or of population size at the time of its diauxic shift could not be definitively determined (Appendix Fig S8).
B. Steady-state phase space for the Aci2 vs Pa on alanine and glutamate competition at various resource supply ratios and dilution factors when modeling Aci2's lag as a function of its population size at the onset of the diauxic shift. The most significant change relative to the original prediction (Fig 5B) is that Pa never fully excludes Aci2. Pa cannot exclude Aci2 because as Aci2 is driven extinct its population size at its diauxic shift as well as its diauxic lag time converge to zero, eliminating the period when Pa can catch up to Aci2.

We did not engage in any further study of how and why Aci2's lag time varies. Similar phenomena have, however, been observed and studied by other researchers. For example, Solopova 2014 ("Bet-hedging during bacterial diauxic shift") saw that when *Lactococcus lactis* switches from glucose to cellobiose the length of its diauxic lag depends on, and increases with, the supply concentration of glucose – very similar to how Aci2's lag time depends on and increases with the alanine supply concentration. Solopova noted that a larger population as the diauxic shift is neared means the preferred resource is being depleted faster, essentially giving the cells less time to prepare.[1] Venturelli 2015 ("Population Diversification in Yeast") similarly showed that what

[1] Because cells hit a starvation state when the first resource is depleted, there is a significant advantage to begin expressing the second-resource metabolism before the resource depletion when cells still have energy and carbon influx to work with. If expression of the second-resource metabolism is triggered when the concentration of the first resource decreases below a key threshold, a larger population and faster resource consumption rate means there is less time between crossing this threshold and the depletion of the first resource. For example, a population twice as large consumes the resources that are remaining when the threshold is crossed in half the time.

fraction of a *Saccharomyces cerevisiae* population begins to prepare ahead of a shift from glucose to galactose can vary with the resource supply concentrations, while Wang 2015 ("Natural Variation in Preparation for Nutrient Depletion") demonstrated the impact of this preparatory period on diauxic lag time and strain fitness. These previous studies provide possible explanations for why Aci2's lag would vary in the way that it does. We have not, however, engaged in any direct mechanistic study of our own.

If we were to assume Solopova's explanation that the rate of resource consumption just before depletion can modulate lag time, the Main Text modeling of Aci2's lag time being a function of the alanine supply (rather than its population size) is a very appropriate choice. This is because Pa also contributes to the alanine depletion rate so the rate of alanine consumption is highly correlated with the total alanine consumed.[2]

To address these points in the paper, Appendix Figure S8 and Figure EV4 have been added along with brief discussion and references to these at figures at a couple points in the manuscript:

- In the Results (line #142 in subsection "Aci2 is the fast-grower, but Pa is the fast-switcher"):

  > " When mostly glutamate was supplied Aci2's lag time was the shortest (~2 hours), and as the supply of alanine increased Aci2's lag time became longer (up to ~12 hours) (Fig 2D, 2H and EV2). We observed no variation with initial population size but note that Aci2's lag time could be a function of either resource supply or its population size at the onset of its diauxic shift (Appendix Fig S8; Materials and Methods). Examples of variable diauxic lag times have been studied (Wang et al, 2015; Venturelli et al, 2015; Vermeersch et al, 2019; Solopova et al, 2014). One particularly similar example concluded that larger supplies of the preferred resource and larger population sizes just before the resource depletion increased the overall rate of resource consumption and suddenness of the depletion, giving the population less time to prepare and a longer diauxic lag (Solopova et al, 2014). This explanation could be applicable here, but we did not investigate further, as our focus was on the ecological consequences, not the origins, of the species' diauxic lags. "

- In the Materials and Methods (line #653 in subsection "Lag Time Measurements"):

---

[2] If the supply of alanine is s_{Ala} and species are modeled with yields equal to 1, then at the alanine depletion time, $t_{\text{dep,Ala}}$, all of the alanine must have become Aci2 or Pa biomass, $s_{\text{Ala}} = n_{\text{Aci2}}(t_{\text{dep,Ala}}) + \frac{2}{5} n_{\text{Pa}}(t_{\text{dep,Ala}})$, with the $\frac{2}{5}$ arising because the other $\frac{3}{5}$ of Pa's biomass comes from glutamate. The concentration of alanine is therefore decreasing at rate $\frac{dc_{\text{Ala}}}{dt} = -(0.88\ \text{hr}^{-1})\ n_{\text{Aci2}} - \frac{2}{5}(0.67\ \text{hr}^{-1})\ n_{\text{Pa}}$ just before the resource depletion. Rearranging, one can obtain $\frac{dc_{\text{Ala}}}{dt} = -\left(\frac{1.47 - 1.02 f_{\text{Pa}}}{1.67 - f_{\text{Pa}}}\right) s_{\text{Ala}}$, with the term $\frac{1.47 - 1.02 f_{\text{Pa}}}{1.67 - f_{\text{Pa}}}$ varying from 0.88 hr^{-1} at $f_{\text{Pa}} = 0$ to 0.67 hr^{-1} at $f_{\text{Pa}} = 1$ (with f_{Pa} being $f_{\text{Pa}}(t_{\text{dep,Ala}})$ in this context). The limited impact of f_{Pa} on $\frac{dc_{\text{Ala}}}{dt}$ means that to decent approximation $\frac{dc_{\text{Ala}}}{dt} \propto s_{\text{Ala}}$ and Aci2's lag time can be modeled as a function of s_{Ala} if it is assumed to truly be a function of $\frac{dc_{\text{Ala}}}{dt}$. Alternatively, one could rearrange to get $\frac{dc_{\text{Ala}}}{dt} = -0.88\ \text{hr}^{-1}\ (0.76\ s_{\text{Ala}} + 0.24\ n_{\text{Aci2}})$, which further illustrates how the diauxic lag time should be expected to be more sensitive to the alanine supply than Aci2's specific population size.

“ Lag times were fit using a growth rate recovery shape in which growth rate recovered proportional to the square of the time since the resource depletion,

$$g(t) = g_{SS}\left(\frac{t - t_0}{t_{lag}}\right)^2 \quad \text{if} \quad t < t_{lag}\,, \quad \text{else} \quad g(t) = g_{SS}\,,$$

where g_{SS} is the steady-state growth rate, t_0 is the resource depletion time, and t_{lag} is the species lag time. This recovery shape was used because it was a single-parameter fit that was a close empirical match to post-shift recoveries observed in the monoculture data, particularly in comparison to other recovery shapes considered (Appendix Fig S2). In the Main Text, lag times are reported as a function of resource supply ratio. Lag times were also fit for different initial dilution factors but no variation was observed (Appendix Fig S8). ”

- In the Materials and Methods (line #717 in subsection “Modeling”):

“ From monoculture experiments, it could not be determined whether Aci2’s lag time was a function of the alanine supply concentration (equivalent to being a function of resource supply ratio with the total concentration constant) or of its population size at the onset of its diauxic shift (Appendix Fig S8). Modeling predictions if Aci2’s lag time were a function of its population size were also determined and seen to be similar (Fig EV4). Other researchers have suggested variable lag times may be the result of larger populations consuming resources more quickly and having less time to prepare for the switch to the next resource (Solopova 2014). If this is the case here, lag times would be approximate functions of the resource supply ratio because both Aci2 and Pa contribute to the coculture-wide resource consumption rate and the total population size at alanine depletion is closely linked to the alanine supply concentration. ”

- Furthermore, is this dependence important to the stable coexistence of the strains, or is it an independent issue here? I would guess it is not important to the coexistence, since the coexistence occurs under each Ala:Glu supply ratio separately, but I did not find any discussion of this.

We thank the reviewer for asking this question as we realized the answer was not provided in the main text. For the most part, that Aci2’s lag time varies is not importance for stable coexistence. For example, if we use a constant value of 8 hours for Aci2’s lag, three quarters (24 out of 32) of the conditions in which coexistence was predicted in the model still have coexistence predicted. Meanwhile three conditions flip in the opposite direction from exclusion having been predicted in the original model to coexistence now being predicted in the constant-lag model.

These results are now provided in Fig EV3B, which is briefly referenced in the Results (line #270 in subsection “Accurate prediction of response to environmental changes validates growth-lag model”):

“ Reducing the model to an even simpler form, it was observed that many of the key features were preserved even when Aci2 was assigned a constant lag time (Fig EV3B). ”

Figure EV3B contains the above-mentioned modeling results, while EV3A is provided in response to a comment from another reviewer.

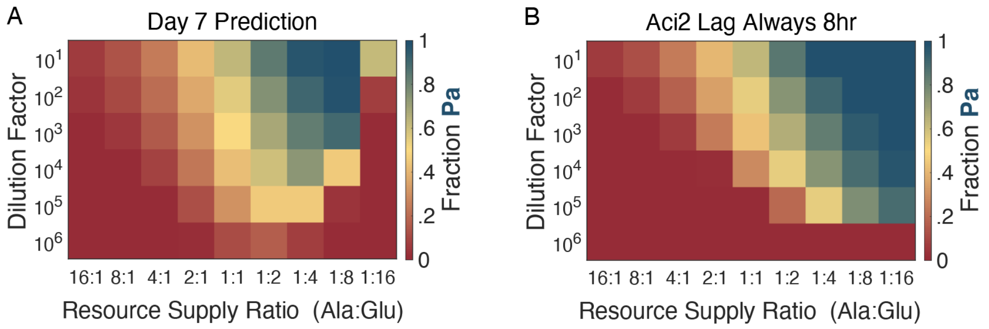

Figure EV3. Predicted mean fraction at Day 7 and predicted steady-state if Aci2's lag did not vary with resource supply.

A. Predicted mean fraction Pa after seven dilution cycles of competition. Shown is the average fraction Pa from competitions started with a Pa fraction of 0.1, 0.25, 0.75, and 0.9 (same as in experiments).
B. Predicted steady-state fraction Pa if Aci2's lag did not vary with resource supply but was instead constant at 8 hours. The value of 8 hours was chosen for being close to Aci2's mean lag time of 9±1 hours and providing a close fit to the experimental results.

3. Finally, I think the authors could make a greater case for the novelty of their result as a mechanism for coexistence. Much emphasis in microbial ecology is currently on interactions like cross-feeding or antagonism, and I think it is important to emphasize that the growth dynamics themselves, even in the absence of interactions besides resource competition, can lead to rich ecological dynamics.

We thank for reviewer for their high opinion of our paper's novelty. We have strengthened language and claims about our results in a few locations:

- The last sentence of our abstract (line #27) now reads as:

  “ Our work illustrates a simple mechanism, based entirely on supplied-resource growth dynamics, for the emergence of multi-resource coexistence. ”

- The end of our introduction (line #87) now reads as:

  “ Our research establishes a mechanism for the otherwise unexpected coexistence of species based entirely on growth dynamics on supplied resources and simple tradeoffs between growth and diauxic lag and highlights the importance of diauxic lags on interspecies competition. ”

- In the Results section when first talking about our modeling decision (line #163 in subsection “Simple growth-lag model reproduces monoculture dynamics”) we now explicit say:

  “ No additional complexities were included in order to have a minimal model with which we could later test the ability of a growth-lag tradeoff to explain coexistence

and predict qualitative trends in community assembly without the need to invoke other, more complicated interactions. ”

- In the Discussion after talking about possible intersections between diauxie and cross-feeding (line #364) we now remind the reader:

  “ But, that diauxic lags alone can produce stable coexistence shouldn’t be overlooked as our results highlight how simple models that don’t attempt to capture every detail can still explain and predict surprising results in community assembly. ”

Minor points

1. Lines 104, 109, 117, 120: These refer to Supp. Fig. 3, but should it be Supp. Fig. 2?

   We thank the reviewer for a sufficiently detailed read so as to catch this error. As we have reorganized the Supplement into the Extended View and Appendix, we have paid careful attention to the numbering of figures.

2. Lines 122-123: "That Pa would still briefly stop growing could be due to not expressing enzymes to convert between glutamate and alpha-ketoglutarate prior to resource depletion." The wording here is confusing, perhaps because of the double negative; can the authors express this more clearly?

   As this sentence was pure conjecture and inherently vague, we have decided to simply remove it.

3. Fig. 2: I would like to see plots (perhaps supplementary) that show more detail on the growth rates during the secondary growth phase of monocultures under Ala+Glu. The authors assume the secondary growth rates equal the initial ones, and Fig. 2C,E makes this sound plausible, but the dynamic range of the color bar is too large to really tell.

   We have added Figure EV1 detailing growth rates in single-resource environments and Appendix Figures S5 and S6 showing clearer information on the post-shift growth in two-resource experiments. The species grow 15-25% slower in the single-resource experiments, and the post-shift growth shows the species reaching approximately the anticipated single-resource growth rates when they have sufficient recovery time between the onset of the diauxic shift and saturation. For example, in the three conditions with the highest fraction glutamate and the most post-shift growth, Aci2 reached peaked growth rates of 0.71 hr^{-1}, 0.76 hr^{-1}, and 0.69 hr^{-1} (compared to an expected value of 0.74 hr^{-1}).

   The specific single-resource growth rates are: for Aci2, 0.68 hr^{-1} on alanine and 0.74 hr^{-1} on glutamate (compared to 0.88 hr^{-1} on both), and, for Pa, 0.5 hr^{-1} on alanine and 0.57 hr^{-1} on glutamate (compared to 0.67 hr^{-1} on both). Modeling with separate single-resource growth rates had little impact on the modeling predictions (largely because the post-shift switch would most frequently be to glutamate and the glutamate growth rates are only 16% slower for Aci2 and 15%

slower for Pa). For simplicity and to have the only tradeoff in the modeling be growth rate vs diauxic lag time (and not also an initial vs post-shift growth rate tradeoff), we decided to model with only a single growth rate for each species.

4. Fig. 2 caption: "Lag times could not be fit in the with too little growth" --- some words appear to be missing here.

   This has been corrected to "Lag times could not be fit for conditions with too little growth on the remaining glutamate.".

5. Fig. 3 caption: "See Supp. Fig. 4 for comparison." Should this be Supp. Fig. 3?

   Yes, it should have been. Numbering has been corrected.

6. Fig. 3 caption and Supp. Fig. 4: Why did the authors decide on a quadratic time-dependence of growth rates after the switch? Do they have any proposed mechanism for this?

   We thank the reviewer for asking this question as we realized the answer was not provided in the original manuscript. In short, the quadratic time-dependence was chosen purely for balancing a good empirical fit to the monoculture data with having a simple functional form that left only a single free parameter. We have not speculated on the mechanism, nor do we claim that the recovery shape is fundamentally quadratic but instead simply observe that a quadratic time-dependence is a good empirical fit to the data.

   The quadratic time-dependence was chosen for having a better empirical fit to Aci2's monoculture data than the two simpler options of (i) a "sharp" lag with no growth for t_{lag} and then an immediate recovery to the full growth rate and (ii) a linear time-dependence. The comparative empirical fits are provided in Appendix Figure S2 (below). As can be seen throughout the data (for example in Figures 2A-B and Appendix Figure S2), Aci2 experiences a gradual growth rate recovery after its diauxic shift. This gradual recovery makes a sharp lag a poor fit to the data. In the cases of 1:8 through 1:16 A:G, a full or nearly full growth rate recovery is realized, so a sharp lag can be fit asymptotically. But for all other conditions using the sharp-lag recovery shape to fit growth rates would involve considerable extrapolation and guesswork, leading us to consider other recovery shapes for fitting lag times to the data (Appendix Figure S2E).

   The next simplest recovery shape we considered was a linearly time-dependent growth rate. The results of using this recovery shape can be seen in Appendix Figure S2C. The fit appears good for 1:8 and 1:16 A:G (largely because the growth rates fully recover, so the $t > t_{\text{lag}}$ limit is realized). But, for other conditions the fit is worse and leads to questionable results. Specifically, for the intermediate conditions of 1:2 and 1:4 A:G, the linear recover shape first displays too rapid and then too slow a recovery, leading to significant uncertainty as to which part of the curve should be fit. For the conditions of 2:1 and 1:1 A:G, the data only or mostly includes the period in which the recovery shape displays too rapid a recovery, so attempts to fit a lag time would produce estimates

that are artificially high. For these reasons, the linear growth rate recovery shape could not be used.

The quadratic growth rate recovery shape was chosen as an in-between (Appendix Figure S2B) and also the next obvious step up in complexity. It initially predicts a slower recovery and smaller population sizes than the linear recovery shape (an improvement over that recovery shape's biggest limitation) and later predicts a smooth recovery as t approaches t_{lag} (an improvement over the sharp-lag recovery shape's biggest limitation). Additionally, the plots of growth rate over time in Appendix Figure S6 show a recovery that does appear to reassemble a quadratic function. Appendix Figures S2D and S3 show the specific fits, which appear satisfactory for all conditions. For this combination of reasons the quadratic growth rate recovery shape was chosen.

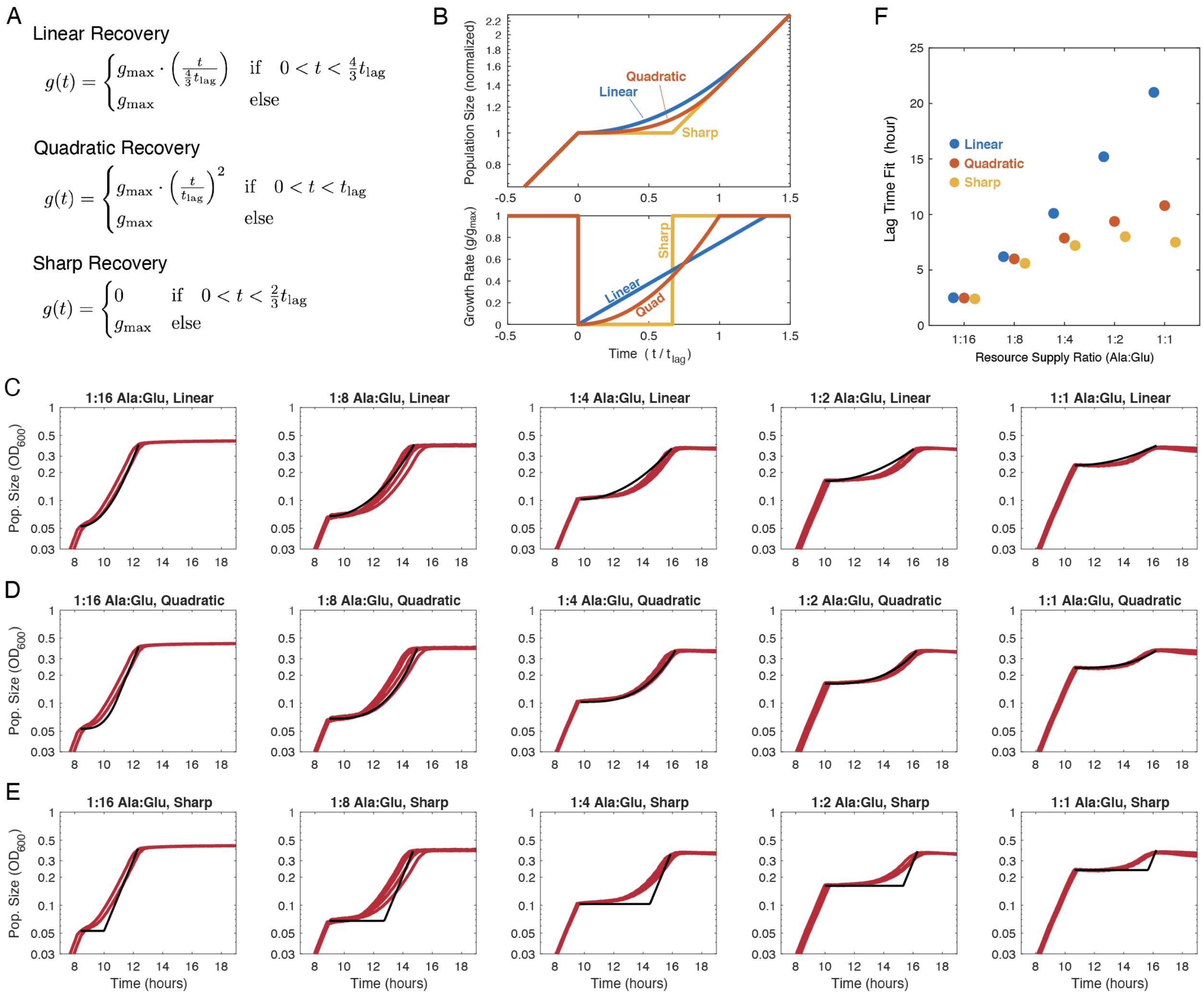

Appendix Figure S2. A quadratic growth rate recovery shape allows for more confident lag time fits than a linear or a sharp recovery. (A) The equations governing three simple empirical models for growth rate (and, after integration, population size) over time after a diauxic shift that starts at time $t = 0$. The steady-state growth rate is g_{SS} and the lag time is t_{lag}. The prefactors in front of t_{lag} in the linear and sharp recovery shapes are included to have the population sizes from each shape converge at time $t >> t_{\text{lag}}$. **(B)** The growth rates (bottom) and population sizes (top) derived from those shapes. The top panel illustrates how the quadratic growth rate shape can be thought of as in between the linear and sharp recovery shapes. **(C – D)** Lag

time fits using the linear, quadratic, and sharp growth rate recovery shapes. In all fits g_{SS} = 0.88 hr^{-1}, so the only free parameter is t_{lag} (fit independently for each recovery shape). The Aci2 optical density data from Fig 2B and EV2A is in the red and the best fit is in black. The quadratic fits in **D** are the same as Fig EV2A and reproduced here to facilitate comparison. **(C)** The linear recovery shape allows for close, confident fits at large glutamate supply fractions (two leftmost panels), but at more equal supply ratios (three center through rightmost panels) it is unclear whether the most appropriate fit would be a long lag time fit that predicts population sizes close to the experimental data before saturation is reached or a shorter lag time fit that would eventually converge towards an extrapolation of the experimental data if saturation were not reached. **(E)** Similarly, the sharp recovery shape can be fit asymptotically at large glutamate supply fractions but becomes more uncertain at more equal supply ratios. **(F)** Comparing the lag times fit using each recovery shape reinforces these considerations. Using the linear recovery shape yields excessively long lag times, while the sharp recovery shape yields lag time values similar to the quadratic model but that actually decrease at the 1:1 Ala:Glu condition (relative to the 1:2 condition) due to the biases of using that recovery shape.

We have briefly clarified the above points in the Results (line #136 in subsection "Aci2 is the fast-grower, but Pa is the fast-switcher"):

> " These monoculture experiments also yielded additional information about the diauxic lag times of each species. Notably, Aci2's diauxic lag time varied considerably with resource supply ratio. For fitting we assumed growth rates recovered proportional to the square of the time since the resource depletion (Materials and Methods). This recovery shape was chosen for having a better empirical fit to Aci2's monoculture growth curves than other shapes considered (Appendix Fig S2) while maintaining a simple, single-parameter functional form."

The Materials and Methods now contains a dedicated subsection "Lag Time Measurements" (line #653), the beginning of which reads as:

> " Lag times were fit using a growth rate recovery shape in which growth rate recovered proportional to the square of the time since the resource depletion,
>
> $$g(t) = g_{SS}\left(\frac{t - t_0}{t_{lag}}\right)^2 \quad \text{if} \quad t < t_{lag}\,, \quad \text{else} \quad g(t) = g_{SS}\,,$$
>
> where g_{SS} is the steady-state growth rate, t_0 is the resource depletion time, and t_{lag} is the species lag time. This recovery shape was used because it was a single-parameter fit that was a close empirical match to post-shift recoveries observed in the monoculture data, particularly in comparison to other recovery shapes considered (Appendix Fig S2). "

7. Figs. 3 and 4: I found all the figures to be rather busy, but these two in particular had a lot of tiny details that may be hard for some readers to glean, especially Figs. 3A an 4A. Can the authors simplify these?

We thank the reviewer for highlighting these two panels for us to work on simplifying. We have revised both panels (shown below). We made significant simplifications to 3A and some improvements to 4A. We have also made small changes to all other figures in an effort towards

reducing busyness. Figure 3 is now significantly decluttered. Figure 4 now has additional subplots added in response to Major Point #1 above, but we made an effort to add these subplots with as little resulting busyness as possible. We hope the reviewer finds the updated versions of our figures to be significantly improved:

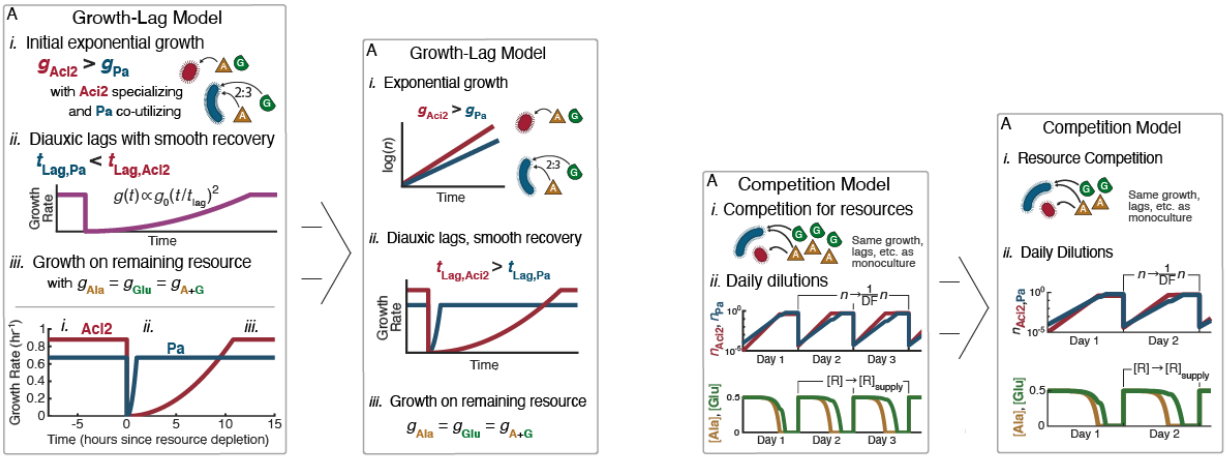

Figure 3A Original Figure 3A Revised Figure 4A Original Figure 4A Revised

8. Lines 221-222: The authors say cross-feeding is a likely explanation for the unexpected coexistence at extreme Ala:Glu ratios. But what about other possibilities? Maybe there are tradeoffs in other growth traits (e.g., death or survival during stationary phase), or history-dependent effects (e.g., growth traits change during subsequent cycles of growth, which cannot be observed in single monoculture growth curves).

Yes, there are many possible explanations, and we thank the reviewer for pointing this out as solely proposing crossfeeding left other possibilities neglected. With the reviewer's Major Point #3 (that much of our paper's value comes from focusing not on crossfeeding but on the growth dynamics themselves) in mind, we have decided to no longer give crossfeeding prominence above other, potentially more interesting, possibilities.

In our investigation associated with Major Point #2 (exploring the variations in Aci2's lag time more deeply), we uncovered that slight variations to the modeling of how Aci2's lag time varies would provide additional coexistence at small dilution factors and large glutamate supply fractions. This additional coexistence occurs because Aci2's lag time shortens as Pa drives it towards extinction, thus reducing the negative selection against Aci2 and preventing Pa from driving Aci2 totally extinct.

With alternative explanations in mind we have revised the corresponding paragraph in the Results (line #248 in subsection "Accurate prediction of response to environmental changes validates growth-lag model") to be:

> " The model did not, however, capture all the experimental observations. In particular, at high alanine supply fractions and low to intermediate dilution factors, the model predicted exclusion or near-exclusion of Pa whereas the experimental

observation was coexistence. This discrepancy was also present to a lesser degree at high glutamate supply fractions and notably at low dilution factors in single-resource competitions (Appendix Fig S9). There are several possible explanations. For example, modeling Aci2's lag time as dependent on its population size at the time of the diauxic shift rather than the initial resource supply ratio would predict coexistence at some conditions in which exclusion is falsely predicted under the presented model (Fig EV4). Dynamics occurring during the stationary phase, a slight density-dependence of Pa's growth rate, and crossfeeding are all alternative possible explanations. But, without reason to pursue any explanation in particular and a possibility of a number of factors each playing a small role, we were satisfied with how many of the key features had been accurately predicted. "

9. Lines 329-341: I don't entirely agree with the authors' characterization of initial vs. diauxic lags. From the perspective of microbial physiology, I would argue they are categorically the same: both represent transitions between growth states (the former just being a transition from zero growth to non-zero growth). However, if they lead to qualitatively different consequences for ecology, then that is the important point to make here. But if that is the case, what is the fundamental difference based on the growth dynamics?

   We thank the reviewer for encouraging us to reconsider this comparison. We agree that initial and diauxic lags are essentially the same phenomenon. When species have only a single growth rate, however, the introduction of a diauxic lag is fundamental different because it divides the growth phase in to two subphases and creates a second environmental parameter (the time of the diauxic lag onset in addition to the final resource depletion time) that adds a new dimension to the feedback between species and environment and the corresponding potential for negative frequency-dependent selection.

   Previous work has shown that initial lags followed by only a single exponential growth phase can produce stable coexistence between species (Manhart 2018), but direct comparison to this work is complicated by subtle but highly consequential differences in how the dilution phase is modeled. As noted above in our response to the reviewer's Major Point #1, a large section of the Appendix has now also been refocused as a comparison between initial and diauxic lags. Main points from this comparison include (i) with all yields equal diauxic lags allow two species to coexist but initial lags do not, (ii) with variable yields and a density-dependent dilution factor (as used in Manhart 2018) initial lags allow two species to coexist while diauxic allow three species to coexist, and (iii) with randomly sampled species coexistence is more likely when lags are diauxic rather than initial.

   We have rewritten the paragraph in question to begin by noting the similarities between initial and diauxic lags, then discuss the significance of a lag effectively splitting a growth phase in two, and finally contrast our results with previously published results on initial lags in a manner that more explicitly addresses the consequences of how dilution phases are modeled. There are interesting consequences of combining lags of either form with frequency-dependent dilution phases that we believe others in the field will benefit from a clarification of, so we have restructured and expanded a section of our Appendix material to explore the differences between diauxic and initial lags in detail. The paragraph (beginning at line #368 in the Discussion) now reads:

“ In addition to diauxic lags, microbes often display initial lags, which occur when previously stationary species are presented with fresh resources. Diauxic and initial lags are similar phenomena: a microbe requiring time to reach its maximum growth rate when presented with a new environment. Diauxic lags do, however, allow for coexistence in ways that initial lags do not, primarily because diauxic lags divide the growth phase into two subphases with distinct frequency-dependent dynamics while initial lags only affect a single growth phase. Recent theoretical work has, however, shown that tradeoffs between growth rate and initial lag can be a source of coexistence with only a single growth phase (Manhart & Shakhnovich, 2018; Manhart et al, 2018), but for this coexistence to be possible without parameter fine-tuning a density-dependent dilution factor is necessary. In the Appendix, we explore the effects of adding initial or diauxic lags in a variety of scenarios including already-biphasic growth and conclude that diauxic lags have a greater tendency to produce coexistence than initial lags do. Regardless of their relative potency, both initial and diauxic lags are ubiquitous phenomena with considerable ecological relevancy that warrant ongoing theoretical and ecological study, ideally from a unifying approach. ”

10. Suppl. Fig. 1: Can the authors please also add a legend to identify the mapping between species and colors?

    Labels have been added. (This figure is now part of Figure EV1.)

11. Suppl. Fig. 3: This figure also needs a legend to distinguish data vs. fits.

    This information has been added to the figure caption. (This figure is now Figure EV2.)

12. Suppl. Fig. 4: It is hard to identify small differences in growth rates from the growth curves themselves. Why not plot inferred growth rate vs. Ala:Glu?

    This figure has been re-envisioned to show greater clarity as to the growth rate at each specific condition and has been entirely remade as Appendix Figure S1:

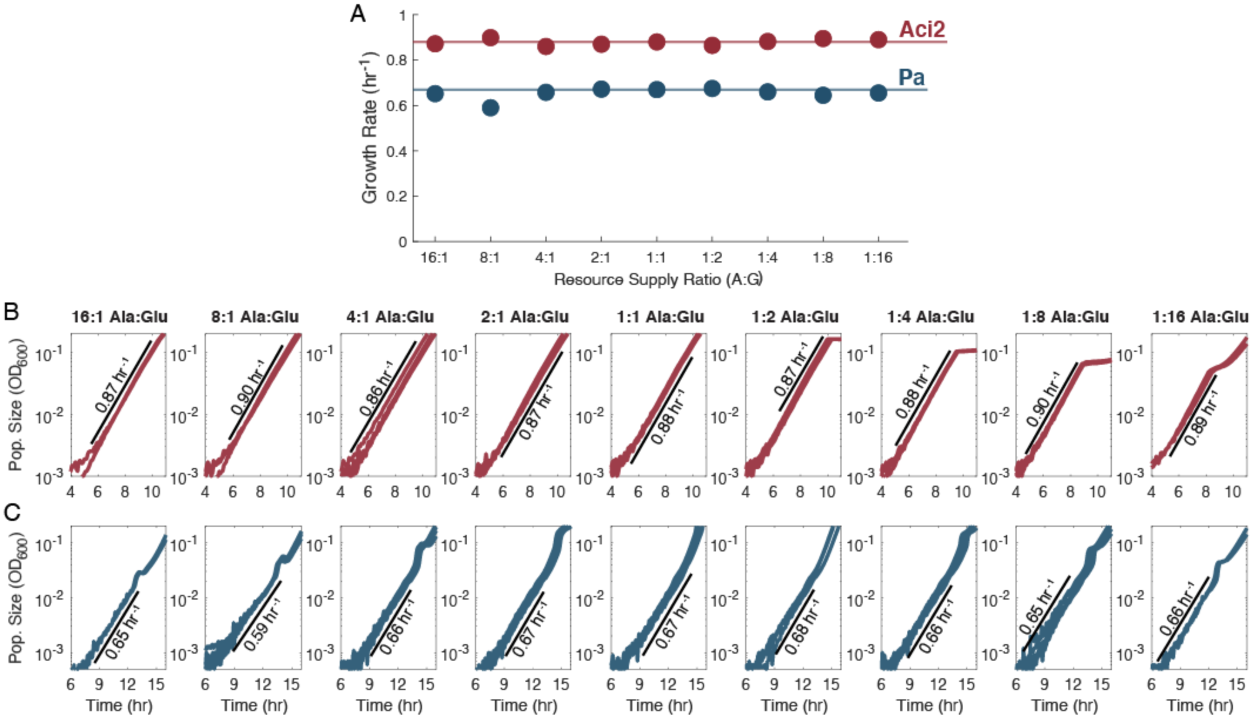

Appendix Figure S1. Two-resource growth rates do not vary with resource supply ratio. (A) Growth rates for Aci2 and Pa at nine different ratios of alanine and glutamate (total supply constant at 0.1%w/v). Aci2's growth rate fluctuates by +/-0.01 hr^{-1} and Pa's by +/-0.03 hr^{-1} (standard deviations of the nine growth rate fits), compared to uncertainties of approximately +/-0.01 hr^{-1}on each individual fit. **(B)** The Aci2 optical density data from Fig 2B in red with the Aci2 growth rates from **A** in black. **(C)** The Pa optical density data from Fig 2E in blue with the Pa growth rates from **A** in black.

13. Suppl. Fig. 6: In the rightmost panel, why is Pa fraction not just zero, since it has slower growth rate in all single-resource conditions? Is it because they are measuring its frequency after finite time (7 days)?

As we have worked to streamline the manuscript and Appendix, we have decided to remove this figure. We nevertheless thank the reviewer for pointing out this lack of clarity.

(To still answer the reader's question: This figure was a weighted average of the experimental results for single-resource environments (Appendix Figure S9) and showed non-zero Pa fractions because there was some experimentally observed coexistence at small dilution factors in the single-resource environments.)

14. Suppl. Fig. 7: What is the correlation coefficient? It might be helpful to also plot this on a log-log plot (in addition to the existing linear-linear plot) to resolve the low-frequency variation.

We have added the correlation coefficient to the list of statistics we provide. The Pearson correlation coefficient is 0.72 ($p < 10^{-9}$). We do note, however, that the intention of our model was

to predict qualitative trends in community composition, rather than being an attempt at a close quantitative fit.

Regarding the suggestion of a log-log plot: Over one third of the data in this figure (20 out of 54 points) has a predicted and/or observed fraction of zero (with Pa predicted to go entirely extinct and/or not a single Pa colony counted). Plotting the data on a log-log scale would therefore necessitate excluded over a third of the data, and we have therefore declined to do so. We have, however, added a plot of the observed fraction Pa for all cases in which Pa was predicted to go extinct in order to resolve the many data points that are stacked in the bottom left corner of the linear-linear plot. The original Suppl. Fig. 7, this new plot, and two additional subplots (provided in response to a question from Reviewer 3) have become Appendix Figure S10 shown below:

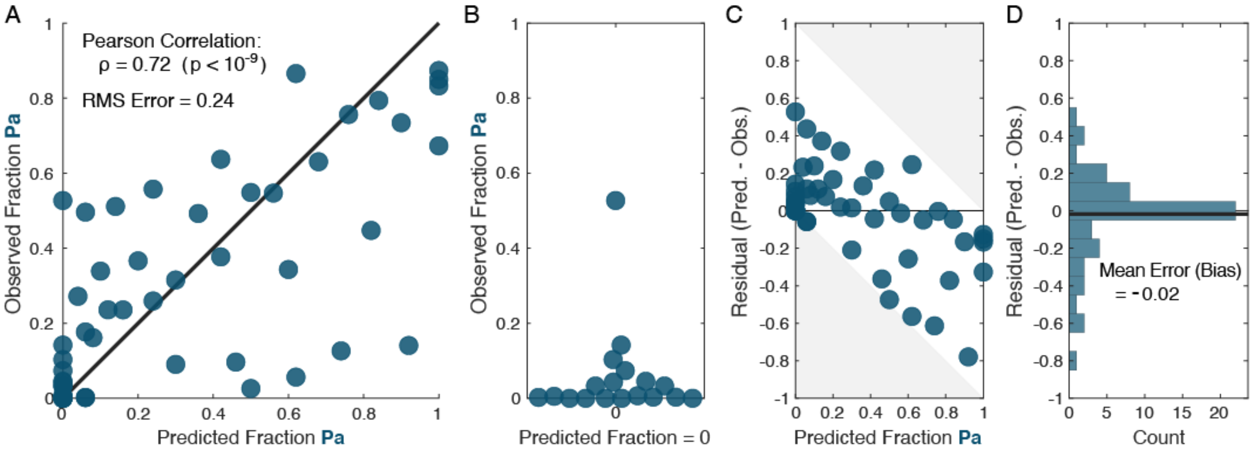

Appendix Figure S10. Quantification of Aci2 and Pa on alanine and glutamate model prediction vs experimentally observed outcome. (A) Steady-state predicted fraction Pa vs experimentally observed fraction Pa for all resource supply ratios and dilution factors tested. **(B)** Observed fraction Pa for all cases in which Pa is predicted to go extinct. Horizontal spacing is added to separate the data points that are otherwise stacked (e.g. in the bottom left corner of **A**). **(C)** Residual plot of the same data. Shaded gray regions are the disallowed regions that would require an observed fraction less than zero or greater than one. **(D)** Histogram of the residuals. Dark horizontal line near center indicates the mean residual, which is a species fraction of -0.02.

15. Suppl. Fig. 9 caption: "with one generalized cross-feeding product excreted from each species." Is this missing the word "each"?

This caption no long exists (see below), but we nevertheless thank the reviewer for the detailed proofreading.

16. Suppl. Fig. 9 caption: Here the authors describe a model for cross-feeding during stationary phase, but the details seems strange to me. How did they choose these details?

We wished to incorporate cross-feeding into a model that still featured sequential growth on resources. We found it difficult to do so in an elegant manner that did not reduce to uninteresting, degenerate behavior. In response to Minor Point #8 above, we have actually to remove this figure

as we felt dedicating the space to cross-feeding as an explanation for the unexpected coexistence drew attention away from other possibilities.

Reviewer #2:

In this report, Bloxham et al address the question of coexistence of species in the presence of two resources. In a specific example where Pseudomonas aurantiaca (Pa) loses in head-on competition against Acinetobacter sp (Aci2) on both alanine and glutamate independently, authors found that both species coexist when both the resources were added together. By analyzing the monoculture growth patterns in two-resource condition, they figured that Pa's shorter diauxic lag might be responsible for the coexistence with Aci2. They built a simple model based on growth rate and diauxic lag to successfully explain the phenomenon, made predictions for few more head-on competitions involving a bunch of more bacterial species and different carbon sources, and verified them experimentally. It is a useful work that sheds light on an important aspect of microbial ecology.

The importance of lag time in dictating fitness effects and relative populations of binary competitors in a single resource environment have been previously demonstrated in both experiments (Adkar, et al, Nat Ecol Evol, 2017) and theory (Manhart et al, RSB, 2018, Nat comm, 2018). It was also explicitly shown that fitness benefits of lag times are best realized under conditions of nutrient limitations. It is not unrealistic to expect that diauxic lags would be an important factor in determining fitness effects (coexistence) in two resource conditions. This idea is nicely demonstrated in this article.

I have a several major methodological concerns and are listed below, not in any order of priority:

We thank the reviewer for an overall positive review of our work and their high opinion of how it fits in with other recent research. The Manhart 2018 papers the reviewer cites were indeed early inspirations and influences for our modeling sections, so we are pleased to see our paper framed as building off this body of work. We appreciate the detail with which the reviewer clearly read our manuscript and the resulting feedback regarding methodology and where descriptions lacked clarity. With the reviewer's feedback now incorporated, we believe the paper is significantly clearer than the original manuscript and that readers will benefit as a result, so we thank the reviewer for their feedback.

1. It is not clear how the diauxic lag times are estimated from experimental data. It is not clearly defined anywhere in the text. Supplementary Fig 43 shows fits for the exponential phases and lag times, but it was not clear how the fits for lag times were done. Was the equation defined in 'Modeling' section used to derive the lag time here? This is a crucial information, as most of paper relies on this.

   We thank the reviewer for pointing out this lack of clarity, especially given its importance to the entirety of the paper. In response, we have provided more explicit and detailed information about how we fit lag times in the Materials and Methods section. Additionally (and also in response to the reviewer's Point 6 below), we have refit a large quantity of lag times using methodology that is now more consistent across the paper, as we suspect variations in methodology may have contributed to the lack of clarity.

As the reviewer determined, all lag times are fit assuming the growth-rate recovery shape defined in the Materials and Methods,

$$g(t) = g_{\mathrm{SS}} \left(\frac{t - t_0}{t_{\mathrm{lag}}} \right)^2 \quad \text{if} \quad t < t_{\mathrm{lag}}\,, \quad \text{else} \quad g(t) = g_{\mathrm{SS}}\,,$$

where g_{SS} is the post-recovery steady-state growth rate (assuming a fully recovery is achieved before saturation), t_{lag} is the lag time, and t_0 is the onset of the diauxic shift. Assuming simple diauxic growth, the steady state growth rate g_{SS} would be the species' single-resource growth rates on the remaining resource. (The quadratic functional form was chosen due to a strong empirical fit to the monoculture data. A thorough discussion and comparison to other options is provided in response to Reviewer 3's Minor Point #10.)

The quadratic growth rate recovery yields a simple cubic form for log-scaled population size as a function of time:

$$\log n(t) = g_{\mathrm{SS}} \frac{(t - t_0)^3}{3\, t_{\mathrm{lag}}{}^2} \quad \text{if} \quad t < t_{\mathrm{lag}}\,, \quad \text{else} \ \log n(t) = g_{\mathrm{SS}} \left(t - \frac{2}{3} t_{\mathrm{lag}} \right).$$

If g_{SS} is fixed and t_0 is known, then the only free parameter is t_{lag}.

The lag time fits for the focal example of Aci2 and Pa on alanine and glutamate used fixed values of g_{SS} and were therefore single-parameter fits. The fixed values of g_{SS} were the species' two-resource growth rates. The two-resource growth rates were used instead of single-resource growth rates to maintain consistency with the modeling decision to only have a single growth rate for each species.[3]

With fixed g_{SS} values defined for Aci2 and Pa on alanine and glutamate, the single-parameter lag time fits were performed manually using a Matlab interface. First, the time and population size at the onset of the diauxic shift were defined using a plot of population size as a function of time. Then, the value of t_{lag} was manually adjusted until a visually best fit was obtained. Example fits are provided in Figure EV2 and the full set in Appendix Figures S3 and S4. A fully programmatic approach to lag time fitting was attempted but deemed unfeasible (with attempts generally resulting in biases towards longer lag times, which are already a below-listed concern of this reviewer), but the reviewer and readers can observe the quality of the fits in Figure EV2 and Appendix Figures S3 and S4.

For the survey of additional species and resources, we have collected new data and refit all growth rates and lag times. (For more detail, see our response to Point #6 below.) Experiments were performed with resource supply ratios of 4:1, 1:1, and 1:4, and lag times were fit to whichever condition had the earliest diauxic shift. The condition with the earliest diauxic shift was used so as to have the longest period of post-shift growth to use for the lag time fits and had the additional relevancy of generally being the shift from the species preferred resource (or the one it consumed

[3] In response to the reviewer's Point 4 below, we have now also provided supplementary information on the slight changes to the fits and modeling results that occur if we instead fit the lag times using the single-resource growth rates. In brief, because the single-resource growth rates are only slightly slower, the fit lag times decrease only slightly (by ~15%) with no qualitative and little quantitative impact on the modeling results.

a larger fraction of if it was co-utilizing) to its second preference. For this set of fits, g_{SS} was not fixed, and both g_{SS} and t_{lag} were adjusted simultaneously to produce the best fit. We decided to leave g_{SS} unfixed for this set of fits as in many cases there was a long period of exponential growth to obtain a reliable g_{SS} and because distinctly better fits were often obtainable due to two-resource and single-resource growth rates sometimes being further from equal than in the case of Aci2 and Pa on alanine and glutamate. The fits for the survey of additional species and resources are available in the Figure 6 Source Data.

The Materials and Methods now contains a dedicated subsection "Lag Time Measurements" (line #653), which reads as:

> " Lag times were fit using a growth rate recovery shape in which growth rate recovered proportional to the square of the time since the resource depletion,
>
> $$g(t) = g_{SS}\left(\frac{t - t_0}{t_{lag}}\right)^2 \quad \text{if} \quad t < t_{lag}\,, \quad \text{else} \quad g(t) = g_{SS}\,,$$
>
> where g_{SS} is the steady-state growth rate, t_0 is the resource depletion time, and t_{lag} is the species lag time. This recovery shape was used because it was a single-parameter fit that was a close empirical match to post-shift recoveries observed in the monoculture data, particularly in comparison to other recovery shapes considered (Appendix Fig S2). In the Main Text, lag times are reported as a function of resource supply ratio. Lag times were also fit for different initial dilution factors but no variation was observed (Appendix Fig S8).

> " Integrating the quadratic growth rate recovery yielded a simple cubic form for log-scaled population size as a function of time:
>
> $$\log n(t) = g_{SS}\frac{(t - t_0)^3}{3\,{t_{lag}}^2} \quad \text{if} \quad t < t_{lag}\,, \quad \text{else} \quad \log n(t) = g_{SS}\left(t - \frac{2}{3}t_{lag}\right).$$

> " For fitting Aci2 and Pa lag times on alanine and glutamate, g_{SS} was fixed as the species' two-resource growth rates (0.88 hr^{-1} and 0.67 hr^{-1}; Fig 2A). (Fitting lag times using the species' single-resource growth rates had only a minor impact on the results (Appendix Fig S7).) Because g_{SS} was fixed, the lag time fit was a single-parameter fit. Lag time fits were performed manually. First, the onset of the diauxic shift was defined on a log-linear plot of optically density over time. Second, the value of t_{lag} was adjusted until the best empirical was obtained. Upper and lower bound estimates were obtained by determining the maximum and minimum values of t_{lag} that produced remotely reasonable fits. All fits, including upper and lower bound estimates, are presented in Appendix Fig S3 and S4.

> " Pa has a small spikes in optical density at the onset of its diauxic shift and at saturation (Appendix Fig S5). These spikes were ignored when fitting the Pa lag times such that lag times correspond to the time it takes Pa to reach its steady-state growth rate not the maximum observed growth rate as this is believed to be an artifact. We did not engage in a mechanistic study of these spikes in optical density, but *Pseudomonas* have been previously observed to rapidly increase per capita

optical density under environmental changes due to morphological changes (Bernheim, 1963).

“ For the survey of additional species and resources, lag times were fit to data from whichever of the three resource ratios (4:1, 1:1, 1:4) produced the earliest-occurring diauxic shift (to maximize post-shift growth available for fitting). For these fits, g_{SS} was not fixed and was manually adjusted in parallel to t_{lag} being adjusted. These fits are all shown in the Figure 6 Source Data. The Aci2 and Pa lag times were refit for this comparison with g_{SS} unfixed so as to be subject to the same biases (if any) as the other species’ fits. In some cases, a reliable lag time fit could not be obtained. In these cases the data and corresponding competitions were excluded from the analysis. Notes on specific fits are provided in the Figure 6 Source Data.”

The Extended View figure referenced above is:

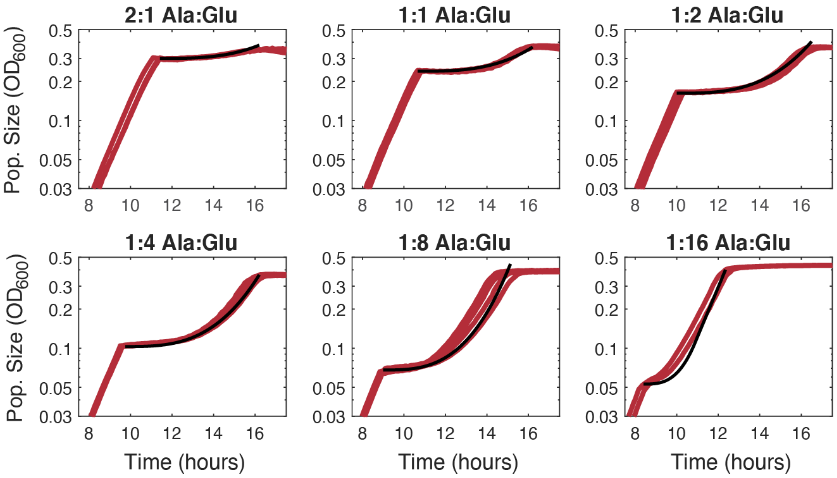

Figure EV2. Example lag time fits. The Aci2 monoculture data from Fig 2B are shown in red with the fits used to produce the lag time estimates shown in red (Materials and Methods). The remaining Aci2 fits are presented in Appendix Fig S3. The Pa fits are presented in Appendix Figure S4 with a note on interpretation of Pa’s optical density data provided in Appendix Fig S5. These fits were performed using the species’ two-resource growth rates as their post-recovery steady-state growth rates to maintain consistency with modeling decisions. Fits using the species’ single-resource growth rates are provided in Appendix Fig S7 and differ only slightly.

The remaining lag time fits for Aci2 and Pa on alanine and glutamate can be found in Appendix Figures S3 and S4 (although we would encourage the reviewer to read our response to their next point before reviewing the Pa fits). The lag time fits for the survey of additional species and resources are available in the Figure 6 Source Data.

We again thank the reviewer for encouraging us to clarify our methodology as we are sure readers will appreciate the description and Appendix material that is now provided.

2. The lag time fits in Supplementary Fig 3 are confusing and seems that the lag times extend until saturation. In that case, lag times for Pa extend beyond 1 hr. I am sure I am not reading it right, but more explanation will help here.

We thank the reviewer for raising this question, as there are complications to Pa's OD curves that warrant explicit acknowledgement and discussion, which has now been added to the manuscript. In short, the appearance of a longer lag time for Pa is due to an artifact in Pa's optical density that occurs when it saturates, and, although it gives the impression of suddenly faster growth, this artifact should not be used for fitting diauxic lag times.

Appendix Figure S5 (below) shows Pa monoculture data for the 1:16 Ala:Glu environment. (The 1:16 condition is used as an example to maximize the temporal separation between the diauxic shift and saturation.) As can be seen, Pa (i) initially grows at a steady rate of 0.67 hr^{-1} on alanine and glutamate, then (ii) has a sudden spike in optical density followed by a brief period of constant optical density, then (iii) grows exponentially for approximately 2.6 hours at 0.57 hr^{-1}, and then (iv) has another sudden spike in optical density before reaching saturation.

The spike in optical density at the diauxic shift has a peak "growth rate" of 3.4 hr^{-1} (a doubling time of 12 minutes compared to its steady-state doubling times of 1.0 – 1.4 hours) and the spike at saturation has a peak of 2.4 hr^{-1} (17-minute doubling time). Because these spikes would correspond to such large growth rates, we cannot label them as ordinary growth but must instead assume they are optical density artifacts.

The growth rate of 0.57 hr^{-1} that Pa holds for 2.6 hours between its diauxic shift and saturation matches the growth rate measured for Pa in a glutamate single-resource environment (Figure EV1A). Because this period of growth is steadily exponential at the expected growth rate and because the later spike in optical density at saturation does not make sense as ordinary growth, Pa should be considered to have recovered from its diauxic lag when it returns to steady growth at the expected rate.

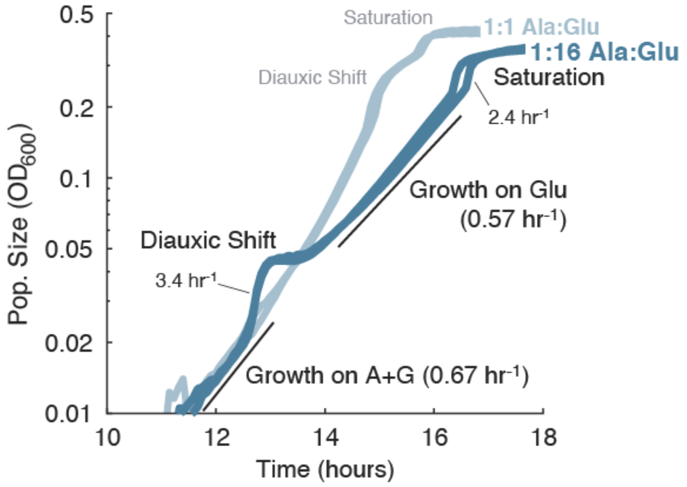

Appendix Figure S5. Small spikes in Pa's optical density at the onset of its diauxic shift and at saturation appear to be artifacts and can be easily accounted for in the data analysis. Shown is an

annotated growth curve for Pa growing in a 1:16 Ala:Glu environment (dark) with a growth curve for Pa growing in a 1:1 environment shown for comparison (light and behind). Periods of steady-state growth can be seen first in the two-resource environment and later on the remaining glutamate. Pa's steady-state growth rate on the remaining glutamate matches the value of 0.57 hr^{-1} from single-resource experiments (Fig EV1A). In addition to periods of steady growth, sudden spikes in optical density are also clearly visible. If these spikes corresponded to ordinary growth (i.e. increase in biomass), Pa's growth rate would reach as high as 3.4 hr^{-1} at the time of its diauxic shift. This is implausible, so these spikes must be changes in *per capita* or *per biomass* optical density. We did not study these spikes in optical density any further, but sudden increases of similar magnitude to the optical density of *Pseudomonas* have been previously observed and linked to changes in cell morphology resulting from environmental perturbations.[1] For fitting Pa's lag times, the time for Pa to reach its steady-state growth rate (and not the time until the spike in optical density at saturation) is what's relevant (Materials and Methods). The greatest uncertainty in the Pa lag time fits comes from determining at what time and optical density to define the onset of Pa's diauxic shift. In the reported fits (Appendix Fig S4) we have defined the onset such that we obtain lag time values in the middle of the possible range. Other choices could have affected Pa's lag times by up to +/-20 minutes, but Pa's lag times would still be very short compared to Aci2's and reasonably approximated by a constant value of 1 hour.

We have not determined the source of Pa's sudden increases in optical density. Pa does produce an orange pigment that becomes noticeable around the time it saturates, but it is unclear if this would be sufficient to explain the increases in OD. The supernatant at the end of experiments lacks sufficient optical density for an excreted pigment to be the source, but some pigment appears to be contained within Pa cells. Another hypothesis is that Pa may respond to sudden nutrient limitation by changing cell morphology or size in a way that increases optical density. Sudden increases to the optical density of *Pseudomonas* in changing environments have been previously observed and linked to rapid changes in cell morphology (Bernheim 1963). If the reviewer is interested in this phenomenon, we suggest *Pseudomonas veronii* (ATCC 700474) as a species with a particularly pronounced OD spike at saturation and the onset of diauxie for an investigation.

Much of the above discussion has been incorporated into the caption of Appendix Figure S5 (above), which is now referenced in the caption to Figure 2E and the Materials and Methods subsection on lag-time fitting (already quoted in our reply to the reviewer's Point #1 above).

3. It is not clear how you get 12h diauxic lag time for 2:1 A:G for Aci2 as shown in Supplementary Fig 3A. One way I can think of is to fix g_mu,0, and extrapolate time based on the quadratic growth recovery equation as defined in the 'Modeling' section to achieve that growth rate. However, it is not clear if that how it is done. Moreover, it is unclear what is g_mu,0. Is it the growth rate for the second phase?

   Yes, that is indeed how it was done. As described in response to the reviewer's Point 1 above, g_{SS} (previously $g_{\mu 0}$) was fixed at 0.88 hr^{-1}. From there, we used the quadratic growth recovery equation to obtain a best fit. There is, as the reviewer points out, some additional uncertainty that is introduced when saturation is reached long before the projected full growth rate recovery. The 12-hour value is, however, consistent with the trends that are present for other conditions that have more post-growth to use for fitting a lag time.

4. If the lag times are derived based on the assumption of the model, i.e., the pre- and post-depletion growth rates are equal, then it may artificially overestimate diauxic lags. I am not suggesting that the diauxic lags may not vary, but in this case, it is important to derive lag times for the second transition without any constraints to make sure the variation is not an artifact. Moreover, I did not find any explanation in the manuscript justifying the assumption regarding growth rates. In fact, from most of the experimental data, it is clear that both phases do not have equal growth rates and the second growth rate is lower than the first one. This assumption of equal rates would introduce quite a bit of error in estimation of proportion of the species. However, it is surprising that despite this, the model predictions match the experimental outcomes in many cases. It will be useful if authors comment on this. I think a model which explicitly makes use of both experimental values of growth rates and lag times would be more appropriate here.

   We thank the reviewer for encouraging us to dive into this issue more deeply. Using an assumption of a faster-than-actual post-shift growth rate does indeed produce a slight bias towards fitting longer-than-actual lag times. However, we found that this bias has only a minor effect on the results of our modeling. Thus, for the Main Text, we decided to fit using the two-resource growth rates for simplicity and for consistency between the data analysis and modeling.

   As the reviewer notes, one of the surprising results of our paper is that a simple model with just a single growth rate and a lag time for each species can actually explain unexpected coexistence and how community composition would respond to environmental changes. In revising our paper, we have made an effort to note the significance of having only used a single growth rate for each species more clearly. We could have chosen to write an arbitrarily complex model (including, for example, growth rates that depend upon nutrient concentrations etc.) and likely gained an incrementally more accurate prediction with each modeling addition, but each additional complexity would have reduced the interpretability of our modeling results. For example, tradeoffs between pre- and post-shift growth rates can lead to stable coexistence in the absence of diauxic lags, so the introduction of multiple growth rates for each species would have complicated any discussion of how coexistence arises within the model.

   Nevertheless, we have provided new Appendix material (and references to it in the Main Text) to demonstrate how more detailed models lead to minor quantitative effects.

   In single-resource experiments, Aci2's growth rates are 0.68 hr^{-1} on alanine and 0.74 hr^{-1} on glutamate, compared to 0.88 hr^{-1} on both. Pa's growth rates are 0.5 hr^{-1} on alanine and 0.57 hr^{-1} on glutamate, compared to 0.67 hr^{-1} on both. (These fits are provided in Figure EV1A.) Compared to the two-resource growth rates, Aci2 is 23% slower on alanine and 16% slower on glutamate, and Pa is 25% slower on alanine and 15% slower on glutamate. These growth rates were used to obtain the alternative lag time fits suggested by the reviewer:

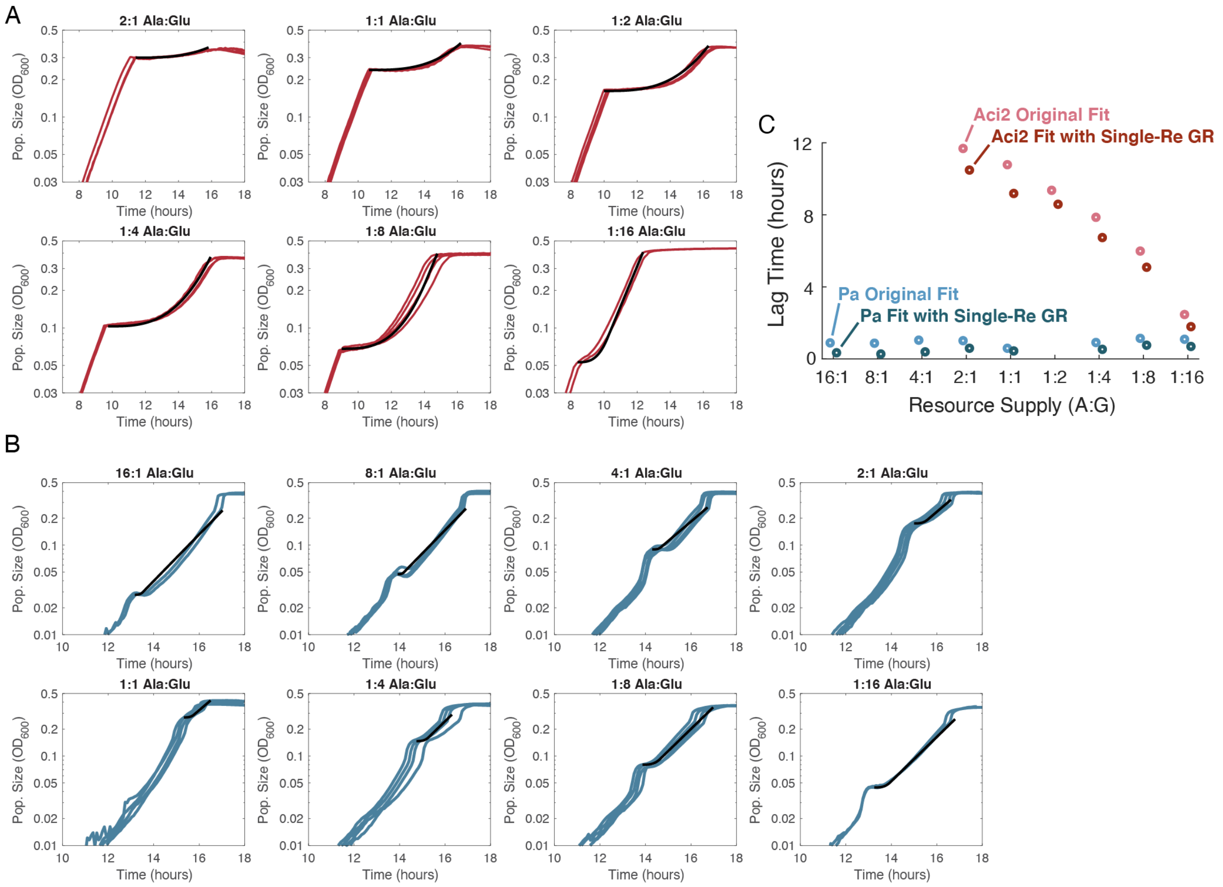

Appendix Figure S7. Lag times fit using single-resource growth rates for post-shift steady-state growth rates are similar to the lag times fit using the two-resource growth rates. The lag times used in all Main Text modeling were fit using g_{SS} (i.e. g(t >> t_{lag})) values equal to the species' two-resource growth rates. This decision was made to maintain consistency with the modeling having only a single growth rate for each species. The measured single-resource growth rates for each species were, however, 15-25% slower than the two-resource growth rates (Fig EV1A). **(A–B)** Lag times were refit using the species' single-resource growth rates for their post-shift g_{SS}. The same Aci2 and Pa optical density data as in Fig 2B (and Appendix Fig 3 and 4) are shown in red and blue respectively, the updated best-fit lag recoveries (from which the lag times are extracted) are shown in black. **(C)** Summary of Aci2 (red) and Pa (blue) diauxic lag times as fit and modeled with in the Main Text narrative (light) and as refit in A and B using the species' single-resource growth rates (dark). Aci2's lag times are on average only 15% +/-7% shorter. Pa's lag times are on average 54% +/-16% shorter, but, because Pa's lag times are already short, this is only 28 +/- 4 minutes shorter. Across the five conditions with lag times for both Aci2 and Pa, the difference in lag times (i.e. $t_{lag,Aci2}$-$t_{lag,Pa}$) decreases by just 13% +/-2%. Results from modeling with these updated lag times are provided in Appendix Fig S11. (Means and standard errors reported.)

As can be seen in Appendix Figure S7C, Aci2's lag time is decreased by only 15% $^{+}$/- 3% on average (mean and standard error). Meanwhile, Pa's lag time (while occasionally decreased by a large fractional amount) is decreased by only 28 $^{+}$/- 4 minutes on average. Additionally, because each species' lag times are decreasing the effects of these changes partially cancel out. Combining the updates to each species' lag times in the five cases in which Aci2 and Pa both had lag times fit, the difference between Aci2's and Pa's lag time decreased by just 13% $^{+}$/- 2% (mean and standard error). Even at a supply ratio of 1:16 A:G (where the difference in lags is the smallest

and, relatedly, the fractional decrease in difference is the largest), the difference in lag times decreased by just 21% (from 1.4 hours to 1.1 hours).

The changes in lag times then produce similarly small changes to the modeling prediction:

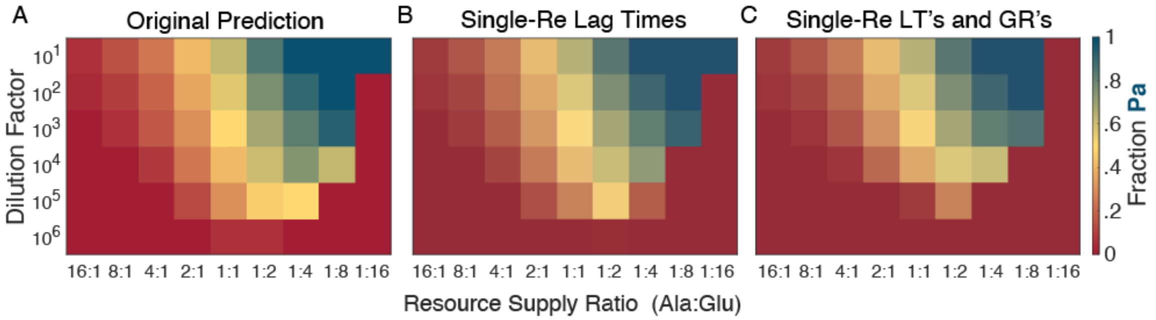

Appendix Figure S11. Removing the assumption of equal pre- and post-shift growth rates changes modeling predictions only slightly. (A) Same modeling prediction as the Main Text reproduced here to facilitate comparison. This modeling used the species' two-resource growth rates for both pre- and post-shift growth to have a single growth rate for each species and used lag times fit with post-shift g_{SS} fixed as the species' two-resource growth rates for consistency. **(B)** Same modeling repeated, still using two-resource growth rates pre- and post-shift, but now using the lag time values fit using single-resource growth rates for post-shift g_{SS} (Appendix Fig S7). **(C)** Modeling repeated again using single-resource growth rates post-shift (such that each species now has three total growth rate) as well as lag times fit using single-resource growth rates.

Because the changes to both lag times and modeling predictions are small, we present the simpler model in the Main Text. The use of this simpler model reinforces our central message that a simple model of growth and lag can insightfully explain and predict competitive dynamics between two species.

Appendix Figure S7 provides the alternative lag time fits and Appendix Figure S11 provides the modeling results using these alternative fits with or without separate post-shift growth rates. Additional discussion and references to this Appendix material have been added at a couple points:

- In the Results (line #263 in subsection "Accurate prediction of the response to environmental changes validates growth-lag model"), the following note has now been added:

  " This error could have been reduced by adding additional model complexities or fitting parameters to competitive outcomes unconstrained by the monoculture characterizations. For example, one complexity would be to use the species' single-resource growth rates for post-shift growth instead of having a single growth rate for each species, but this addition would change the modeling prediction surprisingly little (Appendix Fig S11). The goal of our modeling was not, however, to obtain the closest possible empirical fit, but to instead show that a simple model with a minimal number of elements could predict qualitative features over a wide range of environmental conditions. "

- In the Materials and Methods (line #663 in subsection "Lag Time Measurements"), the following reference to Appendix Figure S7 has been added:

" For fitting Aci2 and Pa lag times on alanine and glutamate, g_{SS} was fixed as the species' two-resource growth rates (0.88 hr^{-1} and 0.67 hr^{-1}; Fig 2A). (Fitting lag times using the species' single-resource growth rates had only a minor impact on the results (Appendix Fig S7).) "

- In the Materials and Methods (line #707 in subsection "Modeling"), the following reference to Appendix Figure S11 has been add:

" Post-switch steady-state growth rates were the same as the pre-shift growth rates ($g_{SS,Aci2} = 0.88\ hr^{-1}$ and $g_{SS,Pa} = 0.67\ hr^{-1}$) to avoid a tradeoff between two-resource and single-resource growth rates complicating the interpretation of the results and because the effect of using the measured single-resource growth rates for post shift g_{SS} was minor (Appendix Fig S11). "

5. Two different methods of growth rate estimations are described on lines 391-393 and 421-425. It is not clear which one is used? The later one, as described, may likely yield growth rates lower than maximum growth rates.

We again thank the reviewer for pointing out a lack of clarity. We used separate methods for plotting instantaneous growth rates and for calculating the reported growth rate values:

1. For plotting instantaneous growth rates, we used the method described at lines 391-393 (in the original submission). This method used a 30-minute rolling window to calculate instantaneous growth rates (7 data points with our 5-minute sampling interval). First, a background value was calculated (for each individual well of the 96-well plate) as the minimum observed OD value after median filtering with a bin width of 30 minutes, and the background value was subtracted from the data set. Then for each timepoint, a linear least squares fit was performed using a 30-minute window centered on the timepoint and the slope of the fit was divided by the optical density value of the center timepoint. This yielded an instantaneous growth rate value at each time in the experiment (excluding the first and last 15 minutes).

2. For calculating steady-state growth rate values, we used the method described at lines 421-425. This used a longer 4.5-hour window (selected as the period of time after the reader noise became insignificant but before any breaks from linear growth associated with diauxic shifts occurred). The same background value as described above was subtracted off and data was log-transformed. We then used a Thiel-Sen estimator (Thiel 1950, Sen 1968) in which a slope was fit through every pair of points and the median slope was used as the reported growth rate. The reported uncertainty is the standard deviation of the set of slopes.

As the reviewer points out, the former method, by using a half-hour window, is good for observing maxima and minima in the growth rates (e.g. before, during, and after a diauxic shift) and allows for plotting instantaneous growth rates as a function of time. The later method, by using a longer window and the highly robust Thiel-Sen estimator, provides a reliable estimate for when a single

growth rate value is desired – in our case steady-state two-resource growth rates to be used in the modelling.

We have clarified in the Methods (line #629 in subsection "Growth Rate Measurements") which method is used for which purposes and juxtaposed their descriptions to facilitate this clarification:

> " Two methods of growth rate measurement were used in this paper: one for determining steady-state growth rates when a single value was desired (e.g. reporting values in the text and determining values to be used in modeling) and one for calculating instantaneous growth rates over time (e.g. Fig 2D and 2G).
>
> " To calculate steady-state growth rate values, optical density data was collected and background-corrected as described above. For Aci2 and Pa on alanine and/or glutamate optical density data to be used for this purpose was collected at 400nm. We defined 4.5-hour windows when optical density was above the noise level of our machine but no diauxic shifts had begun and when growth appeared as close to linear on a log-linear plot as available in the data. We then used a Thiel-Sen estimator in which a slope was fit through every pair of points, and the median slope was used as the reported growth rate. The reported uncertainty is the standard deviation of the set of slopes. The Pa monoculture data contained some minor optical density artifacts (Appendix Fig S5), but the obtained rate value of 0.67 hr^{-1} was a good overall fit to the data (Fig EV1C).
>
> " To extract instantaneous growth rates over time, a linear least square fit was performed on population sizes from a 30-minute rolling window (7 measurements) and the slope of this fit was divided by the population size at the center of the window. When plotting, replicates were median filtered to have an single growth rate timeseries for each condition.
>
> " For the survey of additional species and resources, growth rates were determined by manually fitting a line through the data on a log-linear plot. These fits are provided in the Figure 6 Source Data. In some cases, a reliable growth rate could not be obtained (usually due to there not being a sufficient period of steady-state exponential growth to work with), and the data and corresponding competitions were excluded from the analysis. "

6. The fits in Supplementary Fig 43 don't always look reliable. It will be good to have some kind of goodness of fit estimates.

We agree with the reviewer that the fits originally presented in Supplementary Fig 43 were not always reliable. These fits are no longer a part of the paper. Instead, we have performed a substantial set of additional experiments to collect better data for determining these fits. We greatly thank the reviewer for highlighting this lack of reliability as we believe the old fits were the one weak component of the original manuscript and the new fits make our paper considerably more rigorous as a whole.

The new experiments involved varying the resource supply ratio, using 4:1, 1:1, and 1:4 supply ratios for all species in all two-resource environments. Collecting data from 4:1 and 1:4 environments produced growth curves in which diauxic shift occurred earlier and species had substantially more post-shift growth than in the 1:1 condition. In all cases we fit diauxic lag times to the supply ratio in which the diauxic shift occurred the earliest (so as to have the most post-shift growth to work with). As noted above in our response to the reviewer's Point #1, we used the exact same methodology as for the Aci2 and Pa on alanine and glutamate fits except that the post-shift g_{SS} value was unconstrained, making these two-parameter fits. The new fits are presented in the Figure 6 Source Data. We believe these fits are much more reliable and expect the reviewer will agree.

The new data and updated fits changed our results slightly. Working with the original data, we had seen the seen coexistence in 39% of cases in which the slow-grower was the fast-switcher and in 15% of cases in which it was not. With the new data and improved fits, those numbers have now become 68% and 18%. So, coexistence is now seen to be nearly 4x more likely if the slow-grower is the fast-switcher, compared to having previously been about 2.5x more likely.

	Frequency of coexistence	
	Slow-grower is fast-switcher	Slow-grower *not* fast-switcher
Original data	39%	15%
New data and improved fits	68%	18%

7. The slow growers that are slow-switchers should ideally always lose in competition. It is surprising that in Fig 6, there are few such strains that either coexist or even win the competition. Some insights into this will be helpful.

We agree with the reviewer that, yes, it is surprising that slow-growers that are also slow-switchers could competitively exclude fast-growers/switchers. Monoculture growth rates and lag times are, however, just two parameters of the many that contribute to competitive fitness. Four of the five cases involve Arth being the fast-grower and fast-switcher but nevertheless being excluded by its competitor.[4] Arth tends to have long initial lags and slow post-shift growth rates (see Figure 6 Source Data). To maintain focus on the two-parameter tradeoff between growth rate and diauxic lag, we excluded initial lags and post-shift growth rates from our characterizations, but these are expected to also be important determinants of competitive fitness. It is, if anything, surprising that these additional factors could so often be neglected in favor of only considering initial two-resource growth rates and diauxic lag times when predicted competitive outcomes.

This observation is now noted in the Results (line #294 in subsection "Coexistence is more likely when slow-growers are fast-switchers"):

[4] The four specific cases are Pa and Arth on fructose and citrate, Ka and Arth on fructose and citrate, Ka and Arth on fructose and aspartate, and Ka and Arth on glucose and citrate. The case not involving Arth is Pa and Ka on fructose and citrate.

“ Surprisingly, there were five cases in which the slow-grower was the slow-switcher but actually excluded the fast-grower. In four of these cases the excluded fast-grower was Arth, which tended to have long initial lags and slow post-shift growth rates. These unexpected exclusions highlight how other elements of species' monoculture growth dynamics can also be important determinants of competitive fitness. The relative importance of initial and diauxic lags and single- and two-resource growth rates on determining competitive outcomes is explored in the Appendix, but the higher-dimensional complexity of these tradeoffs meant a thorough, integrated study needed to be left for future work. ”

8. Authors should comment on if acidic carbon sources change the pH of growth media (M9), and also if it affects bacterial growth profiles.

The pH of the media is now briefly noted in the Materials and Methods (line #581 in subsection “Species and Media”):

“ The pH was not adjusted after preparing the solutions, but all competition media consistently had pH 6.9 ± 0.1 with the exception of citrate-containing media, which had pH 6.5 ± 0.3. ”

Because pH was nearly constant across media, we did not investigate what effects the small variation may have had on bacterial growth profiles.

9. Are the colonies for all different bacteria morphologically different to be distinguishable in a competition experiment? If yes, it will be good to mention this, and if possible, a photograph of plate with different bacterial colonies may be helpful.

Yes, the colonies are all morphologically distinct. The following image and caption are now provided as Appendix Figure S13, which is referenced in the Materials and Methods (line #601 in subsection “Coculture Competitions”):

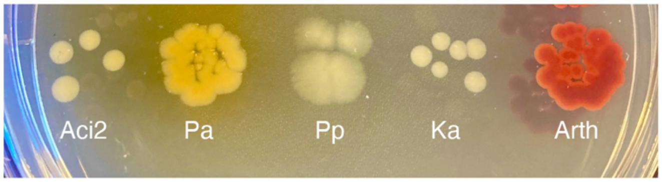

Appendix Figure S13. Images of the colonies formed by each of the five species. The color difference between Aci2 and Ka is becomes more apparent under a transmitted light microscope with Aci2 being considerably more opaque than Ka.

Reviewer #3:

Microbes often co-exist as complex mixed populations. However, the establishment and maintenance of such co-existence is not always trivial, and many of the underlying mechanisms and evolutionary forces remain elusive. This study examines how coexistence is established in multi-resource environments. A clever set of experiments reveal that overall fitness of individual species in a mixture is influenced by both the growth rate in stable conditions as well as the lag times during environmental shifts. A mathematical model further formalizes this effect and shows that the tradeoffs can help support coexistence. A more complex model that also accounts for metabolic strategies further generalizes the findings.

As such, this study furthers our general understanding of microbial ecology by offering a mathematical framework to model, understand and predict the development and evolution of complex microbial communities, with a specific emphasis on the effects of fitness in stable environments vs the speed at which cells adapt to environmental changes (specialist vs generalist startegies).

While I generally like study very much, I do have a few questions and recommendations.

We thank the reviewer for their high opinion of our paper. We are glad they appreciated both our experimental designs and our modeling results. The reviewer's questions helped us identify areas in which our paper lacked clarity and improve our manuscript with better explanations of our methodological decision-making and interpretation of our results, which we are sure many readers will benefit from.

Minor comments:

1. In the methods section it is not specified with which machine the OD is measured and what the detection level or the linear range is of the machine. How was the raw data is processed, for example for the OD measurements, was there a correction for the background?

We thank the reviewer for pointing out this missing information. This has been clarified in the Materials and Methods subsection "Monoculture Experiments" (line #615), part of which now reads:

> " Population size was measured as optical density at either 400nm or 600nm every 5 minutes, using a Tecan Infinite M200 Pro multiplate reader. Which wavelength was used for each experiment is provided in the corresponding figure caption or axis label. The noise level on our machine was approximately ±0.001 OD, and all data presented in the Main Text remained within the linear range. Plates were kept at 25°C with orbital shaking in between OD measurements. For each well, the minimum OD value after median filtering with a bin width of seven measurements was used as a background value and subtracted from the measured values. "

2. Figure 2, for A, C & E the ranges of the x-axis are not consistent. For figure 2E and figure3C there x-axis range differs which is not addressed in the text. It might be important to highlight that when the diauxic lag occurs for Pa is different for the model compared to the experimental data.

   We thank the reviewer for noticing this discrepancy and for encouraging us to highlight it in the Main Text, as this detail indeed merits a brief discussion. The small difference in timing has been noted in the Results (line #184 in subsection "Simple growth-lag model reproduces monoculture dynamics") with additional text:

   > " The timing of Pa's diauxic shift was, however, approximately two hours earlier in the modeling than in the experiments. One hour of this offset is accounted for by the small initial lag that Pa displayed in the experiments (Fig 2A). The remaining hour could be explained by a variety of hypotheticals, such as a slightly density-dependent growth rate (Fig EV3C). Further exploration and additional modeling complexity could have produced a closer fit, but the most significant features are already captured by the presented model and any additional complexity would have diminished interpretability of later results. "

3. Top figure of 2A only the first 8 hours are shown. Is there a specific reason why the growth rates are calculated before 8 hours and an OD below 0.1? is this OD above the detection level of the machine or above the background level? Because if you look at the bottom figure of 2A for the full growth curves (here the x-axis goes from 6 to 18 hours) it seems that the maximal growth rate reached is higher for Pa then for Aci2.

9. Line 335: I think it could be useful to incorporate the initial lag, since Pa really seems to be growing a lot slower in the beginning, and eventually catches up. Also, in figure 2B and 2D you could see a difference in their initial lag phase +- 4hours. But I think this depends on how you define the lag phase. I would be a bit clearer on how you define the lag phases .

   (As Minor Comments #3 and #9 are similar questions with essentially the same response, we have grouped them here to avoid repetition.)

   We thank the reviewer for both of these comments. There are indeed peculiarities in the Pa optical density data that warrant explicit acknowledgement and discussion, which have now been added to the manuscript. In short, we intentionally used only the first 8 hours of data in the top (now left) half of Figure 2A as this window avoids experimental artifacts related to variations in Pa's *per biomass* optical density. Those artifacts could create the impression that Pa either has a long initial lag that stretches almost until its diauxic shift or a growth rate that suddenly increases as it enters its diauxic shift. We have chosen to use the growth rate value that Pa maintains steadily and that provides a good overall fit to 10 hours of growth rather than a higher value that is reached only briefly and suspected to be an artifact.

   We have added two figures that help clarify Pa's optical density curves. The first that we wish to draw the reviewer's attention to is Appendix Figure S5:

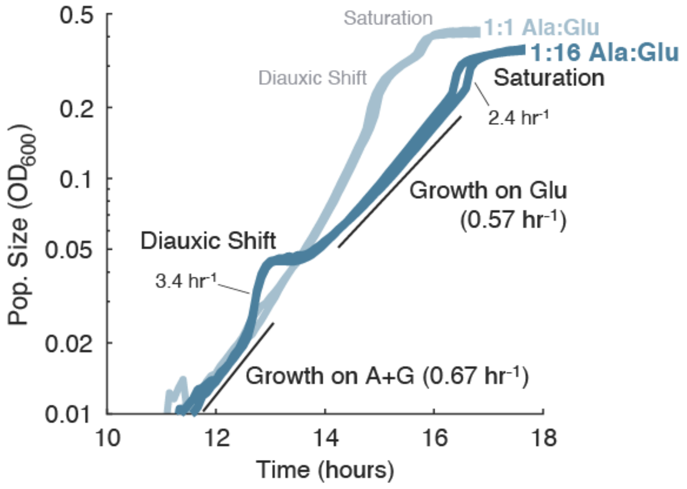

Appendix Figure S5. Small spikes in Pa's optical density at the onset of its diauxic shift and at saturation appear to be artifacts and can be easily accounted for in the data analysis. Shown is an annotated growth curve for Pa growing in a 1:16 Ala:Glu environment (dark) with a growth curve for Pa growing in a 1:1 environment shown for comparison (light and behind). Periods of steady-state growth can be seen first in the two-resource environment and later on the remaining glutamate. Pa's steady-state growth rate on the remaining glutamate matches the value of 0.57 hr^{-1} from single-resource experiments (Fig EV1A). In addition to periods of steady growth, sudden spikes in optical density are also clearly visible. If these spikes corresponded to ordinary growth (i.e. increase in biomass), Pa's growth rate would reach as high as 3.4 hr^{-1} at the time of its diauxic shift. This is implausible, so these spikes must be changes in *per capita* or *per biomass* optical density. We did not study these spikes in optical density any further, but sudden increases of similar magnitude to the optical density of *Pseudomonas* have been previously observed and linked to changes in cell morphology resulting from environmental perturbations.[1] For fitting Pa's lag times, the time for Pa to reach its steady-state growth rate (and not the time until the spike in optical density at saturation) is what's relevant (Materials and Methods). The greatest uncertainty in the Pa lag time fits comes from determining at what time and optical density to define the onset of Pa's diauxic shift. In the reported fits (Appendix Fig S4) we have defined the onset such that we obtain lag time values in the middle of the possible range. Other choices could have affected Pa's lag times by up to +/-20 minutes, but Pa's lag times would still be very short compared to Aci2's and reasonably approximated by a constant value of 1 hour.

Appendix Figure S5 was added primarily to explain how we fit Pa's diauxic lag times, but it does include useful insights for addressing the reviewer's current questions. Specifically, this figure illustrates how Pa has periods of steady exponential growth in the two-resource environment and later on the remaining glutamate along with spikes in optical density at the onset of its diauxic shift and at saturation. Because the spikes in optical density would correspond to growth rates as high as 3.4 hr^{-1} (doubling times as fast as 12 minutes from a species whose doubling times are otherwise around an hour), we cannot label these spikes as ordinary growth. Instead we conclude that these spikes must be changes in *per biomass* optical density, perhaps the result of pigment production or a rapid change in cell morphology.[5]

---

[5] We have not determined the source of Pa's sudden increases in optical density nor of its signs of variable *per biomass* optical density in general. Pa does produce an orange pigment that becomes noticeable around the time it saturates, but it is unclear if this would be sufficient to explain the increases in OD. The supernatant at the end of experiments lacks sufficient optical density for an excreted pigment to be the source, but some pigment appears to be contained with Pa itself. Another hypothesis is that Pa may respond to sudden nutrient limitation by changing cell morphology or size in a way that increases optical density. Sudden increases to the optical density of *Pseudomonas* in changing

This conclusion that some features of the Pa optical density data do not correspond directly to changes in growth rate complicates the determination of Pa's growth rate. To illuminate our handling of these complicates, we now provide Figure 2A for the reviewer's reference along with Figure EV1C, which shows in its bottom half the extension of Pa's growth rate fit through the entirety of the data:

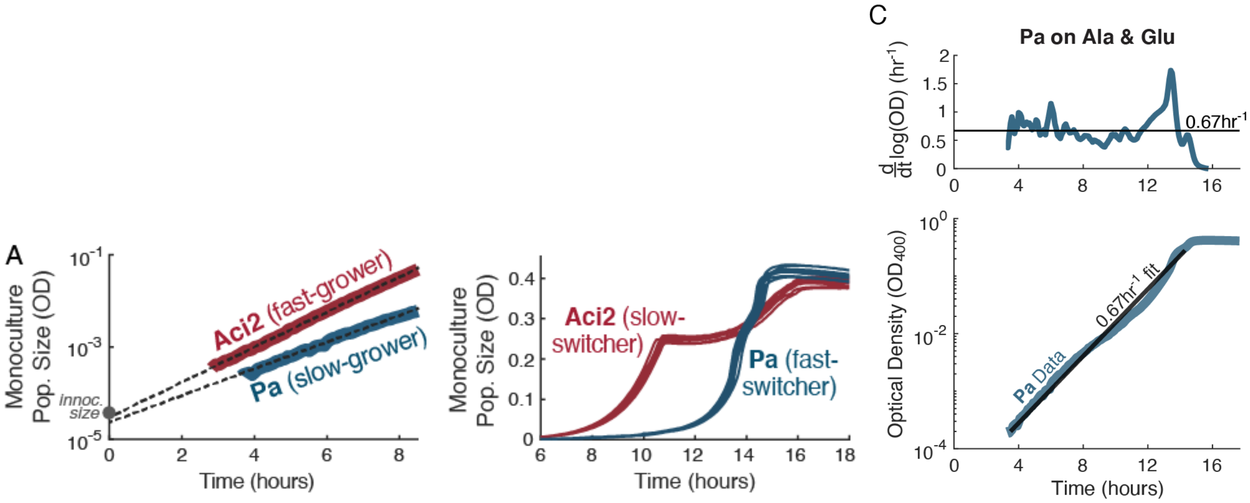

Left and center: **Figure 2A.** Right: **Figure EV1. Pa is the single-resource slow-grower and should be considered the slow-grower despite some fluctuations in its apparent growth rate.** C. Pa's two-resource growth rate fit from Fig 2A extended across the entirety of the data. Although Pa's measured growth rate has some small fluctuations, the growth rate fit of 0.67 hr^{-1} is a good overall fit. The spike in growth rate around 13 hours is discussed in Appendix Figure S5, which also presents reasons why Pa's optical density may not be a constant function of its biomass or population size and why modeling the small variations would likely be overfitting to experimental artifacts and not actual growth dynamics.

As can be seen in Figure EV1C (and particularly in the top half), Pa's optical density grows at a rate close to the 0.67 hr^{-1} fit up until 8 hours into the experiment. At this point, the growth of Pa's optical density actually briefly decreases and then increases, being again close to the 0.67 hr^{-1} fit for hours 9.5 through 12 of the experiment. Pa's optical density then suddenly spikes around 13.5 hours into the experiment, with ramp up the largest spike occurring between hours 12 and 13 of the experiment. This series of events creates a long list of features that *could* be included in our model for Pa, but including all of them would significantly complicate our modeling. Additionally, knowing that Pa's optical density must be a variable function of its biomass in at least some cases suggests an overly detailed model – for example one with a density-dependent growth rate – would be as likely to be an overfit to experimental artifacts as a true capture of Pa's growth dynamics.

For the above-listed reasons, we chose to fit a singular growth rate to Pa's growth dynamics that would be a good fit to the entirety of the OD curve. The longest sustained period of exponential growth at a constant rate was between approximately hours 4 and 8 of the experiment so this period was used to fit a growth rate of 0.67 hr^{-1}. To avoid prompting a long tangential discussion in the

environments have been previously observed and linked to rapid changes in cell morphology (Bernhein 1963). If the reviewer is interested in this phenomenon, we suggest *Pseudomonas veronii* (ATCC 700474) as a species with a particularly pronounced OD spike at saturation and the onset of diauxie for an investigation.

Main Text, we cut the left half of Figure 2A off at 8.5 hours. Figure EV1C, however, is now referenced in the caption for Figure 2A and shows this fit extended across the entirety of the Pa experimental data. As we have acknowledged already, not every feature of the Pa optical density data is captured by assuming exponential growth at a constant rate, but Figure EV1C suggests 0.67 hr^{-1} is good overall representation of Pa's growth in the two-resource environment.

Regarding the species' initial lag times, we define lag time as the time it takes a species to reach its steady-state growth rate. The left half of Figure 2A shows the growth rate fits extrapolated backwards and the initial population size. If the same quadratic growth rate recovery shape as used for the diauxic lags were used here, Aci2's lag time would be 23 minutes and Pa's would be 42 minutes. These fits mean the difference in initial lag times is only 19 minutes, so we did not consider it worth included in the model as this inclusion would have had a very minor effect, especially relative to the complication it would add.

Much of the above discussion has been incorporated into the captions of Figure EV1C and Appendix Figure S5 (above). These figures are referenced in a few places:

- In the caption to Figure 2 the following sentence has been added:

  " More detail on the Pa growth rate fit is provided in Fig EV1C. "

- In the Materials and Methods (line #640 in subsection "Growth Rate Measurements"):

  " The Pa monoculture data contained some minor optical density artifacts (Appendix Fig S5), but the obtained rate value of 0.67 hr-1 was a good overall fit to the data (Fig EV1C). "

- In the Materials and Methods (line #672 in subsection "Lag Time Measurements"):

  " Pa has a small spikes in optical density at the onset of its diauxic shift and at saturation (Appendix Fig S5). These spikes were ignored when fitting the Pa lag times such that lag times correspond to the time it takes Pa to reach its steady-state growth rate not the maximum observed growth rate as this is believed to be an artifact. We did not engage in a mechanistic study of these spikes in optical density, but *Pseudomonas* have been previously observed to rapidly increase per capita optical density under environmental changes due to morphological changes (Bernheim 1962). "

4. Figure 2E and 2F, the diauxic lag time decreases the closer you get to the resource ratio of 2:3 alanine:glutamate. It could be interesting to model this decrease in lag rather than taking a constant lag duration. If you would do that I think figure 3C might resemble 2E even better since the saturation will be reached sooner the closer the ratio is to 2:3. It also seems from figure 2E the closer the resource ratio becomes to 2:3 the less the actual growth rate decreases.

We thank the reviewer for this suggestion and agree that, yes, it would create a slightly closer resemblance between Figures 2 and 3. We are, however, declining to make the change to our modeling for a combination of reasons:

i. Around the 2:3 ratio Aci2's lag times are an order of magnitude longer than Pa's, so slight changes to Pa's lag times will have little impact on the coculture modeling, so the only significant impact will be to the monoculture modeling.
ii. The impact on the monoculture modeling will primarily be noticeable as a slight curvature to the time at which Pa saturates, and the modeling of this curvature would not provide our readers with any particular insight into the nature of diauxic lags and would actually disguise the invariance of the time at which Pa saturates under the current modeling assumptions.
iii. With an already close resemblance between the monoculture experiments and modeling reproduction, we are resistant to adding any additional complexities that might complicate the interpretability of our results.

We do thank the reviewer for the suggestion though, as we are glad to have thought through and confirmed our reasoning for using a constant Pa lag time.

5. Figure 5B & C, would it be useful to also assess the quality of the model by comparing the prediction of the composition after 7 days to the experimental data instead of using the stable state prediction? Still show the stable state prediction since that on itself is interesting.

This has been added as Figure EV3A (below) and is also provided below for reference by the reviewer (along with the steady-state prediction and experimental results for easy comparison):

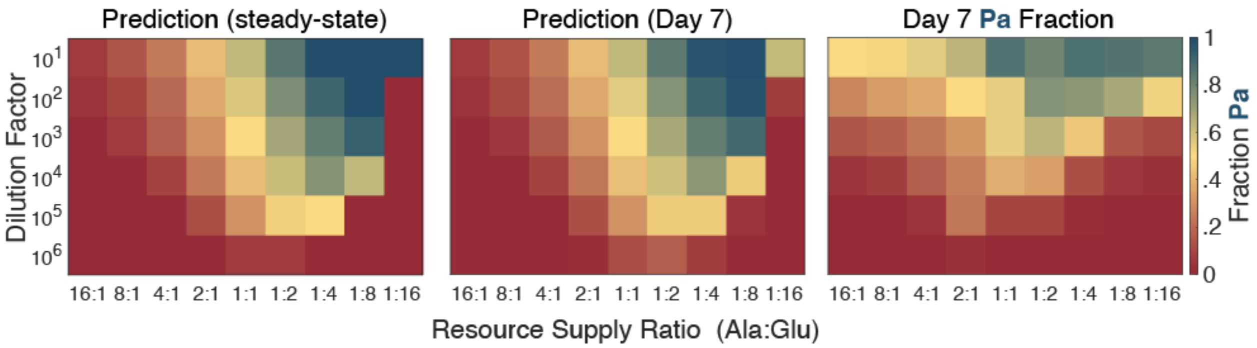

The steady-state and Day 7 predictions are almost identical except at the condition of a 10^1 dilution factor and 1:16 A:G supply ratio. At this condition, Pa excluding Aci2 is a universal attractor of the system (except for an initial fraction Pa equal to zero) but the dynamics are very close to neutrally stable such that equilibration happens on a time scale longer the seven days. At all other conditions, however, the steady-state and Day 7 predictions are very nearly the same. We have chosen to stick with the steady-state prediction in the main text as being able to reference conditions in which Pa or Aci2 are predicted to eventually go entirely extinct is useful in the narrative and because the data shown in Figure 5A suggests the experimental cultures have reached equilibrium by Day 7.

Figure EV3A contains the above-mentioned modeling results, while EV3B is provided in response to a comment from another reviewer:

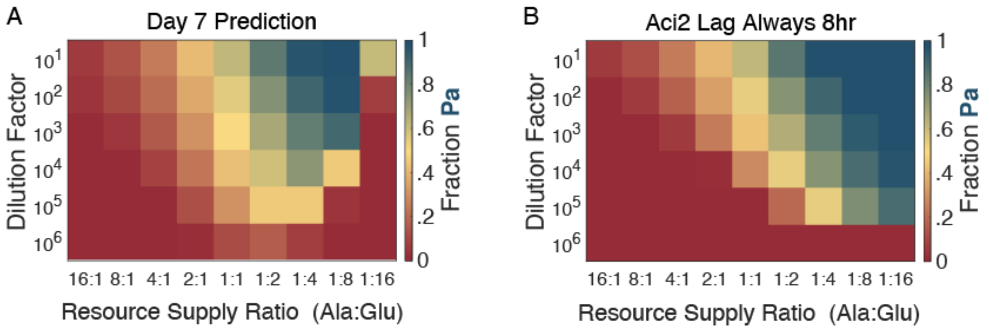

Figure EV3. Predicted mean fraction at Day 7 and predicted steady-state if Aci2's lag did not vary with resource supply.

A. Predicted mean fraction Pa after seven dilution cycles of competition. Shown is the average fraction Pa from competitions started with a Pa fraction of 0.1, 0.25, 0.75, and 0.9 (same as in experiments).
B. Predicted steady-state fraction Pa if Aci2's lag did not vary with resource supply but was instead constant at 8 hours. The value of 8 hours was chosen for being close to Aci2's mean lag time of 9±1 hours and providing a close fit to the experimental results.

6. For supplementary figure 7, where you show the 'observed vs predicted' is the predicted again the stable state prediction? It is also not clear how many points are around the origin. It might be good to show a residual plot, this will show for each combination the error of the prediction separately.

We thank the reviewer for encouraging us in this and the next point to provide more detail as there are indeed more data points stacked at the origin than the viewer can distinguish. To answer the reviewer's first question: yes, this is again the steady state prediction, and this has been clarified in the figure caption.

To address the rest of this comment as well as the next, we have added a few additional plots to Appendix Figure S10 (below; previously Supp Fig 7). In particular, to address the reviewer's concerns about the number of stacked point around the origin, panel B now shows the observed fraction Pa for all conditions in which Pa was predicted to go extinct with manual spacing in the horizontal direction to separate data points. We have also added the requested residual plot as panel C:

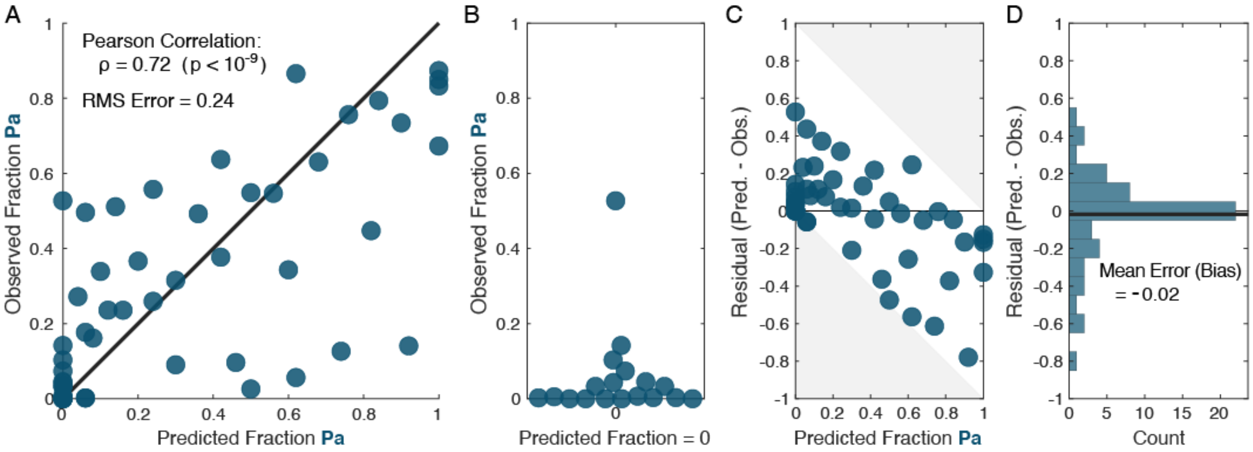

Appendix Figure S10. Quantification of Aci2 and Pa on alanine and glutamate model prediction vs experimentally observed outcome. **(A)** Steady-state predicted fraction Pa vs experimentally observed fraction Pa for all resource supply ratios and dilution factors tested. **(B)** Observed fraction Pa for all cases in which Pa is predicted to go extinct. Horizontal spacing is added to separate the data points that are otherwise stacked (e.g. in the bottom left corner of **A**). **(C)** Residual plot of the same data. Shaded gray regions are the disallowed regions that would require an observed fraction less than zero or greater than one. **(D)** Histogram of the residuals. Dark horizontal line near center indicates the mean residual, which is a species fraction of -0.02.

7. From supplementary figure 7 it also seems that the model is good at predicting exclusions but performs less well at predicting the coexistences. It might also be interesting to give the the actual correlation value in addition to the p-value.

   We have added the correlation coefficient to the list of statistics we provide. The Pearson correlation coefficient is 0.72 ($p < 10^{-9}$). The histogram of the residuals in Appendix Figure S10D (above) shows no overall bias towards under- nor over-predicting the fraction Pa. We do note, however, that the intention of our model was to predict qualitative trends in community composition, rather than being an attempt at a close quantitative fit.

8. Line 203: it is stated that you predict 32 coexistence out of 54, but they do not always agree with the experimental data. This merits more discussion I think.

   The parenthetical "(32/54 in model, 42/54 in experiment)" has been changed to

   > " (32/54 in model and 42/54 in experiment, with 30/42 experimental observations of coexistence correctly predicted) "

   to help highlight that the predicted coexistences do not always agree with the experimental data. We believe sufficient discussion of the discrepancies between the model and experiment is provided two paragraphs later ("The model did not, however, capture all the experimental observations…"), so we have not added additional discussion at the initial statement of the number of predicted vs observed instances of coexistence.

9. Line 335: I think it could be useful to incorporate the initial lag, since Pa really seems to be growing a lot slower in the beginning, and eventually catches up. Also, in figure 2B and 2D you could see a difference in their initial lag phase +- 4hours. But I think this depends on how you define the lag phase. I would be a bit clearer on how you define the lag phases .

   (Due to similarity, Minor Comment #9 was grouped with Minor Comment #3 above. Please see above for our reply.)

10. Is it necessary to square the (t-tdep)/tlag,μ since how I understand it this part is only activated when the resources are depleted and tdep will always be smaller or equal to t. So you would never have the risk of having a negative value. Or is there another reason to square it, eg a better fit?

    We thank the reviewer for asking this question as we realized the answer was not provided in the original manuscript. In short, the t/t_{lag} term was squared solely to produce a better empirical fit to the monoculture data. A linear time dependence was simply not a good fit to the monoculture data (Appendix Figure S2C and S2F below). Because a quadratic was being used to fit lag times to the monoculture data it was also used in the coculture modeling for consistency.

    If the reviewer would like additional detail:

    The quadratic time-dependence was chosen for having a better empirical fit to Aci2's monoculture data than the two simpler options of (i) a "sharp" lag with no growth for t_{lag} and then an immediate recovery to the full growth rate and (ii) a linear time-dependence. The comparative empirical fits are provided in Appendix Figure S2, which is provided below. As can be seen throughout the data (for example in Figures 2A-B and Appendix Figure S2), Aci2 experiences a gradual growth rate recovery after its diauxic shift. This gradual recovery makes a sharp lag a poor fit to the data. In the cases of 1:8 through 1:16 A:G, a full or nearly full growth rate recovery is realized, so a sharp lag could be fit asymptotically. But for all other conditions using the sharp-lag recovery shape to fit growth rates would involve considerable extrapolation and guesswork, leading us to consider other recovery shapes for fitting lag times to the data (Appendix Figure S2E).

    The next simplest recovery shape we considered was a linearly time-dependent growth rate. The results of using this recovery shape can be seen in Appendix Figure S2C. The fit appears good for 1:8 and 1:16 A:G (largely because the growth rates fully recover, so the $t > t_{\mathrm{lag}}$ limit is realized). But, again, for other conditions the fit is worse and leads to questionable results. Specifically, for the intermediate conditions of 1:2 and 1:4 A:G, the linear recover shape first displays too rapid and then too slow a recovery, leading to significant uncertainty as to which part of the curve should be fit. For the conditions of 2:1 and 1:1 A:G, the data only or mostly includes the period in which the recovery shape displays too rapid a recovery, so attempts to fit a lag time would produce estimates that are artificially high. For these reasons, the linear growth rate recovery shape could not be used.

    The quadratic growth rate recovery shape was chosen as an in-between and also the next obvious step up in complexity. It initially predicts a slower recovery and smaller population sizes than the linear recovery shape (an improvement over that recovery shape's biggest limitation) and later

predicts a smooth recovery as t approaches t_{lag} (an improvement over the sharp-lag recovery shape's biggest limitation). Additionally, the plots of growth rate over time in Appendix Figure S6 show a recovery that does appear to reassemble a quadratic function. Appendix Figures S2D and S3 show the specific fits, which appear satisfactory for all conditions. It was for this combination of reasons that the quadratic growth rate recovery shape was chosen.

We have briefly clarified the above point in the Results (line #138 in subsection "Aci2 is the fast-grower, but Pa is the fast-switcher"):

> " For fitting we assumed growth rates recovered proportional to the square of the time since the resource depletion (Materials and Methods). This recovery shape was chosen for having a better empirical fit to Aci2's monoculture growth curves than other shapes considered (Appendix Fig S2) while maintaining a simple, single-parameter functional form. "

The methods section referenced in the above paragraph a new dedicated subsection "Lag Time Measurement", part of which (at line #653) reads:

> " Lag times were fit using a growth rate recovery shape in which growth rate recovered proportional to the square of the time since the resource depletion,
>
> $$g(t) = g_{\text{SS}}\left(\frac{t - t_0}{t_{\text{lag}}}\right)^2 \quad \text{if} \quad t < t_{\text{lag}}\,, \quad \text{else} \quad g(t) = g_{\text{SS}}\,,$$
>
> where g_{SS} is the steady-state growth rate, t_0 is the resource depletion time, and t_{lag} is the species lag time. This recovery shape was used because it was a single-parameter fit that was a close empirical match to post-shift recoveries observed in the monoculture data, particularly in comparison to other recovery shapes considered (Appendix Fig S2)."

And, Appendix Figure S2 is:

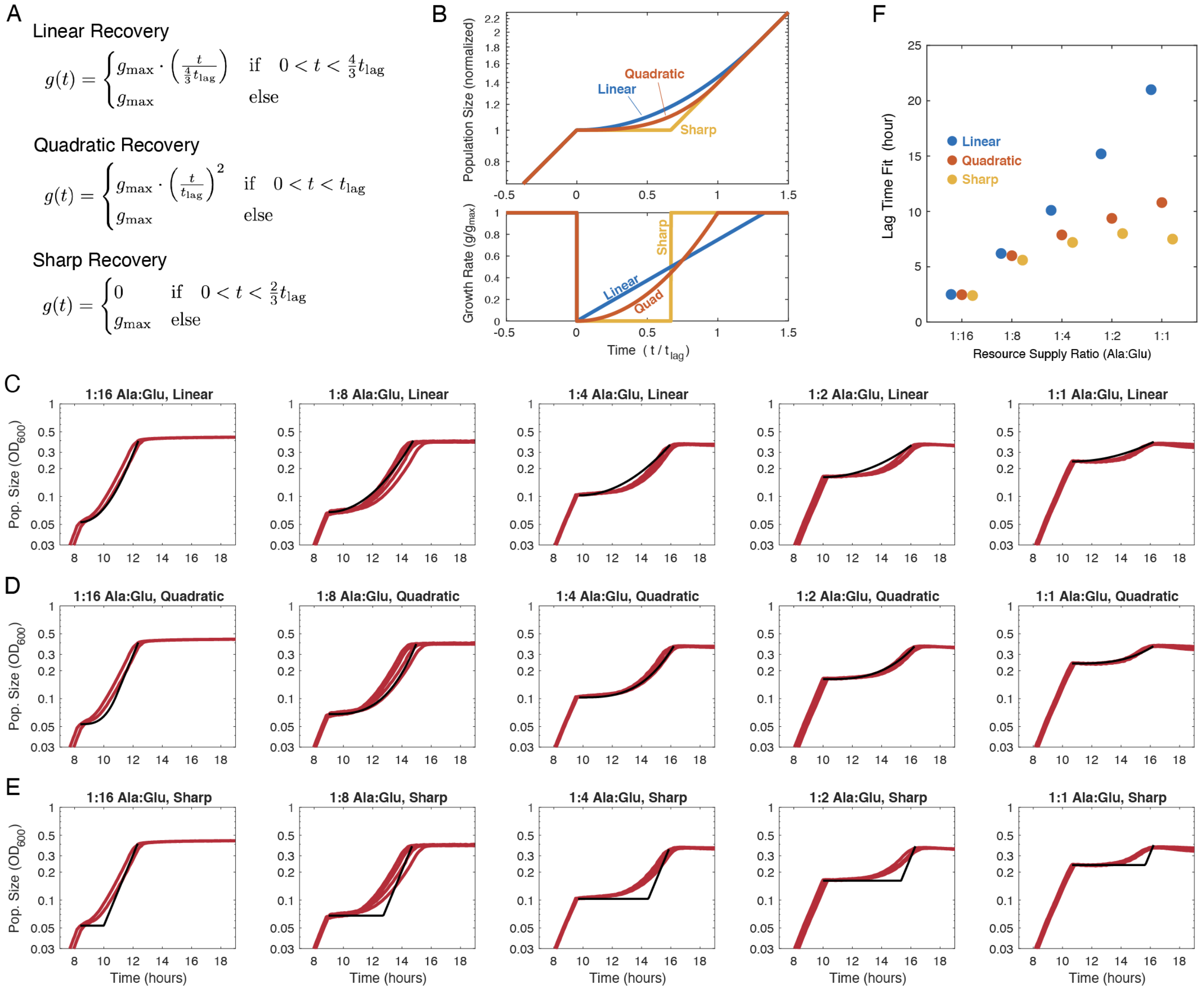

Appendix Figure S2. A quadratic growth rate recovery shape allows for more confident lag time fits than a linear or a sharp recovery. (A) The equations governing three simple empirical models for growth rate (and, after integration, population size) over time after a diauxic shift that starts at time $t = 0$. The steady-state growth rate is g_{SS} and the lag time is t_{lag}. The prefactors in front of t_{lag} in the linear and sharp recovery shapes are included to have the population sizes from each shape converge at time $t >> t_{lag}$. **(B)** The growth rates (bottom) and population sizes (top) derived from those shapes. The top panel illustrates how the quadratic growth rate shape can be thought of as in between the linear and sharp recovery shapes. **(C – D)** Lag time fits using the linear, quadratic, and sharp growth rate recovery shapes. In all fits $g_{SS} = 0.88$ hr^{-1}, so the only free parameter is t_{lag} (fit independently for each recovery shape). The Aci2 optical density data from Fig 2B and EV2A is in the red and the best fit is in black. The quadratic fits in **D** are the same as Fig EV2A and reproduced here to facilitate comparison. **(C)** The linear recovery shape allows for close, confident fits at large glutamate supply fractions (two leftmost panels), but at more equal supply ratios (three center through rightmost panels) it is unclear whether the most appropriate fit would be a long lag time fit that predicts population sizes close to the experimental data before saturation is reached or a shorter lag time fit that would eventually converge towards an extrapolation of the experimental data if saturation were not reached. **(E)** Similarly, the sharp recovery shape can be fit asymptotically at large glutamate supply fractions but becomes more uncertain at more equal supply ratios. **(F)** Comparing the lag times fit using each recovery shape reinforces these considerations. Using the linear recovery shape yields excessively long lag times, while the sharp recovery shape yields lag time values similar to the quadratic model but that actually decrease at the 1:1 Ala:Glu condition (relative to the 1:2 condition) due to the biases of using that recovery shape.

Typo’s etc

1. line 23 missing ‘which’ before ‘must’ the which refers to the dynamics

   We have rephrased this sentence as “These exceptions suggest that dynamics specific to multi-resource environments must be considered to fully understand community assembly.”.

2. line 411 What does DF mean? I Found it o, the supplementary that it is the dilution factor it might be good to quickly state the abbreviation in the main text.

   At that location, “DF” has been replaced by “the dilution factor DF (which ranged from 10 to 10^6 in our modeling and experiments)”.

3. Figure 2B and D and related supplements could use some adaptation to better get the main message across and omit clutter... What I think the main takeaway is from that figure, is that the OD at which the diauxic lag occurs. It might be good to include or switch it with the supplementary figure 2. The supplementary figure is a very nice figure to show the trends at which OD’s the diauxic lag occurs for the different ratios.

   We thank the reviewer for their feedback on Figure 2. After consideration, we agree that the previous version of the figure left the reader with too large a jump from the experimental data to our conclusions about Aci2’s alanine preference and Pa’s resource consumption ratio. We do, however, like to show as close to raw data in our figures as is reasonable. We have, therefore, decided to add the plots from the supplementary figure to main text Figure 2 (as 2C and 2F), while also keeping the plots that were originally 2B and 2D (and are now 2B and 2E). We recognize that including both sets of plots does not solve the issue of clutter within Figure 2 as a whole, so we have also made an effort to reorganize the figure and utilize additional vertical spacing to reduce the feeling of clutter within the figure and benefit its readability. We believe this is a good balance of the various considerations in assembling this figure, and hope the reviewer can agree.

   The new version of Figure 2 is shown below:

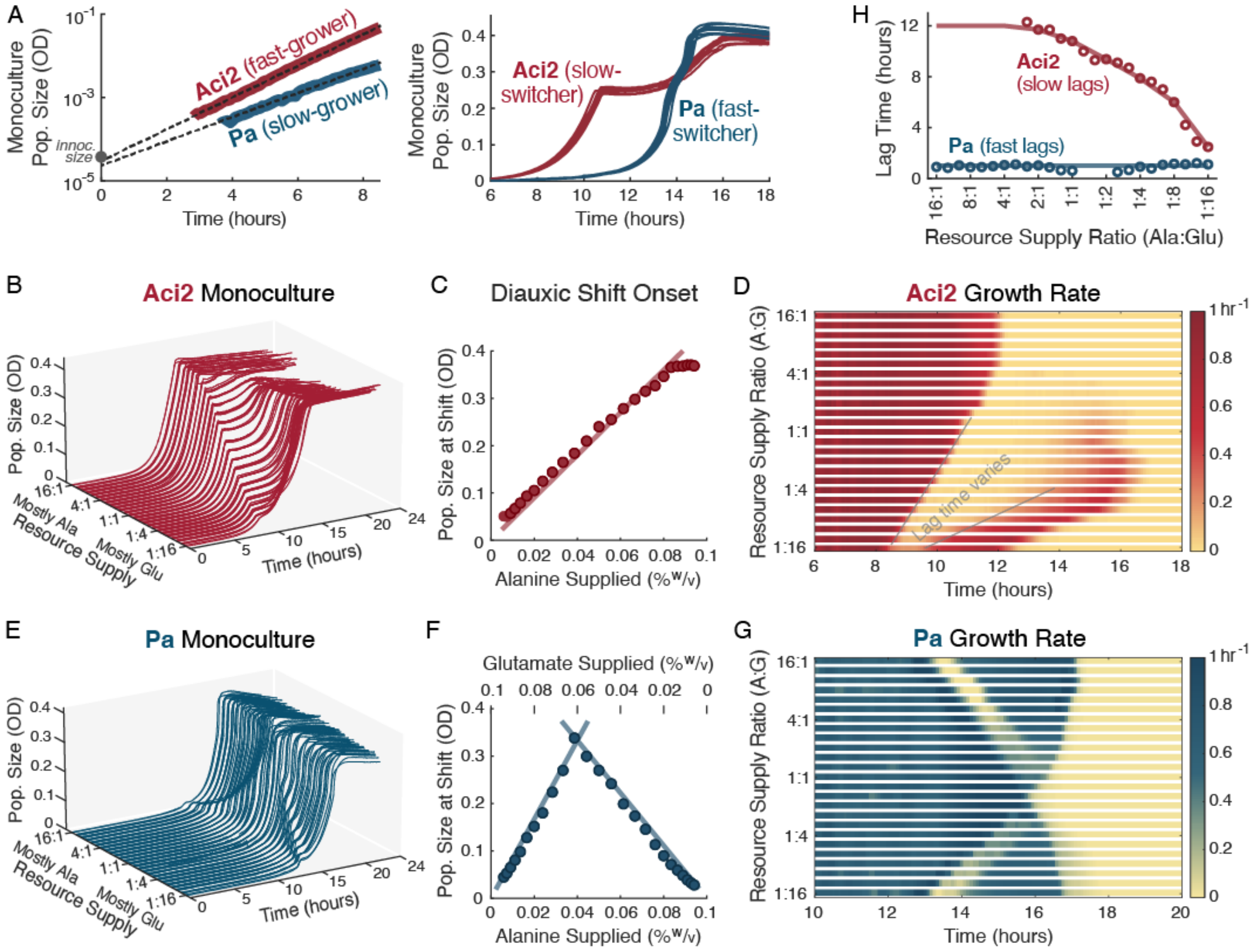

Figure 2. Monoculture growth dynamics reveal Aci2 is a fast-grower but slow-switcher whereas Pa is a slow-grower but fast-switcher.

A. Aci2 and Pa were both grown in monoculture in the two-resource environment. The same data is shown in both plots. The left plot shows the average of eight replicates on a log scale, while the right plot shows each of the replicates on a linear scale. Overlain on the left plot are growth rate fits of $g_{\text{Aci2}} = 0.88\text{hr}^{-1}$ and $g_{\text{Pa}} = 0.67\text{hr}^{-1}$.

B. Aci2 was grown with alanine and glutamate supplied at 25 different ratios from 1:16 to 16:1 with the total supply kept constant at 0.1%w/v (Materials and Methods).

C. The population size at which Aci2's diauxic shift occurred is linearly correlated to the alanine supply, indicating that Aci2 initially consumes almost entirely alanine.

D. Instantaneous growth rates were extracted from the Aci2 monoculture data (Materials and Methods). A variation in Aci2's lag time is clearly visible in this representation.

E. The same monoculture experiments were performed for Pa. The appearance of Pa having a long initial lag is primarily due to the growth that it needs to accomplish before its population size becomes significant on a linear scale as well as a small optical density artifact (Appendix Fig S5).

F. Pa's population size at the onset of its diauxic shift correlates to the supply concentration of whichever resource is supplied in a more limiting amount, indicating coutilization.

G. Instantaneous growth rates extracted from the Pa monoculture data.

H. Diauxic lag times were fit from the monoculture data (Materials and Methods) and are plotted as circles. Lag times could not be fit for conditions with too little growth on the remaining glutamate. The lag times used in the modeling are shown as lines through the data.

Data Information: More detail on the Pa growth rate fit is provided in Fig EV1C. Data in A were collected at 400nm. Data in B–G were collected at 600nm. Example lag time fits are shown in Figure EV2A, and the full set in Appendix Fig S3 and S4. Source data are available for this figure.

4. Not all the colors of the curves in supplementary figure 4 match with the legend colors.

   This figure has been re-envisioned to show greater clarity as to the growth rate at each specific condition and to no longer rely on a colormap and is now Appendix Figure S1:

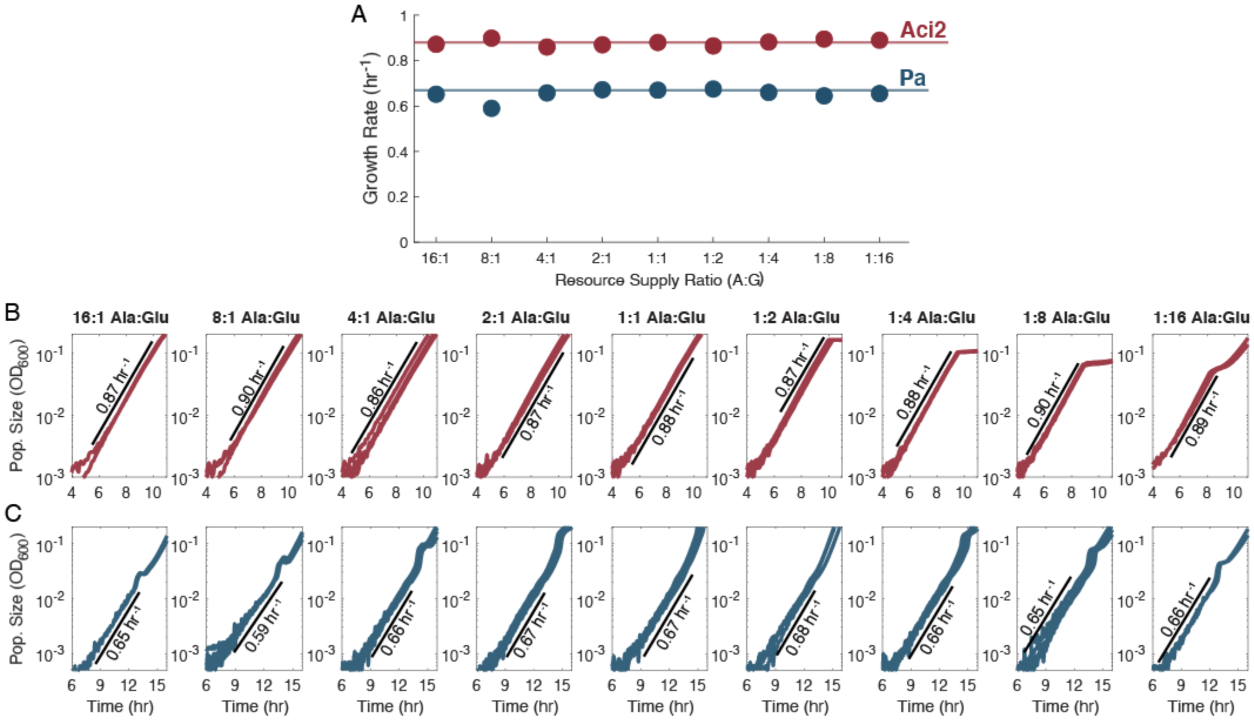

Appendix Figure S1. Two-resource growth rates do not vary with resource supply ratio. (A) Growth rates for Aci2 and Pa at nine different ratios of alanine and glutamate (total supply constant at 0.1\%w/v). Aci2's growth rate fluctuates by +/-0.01 hr^{-1} and Pa's by +/-0.03 hr^{-1} (standard deviations of the nine growth rate fits), compared to uncertainties of approximately +/-0.01 hr^{-1} on each individual fit. **(B)** The Aci2 optical density data from Fig 2B in red with the Aci2 growth rates from **A** in black. **(C)** The Pa optical density data from Fig 2E in blue with the Pa growth rates from **A** in black.

5. Figure 3A ii. Would it be a good idea to keep the inequality signs in the same direction? This is a bit easier for the reader to see immediately that the exponential growth for Aci2 is larger but that it also means that the diauxic lag for the Aci2 is longer.

   We have made this switch and agree that it did make the panel slightly faster to read and process.

6. Figure 3D is missing '(OD) or log(OD)' also in the simulations the populations grow to much higher densities than in the experiments which have an OD of around 0.4. Would it be nice to overlay the simulations with experimental data?

   We thank the reviewer for raising this concern as we realized the units within the modeling were never clearly defined. With the exception of having an explicit time unit, the modeling used dimensionless units. Resources were supplied with total supply concentration of 1 (dimensionless

unit) and species' yields were 1 (dimensionless unit of biomass per dimensionless unit of resource supply). With a slight day-to-day carryover included in the modeling (see below response to #7), populations saturated at $\frac{\mathrm{DF}}{\mathrm{DF}-1} \approx 1$ where DF is the dilution factor. If the reviewer wishes to compare experimental and modeling values: the total resource supply is 0.1%$^{w}/_{v}$ and all yields are approximately $\frac{0.4\ \mathrm{OD}}{0.1\%\mathrm{w/v}}$. Implementing these dimensions would rescale all modeled population sizes by a factor of 0.4 OD, making them directly comparable to the experimental observations. Because modeling units are dimensionless, Figure 3D does not have units of OD.

We have clarified the use of dimensionless units at a few relevant places:

- In the Materials and Methods (line #688 in subsection "Modeling"):

  " Modeling was done using dimensionless resource concentrations and population sizes but with an explicit time dimension (hours). Monoculture and coculture simulations followed the same set of equations. The total resource supply was set to 1 (i.e. $s_{\mathrm{Ala}} + s_{\mathrm{Glu}} = 1$ where s_{Ala} is the alanine supply concentration and s_{Glu} is the glutamate supply concentration), and all yields were implicitly set to 1.

  " For competition simulations, the system was initiated with the resource concentrations, $c_{\mathrm{Ala}}(t)$ and $c_{\mathrm{Glu}}(t)$, equal to the supply concentrations and the total population sizes normalized to the carrying capacity divided by the dilution factor DF (which range from 10 to 10^6 in our modeling and experiments),

  $$n_{\mathrm{Aci2}}(0) + n_{\mathrm{Pa}}(0) = \frac{1}{\mathrm{DF} - 1}$$

  where $\frac{1}{\mathrm{DF}-1}$ appears instead of $\frac{1}{\mathrm{DF}}$ due to the carry capacity being $\frac{\mathrm{DF}}{\mathrm{DF}-1}$ after correcting for the day-to-day population carryover (Appendix)."

- In the caption for Figure 3D the following sentence has been added:

  " Population sizes are presented in dimensionless units (Materials and Methods). "

7. Line 217: At lower dilution fraction does the total population grow to higher OD's or do they stay longer at the carrying capacity? And how does this effect the composition/behavior?

   In our experiments we did not observe any significant change in OD as a function of dilution factor (or resource supply ratio). This is consistent with the population having maximally converted the available resource supply into biomass and simply remaining at carrying capacity until the next dilution.

   Our modeling suggests that time spent at carrying capacity ranged from approximately 8 hour under a 10^6 dilution to approximately 21 hours under a 10^1 dilution. Small changes may occur to the population during this time, and the effect of those changes may vary with time spent at saturation, but, with no evidence of any specific changes and with our modeling being focused on simplicity, we proceeded with an assumption that this phase did not need to be taken into account.

Our modeling does incorporate day-to-day carryover (i.e. the fraction of the previous day's population used as an inoculum contributing to the end-of-day total population size and therefore slightly increasing the carrying capacity), but this never changes the carrying capacity by more than 11%. Specifically, with the dilution factor being DF, the carrying capacity is proportional to $\frac{\mathrm{DF}}{\mathrm{DF}-1}$ as can be obtained by solving $n_{\mathrm{sat}} = \frac{1}{\mathrm{DF}} n_{\mathrm{sat}} + 1$ where DF is the dilution factor, $\frac{1}{\mathrm{DF}} n_{\mathrm{sat}}$ is the day-to-day carryover, and $+1$ represents the additional dimensionless unit of biomass gained over the course of one day.

8. Line 270: In the example with Aci2 becoming the slow grower and fast switcher, is this caused by the other species presence or the other available resources?

We thank the reviewer for this question as it prompted us to reconsider which example would be the most interesting to present. In the original example, both the available resource and the competitor needed to be changed for Aci2 to go from the ast-grower and slow-switcher to the slow-grower and fast-switcher. Upon reconsideration, we have decided it would be more interesting to present an example in which changes to the available resources were enough to switch the relative growth-rate and lag-time orderings of two species. We have thus changed our choice of example to the set of competitions between Pp and Arth and have rewritten the paragraph (Line #302 in Results subsection "Coexistence is more likely when slow-growers are fast-switchers"):

> " The data also contained examples of species being the slow-switcher in some scenarios but the fast-switcher in others. The competitions between Pp and Arth are one set of examples. There were two environments (fructose and citrate and fructose and alanine) in which Pp was the fast-grower but slow-switcher and one environment (fructose and aspartate) in which Arth was the fast-grower but slow-switcher. In all three of these environments the two species coexisted. There were also three environments (glucose and citrate, alanine and glutamate, and alanine and aspartate) in which Pp was both the fast-grower and the fast-switcher, and in all three of these environments Pp excluded Arth. (See Figure 6 Source Data for details.) These examples highlight the importance of the specific environment to the growth dynamics of species and assembly of ecological communities, as well as the power of a single growth-lag characterization to predict community assembly across a wide range of environments. "

We thank the reviewer for asking this question as we believe this set of examples is both more interesting and more straightforward to present than the originally chosen examples.

9. Line 313: I like the idea of the crossfeeding, but why is it not highlighted in the results of the main text as a possible mechanisms to predict the coexistence at low dilutions?

Our limited highlighting of crossfeeding and not mentioning it until the discussion was decided in order to maintain focus on our message. Like Reviewer 1 noted in their Major Point #3, cross-feeding is a heavily studied subject in the field and our paper's novelty comes in part from having

focused on growth dynamics directly on the supplied resources. Crossfeeding may also genuinely not be a relevant phenomenon in this scenario, so speculating too heavily may lead a reader to discount over possibilities or assume we had some reason to favor crossfeeding over alternative explanations.

Additionally, cross-feeding is not as elegantly incorporated into diauxie models as into linear resource consumption and related models, and a thorough investigation of the crossfeeding in this particular interaction would have required substantial additional experiments. This led us to conclude that a discussion of cross-feeding would be best left as a brief mention in the Main Text as anything further would too easily raise additional question and prompt more discussion than we wished to give to this possibility.

10. Line 390: Measuring OD at 400nm or 600nm but it is not always stated when one is used over the other? Also, this is not indicated on the axis of the figures.

The labels ‘400nm’ and ‘600nm’ were removed from the figures to reduce clutter, and we continue to believe this was the best decision. We have, however, stated explicitly in the figure captions when each wavelength was used. The mix of 400nm and 600nm occurred during an attempt to minimize the OD artifacts discussed in response to the reviewer’s Minor Point #3 above. There was, however, no noticeable difference between the results obtained at 400nm and 600nm, so we simply used whichever had the least experimental noise from condensation on the plate lid in the cases in which we had data at both wavelengths.

25th Mar 2022

RE: MSB-2021-10630R, Diauxic lags explain unexpected coexistence in multi-resource environments

Thank you again for sending us your revised study. I would like to apologise for the exceptional delay in getting back to you. This was due to the fact that despite numerous reminders we did not receive a report from reviewer #2, one of the two reviewers who were invited to evaluate the revised study. We have now heard back from reviewer #1 and as you will see below they are satisfied with the modifications made.
In the interest of time we have evaluated your answers to the issues raised by reviewer #2 ourselves and we think that these concerns have been satisfactorily addressed. As such, I am glad to inform you that we can soon proceed with formally accepting the study for publication, pending some minor editorial issues listed below.

- Our data editors have noticed some unclear or missing information in the figure legends, please see the attached .doc file. Please make all requested text changes using the attached file and *keeping the "track changes" mode* so that we can easily access the edits made.

- Please include callouts to Figure 3D and Appendix Figures S1, S6 and S12 in the main text.

- Our data integrity analyst noted an instance figure panel reuse i.e. Figure 5B in Appendix Figure S11. We would ask you to indicate the data/panel reuse in the respective figure legends for transparency.

- I have slightly edited the synopsis and bullet point text to shorten it (see attached file). Could you let me know if it is OK or if you would like to change anything?

Please resubmit your revised manuscript **within one month** and ideally as soon as possible. If we do not receive the revised manuscript within this time period, the file might be closed and any subsequent resubmission would be treated as a new manuscript. Please use the Manuscript Number (above) in all correspondence.

Click on the link below to submit your revised paper.

Link Not Available

Thank you for submitting this paper to Molecular Systems Biology.

Maria Polychronidou, PhD
Senior Editor
Molecular Systems Biology

Reviewer #1:

I thank the authors for their thorough consideration of my comments and corresponding revisions. I believe the revised version of the paper adequately addresses all of my concerns.

The authors performed the requested editorial changes.

1st Apr 2022

Manuscript number: MSB-2021-10630RR
Title: Diauxic lags explain unexpected coexistence in multi-resource environments

Thank you again for sending us your revised manuscript. We are now satisfied with the modifications made and I am pleased to inform you that your study has been accepted for publication.

NOTE: Could you please confirm that you and B. Bloxham are co-corresponding authors as indicated in the submission system? There is no information in the manuscript text itself so we wanted to make sure there are no mistakes.

*** PLEASE NOTE *** As part of the EMBO Publications transparent editorial process initiative (see our Editorial at https://dx.doi.org/10.1038/msb.2010.72), Molecular Systems Biology publishes online a Review Process File with each accepted manuscripts. This file will be published in conjunction with your paper and will include the anonymous referee reports, your point- by-point response and all pertinent correspondence relating to the manuscript. If you do NOT want this File to be published, please inform the editorial office at msb@embo.org within 14 days upon receipt of the present letter.

Should you be planning a Press Release on your article, please get in contact with msb@wiley.com as early as possible, in order to coordinate publication and release dates.

LICENSE AND PAYMENT:
All articles published in Molecular Systems Biology are fully open access: immediately and freely available to read, download and share.

Molecular Systems Biology charges an article processing charge (APC) to cover the publication costs. You, as the corresponding author for this manuscript, should have already received a quote with the article processing fee separately. Please let us know in case this quote has not been received.

Once your article is at Wiley for editorial production you will receive an email from Wiley's Author Services system, which will ask you to log in and will present you with the publication license form for completion. Within the same system the publication fee can be paid by credit card, an invoice or pro forma can be requested.

Payment of the publication charge and the signed Open Access Agreement form must be received before the article can be published online.

Molecular Systems Biology articles are published under the Creative Commons licence CC BY, which facilitates the sharing of scientific information by reducing legal barriers, while mandating attribution of the source in accordance to standard scholarly practice.

Proofs will be forwarded to you within the next 2-3 weeks.

Thank you very much for submitting your work to Molecular Systems Biology.

Maria Polychronidou, PhD
Senior Editor
Molecular Systems Biology

EMBO Press Author Checklist

Corresponding Author Name: William Bloxham	
Journal Submitted to: Molecular Systems Biology	
Manuscript Number: MSB-2021-10630	

USEFUL LINKS FOR COMPLETING THIS FORM
The EMBO Journal - Author Guidelines
EMBO Reports - Author Guidelines
Molecular Systems Biology - Author Guidelines
EMBO Molecular Medicine - Author Guidelines

Reporting Checklist for Life Science Articles (updated January 2022)
This checklist is adapted from Materials Design Analysis Reporting (MDAR) Checklist for Authors. MDAR establishes a minimum set of requirements in transparent reporting in the life sciences (see Statement of Task: 10.31222/osf.io/9sm4x). Please follow the journal's guidelines in preparing your manuscript.
Please note that a copy of this checklist will be published alongside your article.

Abridged guidelines for figures
1. Data
The data shown in figures should satisfy the following conditions:
- the data were obtained and processed according to the field's best practice and are presented to reflect the results of the experiments in an accurate and unbiased manner.
- ideally, figure panels should include only measurements that are directly comparable to each other and obtained with the same assay.
- plots include clearly labeled error bars for independent experiments and sample sizes. Unless justified, error bars should not be shown for technical replicates.
- if n<5, the individual data points from each experiment should be plotted. Any statistical test employed should be justified.
- Source Data should be included to report the data underlying figures according to the guidelines set out in the authorship guidelines on Data Presentation.

2. Captions
Each figure caption should contain the following information, for each panel where they are relevant:
- a specification of the experimental system investigated (eg cell line, species name).
- the assay(s) and method(s) used to carry out the reported observations and measurements.
- an explicit mention of the biological and chemical entity(ies) that are being measured.
- an explicit mention of the biological and chemical entity(ies) that are altered/varied/perturbed in a controlled manner.
- the exact sample size (n) for each experimental group/condition, given as a number, not a range;
- a description of the sample collection allowing the reader to understand whether the samples represent technical or biological replicates (including how many animals, litters, cultures, etc.).
- a statement of how many times the experiment shown was independently replicated in the laboratory.
- definitions of statistical methods and measures:
  - common tests, such as t-test (please specify whether paired vs. unpaired), simple χ2 tests, Wilcoxon and Mann-Whitney tests, can be unambiguously identified by name only, but more complex techniques should be described in the methods section;
  - are tests one-sided or two-sided?
  - are there adjustments for multiple comparisons?
  - exact statistical test results, e.g., P values = x but not P values < x;
  - definition of 'center values' as median or average;
  - definition of error bars as s.d. or s.e.m.

Please complete ALL of the questions below.
Select "Not Applicable" only when the requested information is not relevant for your study.

Materials

Newly Created Materials	Information included in the manuscript?	In which section is the information available? (Reagents and Tools Table, Materials and Methods, Figures, Data Availability Section)
New materials and reagents need to be available; do any restrictions apply?	Not Applicable	

Antibodies	Information included in the manuscript?	In which section is the information available? (Reagents and Tools Table, Materials and Methods, Figures, Data Availability Section)
For **antibodies** provide the following information:<br>- Commercial antibodies: RRID (if possible) or supplier name, catalogue number and or/clone number<br>- Non-commercial: RRID or citation	Not Applicable	

DNA and RNA sequences	Information included in the manuscript?	In which section is the information available? (Reagents and Tools Table, Materials and Methods, Figures, Data Availability Section)
**Short novel DNA or RNA including primers, probes**: provide the sequences.	Not Applicable	

Cell materials	Information included in the manuscript?	In which section is the information available? (Reagents and Tools Table, Materials and Methods, Figures, Data Availability Section)
**Cell lines:** Provide species information, strain. Provide accession number in repository **OR** supplier name, catalog number, clone number, and/**OR** RRID.	Not Applicable	
**Primary cultures:** Provide species, strain, sex of origin, genetic modification status.	Yes	Materials and Methods
Report if the cell lines were recently **authenticated** (e.g., by STR profiling) and tested for mycoplasma contamination.	Not Applicable	

Experimental animals	Information included in the manuscript?	In which section is the information available? (Reagents and Tools Table, Materials and Methods, Figures, Data Availability Section)
**Laboratory animals or Model organisms:** Provide species, strain, sex, age, genetic modification status. Provide accession number in repository **OR** supplier name, catalog number, clone number, **OR** RRID.	Not Applicable	
**Animal observed in or captured from the field:** Provide species, sex, and age where possible.	Not Applicable	
Please detail **housing and husbandry conditions**.	Not Applicable	

Plants and microbes	Information included in the manuscript?	In which section is the information available? (Reagents and Tools Table, Materials and Methods, Figures, Data Availability Section)
**Plants:** provide species and strain, ecotype and cultivar where relevant, unique accession number if available, and source (including location for collected wild specimens).	Not Applicable	
**Microbes:** provide species and strain, unique accession number if available, and source.	Yes	Materials and Methods

Human research participants	Information included in the manuscript?	In which section is the information available? (Reagents and Tools Table, Materials and Methods, Figures, Data Availability Section)
If collected and within the bounds of privacy constraints report on age, sex and gender or ethnicity for all study participants.	Not Applicable	

Core facilities	Information included in the manuscript?	In which section is the information available? (Reagents and Tools Table, Materials and Methods, Figures, Data Availability Section)
If your work benefited from core facilities, was their service mentioned in the acknowledgments section?	Not Applicable	

Design

Study protocol	Information included in the manuscript?	In which section is the information available? (Reagents and Tools Table, Materials and Methods, Figures, Data Availability Section)
If study protocol has been **pre-registered, provide DOI in the manuscript**. For clinical trials, provide the trial registration number **OR** cite DOI.	Not Applicable	
Report the **clinical trial registration number** (at ClinicalTrials.gov or equivalent), where applicable.	Not Applicable	

Laboratory protocol	Information included in the manuscript?	In which section is the information available? (Reagents and Tools Table, Materials and Methods, Figures, Data Availability Section)
Provide DOI OR other citation details if **external detailed step-by-step protocols** are available.	Not Applicable	

Experimental study design and statistics	Information included in the manuscript?	In which section is the information available? (Reagents and Tools Table, Materials and Methods, Figures, Data Availability Section)
Include a statement about **sample size** estimate even if no statistical methods were used.	Yes	Materials and Methdods, Figures, Results, Appendix
Were any steps taken to minimize the effects of subjective bias when allocating animals/samples to treatment (e.g. **randomization procedure**)? If yes, have they been described?	Not Applicable	
Include a statement about **blinding** even if no blinding was done.	Not Applicable	
Describe **inclusion/exclusion criteria** if samples or animals were excluded from the analysis. Were the criteria pre-established?<br><br>If sample or data points were omitted from analysis, report if this was due to attrition or intentional exclusion and provide justification.	Not Applicable	
For every figure, are **statistical tests** justified as appropriate? Do the data meet the assumptions of the tests (e.g., normal distribution)? Describe any methods used to assess it. Is there an estimate of variation within each group of data? Is the variance similar between the groups that are being statistically compared?	Not Applicable	

Sample definition and in-laboratory replication	Information included in the manuscript?	In which section is the information available? (Reagents and Tools Table, Materials and Methods, Figures, Data Availability Section)
In the figure legends: state number of times the experiment was **replicated** in laboratory.	Yes	Materials and Methods, Figures
In the figure legends: define whether data describe **technical or biological replicates**.	Yes	Materials and Methods, Figures

Ethics

Ethics	Information included in the manuscript?	In which section is the information available? (Reagents and Tools Table, Materials and Methods, Figures, Data Availability Section)
Studies involving **human participants**: State details of **authority granting ethics approval** (IRB or equivalent committee(s), provide reference number for approval.	Not Applicable	
Studies involving **human participants**: Include a statement confirming that **informed consent** was obtained from all subjects and that the experiments conformed to the principles set out in the WMA Declaration of Helsinki and the Department of Health and Human Services Belmont Report.	Not Applicable	
Studies involving **human participants:** For publication of **patient photos**, include a statement confirming that consent to publish was obtained.	Not Applicable	
Studies involving experimental **animals**: State details of **authority granting ethics approva**l (IRB or equivalent committee(s), provide reference number for approval. Include a statement of compliance with ethical regulations.	Not Applicable	
Studies involving **specimen and field samples:** State if relevant **permits** obtained, provide details of authority approving study; if none were required, explain why.	Not Applicable	

Dual Use Research of Concern (DURC)	Information included in the manuscript?	In which section is the information available? (Reagents and Tools Table, Materials and Methods, Figures, Data Availability Section)
Could your study fall under dual use research restrictions? Please check biosecurity documents and list of **select agents and toxins** (CDC): https://www.selectagents.gov/sat/list.htm	Not Applicable	
If you used a select agent, is the security level of the lab appropriate and reported in the manuscript?	Not Applicable	
If a study is subject to dual use research of concern regulations, is the name of the **authority granting approval and reference number** for the regulatory approval provided in the manuscript?	Not Applicable	

Reporting

The MDAR framework recommends adoption of discipline-specific guidelines, established and endorsed through community initiatives. Journals have their own policy about requiring specific guidelines and recommendations to complement MDAR.

Adherence to community standards	Information included in the manuscript?	In which section is the information available? (Reagents and Tools Table, Materials and Methods, Figures, Data Availability Section)
State if relevant guidelines or checklists (e.g., **ICMJE, MIBBI, ARRIVE, PRISMA**) have been followed or provided.	Not Applicable	
For **tumor marker prognostic studies,** we recommend that you follow the **REMARK** reporting guidelines (see link list at top right). See author guidelines, under 'Reporting Guidelines'. Please confirm you have followed these guidelines.	Not Applicable	
For **phase II and III randomized controlled trials**, please refer to the **CONSORT** flow diagram (see link list at top right) and submit the CONSORT checklist (see link list at top right) with your submission. See author guidelines, under 'Reporting Guidelines'. Please confirm you have submitted this list.	Not Applicable	

Data Availability

Data availability	Information included in the manuscript?	In which section is the information available? (Reagents and Tools Table, Materials and Methods, Figures, Data Availability Section)
Have **primary datasets** been deposited according to the journal's guidelines (see 'Data Deposition' section) and the respective accession numbers provided in the Data Availability Section?	Not Applicable	
Were **human clinical and genomic datasets** deposited in a public access-controlled repository in accordance to ethical obligations to the patients and to the applicable consent agreement?	Not Applicable	
Are **computational models** that are central and integral to a study available without restrictions in a machine-readable form? Were the relevant accession numbers or links provided?	Not Applicable	
If publicly available data were reused, provide the respective **data citations in the reference list**.	Not Applicable	
